# Serum-dependent transcriptional networks identify distinct functional roles for H-Ras and N-Ras during initial stages of the cell cycle

**DOI:** 10.1186/gb-2009-10-11-r123

**Published:** 2009-11-06

**Authors:** Esther Castellano, Carmen Guerrero, Alejandro Núñez, Javier De Las Rivas, Eugenio Santos

**Affiliations:** 1 Centro de Investigación del Cáncer, IBMCC (CSIC-USAL), University of Salamanca, Campus Unamuno, 37007 Salamanca, Spain; 2 Current address: Signal Transduction Laboratory, Cancer Research UK London Research Institute, 44 Lincoln's Inn Fields, London WC2A 3PX, UK

## Abstract

**Background:**

Using oligonucleotide microarrays, we compared transcriptional profiles corresponding to the initial cell cycle stages of mouse fibroblasts lacking the small GTPases H-Ras and/or N-Ras with those of matching, wild-type controls.

**Results:**

Serum-starved wild-type and knockout *ras *fibroblasts had very similar transcriptional profiles, indicating that H-Ras and N-Ras do not significantly control transcriptional responses to serum deprivation stress. In contrast, genomic disruption of H-*ras *or N-*ras*, individually or in combination, determined specific differential gene expression profiles in response to post-starvation stimulation with serum for 1 hour (G0/G1 transition) or 8 hours (mid-G1 progression). The absence of N-Ras caused significantly higher changes than the absence of H-Ras in the wave of transcriptional activation linked to G0/G1 transition. In contrast, the absence of H-Ras affected the profile of the transcriptional wave detected during G1 progression more strongly than did the absence of N-Ras. H-Ras was predominantly functionally associated with growth and proliferation, whereas N-Ras had a closer link to the regulation of development, the cell cycle, immunomodulation and apoptosis. Mechanistic analysis indicated that extracellular signal-regulated kinase (ERK)-dependent activation of signal transducer and activator of transcription 1 (Stat1) mediates the regulatory effect of N-Ras on defense and immunity, whereas the pro-apoptotic effects of N-Ras are mediated through ERK and p38 mitogen-activated protein kinase signaling.

**Conclusions:**

Our observations confirm the notion of an absolute requirement for different peaks of Ras activity during the initial stages of the cell cycle and document the functional specificity of H-Ras and N-Ras during those processes.

## Abstract 2

Transcriptional and functional analysis reveals that the H-Ras and N-Ras isoforms have different roles in the initial phases of the mouse cell cycle

## Background

The mammalian H-Ras, N-Ras and K-Ras proteins are highly related small GTPases functioning as critical components of cellular signaling pathways controlling proliferation, differentiation or survival. They act as molecular switches cycling between inactive (GDP-bound) and active (GTP-bound) states in a process modulated under physiological conditions by a variety of specific regulatory proteins, including GAPs (GTPase activating proteins) and GEFs (guanine nucleotide exchange factors) [1-3]. Hyperactivating point mutations of these proteins are frequently associated with pathological conditions, particularly the development of various forms of human cancer [4,5]. The three main mammalian *ras *genes appear to be ubiquitously expressed, although specific differences have been reported for particular isoforms regarding their expression levels in different cell types and tissues or their intracellular processing and subsequent location to different subcellular compartments [1,3].

Early studies focusing on the shared sequence homology and identical *in vitro *effector activation pathways suggested that the three Ras protein isoforms were functionally redundant [2,4]. However, many other reports based on different experimental approaches support the notion that these three members of the Ras family may play specialized cellular roles [1,3,6]. Thus, the preferential activation of specific *ras *genes in particular tumor types [4,5], the different transforming potential of transfected *ras *genes in different cellular contexts [7,8], the distinct sensitivities exhibited by different Ras family members for functional interactions with their GAPs, GEFs or downstream effectors [9-15], or differences among Ras isoforms regarding their intracellular processing pathways and their differential compartmentalization to specific plasma membrane microdomains or intracellular compartments [12,14,16-21] provide strong evidence in favor of the notion of functional specificity. The study of Ras knockout strains provides additional *in vivo *evidence for functional specificity. Thus, whereas disruption of K-*ras *4B is embryonic lethal [22,23], H-*ras*, N-*ras *and K-*ras*4A single knockout mice and H-*ras*/N-*ras *double knockout mice are perfectly viable [22,24-26], indicating that only K-*ras *is necessary and sufficient for full embryonic development and suggesting that K-Ras performs specific function(s) that cannot be carried out by either H-Ras or N-Ras. A recent study describing that the knock-in of H-*ras *at the K-*ras *locus results in viable adult mice [27] suggests that the mortality of K-*ras *knockout may derive not from intrinsic inability of the other Ras isoforms to compensate for K-Ras function but rather from their inability to be expressed in the same locations (embryonic compartments) or at the same time (developmental stage) as K-Ras. Finally, additional experimental support for the notion of functional specificity of H-, N- and K-Ras proteins derives from genomic or proteomic profiling of cell lines transformed by exogenous *ras *oncogenes [28-34] or devoid of specific Ras proteins [35]. In particular, our recent characterization of the transcriptional networks of actively growing cultures of fibroblast cells harboring single or double null mutations in the H-*ras *and N-*ras *loci clearly supported the notion of different functions for H-Ras and N-Ras by documenting a significant involvement of N-Ras in immunomodulation/defense and apoptotic responses [35].

It is also well established that Ras proteins play capital roles in regulation of the initiation and progression of the cell cycle [1,3,5,36]. A number of reports have documented the absolute requirement for Ras activity at different points between G0 and S phase, after growth factor stimulation of quiescent, serum-arrested (G0) cells. Indeed, the available experimental evidence indicates that the contribution of Ras activity is absolutely needed for both the initial entry into the cell cycle (G0/G1 transition) and for the subsequent G1 progression, in a process to which multiple Ras effector pathways can contribute [36-41]. However, the exact mechanisms regulating the participation of Ras proteins in cell cycle activation and subsequent progression are still largely unknown. It is also unknown whether the different Ras isoforms play specific or redundant functional roles in those processes.

Our previous characterization of the transcriptional profiles of unsynchronized, exponentially growing cultures of H-ras and N-ras knockout fibroblasts in the presence of serum demonstrated the functional specificity of those proteins in proliferating, actively cycling cells [35]. In this report, we were specifically interested in ascertaining whether N-Ras and H-Ras play also specific - or redundant - functional roles during the initial stages of the cell cycle. In particular, we wished to characterize the participation, if any, of these proteins in the process of entry into the cell cycle of G0, growth arrested cells (G0/G1 transition) and the subsequent steps of progression through early G1. For this purpose, we used commercial microarrays to characterize the profiles of genomic expression of wild-type (WT) and *ras *knockout fibroblasts (H-*ras*^-/-^, N-*ras*^-/-^, H-*ras*^-/-^/N-*ras*^-/-^) that had been subjected to serum starvation (G0) or to subsequent incubation in the presence of serum for a short, 1-hour period (G0/G1 transition) or for 8 hours (mid-G1 progression). Our data support the notion of functional specificity for H-Ras and N-Ras by documenting the occurrence of specific transcriptional profiles associated with the absence of H-Ras and/or N-Ras during defined moments of the early stages of the cell cycle.

## Results

### Analysis of serum-dependent, transcriptional profiles in wild-type and *ras *knockout fibroblasts

**Figure 1:**
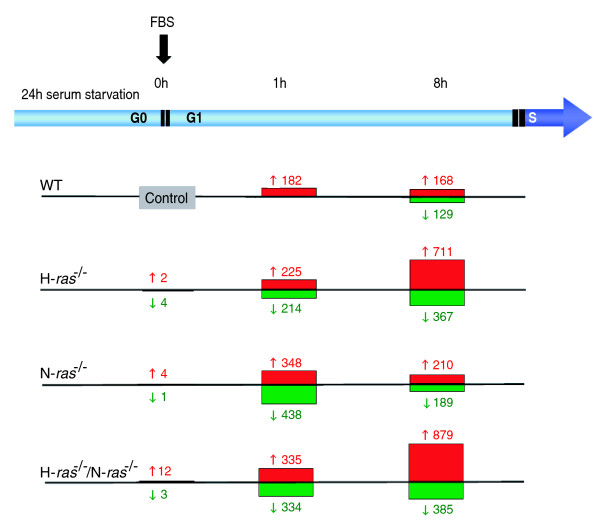
Microarray analysis of differential gene expression in wild-type and knockout fibroblasts (H-*ras*^-/-^, N-*ras*^-/- ^and H-*ras*^-/-^/N-*ras*^-/-^) subjected to serum starvation or stimulation.  Graphical representation of numbers of probesets showing differential gene expression in pair-wise SAM comparisons between the microarray hybridization data of WT fibroblasts that were serum-starved for 24 hours (Control) and corresponding microarray hybridization data of fibroblasts of the indicated WT and *ras *knockout genotypes obtained before (0 h) or after short-term (1 h) or mid-term (8 h) post-starvation incubation of the cultures in the presence of 20% fetal bovine serum (FBS). Four independent microarray hybridizations were performed for all conditions involving WT samples, and at least three independent hybridizations were performed with RNA of each of the different knockout genotypes analyzed. Numbers shown indicate the amount of induced (red) or repressed (green), differentially expressed probesets that were identified in each case using a stringent false discovery rate cut-off parameter value of 0.09.

To ascertain whether or not the different members of the Ras family control the expression of specific gene sets in response to the absence or presence of serum in cell cultures, we used commercial oligonucleotide microarrays to compare the genomic expression profile of serum-starved or serum-treated, WT, immortalized fibroblasts with those of similarly treated fibroblasts derived from knockout mice harboring single- or double-null mutations for the H-*ras *and N-*ras *loci (H-*ras*^-/-^, N-*ras*^-/-^, H-*ras*^-/-^/N-*ras*^-/-^). For this purpose, we analyzed representative RNA samples extracted from cell cultures of the mentioned WT and *ras *knockout genotypes that had been subjected to 24 hours of serum deprivation (Figure 1, 0 h), or to incubation in the presence of serum for 1 hour or 8 hours after the previous 24-hour starvation period (Figure 1, 1 h or 8 h). The results from microarray hybridizations corresponding to cell cultures subjected to serum starvation for 24 hours were instrumental to characterize the transcriptional profile of non-proliferating, off-cycle fibroblasts arrested in G0 because of the absence of growth factors caused by serum withdrawal from the cultures. Addition of serum to the starved (G0) cell cultures causes re-entry of the growth-arrested cells into the cell cycle, thus starting progression through G1 in a process involving an absolute requirement for the participation of Ras proteins [37,39,42]. In this regard, the transcriptional profiles corresponding to cell cultures incubated in the presence of serum for a short period (1 hour) are expected to include loci belonging to the population of immediate early (IE) genes known to be expressed immediately after exposure of serum-depleted fibroblasts to growth factors or serum [43-47]. On the other hand, the transcriptional profiles corresponding to cell cultures incubated in the presence of serum for 8 hours represent the transcriptomic pattern associated with the early stages of G1 progression known to lead to entry into S phase after Rb phosphorylation and subsequent E2F-dependent transcriptional activation [48].

To ensure statistical significance, four independent microarray hybridizations were carried out for each of the time points studied with WT cell samples, and three independent hybridizations were performed for each of the experimental conditions tested in the three different *ras *knockout genotypes under study (H-*ras*^-/-^, N-*ras*^-/-^, H-*ras*^-/-^/N-*ras*^-/-^). After robust normalization of the signals in all 39 separate microarray hybridizations included in this study by means of robust multi-array average software [49], the Significance Analysis of Microarrays (SAM) algorithm [50] was applied to identify the sets of differentially expressed genes showing statistically significant changes of gene expression levels when comparing the transcriptome of starved WT fibroblasts (Figure 1, Control) with that of the rest of the samples and conditions included in this study for WT and knockout cells. Figure 1 summarizes the experimental conditions and quantitative results of the microarray hybridizations performed at the different time points analyzed for each WT and *ras *knockout genotype under study, and shows the numbers of differentially expressed probesets (induced or repressed with regards to the 0 h, WT control) that were identified under the stringent selection conditions (false discovery rate (FDR) = 0.09) applied in the SAM comparisons.

### Transcriptional profiles of serum-starved fibroblasts

Initial comparison of the gene expression patterns obtained for fibroblasts of all different genotypes analyzed after 24 hours of serum starvation showed that the transcriptional profile of the control, WT fibroblasts was very similar to those of similarly treated H-*ras*^-/- ^and N-*ras*^-/- ^knockout cells, indicating that H-Ras and N-Ras exert rather minor influence over the transcriptomic profile resulting from submitting fibroblasts to the stress of serum deprivation (Figure 1). We observed that the individual H-*ras*^-/- ^and N-*ras*^-/- ^knockouts showed negligible numbers of overall transcriptomic changes and only the simultaneous absence of both N-Ras and H-Ras in the double knockout cells allowed identification of a short list of 15 differentially expressed gene probesets in comparison to the serum-starved, control WT fibroblasts at the FDR value applied (Figure 1; Table S1 in Additional data file 1). Consideration of the short list of gene probesets distinguishing the H-*ras*^-/- ^knockout cells from their corresponding WT controls suggested a predominant involvement of genes affecting cell growth and proliferation, whereas the list of genes differentially expressed in serum-starved, N-*ras*^-/- ^knockout cells indicated a higher prevalence of genes related to transcriptional processes and development or differentiation (Table S1a, b in Additional data file 1). The double knockout (H-*ras*^-/-^/N-*ras*^-/-^), starved cells allowed identification of a somewhat more extensive list of differentially expressed genes (Table S1c in Additional data file 1) that confirmed some of the functional tendencies observed in the individual *ras *knockouts. For example, *Crabp2*, a gene coding for a retinoid binding protein functionally involved in morphogenesis and organogenesis [51,52] was highly overexpressed in the single N-*ras*^-/- ^cells and was also the most highly overexpressed locus detected in the double knockout (H-*ras*^-/-^/N-*ras*^-/-^) fibroblasts (Table S1b, c in Additional data file 1).

### Serum-induced transcriptional profiles in wild-type fibroblasts

Besides analyzing the effect of serum deprivation on the cellular transcriptome, we also wished to determine the effect, if any, of eliminating H-Ras and/or N-Ras on the transcriptional profile of fibroblasts cultured in the presence of fetal bovine serum (FBS) for short periods of time (1 hour or 8 hours) post-starvation. Computational, pair-wise comparisons of the transcriptional profile of control WT, serum-starved fibroblasts with those obtained for the same cells after incubation in the presence of FBS generated two separate lists of differentially expressed genes reflecting the actual transcriptional changes caused in WT, growth arrested (G0) fibroblasts by stimulation with serum for 1 hour (Table S2 in Additional data file 1) or after 8 hours of serum incubation (Table S3 in Additional data file 1).

It is noteworthy that the transcriptomic profile depicted in Table S2 in Additional data file 1 for serum-deprived, growth arrested, WT fibroblasts treated with FBS for a short 1-hour period contained only induced genes, as no repressed loci could be identified as differentially expressed under the stringent comparison conditions used. As expected, the subset of loci showing highest transcriptional activation in Table S2 in Additional data file 1 included a series of genes (*Jun*, *Fos*, *Egr*, *Atg*, *Atf-*, *Zfp-Ier-*, and so on) belonging to the previously described category of IE genes [53-55] known to be activated in starved, G0 fibroblasts shortly after exposure to serum [43,46,47,56-58]. Interestingly, the differential expression of a large proportion of the most highly activated IE loci detected in WT fibroblasts (Table S2 in Additional data file 1) was also observed in the transcriptional profiles of H-*ras*^-/-^, N-*ras*^-/- ^and H-*ras*^-/-^/N-*ras*^-/- ^knockout fibroblasts that were similarly starved and treated with serum for 1 hour, suggesting that H-Ras and N-Ras are not participating directly in the regulation of their transcriptional activation. On the other hand, we observed that a significant number of genes listed in Table S2 in Additional data file 1 at medium-low values of transcriptional activation (as judged by R.fold or d(i) values) did not score as differentially expressed in the transcriptional profiles of corresponding *ras *knockout fibroblasts treated under similar conditions (see the column 'Differential expression not kept' in Table S2 in Additional data file 1), suggesting that in those cases H-Ras or N-Ras may be actively involved in regulation of their expression.

The list of loci showing differential expression after 8 hours of serum stimulation (Table S3 in Additional data file 1) was longer and clearly different from that of early-expressed genes after 1 hour of serum treatment. In contrast to Table S2, Table S3 in Additional data file 1 includes both induced (168 probesets; 158 genes) and repressed (129 probesets; 126 genes) loci (Figure 1), and showed very minor overlapping with the list of induced-only, IE genes included in Table S2 in Additional data file 1. Consistent with the previously described molecular mechanisms triggering G1/S transition as a consequence of Rb phosphorylation and subsequent induction of E2F-dependent transcription, this loci list includes a number of known E2F targets (*E2f3*, *Myc*, *Ctfg*, *Smad*, *Cyr61*, *Psme3*, *Tpm2*, *Vegfb*, and so on) [48,59-62]. Interestingly, some of the most highly overexpressed genes in Table S3 (see the 'R.fold' column) were functionally related to inhibition of proteolytic activities (*Serpine1 *and *Serpinb2*, *Timp1*, and so on) or to interaction with components of the extracellular matrix (*Hbegf*, *Ctgf*). Finally, as in Table S2 in Additional data file 1, a significant number of the loci differentially expressed in WT fibroblasts after 8 hours of serum stimulation did not keep such differential expression in the transcriptome of corresponding *ras *knockout fibroblast counterparts subjected to the same 8-hour serum incubation (see the column 'Differential expression not kept' in Table S3 in Additional data file 1). Interestingly, in most cases such loss of transcriptional activation or repression concerned specifically the single N-*ras*^-/- ^or the double H-*ras*^-/-^/N-*ras*^-/- ^knockout cells, an observation suggesting very different functional contributions of N-Ras and H-Ras to the regulation of gene expression during G1 progression in fibroblasts.

### Transcriptional waves induced by serum in H-*ras *and N-*ras *knockout fibroblasts

Whereas the absence of H-Ras or N-Ras caused negligible transcriptional changes relative to WT, serum-deprived fibroblasts (Figure 1, 0 h), genomic disruption of H-*ras*^-/- ^and/or N-*ras*^-/-^, individually or in combination, was associated with the occurrence of significant transcriptional changes caused by short-term incubation of the knockout fibroblasts with serum (Figure 1, 1 h and 8 h). Thus, important numbers of differentially expressed genes were detected when performing stringent pair-wise comparisons (FDR = 0.09) between the microarray hybridization pattern of serum-starved, G0 arrested WT fibroblasts and those of H-*ras*^-/-^, N-*ras*^-/- ^or H-*ras*^-/-^/N-*ras*^-/- ^fibroblasts subjected to serum starvation and subsequent stimulation with serum for 1 hour (G0/G1 transition) or 8 hours (G1 progression) (Figure 1, 1 h and 8 h).

Quantitative analysis of the microarray hybridization data showed that, among all different fibroblast genotypes tested, the N-*ras*^-/- ^fibroblasts exhibited the highest numbers of IE, differentially expressed genes after 1 hour of serum stimulation (786 altered probesets in N-*ras*^-/- ^fibroblasts versus 439 probesets in H-*ras*^-/- ^fibroblasts) (Figure 1, 1 h). In contrast, the H-*ras*^-/- ^genotype was associated with the higher number of differentially expressed loci detected during G1 progression, after 8 hours of serum stimulation (1,078 affected probesets in H-*ras*^-/- ^fibroblasts versus 399 probesets in N-*ras*^-/- ^fibroblasts; Figure 1, 8 h). These data suggest very different roles for H-Ras and N-Ras in regulation of cellular transcriptional responses to serum and reinforces the notion of specific, non-overlapping molecular functions for the different Ras isoforms. Our observation of two distinct waves of transcriptional activation (after 1 hour and 8 hours of serum stimulation) that are preferentially linked, respectively, to the N-*ras*^-/- ^or the H-*ras*^-/- ^genotype is consistent with the previously reported absolute requirement for Ras activity during at least two separate phases of the early G0 to S interval [36-41]. This raises the interesting possibility of a preferential functional involvement of N-Ras during the early phase and of H-Ras during a later phase of the period of absolute Ras activity requirement defined by means of microinjection of neutralizing Ras antibodies and dominant negative Ras forms [63-65].

**Figure 2:**
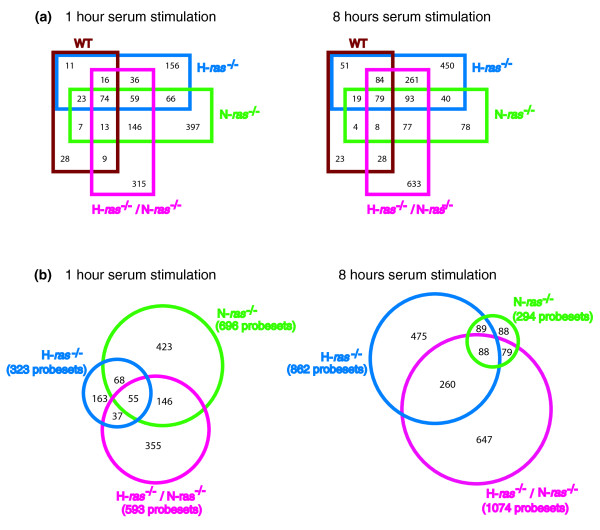
Overlapping of differential gene expression patterns from wild-type and *ras *knockout fibroblasts after serum stimulation for 1 hour or 8 hours. ** (a)** Venn diagrams showing number of probesets contained in the intersections among the different lists of differentially expressed genes occurring simultaneously in WT, H-*ras*^-/-^, N-*ras*^-/- ^or H-*ras*^-/-^/N-ras^-/-^fibroblasts after incubation of serum-starved cells in the presence of serum for 1 hour or 8 hours. **(b) **Venn diagrams showing overlapping among the lists of differentially expressed genes of H-*ras*^-/-^, N-*ras*^-/- ^or H-*ras*^-/-^/N-ras^-/- ^fibroblasts generated after excluding from them those loci showing similar values of differential expression (ratio of the R-fold values within the range 0.6 to 1.5) in the corresponding 1-hour or 8-hour WT controls.

Our initial analysis of the microarray hybridization data generated in this study focused on identifying the loci sharing differential expression among the different genotypes and experimental conditions tested (Figure 2). Figure 2a identifies and quantifies the overlapping of differentially expressed probesets occurring among all the WT, H-*ras*^-/-^, N-*ras*^-/- ^or H-*ras*^-/-^/N-ras^-/- ^genotypes analyzed, after 1 hour or 8 hours of serum treatment. On the other hand, in order to better identify the genes whose differential expression is exclusively due to the presence/absence of Ras proteins in the fibroblasts, Figure 2b shows the intersections occurring among the lists of differentially expressed genes for the H-*ras*^-/-^, N-*ras*^-/- ^or H-*ras*^-/-^/N-*ras*^-/- ^genotypes that were generated after excluding from them all the loci showing similar values of differential expression in their corresponding (1 hour or 8 hours) WT controls. Thus, Tables S4, S5 and S6 in Additional data file 1 list, respectively, the individual gene probeset composing the wave of differential expression occurring after 1 hour of serum stimulation in only the H-*ras*^-/-^, N-*ras*^-/- ^or H-*ras*^-/-^/N-*ras*^-/- ^fibroblasts but not in the WT control cells. Similarly, Tables S7, S8 and S9 in Additional data file 1 describe the wave of differentially expressed genes occurring only in H-*ras*^-/-^, N-*ras*^-/- ^or H-*ras*^-/-^/N-*ras*^-/- ^fibroblasts, respectively, but not in WT fibroblasts, after 8 h of serum incubation. To facilitate the detailed analysis of our microarray expression data, all these tables present gene lists categorized according to their degree of overexpression/repression and functional category.

### Functional signatures linked to deficiency of H-Ras or N-Ras in the transcriptional profile of serum-induced fibroblasts

**Figure 3:**
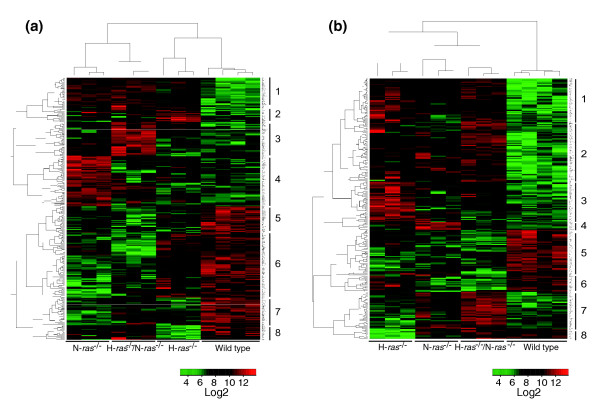
Hierarchical clustering of differentially expressed genes occurring in *ras *knockout cell lines after stimulation with serum.  **(a)** After stimulation with serum for 1 hour; **(b) **after stimulation with serum for 8 hours. Heatmaps generated by cluster analysis of absolute expression values of a selected group of gene probesets showing the highest levels of differential expression (induction or repression; stringent cutoff parameters set as FDR = 0.05 and *P*-value < 0.003) in the lists of differentially expressed genes corresponding to starved control WT fibroblasts and H-*ras*^-/-^, N-*ras*^-/-^and H-*ras*^-/-^/N-*ras*^-/- ^fibroblasts cultured after starvation in the presence of serum for 1 hour (a) (267 different probesets) or 8 hours (b) (239 different probesets). Horizontal rows represent individual gene probesets and vertical columns depict results from single microarray hybridizations. The intensity of color saturation in each probeset box (ranging from 2 to 14 in a log2 scale) provides a quantitative estimation of its expression level. Red color denotes over-expression, increasing in brightness with higher values. Green color denotes repression, increasing in brightness with lower values. Black color denotes unchanged expression signals relative to controls. Cluster blocks numbered on the right side of each heatmap identify gene sets sharing common expression behavior under the genotypes and experimental conditions indicated.

Initial qualitative analysis of the genes showing differential expression in fibroblasts after serum stimulation was provided by the global, multi-class comparisons represented by the dendrograms in Figure 3. These heatmaps were generated by means of hierarchical clustering of shortened gene lists containing the loci simultaneously showing the highest levels of induction or repression when comparing the sets of hybridization data corresponding to serum-starved, WT fibroblasts with those of the three different *ras *knockout genotypes (H-*ras*^-/-^, N-*ras*^-/- ^and H-*ras*^-/-^/N-*ras*^-/-^) tested in the presence of serum for 1 hour (Figure 3a) or 8 hours (Figure 3b).

The dendrogram analyzing the short-term wave of transcriptional response to serum stimulation for 1 hour allowed discrimination of two main vertical branches (Figure 3a). One of them encompassed the hybridization data corresponding to the N-*ras*^-/- ^and H-*ras*^-/-^/N-*ras*^-/- ^knockout cells, whereas the second one contained those of the H-*ras*^-/- ^and WT fibroblasts (Figure 3a, columns). This branching distribution indicated that the transcriptional profile of H-*ras*^-/- ^cells after 1 hour of serum induction is closest to that of WT fibroblasts, whereas the expression pattern of the H-*ras*^-/-^/N-*ras*^-/- ^cells is intermediate and more similar to that of the N-*ras*^-/- ^cells, which is located farthest away from the WT branch. This behavior is consistent with our previous suggestion (Figure 1) of a preferential contribution of N-Ras over H-Ras in generating the first transcriptional wave of immediate-early responses to serum stimulation for 1 hour. The horizontal branching of the dendrogram allowed identification of a series of gene blocks that clearly discriminated the transcriptional profiles of the different WT and *ras *knockout genotypes under study (Figure 3a, blocks 1-8).

Using GeneCodis software [66], we analyzed the functional annotations of the different loci comprising the clusters defined in Figure 3a and uncovered statistically significant associations linking specific cellular functions to the individual H-*ras*^-/- ^or N-*ras*^-/- ^genotypes (Table 1). In particular, we observed that specific subsets of genes over-expressed in N-*ras*^-/- ^fibroblasts stimulated with serum for 1 hour are linked, with a very high degree of statistical probability, to four particular functional categories, including immune responses, apoptosis, transcription and MAPK signaling (Table 1; Figure 3a, blocks 1 and 4). In addition, the clusters containing repressed genes in the N-*ras*^-/- ^columns of the same dendrogram (Figure 3a) were observed to include genes linked, with a high degree of statistical significance, to cellular functions related to cell cycle and cell adhesion and insulin signaling (Table 1a; Figure 3a, blocks 5 to 7). Similar computational analysis identified a specific subgroup of genes over-expressed in the H-*ras*^-/- ^fibroblasts stimulated with serum for 1 hour that was functionally linked to cell growth and proliferation with high statistical significance (Table 1; Figure 3a, blocks 2 and 3). In contrast, no significant functional associations were detected under similar selection conditions for the clusters containing genes down-regulated in the H-*ras*^-/- ^fibroblasts incubated with serum for 1 hour.

**Table 1:** Functional signatures of differentially expressed genes induced or suppressed in H-*ras*^-/- ^and/or N-*ras*^-/- ^fibroblasts after serum stimulation for 1 hour (G0/G1 transition)

GO ID	Functional category	Gene %	*P*-value	Relevant genotype	Significant loci
Up-regulated genes					
GO:0006955	Immunity and defense	10.5%	0.000209	N-*ras*^-/-^	*Fas*, *Cxcl10*, *Il6*, *Irf1*, *Psmb9*, *Mx1*, *Mx2*, *Cxcl2*, *Tap1*, *Ifi202b*
GO:0006915	Apoptosis	9.6%	0.000250	N-*ras*^-/-^	*Bax*, *Bid*, *Fas*, *Gadd45b*, *Perp*, *Tnfrsf11b*, *Phlda1*, *Tnfaip3*, *Trp53*
GO:0003677	Transcription	4.3%	0.000400	N-*ras*^-/-^	*Rela*, *Stat1*, *Stat5a*, *Trp53*
GO:0005515	MAPK signaling cascade	3.2%	0.000896	N-*ras*^-/-^	*Fas*, *Mapkapk2*, *Gadd45b*, *Dusp8*, *Trp53*, *Map3k8*, *Flnb*
GO:0003924	GTPase activity	5.3%	0,002511	N-*ras*^-/-^	*Ehd1*, *Mx1*, *Mx2*, *Iigp2*, *Rhoj*
GO:0008283	Cell proliferation	10.3%	0.006678	H-*ras*^-/-^	*Gnb1*, *Vegfa*, *Irs2*
Down-regulated genes					
GO:0007049	Cell cycle	16.7%	0.000109	N-*ras*^-/-^	*Ccnd2*, *Ccng2*, *Cdkn2a*, *Ppp1cc*, *Spin*, *Tsc2*, *Anapc4*, *Sash1*
GO:0005515	Cell adhesion and cytoskeleton organization	6.3%	0.000244	N-*ras*^-/-^	*Nras*, *Pik3r2*, *Ppp1cc*
GO:0004910	Insulin signaling pathway	10.4%	0,000720	N-*ras*^-/-^	*Nras*, *Pik3r2*, *Ppp1cc*, *Tsc2*, *Pck2*

Specific functional categories assigned by GeneCodis software [66] to particular subsets of the induced or repressed genes included in the dendrograms in Figure 3a. The software tool was used to search for gene annotation co-occurrences in the Gene Ontology (GO) and KEGG pathways databases, assigning values of statistical significance in each case. Functional categories are listed according to increasing *P*-value of significance for each relevant genotype. Columns provide information on functional GO ID and denomination, percentage of total number of induced or repressed genes in Figure 3a, statistical significance (*P*-value) of the functional assignment made in each case, and a representative list of differentially expressed loci associated with each functional category.

Two main vertical branches were also identified in the dendrogram containing the genes showing highest differential expression (induction or repression) after 8 hours of incubation in the presence of serum (Figure 3b). In this case, the two branches discriminated clearly the hybridization pattern of the WT fibroblasts from those of the three knockout genotypes under study (H-*ras*^-/-^, N-*ras*^-/- ^and H-*ras*^-/-^/N-*ras*^-/-^; Figure 3b, columns). Consistent with our previous suggestion of the preferential implication of H-Ras in the generation of the transcriptional wave produced in response to serum stimulation for 8 hours, the H-*ras*^-/- ^hybridization profiles clustered farthest away from the WT transcriptional profiles in this particular dendrogram (Figure 3b). Functional annotation analysis of the clusters of induced or repressed genes defined in the Figure 3b dendrogram also revealed statistically significant associations linking specific cellular functions to some of the individual *ras *knockout genotypes under study (Table 2). Thus, GeneCodis analysis of the overexpressed gene clusters occurring in H-Ras-deficient fibroblasts incubated with serum for 8 hours showed significant up-regulation of gene subsets functionally related to processes of cellular growth and proliferation, such as RNA binding/metabolism/processing and ribosomal protein biosynthesis (Table 2; Figure 3b, blocks 1 and 3). On the other hand, analysis of the population of genes over-expressed in the Figure 3b dendrogram for N-*ras*^-/- ^cells treated with serum for 8 hours allowed identification of specific subgroups that were functionally linked to cellular processes concerned with extracellular matrix interactions, cell cycle progression, DNA replication or apoptosis (Table 2; Figure 3b, blocks 4 and 7). Finally, among the population of loci repressed in N-*ras*^-/- ^cells treated with serum for 8 hours, a small gene subset was also identified that showed functional links to transcriptional processes with a high degree of statististical significance (Table 2; Figure 3b, block 6).

**Table 2:** Functional signatures of differentially expressed genes induced or suppressed in H-*ras*^-/- ^and/or N-*ras*^-/- ^fibroblasts after serum stimulation for 8 hours (G1 progression)

GO ID	Functional category	Gene %	*P*-value	Relevant genotype	Significant loci
Up-regulated genes					
GO:0003723	RNA binding	15.9%	0,000055	H-*ras*^-/-^	*Eif2s1*, *Rnu3ip2*, *Nola2*, *Cpsf4*, *Rnpc1*, *Mrpl20*, *Ddx18*, *Sf3a1*, *Hnrpll*, *Lsm8*
GO:0006412	Protein biosynthesis	11.1%	0,000405	H-*ras*^-/-^	*Iars*, *Tars*, *Eif2s1*, *Eftud2*, *Nola2*, *Rpp30*, *Mrpl20*
GO:0030529	Ribonucleoprotein complex	9.5%	0,001480	H-*ras*^-/-^	*Eftud2*, *Rnu3ip2*, *Nola2*, *Mrpl20*, *Hnrpll*, *Lsm8*
GO:0000398	mRNA splicing	6.3%	0,002982	H-*ras*^-/-^	*Rnps1*, *Eftud2*, *Sf3a1*, *Lsm8*
GO:0003743	Translation initiation factor activity	4.8%	0,007354	H-*ras*^-/-^	*Eif2s1*, *Eif4ebp1*, *AU014645*
GO:0000074	Regulation of cell cycle	4.8%	0,045790	H-*ras*^-/-^	*Ccnd2*, *Junb*, *Kras*
GO:0005578	Extracellular matrix interaction	9.8%	0,000006	N-*ras*^-/-^	*Col18a1*, *Mmp10*, *Mmp13*, *Mmp9*
GO:0005634	Cell cycle	14.6%	0,000057	N-*ras*^-/-^	*Ccne2*, *Mcm5*, *Rbl1*, *Trp53*, *Cdc6*
GO:0006260	DNA replication	12,2%	0,000035	N-*ras*^-/-^	*Mcm5*, *Pold1*, *Rrm2*, *Myst2*, *Cdc6*
GO:0006915	Apoptosis	12.2%	0,002126	N-*ras*^-/-^	*Birc5*, *Bcap29*, *Perp*, *Tnfrsf11b*, *Trp53*
Down-regulated genes					
GO:0003677	Transcription	21.4%	0,003721	N-*ras*^-/-^	*Ankrd1*, *Meis1*, *Tcf20*

Specific functional categories assigned by GeneCodis software [66] to particular subsets of the induced or repressed genes included in the dendrogram in Figure 3b. The software tool was used to search for gene annotation co-occurrences in the Gene Ontology (GO) and KEGG pathways databases, assigning values of statistical significance in each case. Functional categories are listed according to increasing *P*-value of significance for each relevant genotype. Columns provide information on functional GO ID and denomination, percentage of total number of induced or repressed genes in Figure 3b, statistical significance (*P*-value) of the functional assignment made in each case, and a representative list of differentially expressed loci associated with each functional category.

Taken together, these data reinforce the notion of non-overlapping functional roles for H-Ras and N-Ras in mammalian fibroblast cells and are consistent with our previous observations on actively growing fibroblasts [35] that pointed to preferential functional roles of H-Ras in growth and proliferation and of N-Ras in transcriptional regulation of immune/defense responses and apoptosis.

### Serum-dependent gene expression signatures linked to deficiency of H-*ras *and/or N-*ras*

**Figure 4:**
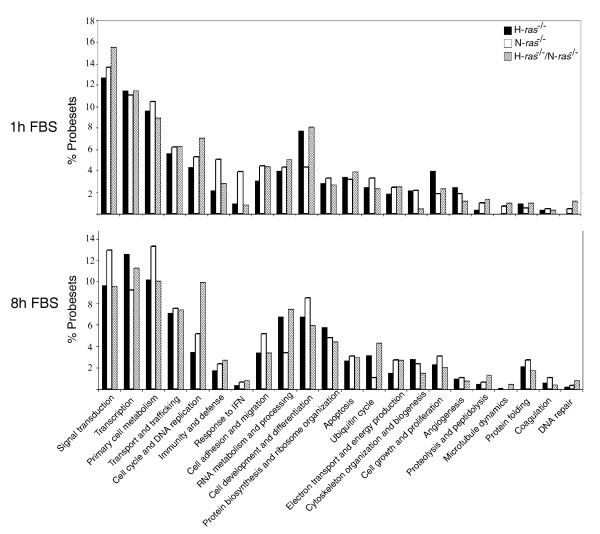
**Functional categories affected by differential gene expression in *ras *knockout fibroblasts stimulated with serum**. Bars represent percentage of total number of differentially expressed probesets (Tables S4 to S9 in Additional data file 1) corresponding to the indicated functional categories in H-*ras*^-/-^, N-*ras*^-/- ^and H-*ras*^-/-^/N-*ras*^-/- ^fibroblasts (see the legend in the figure) that were subjected to starvation and subsequent stimulation with serum (FBS) for 1 hour (upper panel) or 8 hours (lower panel). IFN, interferon.

To complement the global functional analyses derived from simultaneous, multi-class comparisons in Figure 3 and Tables 1 and 2, we also focused on identifying specific gene signatures for H-Ras or N-Ras by analyzing in detail the nature and functional annotations of the individual differentially expressed loci listed in Tables S4 to S9 in Additional data file 1 that were identified by pair-wise comparisons between the serum-starved, WT fibroblasts (0 hours) and the H-*ras*^-/-^, N-*ras*^-/- ^or H-*ras*^-/-^/N-*ras*^-/- ^fibroblasts subjected to post-starvation serum stimulation for 1 hour (G0/G1 transition; Tables S4, S5 and S6 in Additional data file 1) or 8 hours (G1 progression; Tables S7, S8 and S9 in Additional data file 1). To emphasize identification of genes whose differential expression was exclusively linked to the presence/absence of H-Ras and/or N-Ras in the fibroblasts, the lists in these tables exclude all loci showing similar values of differential expression in each of the *ras *knockout fibroblasts stimulated with serum (for 1 hour or 8 hours) and their corresponding, serum-stimulated WT controls. Functional categories such as signal transduction, transcription, primary metabolism, cell development, cell cycle, or transport and trafficking are highly represented in all cases (Figure 4). However, the identities of genes listed under each functional category are rather specific and are defined for each table, with very minor overlapping existing among the different *ras *knockout genotypes and conditions tested (Tables S4 to S9 in Additional data file 1). Here we describe some general observations concerning specific signatures detected in the different individual *ras *knockout genotypes analyzed.

The list of differentially expressed genes identified in H-*ras*^-/- ^fibroblasts stimulated with serum for 1 hour (Table S4 in Additional data file 1) includes a high percentage of loci related to signal transduction pathways (Figure 4), including Wnt-, transforming growth factor beta- and Ras-dependent signaling pathways. Among others, a notable change was a significant reduction in the expression level of the p110alpha subunit of phosphoinositide-3 kinase (PI3K; Table S4 in Additional data file 1). Furthermore, confirming the conclusions from the global analyses in Figure 3 and Tables 1 and 2, the expression profile of H-*ras*^-/- ^fibroblasts stimulated with serum for 1 hour showed specifically increased percentages of differentially expressed genes functionally related to cell development and cell growth and proliferation (Figure 4; Table S4 in Additional data file 1).

Differential gene expression during G1 progression in H-*ras*^-/- ^fibroblasts stimulated with serum for 8 hours (Table S7 in Additional data file 1) involved a high percentage of loci related to specific functional categories such as signal transduction, transcription, RNA processing, protein biosynthesis or ubiquitin interaction (Figure 4). Noticeable with regard to signal transduction was the increased expression of a number of important G protein subunits or small GTPases (including, among others, K-Ras), as well as specific regulatory proteins with GAP or GEF activity (Table S7 in Additional data file 1). In contrast to the profile of IE gene expression in H-*ras*^-/- ^cells during G0/G1 transition, the profile of H-*ras*^-/- ^cells stimulated with serum for 8 hours showed a clear increase in the number of differentially expressed loci related to functional categories such as RNA metabolism and processing, protein biosynthesis and ribosome biogenesis (Figure 4). Particularly interesting in this regard was the specific detection of significantly increased expression levels of various tRNA synthetases, translation regulatory factors and ribosomal proteins (both cytoplasmic and mitochondrial; Table S7 in Additional data file 1). Interestingly, the increased expression of tRNA acyl synthetases was conserved in similarly treated, double knockout H-*ras*^-/-^/N-*ras*^-/-^cells, but not in single knockout N-*ras*^-/- ^cells (Tables S8 and S9 in Additional data file 1). The concentration of specific transcriptional alterations on functional categories related to cellular growth and proliferation (that is, transcription, protein biosynthesis or primary cell metabolism) is consistent with our previous proposition of a predominant role of H-Ras in controlling the second wave of serum-induced transcriptional activation occurring in fibroblasts during G1 progression after 8 h of incubation in the presence of serum (Figure 1, Tables 1 and 2).

The list of differentially expressed genes specifically associated with the absence of N-Ras in fibroblasts stimulated with serum for 1 hour (Table S5 in Additional data file 1) showed a high proportion of loci functionally related to processes of cellular signal transduction, transcription and primary metabolism. Although similarly treated H-*ras*^-/- ^fibroblasts also showed predominant alteration of these functional categories (Table S4 in Additional data file 1), the identity of the genes listed under these functional headings differed significantly between the H-*ras*^-/- ^and N-*ras*^-/- ^genotypes. In particular, the elevated levels of specific transcription-related genes detected in N-*ras*^-/- ^fibroblasts incubated with serum for 1 hour (Table S5 in Additional data file 1; Figure 4) confirms the functional signature for transcription detected in the global, multi-class analyses depicted in Tables 1 and 2 and is consistent with the predominant regulatory role previously attributed to N-Ras during the first transcriptional wave of the response of fibroblasts to serum (Figure 1). The detection of significantly increased levels of genes concerned with immunity/defense and response to interferon in these N-*ras*^-/- ^fibroblasts was also striking (Table S5 in Additional data file 1; Figure 4). Interestingly, the increased expression of this functional category of genes was restricted to, and highly specific for, the N-*ras*^-/- ^genotype and was of greater quantitative significance during the early transcriptional wave of response to 1 hour of stimulation with serum (G0/G1) than during G1 progression after 8 hours of serum stimulation (Figure 4). Consistent with these observations, a preferential functional involvement of N-Ras with immunity and defense responses was also previously described in serum-supplemented, unsynchronized, actively growing cultures of N-*ras*^-/- ^cells [35]. Regarding signal transduction, Table S5 in Additional data file 1 includes significant numbers of over-expressed kinase kinases as well as repressed phosphatases, G protein subunits and Ras-related small GTPases. It was also remarkable to identify *Pik3ca *(the p110 alpha polypeptide of PI3K) and *Pik3r2 *(its regulatory p85 subunit) among the most highly repressed loci in the list (Table S5 in Additional data file 1). The simultaneous differential expression of genes related to cell migration and adhesion, together with the repression of specific members of the Rho and Rac families, may suggest functional effects over cell motility under these particular experimental conditions.

The transcriptional profile of N-*ras*^-/- ^cells stimulated with serum for 8 hours (Table S8 in Additional data file 1) showed specifically high representation of functional categories such as primary cell metabolism, signal transduction, cell development and differentiation and cell adhesion (Figure 4). In particular, the categories of primary cell metabolism and cell development and differentiation showed the highest quantitative increases in comparison to the same cells stimulated with serum for 1 hour only (Figure 4). The list of differentially expressed genes related to signal transduction is shorter for N-*ras*^-/- ^cells stimulated with serum for 8 hours (Table S8 in Additional data file 1) than in the same cells treated with serum for 1 hour (Table S5 in Additional data file 1). *Penk*, coding for proenkephalin1 [67,68], was the most highly over-expressed probeset under this functional category. Interestingly, this locus was also highly over-expressed in the same N-*ras*^-/- ^fibroblasts subjected to starvation alone (Table S1 b in Additional data file 1) or to starvation and subsequent short-term, 1-hour serum stimulation (Table S5 in Additional data file 1). Compared to its transcriptional profile during G0/G1 transition, the N-*ras*^-/- ^cells stimulated with serum for 8 hours shared similar repression of *Pi3Kr2 *and over-expression of a smaller number of different kinases. Over-expression of GAPs and repression of GEFs, as well as induction or repression of specific *ras*-related loci, was also observed in this case (Table S8 in Additional data file 1). Regarding cell development and differentiation, *Mpg *(matrix G1a protein) and *Crabp2 *(retinoic acid binding protein) showed the highest levels of over-expression under these conditions of serum stimulation. As with *Penk*, *Crabp2 *was already highly over-expressed in the same cells subjected to starvation alone (Table S1b in Additional data file 1). Finally, the group of differentially expressed genes listed under cell adhesion and migration showed great increases in the level of expression of specific matrix metallopeptidases or gap junction membrane channel proteins, suggesting specific functional effects on cell-extracellular matrix or cell-cell interactions in fibroblasts of this particular genotype (Table S8 in Additional data file 1).

Differential gene expression in double knockout H-*ras*^-/-^/N-*ras*^-/- ^fibroblasts stimulated with serum for 1 hour (Table S6 in Additional data file 1) involved a significant percentage of genes related to signaling, metabolism and transcription. There was a specific quantitative increase in the functional categories of signal transduction and cell cycle/DNA replication when compared to the other knockout genotypes analyzed (Figure 4). In these double H-*ras*^-/-^/N-*ras*^-/- ^knockout cells, the percentage of differentially expressed genes functionally assigned to signal transduction was higher during G0/G1 transition than during G1 progression (Figure 4). At both stages of the cell cycle we observed increased expression of a number of kinases, small GTPases and other G proteins as well as repression of PI3K subunits (*Pik3r2*, *Pik3ca*) (Tables S6 and S9 in Additional data file 1), a pattern consistent with that previously described in the single knockout H-*ras*^-/- ^or N-*ras*^-/- ^cells (Tables S4 and S5 in Additional data file 1)

The specific transcriptional profile of fibroblasts lacking both H-Ras and N-Ras during G1 progression (8 hours with serum; Table S9 in Additional data file 1) also showed significant involvement of signaling, transcription or cell metabolism. A specific, visible increase in the categories of cell cycle/DNA replication, RNA processing and ubiquitin cycle was also observed in this case (Figure 4).

In general, the percentage profile of functional categories associated with the absence of both H-Ras and N-Ras in fibroblasts paralleled for the most part that of the same functional categories in one or both of the individual H-*ras*^-/- ^or N-*ras*^-/-^knockout genotypes. For example, the H-*ras*^-/-^/N-*ras*^-/- ^fibroblasts behaved like H-*ras*^-/- ^cells with regard to development and differentiation or like N-*ras*^-/- ^cells with regard to growth and proliferation after 1 hour of serum stimulation. Likewise, a similar percentage distribution was detected for functional categories such as RNA metabolism or ubiquitin cycle between H-*ras*^-/-^/N-*ras*^-/- ^and H-*ras*^-/-^fibroblasts stimulated with serum for 8 hours (Figure 4). A contrasting exception to that behavior was seen with the category of cell cycle/DNA replication, which clearly showed an additive behavior in comparison to the individual H-*ras*^-/- ^and N-*ras*^-/- ^knockout cells (Figure 4).

### Functional verification of microarray-based expression data

Various alternative experimental approaches were used to validate the transcriptional data generated with microarrays. Quantitative real time PCR of a randomly selected collection of the differentially expressed genes listed in Tables S4 to S9 in Additional data file 1 was first carried out with microfluidic cards using the signal of the18S ribosomal subunit as control. Confirmation by this technique of the transcriptional trends previously detected with microarrays is indicated by the asterisks in the R.fold column of Tables S4 to S9. In general, a good qualitative agreement was observed between the microarray-derived data and the quantitative real time PCR results, although some quantitative differences were sometimes observed. Additional validation of the microarray-based transcriptional data was obtained in other cases by means of western immunoblots of cellular extracts of the same *ras *knockout fibroblast lines analyzed with microarrays after serum stimulation. This approach also confirmed the over-expression or the repression of the protein products of a series of differentially expressed genes, as indicated by the hash signs in the R.fold columns of the pertinent tables.

**Figure 5:**
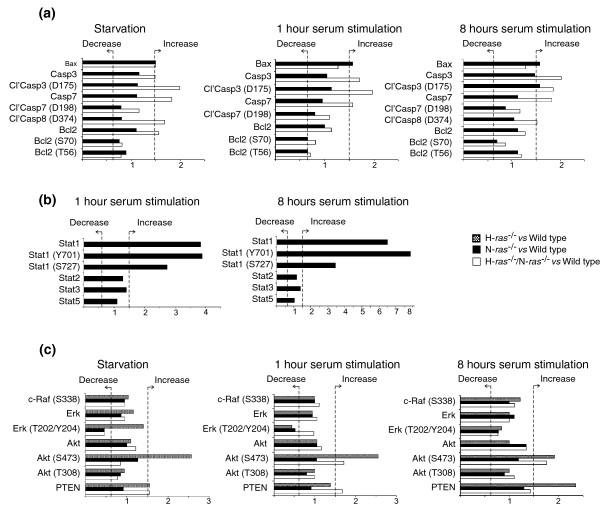
Reverse phase protein arrays. Fibroblast protein lysates of the indicated genotypes (H-*ras*^-/-^, N-*ras*^-/-^, H-*ras*^-/-^/N-*ras*^-/- ^and their WT control counterparts) were printed as indicated in Materials and methods onto slides containing two sets of spots corresponding, respectively, to WT controls and knockout samples after being subjected to the specified culture conditions (starvation or stimulation with serum for 1 hour or 8 hours). All samples were printed in duplicate, using five-point, two-fold dilution curves (starting at 2 μg/μl). The sixth point was always a negative control consisting of lysis buffer alone. After staining and development with the specific antibodies indicated on the y-axis, the slides were scanned and the ratios of the signals of the different *ras *knockout samples, normalized in relation to their respective WT controls, are depicted as bars on the x-axis of the graphs. Ratios smaller than 0.6 are considered to indicate decreased protein expression, whereas ratios higher than 1.5 are considered as indicative of increased expression. Similar results were obtained in two separate experiments. Results depicted here represent the proteomic validation of the genomic expression changes corresponding to **(a) **various apoptotic proteins, **(b) **proteins in the JAK/STAT signaling pathway, and **(c) **well known Ras effectors in fibroblasts.

Further, detailed confirmation of specific sets of the genomic transcriptional data detected with microarrays was also obtained at the protein level by means of reverse phase protein microarray analysis of appropriate cellular extracts (Figure 5). Using this approach, we documented the increased expression levels and/or activation of a number of pro-apoptotic proteins in N-*ras *and/or H-*ras*^-/-^/N-*ras*^-/- ^fibroblasts (Figure 5a), thus confirming our previous transcriptomic data (Tables 1 and 2) suggesting an increase in the apoptotic response in N-Ras deficient fibroblasts. Our microarray transcriptional data also suggested an involvement of N-Ras with immunity/defense, especially the interferon response. Validating those observations, the protein arrays demonstrated the occurrence of significantly increased levels of cellular Stat1 (signal transducer and activator of transcription 1) protein, together with an increase in its tyrosine (Y701) or serine (S727) phosphorylated forms, indicating full activation of this protein in the N-*ras*^-/- ^deleted fibroblasts [69-71]. Interestingly, no differences were detected in the expression levels of other members of the STAT family of proteins (Figure 5b). These observations in the N-*ras *and/or H-*ras*^-/-^/N-*ras*^-/- ^fibroblasts stimulated with serum for short periods (1 hour or 8 hours) are fully consistent with our previous observations in non-starved, actively growing N-Ras-deficient fibroblasts [35].

We also explored the possibility of functional links between the above described alterations of gene expression and potential defects in signal transduction. Analysis with protein microarrays of the status of a number of known components of Ras effector signaling pathways showed in N-*ras*^-/- ^knockout cells a significant decrease in extracellular signal-regulated kinase (ERK) phosphorylation (T202/Y204 residues) occurring after both starvation or short-term serum stimulation (1 hour), suggesting a specific deficiency in ERK-related signaling under those conditions (Figure 5c). Regarding the H-*ras*^-/- ^fibroblasts, our data suggested a specific deregulation in Ras-PI3K pathways as we consistently detected a significant increase of phosphorylated AKT (S473 residue) in these cells under both starvation and/or serum stimulation, as well as increased PTEN levels after stimulation with serum for 8 hours (Figure 5c).

### N-Ras regulation of Stat1 expression and activity through the Ras-ERK signaling pathway

**Figure 6:**
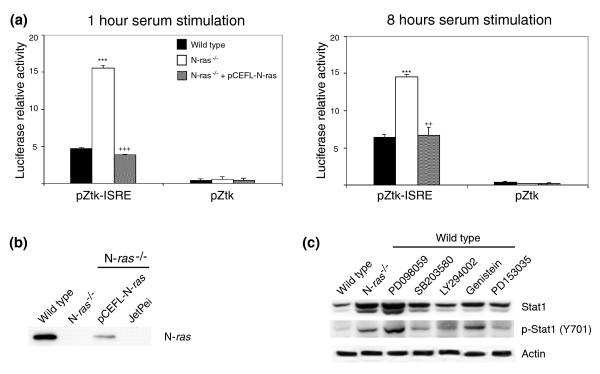
N-Ras regulation of Stat1 through the Ras-ERK pathway. **(a) **N-Ras controls transcriptional activity of ISREs in fibroblasts. Relative luciferase activity of transfected reporter ISRE constructs versus their empty vector controls was measured as described in Materials and methods after 1 hour or 8 hours of serum stimulation in cultures of WT, N-*ras*^-/- ^or N-*ras*^-/- ^cells transfected with an appropriate N-Ras construct. The assays were carried out in triplicate, with error bars indicating standard deviation (***P < 0.001 versus WT; ^+++^*P *< 0.001, ^++^*P *< 0.01 versus N-*ras*^-/- ^fibroblasts). **(b) **Restored N-Ras expression in N-*ras*^-/- ^fibroblasts. Western immunoblot showing partial recovery of N-Ras expression after transfecting N-*ras*^-/- ^fibroblasts with a vector containing a single N-*ras *copy. **(c) **Regulation of Stat1 expression and activation through the ERK pathway. WT control fibroblasts were treated with different inhibitors as indicated and total Stat1 or pStat1 (Y701) levels were detected by immunoblot. Controls of activity of the kinase inhibitors are included in Figure 9e.

We described previously that in long-term, actively growing N-*ras*^-/- ^cultures, the over-expression of Stat1 was accompanied by increased transcriptional activation of genes containing interferon-stimulated response elements (ISREs) in their promoter sequence [35]. Here we wished to determine whether those transcriptional alterations are specifically regulated by N-Ras and whether similar changes are also observable at the beginning of the cell cycle after short-term stimulation of N-Ras deficient cells with serum. Figure 6a documents our observation of significantly increased transcriptional activity mediated by ISREs in N-*ras*^-/- ^cultures stimulated with serum for 1 hour or 8 hours. Furthermore, when N-Ras expression was restored in the N-*ras *knockout cells by transfection with an appropriate construct (Figure 6b), the ISRE-dependent transcriptional activity reverted to levels similar to those found in WT control fibroblasts, confirming that N-Ras is a regulator of Stat1 activity in these cells (Figures 6a, b). To gain further insight into which specific effector pathways might be involved in regulation of Stat1 by N-Ras, we treated WT control fibroblasts with inhibitors of ERK (PD98059), p38 (SB203580), PI3K (LY294002) or epidermal growth factor receptor (PD153035) signaling, as well as a tyrosine kinase inhibitor (Genistein) and compared their resulting levels of cellular Stat1 with those of N-Ras-deficient cells (Figure 6c). We observed that down-regulation of the ERK signaling pathway produced an increase in the expression level and activation state of the Stat1 protein that was comparable to that found in N-*ras*^-/- ^fibroblasts, demonstrating that N-Ras regulates Stat1 through the ERK pathway (Figure 6c).

### Enhanced apoptosis in N-*ras*^-/- ^and H-*ras*^-/-^N-*ras*^-/- ^fibroblasts involves intrinsic and extrinsic pathway components

**Figure 7:**
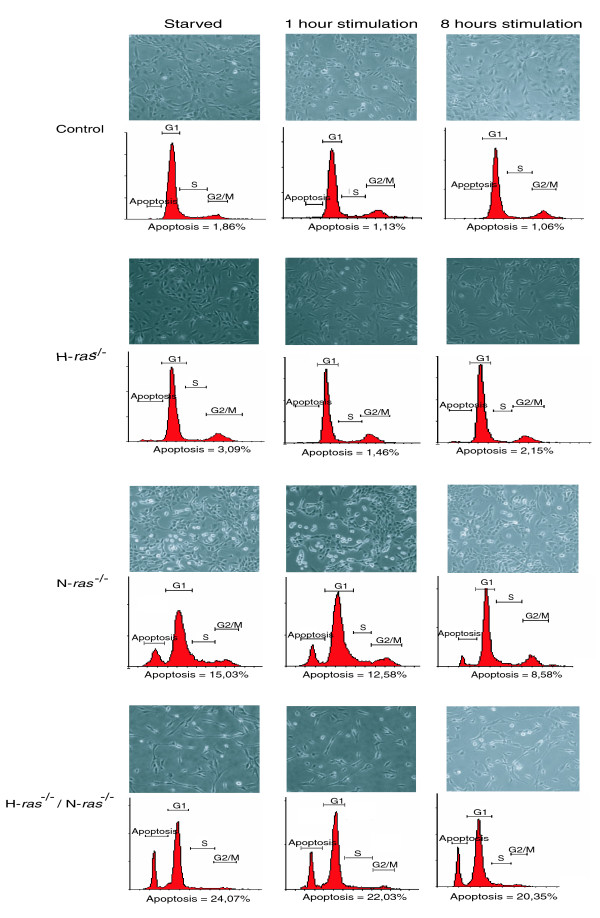
Enhanced apoptosis in N-*ras*^-/- ^and H-ras^-/-^/N-*ras*^-/- ^fibroblast cultures.  Representative phase-contrast microscopic images of WT and *ras *knockout fibroblast cultures that were serum-starved, or stimulated with serum for 1 hour or 8 hours, as indicated. Flow cytometric analyses of corresponding preconfluent cultures of the same control and knockout cell lines are also presented. Graphics are representative of three separate determinations with similar results. The averaged percentage of apoptotic cells is included in each case.

As mentioned above, our microarray-based transcriptional data and the results obtained with reverse phase protein arrays documented the increased expression and activation levels of various pro-apoptotic proteins, which suggested the possibility of increased apoptotic responses in N-*ras*^-/- ^and H-*ras*^-/-^/N-*ras*^-/- ^fibroblasts. Morphological alterations associated with apoptosis include changes in the refractive index of the cellular membrane, loss of cellular contacts, appearance of cellular blebbing and cell detachment. Accordingly, we used phase-contrast microscopy in order to detect and quantify the presence of apoptotic cells in cultures of starved and serum-stimulated fibroblasts of the various WT and *ras *knockout genotypes under study. This experimental approach demonstrated the presence of high numbers of morphologically apoptotic cells in starved and serum-stimulated N-*ras*^-/- ^cell cultures and, to a somewhat lesser extent, also in H-*ras*^-/-^/N-*ras*^-/- ^cultures (Figure 7). In contrast, consistent with the genomic and proteomic expression data, the H-*ras*^-/- ^fibroblast cultures did not display any morphological features of apoptosis and were similar to WT fibroblasts in appearance (Figure 7). These morphological observations were confirmed at the quantitative level by means of fluoresence activated cell sorting (FACS) analysis of the same fibroblast cultures, which revealed a 5 to 20% increase in the number of apoptotic cells in N-*ras*^-/- ^and H-*ras*^-/-^/N-*ras*^-/- ^fibroblasts compared to their control counterparts (Figure 7).

**Figure 8:**
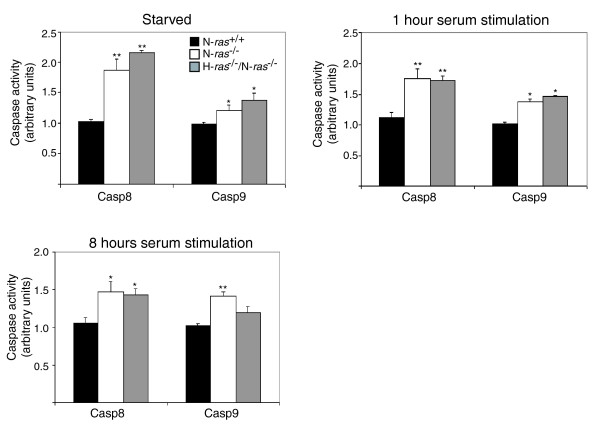
Increased caspase 8 and 9 activation in N-*ras*^-/-^and H-*ras*^-/-^/N-*ras*^-/- ^fibroblasts. Caspase 8 and 9 activities were measured as described in Materials and methods in WT, N-*ras*^-/- ^and H-*ras*^-/-^/N-*ras*^-/- ^cell lines that had been subjected to different culture conditions, including, as indicated, serum starvation for 24 hours or subsequent stimulation with serum for 1 hour or 8 hours. Three separate determinations were performed where all individual assays were carried out in triplicate. All values were normalized against their respective N-*ras *WT controls. Error bars indicate standard deviation (*P < 0.05; **P < 0.01).

Two major pathways regulate apoptosis induction in mammalian cells. In the extrinsic pathway, apoptosis is induced through specialized surface receptors such as FAS or tumor necrosis factor-α [72,73], whereas in the intrinsic pathway, this process is mainly induced through release of mitochondrial pro-apoptotic factors [72,74]. Our proteomic data showed increased expression of proteins involved in both the intrinsic (Bax, p53) and extrinsic (Casp8, FAS) pathways, together with some effector caspases and Bid, which connect both pathways. We confirmed these data and checked the functionality of both apoptotic pathways by measuring Casp8 (extrinsic pathway) and Casp9 (intrinsic pathway) activity in N-*ras*^-/- ^and H-*ras*^-/-^/N-*ras*^-/- ^fibroblasts (Figure 8). These assays showed increased activity of both caspases in the knockout cell lines compared to the WT controls and did not show predominance of either pathway in our *ras *knockout cell lines. All together, these results support our genomic and proteomic data and demonstrate an increase in the apoptotic response associated with the absence of N-Ras in N-*ras*^-/- ^and H-*ras*^-/-^/N-*ras*^-/- ^fibroblasts.

### N-Ras is a direct regulator of Bax and Perp expression

**Figure 9:**
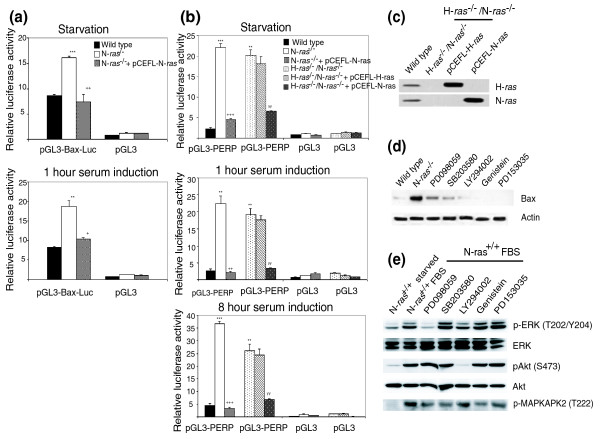
N-Ras regulation of Bax and PERP expression. **(a) **Transcriptional activation of *Bax *is N-Ras-dependent. Relative luciferase activity of transfected Bax construct versus their empty vector controls was measured in WT, N-*ras*^-/- ^and N-*ras*^-/- ^with partially restored N-*ras *expression cells as described in Materials and methods. The assays were carried out twice, each time in triplicate, with error bars indicating standard deviation (**P < 0.01; ***P < 0.001 versus control; ^+^*P *< 0.05; ^++^*P *< 0.01 versus N-*ras*^-/- ^fibroblasts). **(b) **Transcriptional activation of *Perp *is N-Ras-dependent. Relative luciferase activity was measured in transfected WT, N-*ras*^-/- ^and H-*ras*^-/-^/N-*ras*^-/- ^fibroblasts as well as in double knockout fibroblasts with partially restored expression of either H-*ras *or N-*ras*. The assays were done twice, each time in triplicate, with error bars indicating standard deviation (*P < 0.05; **P < 0.01; ***P < 0.001 versus WT cell lines; ^+^*P *< 0.05; ^++^*P *< 0.01 versus N-*ras*^-/- ^fibroblasts; Ψ *P *< 0.05; ΨΨ *P *< 0.01 versus H-*ras*^-/-^/N-*ras*^-/- ^fibroblasts). **(c) **Western immunoblot showing recovery of H-Ras and N-Ras expression after transfection of double knockout H-*ras*^-/-^/N-*ras*^-/- ^fibroblasts with vectors containing a copy of either H-*ras *or N-*ras*. **(d) **Regulation of *Bax *expression and activation through the ERK and p38 pathways. Control fibroblasts were treated with different Ras-effector inhibitors as indicated in Materials and methods, and total Bax levels were detected by immunoblot. **(e) **Controls of activity of the chemical inhibitors on their corresponding molecular targets.

Our microarray hybridization data consistently detected the over-expression of the apoptotic *Bax *and *Perp *loci in N-*ras*^-/- ^and/or H-*ras*^-/-^/N-*ras*^-/- ^fibroblast cultures (Tables S5 to S9 in Additional data file 1). To gain further insight into the functional significance of these observations, we carried out luciferase assays to quantify the transcriptional activation of the *Bax *and *Perp *promoters in the N-*ras*^-/- ^and H-*ras*^-/-^/N-*ras*^-/- ^fibroblasts compared to their WT controls (Figure 9). Our assays using specific reporter constructs demonstrated in both cases the transcriptional activation of these promoters in the absence of N-Ras expression in single or double knockout cells (Figures 9a, b). In order to confirm the specific implication of N-Ras in regulating the transcriptional activation of both genes, we transfected the knockout cells with vectors containing either H-*ras *or N-*ras*, thus recovering expression of these genes in the corresponding null cell lines (Figures 9a-c). When N-*ras *expression was restored in either single or double knockout cell lines, the activity of the *Bax *and *Perp *promoters decreased to values similar to those found in WT control fibroblasts. In contrast, when H-*ras *expression was recovered in the double knockout fibroblasts we did not observe any change in the activity of the *Perp *promoter, implying that deregulation of this gene in H-*ras*^-/-^/N-*ras*^-/- ^fibroblasts was due to the absence of N-Ras, but not of H-Ras (Figure 9b, c). Finally, further information concerning possible effector pathways involved in transcriptional regulation of *Bax *by N-Ras was obtained by using a battery of specific inhibitors on control WT fibroblasts and quantifying the resulting levels of Bax protein expression (Figure 9d). We observed increased expression levels of Bax protein after 24 hours incubation in the presence of specific inhibitors of ERK or p38 signaling (Figure 9d), suggesting the possible participation of these two pathways in the regulatory effect of N-Ras on Bax protein levels. Interestingly, no significant changes in the transcriptional activities of the Bax and Perp reporters were observed when the luciferase assays were performed in the presence of ERK or p38 inhibitors (not shown), suggesting that the enhancing effect of those inhibitors on Bax protein expression levels detected by WB (Figure 9d) may involve additional post-transcriptional regulatory mechanisms. Overall, our data support the notion of a specific, direct involvement of N-Ras through transcriptional and post-transcriptional regulatory mechanisms in the control of apoptotic responses in fibroblasts.

## Discussion

Various experimental approaches, including studies of over-expression, subcellular location/processing, genomic disruption and genomic/proteomic profiling support the notion that the mammalian H-Ras, N-Ras and K-Ras isoforms play non-overlapping, differentiated functional roles [1,3,6]. For example, our recent characterization of the transcriptomic profile of actively growing fibroblasts lacking H-Ras and/or N-Ras provided significant evidence for the functional involvement of N-Ras in cellular responses related to immunomodulation/host defense and apoptosis [35]. Other reports indicate also that the mammalian Ras proteins play essential functional roles in regulation of the cell cycle [1,3,5,36]. This is based on the observation that microinjection of non-specific, neutralizing Ras antibodies has demonstrated an absolute requirement for Ras activity at several points during serum stimulation of quiescent cells [36-41]. However, little is known about the exact mechanisms mediating the participation of Ras proteins in cell cycle progression or about the possibility that different Ras isoforms play differential functional contributions in this process.

The present study, focused on the joint analysis of the genomic expression profiles of WT and *ras *knockout (H-*ras*^-/-^, N-*ras*^-/-^, H-*ras*^-/-^/N-*ras*^-/-^) fibroblasts subjected to serum starvation or to subsequent stimulation with serum for short periods of time, provides a valid experimental system to test whether N-Ras and H-Ras play specific -or redundant - functional roles during the initial stages of the cell cycle, and to analyze potential mechanisms involved. Thus, microarray-based analysis of the transcriptomic profiles of the serum starved, G0-arrested fibroblasts enables the participation of the Ras isoforms in cellular responses to the stress of serum deprivation to be gauged. On the other hand, the study of the transcriptomic profiles of the same set of serum-arrested fibroblast lines after stimulation with serum for 1 hour or 8 hours was instrumental to discern different functional contributions of N-Ras or H-Ras during G0/G1 transition (1 hour) or mid-G1 progression (8 hours).

The meaningful, joint analysis of the complete set of different transcriptional profiles generated in this study involved in most instances the comparison of the profiles of G0-arrested WT cells with those of the other samples and conditions studied here by means of microarray hybridization. Interestingly, the comparison of the gene expression patterns of G0-arrested fibroblasts (after 24 hours of serum starvation) of all different genotypes tested showed negligible differences among the transcriptional profiles of the WT controls and those of the H-*ras*^-/- ^or N-*ras*^-/- ^knockout cells (Tables 1 and 2), indicating that H-Ras and N-Ras do not play a highly significant functional role in generating the transcriptional response of cultured fibroblasts to the stress of serum deprivation.

The hybridization data generated here also allowed us to ascertain whether H-Ras and N-Ras had any specific effect on the transcriptional responses of the starved fibroblasts to serum stimulation. In particular, the microarray hybridizations corresponding to fibroblasts incubated with serum for 1 hour were aimed at targeting the specific gene population transcribed immediately after exit of G0 and re-entry into G1 of the cell cycle (G0/G1 transition) [43,46,47,56-58], whereas those corresponding to cells stimulated with serum for 8 hours were geared to characterize the profile of induced/repressed genes occurring in fibroblasts progressing through the early-mid stages of G1 phase in the cell cycle [48,59-62]. Accordingly, the list of differentially expressed genes resulting from comparing the profile of G0-arrested WT cells with that of the same WT cells after short-term stimulation (1 hour) with serum contained only induced genes that corresponded, for the most part, with the expected population of so-called IE genes (*jun*, *fos*, and so on) known to be transcribed in starved G0 fibroblasts shortly after exposure to serum in culture [43,46,47,56-58]. Interestingly, the profiles of H-*ras*^-/-^, N-*ras*^-/- ^and H-*ras*^-/-^/N-*ras*^-/- ^knockout fibroblasts shared high differential expression of many of the IE loci detected in WT cells, suggesting that, in those cases, H-Ras and N-Ras do not have a direct functional contribution to the transcriptional activation of IE loci and that the regulation of these early serum responses is probably mediated through other Ras-independent signaling pathways. On the other hand, a significant number of differentially expressed, primary response genes were also identified in the WT cells that did not score as differentially expressed in the transcriptional profiles of corresponding *ras *knockout fibroblasts treated under similar conditions, suggesting that in those cases H-Ras or N-Ras may be actively involved in regulation of their expression. The transcriptional profile of WT fibroblasts stimulated with serum for 8 hours was clearly different from that detected during G0/G1 transition (1 hour) and includes a long list of induced and repressed genes encompassing E2F targets that would be expected as a consequence of the process of G1 to S progression, after Rb phosphorylation and subsequent E2F transcriptional activation [48,59-62]. Interestingly, the transcriptional activation of many differentially expressed loci detected in the WT cells was lost in the *ras *knockout fibroblasts subjected to the same treatment with serum. Such loss of transcriptional activation was particularly noticeable in the case of the N-*ras*^-/- ^and H-*ras*^-/-^/N-*ras*^-/- ^knockout cells, suggesting a major functional participation of Ras proteins, particularly N-Ras, in the regulation of transcriptional programs during early G1 progression.

Whereas the absence of H-Ras or N-Ras did not seem to modify the cellular responses to serum deprivation stress, the genomic disruption of H-*ras*^-/- ^and/or N-*ras*^-/-^, individually or in combination, led to very different transcriptional responses to serum stimulation in comparison to the G0-arrested, WT fibroblasts. Our data clearly show that the absence of N-Ras causes the highest quantitative changes in the first wave of transcriptional activation occurring during G0/G1 transition (1-hour serum stimulation), whereas the absence of H-Ras was associated with the largest size of the second wave of transcriptional activation corresponding to mid-G1 progression (8-hour serum stimulation). The preferential association of N-Ras and H-Ras with each of these two distinct transcriptional waves is consistent with previous reports documenting the absolute requirement for Ras activity during different moments of the early G0 to S interval [36-41], and raises the interesting possibility of a preferential functional involvement of N-Ras with the immediate-early cellular responses to serum stimulation and of H-Ras with the cellular responses related to growth and proliferation during mid-G1 progression.

The analysis of functional annotations corresponding to the differentially expressed genes identified in the multi-class comparisons depicted in the Figure 3 dendrograms and the pair-wise comparisons described in Tables S4 to S9 in Additional data file 1 was instrumental for the assignment of specific functional signatures to H-Ras and N-Ras during the two specific stages of the early cell cycle (G0/G1 transition and mid-G1) that were studied here. Thus, consistent with our previous conclusion attributing a preferential functional role to N-Ras in control of the early (G0/G1 transition) transcriptional wave, and to H-Ras in control of the second (mid-G1) transcriptional wave, the branching of the respective dendrograms clearly shows that the transcriptional pattern of N-*ras*^-/- ^cells was the most distant from that of the WT control during the early G0/G1 transition and, in contrast, that of H-*ras*^-/- ^fibroblasts clustered farthest away from its WT control in the set of samples corresponding to stimulation with serum for 8 hours, during mid-G1 progression. Computational evaluation (Genecodis) of the functional annotations for the components of the clusters in the dendrograms provided statistically significant evidence linking the absence of N-Ras during G0/G1 transition to induction of loci related to four main categories of cellular functions, including immune defense responses, apoptosis, transcription and MAPK signaling, and to repression of loci functionally related to cell cycle control, cell adhesion and insulin signaling. The same computational analyses also demonstrated the occurrence of a statistically significant link between the absence of H-Ras and induction of genes related to RNA binding/metabolism/processing and ribosomal protein biosynthesis during the second transcriptional wave analyzed in this study (8-hour serum stimulation; mid-G1 progression). These observations during early stages of the cell cycle are clearly consistent with previous observations from our laboratory with actively growing fibroblasts [35] that pointed to preferential functional roles of H-Ras in growth and proliferation and of N-Ras in transcriptional regulation of apoptosis and immune/defense responses. Our conclusions are further supported by recent reports [75-77] on the contribution of Stat proteins and interferon signaling to oncogenic transformation and human tumor development. All these observations thus reinforce the notion of non-overlapping functional roles for H-Ras and N-Ras in mammalian fibroblast cells.

The global functional analyses were further complemented and reinforced by the study of the functional annotations of the individual genes listed in the pair-wise comparisons summarized in Tables S4 to S9 in Additional data file 1. The identification of individual genes whose transcription was most specifically linked to the absence of either H-Ras or N-Ras was facilitated by excluding from consideration all loci showing similar levels of differential expression (d-value or R.fold parameters in pair-wise comparisons to G0-arrested, WT cells) for both the WT and the *ras *knockout (H-*ras*^-/-^, N-*ras*^-/- ^or H-*ras*^-/-^/N-*ras*^-/-^) cells subjected to stimulation with serum for the same time (1 hour or 8 hours). Confirming the previous global analysis, the list of differentially expressed genes in H-*ras*^-/- ^fibroblasts subjected to serum stimulation included many different loci that were functionally related to development, growth and proliferation. Particularly striking in this regard was the elevated number of genes coding for tRNA synthetases and ribosomal proteins in both the single H-*ras*^-/- ^and double H-*ras*^-/-^/N-*ras*^-/- ^knockout cells, but not in N-*ras*^-/- ^cells, suggesting a specific, direct link between H-Ras and these types of cellular functions related to growth processes. The transcriptional profile of N-Ras-deficient cells displayed many individual genes falling under the functional categories of defense and apoptosis (as previously noted), as well as cell adhesion, motility and signal transduction processes. Regarding this latter category, it was remarkable to observe in serum-stimulated N-*ras*^-/- ^cells a significant reduction in expression level of components of PI3K signaling pathways, in particular the p85 and p110 subunits of this enzyme, suggesting a significant contribution of N-Ras to cellular signaling through this pathway. All in all, these observations are consistent with the suggestion of a significant functional contribution of N-Ras to the first wave of transcriptional activation associated with G0/G1 re-entry into the cell cycle. Finally, the profile of functional categories affected in the double H-*ras*^-/-^/N-*ras*^-/- ^knockouts reflected, in general, the individual profiles exhibited by the individual H-*ras*^-/-^or N-*ras*^-/- ^genotypes, with a notable exception in the category of cell cycle/DNA replication, where the behavior of the double knockout fibroblasts was additive in relation to the individual knockout genotypes, suggesting that H-Ras and N-Ras complement each other functionally with regards to cellular functions affecting cell cycle progression. In any event, the validation of any proposed functional link resulting from the analysis of transcriptional profiles requires further direct confirmation by means of specific, *in vivo *functional assays.

Various experimental approaches, including reverse phase protein arrays and direct functional assays of knockout fibroblasts of the specific genotypes under study provided direct support for some of the functional roles attributed to N-Ras or H-Ras on the basis of the transcriptional profiles of pertinent knockout cells, and also offered specific hints on the possible mechanisms involved. For example, with regards to cellular defense processes, our results demonstrated the specific increase of Stat1 expression and phosphorylation in N-Ras-deficient cells and provided direct evidence for the participation of Ras-ERK signaling pathways to mediate the transcriptional regulation of Stat1 by N-Ras. Our data also documented the enhanced apoptotic responses associated with the absence of N-Ras in fibroblasts and provided evidence for the participation of both intrinsic and extrinsic pathways in a process involving direct transcriptional and post-transcriptional regulation by N-Ras of major components, such as Bax and Perp, through ERK- and p38-mediated pathways.

## Conclusions

We have shown that the transcriptional profiles of G0-arrested, serum-starved WT and *ras *knockout fibroblasts (H-*ras*^-/-^, N-*ras*^-/-^, H-*ras*^-/-^/N-*ras*^-/-^) are very similar, indicating that these Ras proteins do not play highly important roles in regulation of transcriptional responses to the stress of serum deprivation. In sharp contrast, the transcriptional profiles of knockout fibroblasts lacking H-Ras and/or N-Ras are very different from those of their WT controls after serum stimulation for 1 hour (G0/G1 transition) or 8 hours (mid G1 progression), indicating that H-Ras and N-Ras exert distinct, specific cellular functions during the initial stages of the cell cycle. Whereas all three different *ras *knockout strains exhibited important transcriptional alterations during both stages of the cell cycle, the absence of N-Ras was quantitatively more disruptive for the first transcriptional wave linked to G0/G1 transition, and the absence of H-Ras affected more potently the transcriptional wave linked to G1 progression. Furthermore, the transcriptional changes of H-Ras-deficient cells showed preferential involvement of loci functionally related to growth and proliferation whereas those of N-Ras-deficient cells were more frequently concerned with development, cell cycle regulation, immunomodulation and apoptosis. Functional analysis indicates that N-Ras contributions to cellular immunity/defense responses is mediated, at least in part, through ERK-dependent regulation of Stat1 expression and activity, whereas its participation in apoptotic responses involves transcriptional regulation of various genes (*Bax *and *Perp*) via ERK and p38 signaling pathways.

Our data documenting the occurrence of specific transcriptional profiles associated with the absence of H-Ras and/or N-Ras during early cell cycle stages are consistent with previous reports showing absolute requirements for different peaks of Ras activity during the initial stages of the cell cycle and confirm the notion of functional specificity for the H-Ras and N-Ras isoform proteins.

## Materials and methods

### Cell culture

Cell lines from the appropriate *ras *genotype were harvested on Dulbecco's modified Eagle's medium (DMEM; Gibco Paisley, UK) supplemented with FBS (10%; Hyclone, Logan, Utah, USA), glutamine (2 mM), penicillin (100 U/ml) and streptomycin (100 mg/ml). Cultures were grown in a humidified CO_2 _(5%) atmosphere at 37°C and when subconfluent cells were starved for 24 hours. After starvation cells were either used for RNA/protein isolation, or induced for 1 hour or 8 hours with 20% FBS and then RNA/protein isolation was carried out.

When using the pharmacological inhibitors PD098059 (37 μM), SB203580 (10 μM), LY294002 (20 μM), Genistein (100 μM), and PD153035 (10 μM), WT fibroblasts were cultured as usual and when 70 to 80% confluence was reached they were treated for 24 to 48 hours in the presence of the inhibitor and then collected for protein extraction. All the inhibitors were purchased from Calbiochem^® ^(Darmstadt, Germany).

### RNA isolation, cDNA synthesis and microarray hybridization

For each cell line and time point under study RNA was purified from two 10-cm culture dishes per cell line using a commercial kit (RNeasy, Qiagen, Hilden, Germany). Concentration was measured at 260 nm (Ultrospec 2000, Pharmacia Biotech Buckinghamshire, UK) and purity and quality was determined using RNA 6000 Nanochips (Agilent Technologies, Santa Clara, CA, USA). RNA was then used to synthesize cRNA probes for hybridization to Affymetrix MGU74Av2 GeneChip high-density oligonucleotide microarrays. Microarray hybridization was carried out as described in the *Gene Expression Analysis Technical Manual *provided by Affymetrix [78].

### Microarray hybridization data analysis: normalization, differential gene expression and clustering

Pre-confluent cultures of at least two separate cell lines belonging to each of the *ras-*related genotype(s) under study (WT, H-*ras*^-/- ^and N-*ras*^-/- ^and H-*ras*^-/-^/N-*ras*^-/-^) were harvested and their RNA extracted for subsequent analysis using Affymetrix high density oligonucleotide microarrays MGU74Av2. At least three independent microarray hybridizations were performed with RNA corresponding to each of the null mutant *ras *genotypes in the experimental conditions under study. Thus, this study encompassed a total of 3 different data sets (starved cells, cells stimulated with serum for 1 hour and cells stimulated with serum for 8 hours), each consisting of 13 separate chip microarray hybridizations (4 for controls and 3 for each of the three null mutant genotypes). All array hybridization data are available at the NCBI, Gene Expression Omnibus database [GEO:GSE14829] [79].

Data analysis was carried out using the robust multi-array average and SAM algorithms as previously described [35]. Changes in probeset expression level in knockout cell lines compared to their WT counterparts were identified as significant using a FDR cutoff value of 0.09. Following identification of the differentially expressed probesets, the corresponding matrix of expression values for all microarray hybridizations performed were analyzed using the *hclust *clustering algorithm implemented in R [80]. This algorithm performs hierarchical cluster analysis with complete linkage to find similarity between probesets based on their expression values in the different chip microarrays analyzed. The algorithm classifies the probesets in correlated groups presenting similar expression profiles or expression signatures. The statistical significance of functional Gene Ontology annotations was estimated by means of *P*-values of confidence calculated by running Fisher's exact test to compare the number of genes assigned to the various functional categories within each cluster of the dendrogram.

### Functional analysis

Functional analysis of the significant genes obtained for each induced state was done using a functional annotation tool called GeneCodis (Gene Annotation Co-occurrence Discovery) [66,81]. This tool finds combinations of co-occurrent annotations that are significantly associated with a list of genes under study with respect to a reference list. The significance of the annotations is calculated using a hypergeometric statistical test with FDR *P*-value correction and using as reference the mouse genome. The annotations were done at the same time to the full Gene Ontology) database [82] and to the Kyoto Encyclopedia of Genes and Genomes (KEGG) pathways database [83]. After the analyses were done with GeneCodis, the redundancy on the list of genes that are assigned to each functional class was depurated by manual curation in order to identify distinct groups of genes that include similar or related biological functions and that can be enclosed in more general cellular processes as presented in Tables 1 and 2.

### Microfluidic cards

RNA from mouse embryo fibroblasts subjected to the different experimental conditions under study was used for quantitative PCR validation on low density microarrays, microfluidic cards (Applied Biosystems, Foster City, CA, USA) using the 18 s ribosomal subunit as an internal control. RNA (10 μg; 1 μl final reaction volume) were reverse transcribed using the High Capacity cDNA Archive Kit (Applied Biosystems) as recommended by the supplier. The previously synthesized cDNA (5 μl) was then mixed with 50 μl of the Taqman^® ^Universal PCR Master Mix (Applied Biosystems) and 50 μl of RNAses free water. Samples were loaded into the microfluidic cards containing the lyophilized oligos in each well and then centrifuged at 1,200 rpm for 2 minutes. Cards were sealed using a Low Density Array Sealer (Applied Biosystems) and the PCR reaction was carried out in an ABI PRISM^® ^7900HT termocycler (Applied Biosystems). Results were analyzed using the software Sequence Detection Systems (SDS) v2.1 (Applied Biosystems).

### Western blot analysis of cellular extracts

Protein lysates were obtained and quantified as previously described [35] Lysates (30 to 40 μg/lane) were loaded onto SDS polyacrylamide gels and the electrophoresed proteins transferred to polyvinylidene difluoride membranes (Millipore Immobilon-P, Billerica, MA, USA) by electroblotting. Membranes blocked in Tween 20-tris-buffered saline (10 mM Tris-HCl (pH 8.0), 150 mM NaCl, 0.05% Tween 20) plus 1% bovine serum albumin were incubated, as appropriate, with dilutions of 0.2 mg/ml of commercial antibodies from Santa Cruz Biotechnologies (Santa Cruz, CA, USA) and horseradish peroxidase-conjugated (Amersham Bioscience, Buckinghamshire, UK) were used as secondary antibodies. Immunoblots were developed using the commercial Enhanced Chemiluminescence (ECL) and ECL plus kits (Amershan Pharmacia Biotech, Piscataway, NJ, USA) following the supplier's recommendations.

### Reverse-phase protein lysate array layout and antibody staining

Reverse phase protein microarrays were done as previously described [35]. Origin and dilution of the antibodies used is shown in Table S10 in Additional data file 1. Development of antibody-stained arrays and quantification of the signal data obtained after scanning the arrays were carried out as described [84,85].

### Luciferase reporter assays

Transcriptional activity of control, N-*ras*^-/- ^and the double H-*ras*^-/-^/N-*ras*^-/- ^cells was assayed using luciferase reporter constructs 8 ISRE-tkLuc (kindly provided by Dr R Pine, The Public Health Research Institute, Newark, NJ, USA)), Bax-pGL3 and PERP-pGL3 (kindly provided by Dr P Lazo, Centro de Investigacion del Cancer, Salamanca, Spain). Cells seeded in six-well plates (5 × 10^5 ^cells/well) and cultured for 12 hours were transfected with reporter plasmids (5.0 μg) using JetPEI (Polyplus transfection, Illkirch, France). phRL-tk plasmid (50 ng; Promega, Madison, WI, USA) was co-transfected as an internal control. After further culture for 24 to 36 hours in DMEM with 10% FBS serum, cell extracts were assayed for luciferase activity. Where indicated, cotransfections were done by adding 5.0 μg of a construct containing N-*ras *(N-*ras*-pCEFL) or H-*ras *(H-*ras*-pCEFL) genes. Luciferase assays were performed using a dual luciferase reporter kit (Promega). Luminescence was determined with a MiniLumat LB9506 luminometer (Berthold, Bad Wildbad, Germany).

### Caspase 8 and caspase 9 activity assays

We seeded 5 × 10^5 ^cells in six-well plates and once attached they were starved for 24 hours and/or serum stimulated for 1 hour or 8 hours as previously described. After washing twice with cold phosphate-buffered saline cells were lysated with Reporter lysis buffer 1× (Promega), centrifuged for 5 minutes at 12,000 rpm and 4°C and supernatant collected into a new tube. Caspase 8 and 9 activity was measured by adding to the lysates the corresponding reagent (Caspase-Glo^® ^8 or Caspase-Glo^® ^9, Promega) in a 1:1 ratio. After 1 hour incubation at room temperature caspase 8 and caspase 9 activity was determined using a MiniLumat LB506 luminometer (Berthold).

## Abbreviations

DMEM: Dulbecco's modified Eagle's medium; ERK: extracellular signal-regulated kinase; FBS: fetal bovine serum; FDR: false discovery rate; GAP: GTPase activating protein; GEF: guanosine nucleotide exchange factor; IE: immediate early; ISRE: interferon-stimulated response element; KO: knockout; SAM: Significance Analysis of Microarrays; Stat: signal transducer and activator of transcription; WT: wild type.

## Authors' contributions

EC carried out the experiments and data gathering and wrote the initial version of the manuscript. CG participated in data collection and manuscript editing. AN performed tasks related to maintenance and use of the animal knockout colony. JdlR carried out the bioinformatics analyses of the transcriptional data. ES designed and coordinated the study and wrote the manuscript. All authors read and approved the final manuscript.

## Additional data files

The following additional data are available with the online version of this paper: Tables S1 to S10 listing the differential expression detected in WT and knockout fibroblasts of the indicated genotypes that were cultured under conditions of serum starvation or stimulation, as specified in each case (Additional data file 1).

## Supplementary Material

Additional data file 1 Table S1: differential gene expression in *ras *knockout fibroblasts after serum starvation. Table S2: differential gene expression in serum starved, G0-arrested WT fibroblasts after incubation of cell cultures in the presence of serum for 1 hour. Table S3: differential gene expression in serum-starved, G0-arrested WT fibroblasts after stimulation with serum for 8 hours. Table S4: differential gene expression in serum-starved, G0-arrested H-*ras*^-/- ^fibroblast cultures after stimulation with serum for 1 hour. Table S5: differential gene expression in serum-starved, G0-arrested N-*ras*^-/- ^fibroblasts after incubation of cell cultures in the presence of serum for 1 hour. Table S6: differential gene expression in serum-starved, G0-arrested H-*ras*^-/-^/N-*ras*^-/- ^fibroblasts after incubation of cell cultures in the presence of serum for 1 hour. Table S7: differential gene expression in serum-starved, G0-arrested H-*ras*^-/- ^fibroblasts after incubation of cell cultures in the presence of serum for 8 hours. Table S8: differential gene expression in serum-starved, G0-arrested N-*ras*^-/- ^fibroblasts after incubation of cell cultures in the presence of serum for 8 hours. Table S9: differential gene expression in serum-starved, G0-arrested H-*ras*^-/-^/N-*ras*^-/- ^fibroblasts after incubation of cell cultures in the presence of serum for 8 hours. Table S10: antibodies used in reverse phase protein microarrays. Click here for file
