# Supplementary material for: Serum-dependent transcriptional networks identify distinct functional roles for H-Ras and N-Ras during initial stages of the cell cycle
Source: Genome Biol. 2009 Nov 6;10(11):R123. doi: 10.1186/gb-2009-10-11-r123 (PMC3091317; doi:10.1186/gb-2009-10-11-r123)
Supplement: Additional data file 1 — Table S1: differential gene expression in ras knockout fibroblasts after serum starvation. Table S2: differential gene expression in serum starved, G0-arrested WT fibroblasts after incubation of cell cultures in the presence of serum for 1 hour. Table S3: differential gene expression in serum-starved, G0-arrested WT fibroblasts after stimulation with serum for 8 hours. Table S4: differential gene expression in serum-starved, G0-arrested H-ras-/- fibroblast cultures after stimulation with serum for 1 hour. Table S5: differential gene expression in serum-starved, G0-arrested N-ras-/- fibroblasts after incubation of cell cultures in the presence of serum for 1 hour. Table S6: differential gene expression in serum-starved, G0-arrested H-ras-/-/N-ras-/- fibroblasts after incubation of cell cultures in the presence of serum for 1 hour. Table S7: differential gene expression in serum-starved, G0-arrested H-ras-/- fibroblasts after incubation of cell cultures in the presence of serum for 8 hours. Table S8: differential gene expression in serum-starved, G0-arrested N-ras-/- fibroblasts after incubation of cell cultures in the presence of serum for 8 hours. Table S9: differential gene expression in serum-starved, G0-arrested H-ras-/-/N-ras-/- fibroblasts after incubation of cell cultures in the presence of serum for 8 hours. Table S10: antibodies used in reverse phase protein microarrays. [file gb-2009-10-11-r123-S1.DOC]

**Table S1. Differential gene expression in *ras* knockout fibroblasts after serum starvation.** Differentially expressed genes identified in SAM contrasts (FDR=0,09) comparing 24h serum-starved, WT fibroblasts to similarly treated fibroblast cultures of the indicated *ras* knockout genotypes (a. H-*ras*^-/-^; b. N-*ras*^-/-^; c. H-*ras*^-/-^/N-*ras*^-/-^). Affymetrix probeset ID, genetic locus and a descriptive gene name are indicated in each case. The lists are organized according to degree of overexpression or repression (quantitated by R.fold and d(i) values). d(i) is a parameter measuring the statistical distance separating the calculated expression value of each gene probeset from the null hypothesis (no-change). p-value is an statistical measure indicating the probability of random expression for that probeset. R fold is the log2 value of the fold change measuring the overexpression or repression of the probesets in the collection of microarrays. The * and # symbols in the R.fold column denote independent validation of the transcriptional data obtained by means of quantitative RT-PCR or Western immunoblot, respectively.

| **Probeset ID** | **Gene name** | **Description** | **d(i)** | **p.value** | **R.fold** |
| --- | --- | --- | --- | --- | --- |
| **a) H-*ras*^-/-^** | |  |  |  |  |
| 97520_s_at | Nnat | Neuronatin | 10.225 | 4.58E-06 | 4.366 |
| 94853_at | Gnb1 | Guanine nucleotide binding protein, beta 1 | 7.967 | 1.60E-05 | 2.596 |
| 95052_at | C1qdc2 | C1q domain containing 2 | -8.600 | 9.15E-06 | 0.302 |
| 92582_at | Slc1a5 | Solute carrier family 1 (neutral amino acid transporter), member 5 | -6.521 | 2.97E-05 | 0.490 |
| 103671_at | Htatip2 | HIV-1 tat interactive protein 2, homolog (human) | -5.774 | 5.03E-05 | 0.469 |
| 94767_at | Rps11 | Ribosomal protein S11 | -5.774 | 5.26E-05 | 0.442 |
| **b) N-*ras*^-/-^** | |  |  |  |  |
| 100127_at | Crabp2 | Cellular retinoic acid binding protein II | 30.860 | 2.29E-06 | 18.318 |
| 94516_f_at | Penk1 | Preproenkephalin 1 | 22.408 | 4.58E-06 | 30.681 |
| 100471_at | 1110036O03Rik | RIKEN cDNA 1110036O03 gene | 15.762 | 2.06E-05 | 2.217 |
| 95641_at | Plac9 | Placenta specific 9 | 15.139 | 3.20E-05 | 1.681 |
| 92557_at | Hsd17b1 | Hydroxysteroid (17-beta) dehydrogenase 1 | -12.351 | 8.92E-05 | 0.745 |
| **c) H-*ras*^-/-^/N-*ras*^-/-^** | |  |  |  |  |
| 99148_at | Fh1 | Fumarate hydratase 1 | 16.868 | 6.86E-06 | 1.677 |
| 100127_at | Crabp2 | Cellular retinoic acid binding protein II | 12.919 | 2.75E-05 | 10.748 |
| 160127_at | Ccng1 | Cyclin G1 | 12.713 | 3.20E-05 | 8.231^#^ |
| 102887_at | Tnfrsf11b | Tumor necrosis factor receptor superfamily, member 11b (osteoprotegerin) | 12.141 | 4.35E-05 | 3.149^#^ |
| 99457_at | Mki67 | Antigen identified by monoclonal antibody Ki 67 | 11.910 | 5.03E-05 | 2.142 |
| 95978_at | Atp13a3 | ATPase type 13A3 | 11.566 | 6.41E-05 | 3.309 |
| 93626_at | Abcg2 | ATP-binding cassette, sub-family G (WHITE), member 2 | 11.165 | 7.78E-05 | 2.273 |
| 101694_f_at | Myst2 | MYST histone acetyltransferase 2 | 10.368 | 9.15E-05 | 1.197 |
| 99366_at | Pqlc3 | PQ loop repeat containing | 10.064 | 1.08E-04 | 3.199 |
| 104154_at | Trp53 | Transformation related protein 53 | 9.587 | 1.37E-04 | 1.695 |
| 160203_at | Dnajc9 | Dnaj (Hsp40) homolog, subfamily C, member 9 | 9.019 | 1.83E-04 | 2.351 |
| 94896_at | Hnrpab | Heterogeneous nuclear ribonucleoprotein A/B | 8.964 | 1.99E-04 | 1.593 |
| 94362_at | Nras | Neuroblastoma ras oncogene | -11.659 | 5.72E-05 | 0.179 |
| 102048_at | Ankrd1 | Ankyrin repeat domain 1 (cardiac muscle) | -9.660 | 1.33E-04 | 0.058 |
| 95070_at | Nars | Asparaginyl-tRNA synthetase | -8.999 | 1.88E-04 | 0.623 |

**Table S2. Differential gene expression in serum starved, G0-arrested WT fibroblasts after incubation of cell cultures in the presence of serum for 1hour.** List of 182 differentially expressed probesets (162 different genes) identified by means of SAM contrast (FDR=0,09) comparing the microarray-generated transcriptional profile of 24h serum-starved, WT fibroblasts to that of similarly starved, WT fibroblast cultures submitted to subsequent incubation in the presence of 20% FBS for 1 hour. Differentially expressed loci are identified by Affymetrix probeset ID, gene name and symbol, and listed according to their degree of overexpression or repression, quantitated by R.fold value. d(i) is a parameter measuring the statistical distance separating the calculated expression value of each gene probeset from the null hypothesis (no-change). p-value is an statistical measure indicating the probability of random expression for that probeset. R fold is the log2 value of the fold change measuring the overexpression or repression of the probesets in the collection of microarrays. The “Differential expression not kept” column identifies specific fibroblast knockout genotypes (H: H-*ras^-/-^*; N: N-*ras^-/-^* or HN:H-*ras^-/-^*/N-*ras^-/-^*) where no probeset differential expression was detected for that particular probeset under the same experimental conditions.

| **Probeset ID** | **Symbol** | **Gene name** | **d(i)** | **p-value** | **R.fold** | **Differential expression not kept in** |
| --- | --- | --- | --- | --- | --- | --- |
| 160901_at | Fos | FBJ osteosarcoma oncogene | 34.231 | 2.29E-06 | 179.866 |  |
| 104155_f_at | Atf3 | Activating transcription factor 3 | 17.639 | 1.83E-05 | 43.294 |  |
| 99109_at | Ier2 | Immediate early response 2 | 21.616 | 1.14E-05 | 37.834 |  |
| 103990_at | Fosb | FBJ osteosarcoma oncogene B | 20.454 | 1.60E-05 | 33.278 | N |
| 102371_at | Nr4a1 | Nuclear receptor subfamily 4, group A, member 1 | 34.184 | 4.58E-06 | 31.999 |  |
| 102661_at | Egr2 | Early growth response 2 | 22.278 | 6.86E-06 | 31.159 |  |
| 98579_at | Egr1 | Early growth response 1 | 14.036 | 2.29E-05 | 25.791 |  |
| 102362_i_at | Junb | Jun-B oncogene | 22.141 | 9.15E-06 | 21.727 |  |
| 93294_at | Ctgf | Connective tissue growth factor | 4.737 | 3.34E-04 | 20.239 |  |
| 99392_at | Tnfaip3 | Tumor necrosis factor, alpha-induced protein 3 | 2.925 | 2.40E-03 | 19.446 |  |
| 92830_s_at | Zfp36 | Zinc finger protein 36 | 11.476 | 3.43E-05 | 19.247 |  |
| 92777_at | Cyr61 | Cysteine rich protein 61 | 12.724 | 2.52E-05 | 15.174 |  |
| 94147_at | Serpine1 | Serine (or cysteine) peptidase inhibitor, clade E, member 1 | 3.261 | 1.44E-03 | 13.303 |  |
| 104156_r_at | Atf3 | Activating transcription factor 3 | 10.262 | 4.12E-05 | 11.730 |  |
| 160092_at | Ifrd1 | Interferon-related developmental regulator 1 | 8.595 | 5.49E-05 | 11.568 |  |
| 101583_at | Btg2 | B-cell translocation gene 2, anti-proliferative | 11.526 | 3.20E-05 | 11.459 |  |
| 102363_r_at | Junb | Jun-B oncogene | 14.380 | 2.06E-05 | 11.300 |  |
| 101979_at | Gadd45g | Growth arrest and DNA-damage-inducible 45 gamma | 11.618 | 2.97E-05 | 10.951 |  |
| 104598_at | Dusp1 | Dual specificity phosphatase 1 | 7.636 | 6.86E-05 | 10.547 |  |
| 100130_at | Jun | Jun oncogene | 10.762 | 3.66E-05 | 10.217 |  |
| H, N, HN 96254_at | Dnajb1 | DnaJ (Hsp40) homolog, subfamily B, member 1 | 7.779 | 6.63E-05 | 9.988 |  |
| 94384_at | Ier3 | Immediate early response 3 | 6.896 | 9.38E-05 | 9.485 |  |
| 92614_at | Id3 | Inhibitor of DNA binding 3 | 4.683 | 3.55E-04 | 8.183 |  |
| 100050_at | Id1 | Inhibitor of DNA binding 1 | 4.736 | 3.36E-04 | 7.125 | N |
| 93974_at | Errfi1 | ERBB receptor feedback inhibitor 1 | 3.126 | 1.75E-03 | 6.593 |  |
| 99535_at | Ccrn4l | CCR4 carbon catabolite repression 4-like (S. cerevisiae) | 2.862 | 2.69E-03 | 6.473 | H |
| 94378_at | Rgs16 | Regulator of G-protein signaling 16 | 5.245 | 2.15E-04 | 6.207 |  |
| 160834_at | Sertad1 | SERTA domain containing 1 | 7.451 | 7.55E-05 | 5.952 |  |
| 104149_at | Nfkbia | Nuclear factor of kappa light chain gene enhancer in B-cells inhibitor, alpha | 6.654 | 1.10E-04 | 5.596 |  |
| 104509_at | Ch25h | Cholesterol 25-hydroxylase | 7.481 | 7.32E-05 | 5.438 | H, HN |
| 98988_at | Nfkbiz | Nuclear factor of kappa light polypeptide gene enhancer in B-cells inhibitor, zeta | 5.046 | 2.63E-04 | 5.338 |  |
| 93975_at | Errfi1 | ERBB receptor feedback inhibitor 1 | 2.976 | 2.19E-03 | 5.035 | H |
| 101554_at | Nfkbia | Nuclear factor of kappa light chain gene enhancer in B-cells inhibitor, alpha | 6.934 | 9.15E-05 | 5.021 |  |
| 161609_at | Rgs16 | Regulator of G-protein signaling 16 | 4.970 | 2.79E-04 | 4.967 | H |
| 102048_at | Ankrd1 | Ankyrin repeat domain 1 (cardiac muscle) | 2.873 | 2.62E-03 | 4.739 | HN |
| 104712_at | Myc | Myelocytomatosis oncogene | 3.606 | 9.61E-04 | 4.712 |  |
| 92216_at | Smad7 | MAD homolog 7 (Drosophila) | 9.923 | 4.35E-05 | 4.688 |  |
| 92730_at | Hbegf | Heparin-binding EGF-like growth factor | 3.911 | 6.80E-04 | 4.617 |  |
| 98569_at | Slc25a25 | Solute carrier family 25 (mitochondrial carrier, phosphate carrier), member 25 | 11.790 | 2.75E-05 | 4.581 |  |
| 99603_g_at | Klf10 | Kruppel-like factor 10 | 3.133 | 1.73E-03 | 4.575 |  |
| 104640_f_at | 4930553M18Rik | RIKEN cDNA 4930553M18 gene | 5.865 | 1.51E-04 | 4.548 |  |
| 100302_at | Maff | v-maf musculoaponeurotic fibrosarcoma oncogene family, protein F (avian) | 7.573 | 7.09E-05 | 4.415 |  |
| 97890_at | Sgk | Serum/glucocorticoid regulated kinase | 5.707 | 1.58E-04 | 4.379 |  |
| 161666_f_at | Gadd45b | Growth arrest and DNA-damage-inducible 45 beta | 4.188 | 5.06E-04 | 4.317 |  |
| 98946_at | Wsb1 | WD repeat and SOCS box-containing 1 | 5.180 | 2.38E-04 | 4.214 |  |
| 104639_i_at | 4930553M18Rik | RIKEN cDNA 4930553M18 gene | 6.739 | 1.01E-04 | 3.864 |  |
| 102779_at | Gadd45b | Growth arrest and DNA-damage-inducible 45 beta | 4.598 | 3.71E-04 | 3.859 |  |
| 101030_at | Rhob | Ras homolog gene family, member B | 7.372 | 8.01E-05 | 3.839 |  |
| 102737_at | Edn1 | Endothelin 1 | 4.471 | 4.03E-04 | 3.629 |  |
| 102955_at | Nfil3 | Nuclear factor, interleukin 3, regulated | 5.723 | 1.56E-04 | 3.581 |  |
| 102401_at | Irf1 | Interferon regulatory factor 1 | 4.827 | 3.11E-04 | 3.522 |  |
| 100946_at | Hspa1b | Heat shock protein 1B | 3.842 | 7.46E-04 | 3.507 | HN |
| 101973_at | Cited2 | Cbp/p300-interacting transactivator, with Glu/Asp-rich carboxy-terminal domain, 2 | 3.470 | 1.11E-03 | 3.440 |  |
| 101002_at | Azin1 | antizyme inhibitor 1 | 6.543 | 1.14E-04 | 3.412 |  |
| 98083_at | Klf6 | Kruppel-like factor 6 | 4.945 | 2.93E-04 | 3.396 |  |
| 92534_at | Gem | GTP binding protein (gene overexpressed in skeletal muscle) | 4.223 | 4.87E-04 | 3.268 | HN |
| 92310_at | Plk2 | polo-like kinase 2 (Drosophila) | 2.895 | 2.52E-03 | 3.262 |  |
| 93858_at | Cxcl10 | Chemokine (C-X-C motif) ligand 10 | 3.058 | 1.95E-03 | 3.251 | H, HN |
| 99923_at | 2500001H09Rik | RIKEN cDNA 2500001H09 gene | 3.347 | 1.28E-03 | 3.223 |  |
| 93274_at | Clk1 | CDC-like kinase 1 | 6.552 | 1.12E-04 | 3.195 | HN |
| 160463_at | Myd116 | Myeloid differentiation primary response gene 116 | 4.971 | 2.77E-04 | 3.154 | HN |
| 99621_s_at | Sfpq | Splicing factor proline/glutamine rich (polypyrimidine tract binding protein associated) | 4.964 | 2.86E-04 | 3.140 |  |
| 94761_at | Ccl7 | Chemokine (C-C motif) ligand 7 | 3.120 | 1.76E-03 | 3.118 | HN |
| 94805_f_at | Hist3h2a | Histone 3, H2a | 2.880 | 2.57E-03 | 3.070 | H |
| 93875_at | Hspa1a | Heat shock protein 1A | 3.579 | 9.93E-04 | 3.038 | N, HN |
| 93093_at | Mcl1 | Myeloid cell leukemia sequence 1 | 6.250 | 1.24E-04 | 2.973 |  |
| 93697_at | Cbx4 | Chromobox homolog 4 (Drosophila Pc class) | 4.955 | 2.88E-04 | 2.905 | N |
| 93573_at | Mt1 | Metallothionein 1 | 3.527 | 1.05E-03 | 2.848 |  |
| 101937_s_at | Clk4 | CDC like kinase 4 | 3.864 | 7.30E-04 | 2.728 |  |
| 160588_at | Zfp131 | Zinc finger protein 131 | 5.273 | 2.10E-04 | 2.651 | N |
| 101561_at | Mt2 | Metallothionein 2 | 2.851 | 2.75E-03 | 2.651 |  |
| 93985_at | Tiparp | TCDD-inducible poly(ADP-ribose) polymerase | 4.722 | 3.39E-04 | 2.646 |  |
| 100599_at | Atf4 | Activating transcription factor 4 | 6.750 | 9.84E-05 | 2.526 | HN |
| 98624_at | Rnpc1 | RNA-binding region (RNP1, RRM) containing 1 | 2.859 | 2.70E-03 | 2.498 | N, HN |
| 98066_r_at | Brd2 | Bromodomain containing 2 | 5.817 | 1.53E-04 | 2.401 |  |
| 93853_at | Dnajb4 | DnaJ (Hsp40) homolog, subfamily B, member 4 | 6.765 | 9.61E-05 | 2.389 |  |
| 103980_at | Epha2 | Eph receptor A2 | 5.394 | 2.01E-04 | 2.374 |  |
| 97838_at | Rnu22 | RNA, U22 small nucleolar | 3.374 | 1.23E-03 | 2.352 | N |
| 92608_at | Csrp1 | Cysteine and glycine-rich protein 1 | 2.834 | 2.82E-03 | 2.339 |  |
| 99628_at | Sfrs5 | Splicing factor, arginine/serine-rich 5 (SRp40, HRS) | 8.201 | 6.18E-05 | 2.281 |  |
| 103699_i_at | Frat2 | Frequently rearranged in advanced T-cell lymphomas 2 | 4.443 | 4.07E-04 | 2.280 | H, N, HN |
| 102736_at | Ccl2 | Chemokine (C-C motif) ligand 2 | 3.801 | 7.85E-04 | 2.278 | H, HN |
| 92925_at | Cebpb | CCAAT/enhancer binding protein (C/EBP), beta | 3.821 | 7.69E-04 | 2.237 |  |
| 96083_s_at | Hnrpdl | Heterogeneous nuclear ribonucleoprotein D-like | 5.059 | 2.61E-04 | 2.229 | N, HN |
| 97740_at | Dusp16 | Dual specificity phosphatase 16 | 4.132 | 5.35E-04 | 2.216 | H |
| 94017_s_at | Sfrs2 | Splicing factor, arginine/serine-rich 2 (SC-35) | 2.999 | 2.12E-03 | 2.213 |  |
| 161281_f_at | Ier3 | Immediate early response 3 | 3.294 | 1.38E-03 | 2.195 |  |
| 94476_at | 4930553M18Rik | RIKEN cDNA 4930553M18 gene | 3.670 | 8.92E-04 | 2.189 | HN |
| 93309_at | Ddx3x | DEAD/H (Asp-Glu-Ala-Asp/His) box polypeptide 3, X-linked | 3.206 | 1.53E-03 | 2.175 | N |
| 161681_i_at | Sars2 | Seryl-aminoacyl-tRNA synthetase 2 | 5.703 | 1.60E-04 | 2.161 | N |
| 95486_at | Ptdsr | Phosphatidylserine receptor | 4.589 | 3.73E-04 | 2.141 | HN |
| 103082_at | E230022H04Rik | RIKEN cDNA E230022H04 gene | 4.584 | 3.75E-04 | 2.133 | H, N, HN |
| 92219_s_at | Mid1 | Midline 1 | 3.049 | 1.97E-03 | 2.129 | H, N, HN |
| 94008_at | Tceb1 | Transcription elongation factor B (SIII), polypeptide 1 | 4.010 | 6.06E-04 | 2.124 |  |
| 104745_at | Arl6ip2 | ADP-ribosylation factor-like 6 interacting protein 2 | 2.869 | 2.65E-03 | 2.121 | N, HN |
| 102102_at | NA | NA | 4.125 | 5.45E-04 | 2.100 | H, N, HN |
| 94964_at | Vcl | Vinculin | 2.833 | 2.83E-03 | 2.090 | H |
| 94088_at | Ptbp2 | Polypyrimidine tract binding protein 2 | 4.018 | 6.04E-04 | 2.065 | N |
| 96755_at | Etf1 | Eukaryotic translation termination factor 1 | 3.713 | 8.56E-04 | 2.060 |  |
| 94448_at | Bcl10 | B-cell leukemia/lymphoma 10 | 5.517 | 1.85E-04 | 2.040 | HN |
| 93619_at | Per1 | period homolog 1 (Drosophila) | 5.385 | 2.04E-04 | 2.035 |  |
| 93267_at | Rnpc2 | RNA-binding region (RNP1, RRM) containing 2 | 3.736 | 8.26E-04 | 2.027 | H, N, HN |
| 103015_at | Bcl6 | B-cell leukemia/lymphoma 6 | 3.219 | 1.51E-03 | 2.020 | H, N, HN |
| 102738_s_at | Edn1 | Endothelin 1 | 3.517 | 1.07E-03 | 2.007 | HN |
| 102379_at | Rassf1 | Ras association (ralgds/AF-6) domain family 1 | 4.442 | 4.10E-04 | 2.003 | H, HN |
| 100986_at | Fhl2 | Four and a half LIM domains 2 | 3.958 | 6.47E-04 | 1.982 | HN |
| 96841_at | Pim3 | Proviral integration site 3 | 5.504 | 1.90E-04 | 1.965 | HN |
| 104645_at | Klf7 | Kruppel-like factor 7 (ubiquitous) | 3.949 | 6.52E-04 | 1.960 | H |
| 103830_at | Snai1 | Snail homolog 1 (Drosophila) | 4.192 | 5.03E-04 | 1.958 |  |
| 92233_at | 1810007M14Rik | RIKEN cDNA 1810007M14 gene | 3.645 | 9.22E-04 | 1.946 | H, N, HN |
| 93670_at | Erf | Ets2 repressor factor | 2.886 | 2.56E-03 | 1.946 | HN |
| 94752_s_at | Skil | SKI-like | 3.137 | 1.71E-03 | 1.923 | N |
| 98151_s_at | Ctnnd1 | Catenin (cadherin associated protein), delta 1 | 2.956 | 2.27E-03 | 1.904 | N |
| 160313_at | A730098D12Rik | RIKEN cDNA A730098D12 gene | 3.466 | 1.12E-03 | 1.884 | N, HN |
| 102364_at | Jund1 | Jun proto-oncogene related gene d1 | 3.340 | 1.29E-03 | 1.881 |  |
| 101542_f_at | Ddx3x | DEAD/H (Asp-Glu-Ala-Asp/His) box polypeptide 3, X-linked | 2.964 | 2.23E-03 | 1.865 | N |
| 96754_s_at | Etf1 | Eukaryotic translation termination factor 1 | 6.174 | 1.26E-04 | 1.862 | HN |
| 103091_at | Relb | Avian reticuloendotheliosis viral (v-rel) oncogene related B | 3.508 | 1.08E-03 | 1.837 | H, HN |
| 99162_at | Smndc1 | Survival motor neuron domain containing 1 | 3.299 | 1.37E-03 | 1.823 | H, N |
| 92398_at | Vps37b | Vacuolar protein sorting 37B (yeast) | 7.329 | 8.47E-05 | 1.817 | HN |
| 98990_at | Bcar1 | breast cancer anti-estrogen resistance 1 | 3.388 | 1.21E-03 | 1.809 |  |
| 97892_at | Brd8 | Bromodomain containing 8 | 4.768 | 3.29E-04 | 1.791 | H, N, HN |
| 161557_at | Mnat1 | Menage a trois 1 | 4.562 | 3.80E-04 | 1.775 | N |
| 160451_at | Etf1 | Eukaryotic translation termination factor 1 | 3.877 | 7.12E-04 | 1.770 | H, HN |
| 97273_at | MGI:1933527 | Arsenate resistance protein 2 | 3.038 | 1.99E-03 | 1.770 | HN |
| 101422_at | Fnbp4 | Formin binding protein 4 | 4.258 | 4.71E-04 | 1.765 |  |
| 101805_f_at | Nfil3 | Nuclear factor, interleukin 3, regulated | 4.552 | 3.87E-04 | 1.763 |  |
| 94434_at | Zfp95 | Zinc finger protein 95 | 5.231 | 2.17E-04 | 1.755 | HN |
| 100516_at | Chka | Choline kinase alpha | 3.418 | 1.18E-03 | 1.754 | N, HN |
| 96174_at | Pom121 | Nuclear pore membrane protein 121 | 3.827 | 7.64E-04 | 1.746 | N, HN |
| 103263_at | Epc1 | Enhancer of polycomb homolog 1 (Drosophila) | 3.313 | 1.35E-03 | 1.723 | H, N, HN |
| 102239_at | Bcl3 | B-cell leukemia/lymphoma 3 | 2.923 | 2.41E-03 | 1.712 | H |
| 103927_at | C80731 | Expressed sequence C80731 | 2.939 | 2.34E-03 | 1.701 |  |
| 92355_at | Zfpm2 | Zinc finger protein, multitype 2 | 3.375 | 1.23E-03 | 1.689 | HN |
| 93538_at | Ttrap | Traf and Tnf receptor associated protein | 3.328 | 1.32E-03 | 1.689 | H, N, HN |
| 160265_at | Eif5 | Eukaryotic translation initiation factor 5 | 3.372 | 1.24E-03 | 1.688 | H, N, HN |
| 97919_at | Prpf38b | PRP38 pre-mRNA processing factor 38 (yeast) domain containing B | 2.848 | 2.76E-03 | 1.661 | H, N, HN |
| 160364_at | Sfrs10 | Splicing factor, arginine/serine-rich 10 (transformer 2 homolog, Drosophila) | 3.190 | 1.58E-03 | 1.652 | H, N |
| 102919_at | Mafk | v-maf musculoaponeurotic fibrosarcoma oncogene family, protein K (avian) | 3.003 | 2.10E-03 | 1.648 | H |
| 160225_at | Gtf2b | General transcription factor IIB | 3.189 | 1.58E-03 | 1.638 | H, N, HN |
| 104261_at | Asxl1 | Additional sex combs like 1 (Drosophila) | 2.847 | 2.76E-03 | 1.628 | N, HN |
| 98608_at | Etf1 | Eukaryotic translation termination factor 1 | 3.277 | 1.41E-03 | 1.625 | HN |
| 95033_at | Jmjd1a | Jumonji domain containing 1A | 2.893 | 2.53E-03 | 1.623 | H, N, HN |
| 103989_at | Prdm5 | PR domain containing 5 | 4.409 | 4.19E-04 | 1.620 | H, N |
| 104666_at | Zfp653 | Zinc finger protein 653 | 3.645 | 9.24E-04 | 1.613 | H |
| 160645_at | Birc3 | Baculoviral IAP repeat-containing 3 | 3.144 | 1.69E-03 | 1.608 | H, N, HN |
| 95077_at | Rabggtb | RAB geranylgeranyl transferase, b subunit | 3.047 | 1.98E-03 | 1.587 | H, N, HN |
| 162125_f_at | Ubc | Ubiquitin C | 3.880 | 7.09E-04 | 1.581 |  |
| 95563_at | Arih1 | Ariadne ubiquitin-conjugating enzyme E2 binding protein homolog 1 (Drosophila) | 2.934 | 2.37E-03 | 1.581 | H, N, HN |
| 99982_at | Nfkbib | Nuclear factor of kappa light chain gene enhancer in B-cells inhibitor, beta | 3.459 | 1.13E-03 | 1.578 | H |
| 160842_at | Kin | Antigenic determinant of rec-A protein | 4.699 | 3.50E-04 | 1.571 | H, N |
| 94010_g_at | Tceb1 | Transcription elongation factor B (SIII), polypeptide 1 | 4.099 | 5.56E-04 | 1.569 | H, N |
| 103023_at | Lcat | Lecithin cholesterol acyltransferase | 3.825 | 7.66E-04 | 1.567 | N, HN |
| 162018_r_at | Cryge | Crystallin, gamma E | 3.139 | 1.70E-03 | 1.558 | HN |
| 96730_at | Tpp2 | Tripeptidyl peptidase II | 3.406 | 1.19E-03 | 1.557 | H, N, HN |
| 101996_at | Ptpn2 | Protein tyrosine phosphatase, non-receptor type 2 | 3.361 | 1.25E-03 | 1.557 | H, N, HN |
| 94898_at | Rbm18 | RNA binding motif protein 18 | 2.900 | 2.51E-03 | 1.554 | H, N, HN |
| 94009_at | Tceb1 | Transcription elongation factor B (SIII), polypeptide 1 | 4.708 | 3.48E-04 | 1.552 | N |
| M12481_5_at | Actb | Actin, beta, cytoplasmic | 4.372 | 4.23E-04 | 1.546 | H |
| 99987_at | Zfp574 | Zinc finger protein 574 | 3.330 | 1.32E-03 | 1.531 | H, N, HN |
| 93621_at | Tgif2 | TGFB-induced factor 2 | 3.669 | 8.97E-04 | 1.530 | N |
| 99384_at | Pim1 | Proviral integration site 1 | 2.947 | 2.30E-03 | 1.524 | H, N, HN |
| 160162_at | Tagln2 | Transgelin 2 | 3.169 | 1.64E-03 | 1.521 | H |
| 103255_at | Traf5 | Tnf receptor-associated factor 5 | 3.382 | 1.22E-03 | 1.520 | N, HN |
| 104364_at | Mapkapk5 | MAP kinase-activated protein kinase 5 | 3.638 | 9.36E-04 | 1.506 | N, HN |
| 95215_f_at | Ubc | Ubiquitin C | 3.655 | 9.08E-04 | 1.498 | HN |
| 98882_s_at | Ndel1 | Nuclear distribution gene E-like homolog 1 (A. nidulans) | 3.420 | 1.17E-03 | 1.495 | HN |
| 100469_at | Nfya | Nuclear transcription factor-Y alpha | 3.593 | 9.79E-04 | 1.474 | N |
| 98868_at | Bcl2 | B-cell leukemia/lymphoma 2 | 2.895 | 2.52E-03 | 1.470 | H, N |
| 92432_at | Zfp574 | Zinc finger protein 574 | 3.337 | 1.30E-03 | 1.467 | H, HN |
| 100127_at | Crabp2 | Cellular retinoic acid binding protein II | 2.930 | 2.38E-03 | 1.467 |  |
| 102018_at | Cnot3 | CCR4-NOT transcription complex, subunit 3 | 2.990 | 2.15E-03 | 1.442 | H, N, HN |
| 95600_at | Arih2 | Ariadne homolog 2 (Drosophila) | 3.126 | 1.75E-03 | 1.423 | H, N, HN |
| 95482_at | Usp7 | Ubiquitin specific peptidase 7 | 3.674 | 8.85E-04 | 1.422 | H, N |
| 96305_at | C77604 | Expressed sequence C77604 | 3.215 | 1.51E-03 | 1.410 | H, N, HN |
| M12481_M_at | Actb | Actin, beta, cytoplasmic | 4.267 | 4.67E-04 | 1.409 | H, N |
| 93741_at | Nmd3 | NMD3 homolog (S. cerevisiae) | 2.955 | 2.29E-03 | 1.399 | N |
| 97284_at | Bcl2l13 | BCL2-like 13 (apoptosis facilitator) | 2.876 | 2.61E-03 | 1.378 | N |
| 92872_at | Necap1 | NECAP endocytosis associated 1 | 3.271 | 1.42E-03 | 1.360 | H, N, HN |
| 96109_at | Klf2 | Kruppel-like factor 2 (lung) | 3.455 | 1.13E-03 | 1.356 |  |
| 104150_at | Lrch4 | Leucine-rich repeats and calponin homology (CH) domain containing 4 | 2.992 | 2.15E-03 | 1.312 | H, N, HN |
| 92664_at | 2500002G23Rik | RIKEN cDNA 2500002G23 gene | 3.277 | 1.41E-03 | 1.289 | H, N, HN |

**Table S3. Differential gene expression in serum starved, G0-arrested WT fibroblasts after stimulation with serum for 8hours.** List of 297 differentially expressed gene probesets (284 different genes) identified by means of SAM contrast (FDR=0,09) comparing the microarray-generated transcriptional profile of WT fibroblasts that had been serum-starved for 24h to that of similarly starved, WT fibroblast cultures submitted to subsequent incubation in the presence of 20% FBS for 8 hours. The differentially expressed loci are identified by Affymetrix probeset ID, gene name and symbol, and listed according to their degree of overexpression or repression, quantitated by R.fold value. d(i) is a parameter measuring the statistical distance separating the calculated expression value of each gene probeset from the null hypothesis (no-change). p-value is an statistical measure indicating the probability of random expression for that probeset. R fold is the log2 value of the fold change measuring the overexpression or repression of the probesets in the collection of microarrays. The “Differential expression not kept” column identifies specific fibroblast knockout genotypes (H: H-*ras^-/-^*; N: N-*ras^-/-^* or HN:H-*ras^-/-^*/N-*ras^-/-^*) where no differential expression was detected for that particular probeset under the same experimental conditions.

| **Probeset ID** | **Symbol** | **Gene name** | **d.value** | **p.value** | **R.fold** | **Differential expression not kept in** |
| --- | --- | --- | --- | --- | --- | --- |
| 93294_at | Ctgf | Connective tissue growth factor | 12.343 | 2.97E-05 | 19.376 |  |
| 94147_at | Serpine1 | Serine (or cysteine) peptidase inhibitor, clade E, member 1 | 8.691 | 2.22E-04 | 17.125 |  |
| 92978_s_at | Serpinb2 | Serine (or cysteine) peptidase inhibitor, clade B, member 2 | 6.660 | 9.22E-04 | 15.182 |  |
| 98501_at | Il1rl1 | Interleukin 1 receptor-like 1 | 5.920 | 1.66E-03 | 14.622 |  |
| 104647_at | Ptgs2 | Prostaglandin-endoperoxide synthase 2 | 5.442 | 2.51E-03 | 9.985 |  |
| 98802_at | Ereg | Epiregulin | 5.571 | 2.23E-03 | 9.108 | HN |
| 100050_at | Id1 | Inhibitor of DNA binding 1 | 6.637 | 9.36E-04 | 7.751 | HN |
| 101464_at | Timp1 | Tissue inhibitor of metalloproteinase 1 | 10.888 | 5.72E-05 | 6.247 |  |
| 92730_at | Hbegf | Heparin-binding EGF-like growth factor | 7.206 | 6.29E-04 | 5.224 | N |
| 99835_at | Fosl1 | Fos-like antigen 1 | 6.853 | 8.17E-04 | 4.991 |  |
| 92777_at | Cyr61 | Cysteine rich protein 61 | 8.146 | 3.43E-04 | 4.391 |  |
| 92216_at | Smad7 | MAD homolog 7 (Drosophila) | 8.826 | 1.99E-04 | 4.050 |  |
| 103005_s_at | Cd44 | CD44 antigen | 6.656 | 9.24E-04 | 4.012 |  |
| 96849_at | Timm8a | Translocase of inner mitochondrial membrane 8 homolog a (yeast) | 6.680 | 9.13E-04 | 3.977 | N |
| 92614_at | Id3 | Inhibitor of DNA binding 3 | 5.727 | 1.97E-03 | 3.706 | N, HN |
| 97237_at | 1810003N24Rik | RIKEN cDNA 1810003N24 gene | 6.871 | 8.10E-04 | 3.587 |  |
| 102663_at | Plaur | Urokinase plasminogen activator receptor | 6.170 | 1.33E-03 | 3.577 | N |
| 92794_f_at | Nme1 | Expressed in non-metastatic cells 1, protein | 7.233 | 6.13E-04 | 3.557 |  |
| 92564_at | Lrrfip1 | Leucine rich repeat (in FLII) interacting protein 1 | 5.634 | 2.14E-03 | 3.410 | N |
| 162362_f_at | Tnc | Tenascin C | 5.800 | 1.83E-03 | 3.402 | N, HN |
| 96297_at | Ebna1bp2 | EBNA1 binding protein 2 | 5.668 | 2.09E-03 | 3.253 | N |
| 99475_at | Socs2 | Suppressor of cytokine signaling 2 | 6.029 | 1.51E-03 | 3.243 | N |
| 104712_at | Myc | Myelocytomatosis oncogene | 9.446 | 1.17E-04 | 3.221 | N |
| 96778_at | Rrs1 | RRS1 ribosome biogenesis regulator homolog (S. Cerevisiae) | 7.273 | 5.95E-04 | 3.107 | N |
| 97819_at | Gsto1 | Glutathione S-transferase omega 1 | 5.439 | 2.53E-03 | 3.084 | N, HN |
| 102315_at | Tex292 | Testis expressed gene 292 | 8.662 | 2.31E-04 | 3.076 |  |
| 100125_at | Pa2g4 | Proliferation-associated 2G4 | 10.619 | 6.63E-05 | 3.062 |  |
| 95109_at | Nol5a | Nucleolar protein 5A | 5.090 | 3.23E-03 | 3.042 | N |
| 94452_g_at | Nsun2 | NOL1/NOP2/Sun domain family 2 | 8.620 | 2.33E-04 | 2.975 |  |
| 94450_at | Nsun2 | NOL1/NOP2/Sun domain family 2 | 7.576 | 4.74E-04 | 2.936 |  |
| 99602_at | Klf10 | Kruppel-like factor 10 | 6.700 | 9.01E-04 | 2.924 |  |
| 103039_at | Itga5 | Integrin alpha 5 (fibronectin receptor alpha) | 6.475 | 1.03E-03 | 2.924 | N |
| 92569_f_at | Nol5 | Nucleolar protein 5 | 5.942 | 1.64E-03 | 2.861 | N |
| 99603_g_at | Klf10 | Kruppel-like factor 10 | 5.403 | 2.58E-03 | 2.843 |  |
| 94361_at | Ddx21 | DEAD (Asp-Glu-Ala-Asp) box polypeptide 21 | 7.052 | 7.05E-04 | 2.828 | N |
| 95431_at | Tomm70a | Translocase of outer mitochondrial membrane 70 homolog A (yeast) | 5.580 | 2.21E-03 | 2.822 | N |
| 93803_at | Psme3 | Proteaseome (prosome, macropain) 28 subunit, 3 | 5.723 | 1.98E-03 | 2.806 | N |
| 95657_f_at | Atp6v1f | ATPase, H+ transporting, V1 subunit F | 6.065 | 1.47E-03 | 2.745 | N |
| 103853_at | Grwd1 | Glutamate-rich WD repeat containing 1 | 6.070 | 1.46E-03 | 2.725 |  |
| 102280_at | Pcdh7 | Protocadherin 7 | 6.851 | 8.19E-04 | 2.689 |  |
| 160227_s_at | Bysl | Bystin-like | 5.641 | 2.13E-03 | 2.642 |  |
| 160878_at | Bop1 | Block of proliferation 1 | 6.072 | 1.46E-03 | 2.637 |  |
| 96146_at | Btg3 | B-cell translocation gene 3 | 5.515 | 2.35E-03 | 2.593 | N, HN |
| 160092_at | Ifrd1 | Interferon-related developmental regulator 1 | 9.103 | 1.62E-04 | 2.583 | N |
| 94451_at | Nsun2 | NOL1/NOP2/Sun domain family 2 | 7.362 | 5.58E-04 | 2.547 |  |
| 94860_at | Timm17a | Translocator of inner mitochondrial membrane 17a | 6.973 | 7.48E-04 | 2.510 | N |
| 100323_at | Amd2 | S-adenosylmethionine decarboxylase 2 | 6.288 | 1.19E-03 | 2.501 |  |
| 98850_at | Tll1 | Tolloid-like | 6.192 | 1.31E-03 | 2.498 | N, HN |
| 93235_at | BB128963 | Expressed sequence BB128963 | 5.723 | 1.98E-03 | 2.439 | H, N, HN |
| 97305_at | 1110017C15Rik | RIKEN cDNA 1110017C15 gene | 5.947 | 1.63E-03 | 2.437 | N |
| 95656_i_at | Atp6v1f | ATPase, H+ transporting, V1 subunit F | 6.128 | 1.38E-03 | 2.421 | N |
| 97451_at | Mcfd2 | Multiple coagulation factor deficiency 2 | 7.318 | 5.70E-04 | 2.419 | N |
| 100618_f_at | Slc25a5 | Solute carrier family 25 (mitochondrial carrier, adenine nucleotide translocator), member 5 | 6.619 | 9.47E-04 | 2.365 |  |
| 160308_at | Msn | Moesin | 8.524 | 2.56E-04 | 2.358 | N |
| 93980_at | Rbm13 | RNA binding motif protein 13 | 5.862 | 1.74E-03 | 2.339 | N |
| 94489_at | Ptp4a1 | Protein tyrosine phosphatase 4a1 | 5.838 | 1.78E-03 | 2.334 | N, HN |
| 98132_at | Cycs | Cytochrome c, somatic | 6.234 | 1.25E-03 | 2.332 |  |
| 94486_at | AA959742 | Expressed sequence AA959742 | 5.734 | 1.95E-03 | 2.332 |  |
| 96647_at | 4833420K19Rik | RIKEN cDNA 4833420K19 gene | 5.031 | 3.40E-03 | 2.332 | N |
| 93277_at | Hspd1 | Heat shock protein 1 (chaperonin) | 5.287 | 2.79E-03 | 2.331 | N |
| 160539_at | Sfrs1 | Splicing factor, arginine/serine-rich 1 (ASF/SF2) | 5.668 | 2.09E-03 | 2.311 | H, N |
| 160503_at | Fbl | Fibrillarin | 6.036 | 1.50E-03 | 2.219 | H, N |
| 96804_at | Nol1 | Nucleolar protein 1 | 5.845 | 1.76E-03 | 2.203 |  |
| 94510_at | Wdr77 | WD repeat domain 77 | 6.909 | 7.80E-04 | 2.189 | N |
| 104326_at | Adpgk | ADP-dependent glucokinase | 5.872 | 1.73E-03 | 2.184 | H, N |
| 95791_s_at | Sfrs2 | Splicing factor, arginine/serine-rich 2 (SC-35) | 5.569 | 2.23E-03 | 2.171 |  |
| 99138_at | Rcc1 | Regulator of chromosome condensation 1 | 5.104 | 3.19E-03 | 2.156 | N |
| 92580_at | Hars | Histidyl-tRNA synthetase | 9.122 | 1.58E-04 | 2.147 |  |
| 96756_at | 1110007M04Rik | RIKEN cDNA 1110007M04 gene | 6.100 | 1.43E-03 | 2.146 |  |
| 95735_at | Nolc1 | Nucleolar and coiled-body phosphoprotein 1 | 5.895 | 1.69E-03 | 2.143 | N |
| 101982_at | Vasp | Vasodilator-stimulated phosphoprotein | 5.964 | 1.59E-03 | 2.117 | HN |
| 160802_at | Ppan | Peter pan homolog (Drosophila) | 6.548 | 9.88E-04 | 2.112 | N |
| 160162_at | Tagln2 | Transgelin 2 | 8.329 | 2.91E-04 | 2.109 |  |
| 96755_at | Etf1 | Eukaryotic translation termination factor 1 | 5.976 | 1.58E-03 | 2.083 | N |
| 95543_at | Tpm4 | Tropomyosin 4 | 7.206 | 6.27E-04 | 2.076 | N |
| 160310_at | D19Bwg1357e | DNA segment, Chr 19, Brigham & Women's Genetics 1357 expressed | 5.274 | 2.82E-03 | 2.065 | H |
| 102385_at | Wdr43 | WD repeat domain 43 | 6.096 | 1.43E-03 | 2.064 |  |
| 160377_at | Tardbp | TAR DNA binding protein | 5.889 | 1.70E-03 | 2.049 |  |
| 100144_at | Ncl | Nucleolin | 8.350 | 2.88E-04 | 2.047 |  |
| 97964_at | Fkbp11 | FK506 binding protein 11 | 6.296 | 1.18E-03 | 2.036 | H, HN |
| 96531_at | Tbl3 | Transducin (beta)-like 3 | 5.850 | 1.75E-03 | 2.032 | N |
| 102896_at | Dok1 | Docking protein 1 | 5.107 | 3.18E-03 | 2.017 | HN |
| 93058_at | Eif1a | Eukaryotic translation initiation factor 1A | 6.046 | 1.48E-03 | 2.013 |  |
| 160585_at | Bxdc1 | Brix domain containing 1 | 6.128 | 1.38E-03 | 2.011 | N |
| 103620_s_at | Smn1 | Survival motor neuron 1 | 6.637 | 9.38E-04 | 2.006 | N, HN |
| 103385_at | MGI:1929091 | Teratocarcinoma expressed, serine rich | 5.719 | 1.99E-03 | 2.002 | N |
| 160707_at | D10Wsu102e | DNA segment, Chr 10, Wayne State University 102, expressed | 7.740 | 4.23E-04 | 1.997 | N |
| 100622_at | Prdx6 | Peroxiredoxin 6 | 6.083 | 1.44E-03 | 1.996 | N |
| 160962_at | Bag2 | Bcl2-associated athanogene 2 | 6.724 | 8.90E-04 | 1.994 | N, HN |
| 99180_at | Gtpbp4 | GTP binding protein 4 | 9.338 | 1.30E-04 | 1.989 |  |
| 160521_at | 2610020N02Rik | RIKEN cDNA 2610020N02 gene | 6.266 | 1.22E-03 | 1.974 | N |
| 92596_at | Cacybp | Calcyclin binding protein | 8.313 | 2.95E-04 | 1.970 | N |
| 93727_at | Gspt1 | G1 to S phase transition 1 | 5.093 | 3.22E-03 | 1.964 | N |
| 94001_at | Elavl1 | ELAV (embryonic lethal, abnormal vision, Drosophila)-like 1 (Hu antigen R) | 6.333 | 1.14E-03 | 1.963 | N |
| 93741_at | Nmd3 | NMD3 homolog (S. cerevisiae) | 7.847 | 3.96E-04 | 1.956 | N |
| 100332_s_at | Prdx6 | Peroxiredoxin 6 | 5.808 | 1.81E-03 | 1.955 | N, HN |
| 97504_at | Ccnd2 | Cyclin D2 | 6.051 | 1.48E-03 | 1.929 | H, N |
| 160248_at | Fubp1 | Far upstream element (FUSE) binding protein 1 | 5.089 | 3.23E-03 | 1.929 | N, HN |
| 95470_at | Pak1ip1 | PAK1 interacting protein 1 | 5.554 | 2.26E-03 | 1.923 | N, HN |
| 100091_at | Slc35b1 | Solute carrier family 35, member B1 | 5.135 | 3.12E-03 | 1.918 |  |
| 95749_at | Armet | Arginine-rich, mutated in early stage tumors | 6.128 | 1.38E-03 | 1.891 | H |
| 93130_at | 2600005C20Rik | RIKEN cDNA 2600005C20 gene | 5.671 | 2.09E-03 | 1.879 | N |
| 160974_at | AA408556 | Expressed sequence AA408556 | 5.471 | 2.46E-03 | 1.875 |  |
| 160111_at | Eif1ay | Eukaryotic translation initiation factor 1A, Y-linked | 5.363 | 2.64E-03 | 1.823 | H, N |
| 96663_at | Surf6 | Surfeit gene 6 | 5.028 | 3.42E-03 | 1.813 | N, HN |
| 96754_s_at | Etf1 | Eukaryotic translation termination factor 1 | 5.636 | 2.14E-03 | 1.790 | N, HN |
| 160806_at | Stk39 | Serine/threonine kinase 39, STE20/SPS1 homolog (yeast) | 9.006 | 1.76E-04 | 1.782 |  |
| 99639_at | Usp10 | Ubiquitin specific peptidase 10 | 5.594 | 2.19E-03 | 1.779 | H, N |
| 104110_at | Gatad2a | GATA zinc finger domain containing 2A | 6.120 | 1.39E-03 | 1.775 | N |
| 97882_at | Sec61a1 | Sec61 alpha 1 subunit (S. Cerevisiae) | 5.764 | 1.89E-03 | 1.749 |  |
| 98606_s_at | Wars | Tryptophanyl-tRNA synthetase | 6.898 | 7.87E-04 | 1.745 |  |
| 160466_at | Rae1 | RAE1 RNA export 1 homolog (S. pombe) | 5.429 | 2.54E-03 | 1.743 | N, HN |
| 94951_at | Las1l | LAS1-like (S. cerevisiae) | 8.373 | 2.81E-04 | 1.736 | N |
| 101956_at | Ckap4 | Cytoskeleton-associated protein 4 | 5.535 | 2.31E-03 | 1.736 | H, N |
| 98059_s_at | Lmna | Lamin A | 5.701 | 2.02E-03 | 1.717 |  |
| 93918_at | Taf9 | TAF9 RNA polymerase II, TATA box binding protein (TBP)-associated factor | 5.354 | 2.67E-03 | 1.701 | N |
| 101522_at | Tm4sf1 | Transmembrane 4 superfamily member 1 | 6.654 | 9.27E-04 | 1.689 | HN |
| 160239_at | Psmd6 | Proteasome (prosome, macropain) 26S subunit, non-atpase, 6 | 5.091 | 3.22E-03 | 1.680 | N |
| 95601_at | Ubqln1 | Ubiquilin 1 | 6.196 | 1.30E-03 | 1.679 |  |
| 94855_at | Phb | Prohibitin | 6.439 | 1.05E-03 | 1.652 | H, N |
| 96646_at | Usp39 | Ubiquitin specific peptidase 39 | 5.137 | 3.12E-03 | 1.651 | N |
| 160299_at | Rwdd1 | RWD domain containing 1 | 5.130 | 3.14E-03 | 1.635 | H, N |
| 96075_at | Wdr1 | WD repeat domain 1 | 5.165 | 3.05E-03 | 1.629 | N |
| 160362_at | Mat2a | Methionine adenosyltransferase II, alpha | 11.165 | 5.26E-05 | 1.622 | N |
| 101097_at | Mrpl21 | Mitochondrial ribosomal protein L21 | 5.362 | 2.65E-03 | 1.621 | N, HN |
| 160257_at | Fkbp1a | FK506 binding protein 1a | 5.515 | 2.35E-03 | 1.619 | N, HN |
| 98089_at | 1190005P17Rik | RIKEN cDNA 1190005P17 gene | 8.482 | 2.63E-04 | 1.618 | HN |
| 100605_at | Tpm2 | Tropomyosin 2, beta | 5.252 | 2.86E-03 | 1.613 | HN |
| M12481_5_at | Actb | Actin, beta, cytoplasmic | 7.820 | 4.03E-04 | 1.608 | N |
| 100986_at | Fhl2 | Four and a half LIM domains 2 | 5.162 | 3.07E-03 | 1.603 | N, HN |
| 160333_at | 1110008F13Rik | RIKEN cDNA 1110008F13 gene | 10.870 | 5.95E-05 | 1.594 | N, HN |
| 104573_at | Bola2 | BolA-like 2 (E. coli) | 5.881 | 1.72E-03 | 1.581 | H, N |
| 102090_f_at | NA | NA | 5.983 | 1.57E-03 | 1.576 | H, N |
| 94323_at | LOC433253 | NA | 5.243 | 2.88E-03 | 1.571 | N |
| 100469_at | Nfya | Nuclear transcription factor-Y alpha | 5.547 | 2.28E-03 | 1.556 | N |
| 98944_at | Sec23b | SEC23B (S. cerevisiae) | 5.338 | 2.71E-03 | 1.556 |  |
| M12481_M_at | Actb | Actin, beta, cytoplasmic | 14.880 | 1.60E-05 | 1.555 |  |
| 97703_at | Bxdc1 | Brix domain containing 1 | 6.141 | 1.37E-03 | 1.545 | H, N |
| 99159_at | Ppifs | Peptidylprolyl isomerase F, opposite strand transcription unit | 5.098 | 3.21E-03 | 1.542 | N, HN |
| 95091_at | Sec13l1 | SEC13-like 1 (S. Cerevisiae) | 6.565 | 9.75E-04 | 1.540 | N |
| 94931_at | Ufm1 | Ubiquitin-fold modifier 1 | 6.968 | 7.50E-04 | 1.536 | H, HN |
| 162384_f_at | Ccrn4l | CCR4 carbon catabolite repression 4-like (S. cerevisiae) | 6.937 | 7.69E-04 | 1.529 | N |
| 160988_r_at | Fubp1 | Far upstream element (FUSE) binding protein 1 | 6.312 | 1.16E-03 | 1.493 | H, N, HN |
| 95726_at | Mlf2 | Myeloid leukemia factor 2 | 5.049 | 3.35E-03 | 1.484 | N, HN |
| 98404_at | U2af2 | U2 small nuclear ribonucleoprotein auxiliary factor (U2AF) 2 | 5.508 | 2.39E-03 | 1.483 | N |
| 93083_at | Anxa5 | Annexin A5 | 5.266 | 2.83E-03 | 1.476 | N, HN |
| 93812_at | Clns1a | Chloride channel, nucleotide-sensitive, 1A | 5.560 | 2.25E-03 | 1.475 | H |
| 101528_at | Tcea1 | Transcription elongation factor A (SII) 1 | 5.262 | 2.84E-03 | 1.466 | N |
| 101485_at | Ddx48 | DEAD (Asp-Glu-Ala-Asp) box polypeptide 48 | 5.122 | 3.15E-03 | 1.466 |  |
| 101476_at | Pabpn1 | Poly(A) binding protein, nuclear 1 | 6.211 | 1.29E-03 | 1.452 | N, HN |
| 98569_at | Slc25a25 | Solute carrier family 25 (mitochondrial carrier, phosphate carrier), member 25 | 6.145 | 1.36E-03 | 1.436 | N, HN |
| 94508_at | 1810020E01Rik | RIKEN cDNA 1810020E01 gene | 5.257 | 2.85E-03 | 1.436 | H, N, HN |
| 94752_s_at | Skil | SKI-like | 7.545 | 4.90E-04 | 1.396 | N, HN |
| 160381_at | Mrpl50 | Mitochondrial ribosomal protein L50 | 6.250 | 1.24E-03 | 1.393 | H, N, HN |
| 93405_at | E2f3 | E2F transcription factor 3 | 9.946 | 9.38E-05 | 1.374 | N |
| 98990_at | Bcar1 | Breast cancer anti-estrogen resistance 1 | 5.338 | 2.71E-03 | 1.366 | N |
| 94920_at | Pnpo | Pyridoxine 5'-phosphate oxidase | 5.359 | 2.66E-03 | 1.349 | H, N, HN |
| 99169_at | Carm1 | Coactivator-associated arginine methyltransferase 1 | 6.293 | 1.19E-03 | 1.345 | H, N, HN |
| 160423_at | Mrps2 | Mitochondrial ribosomal protein S2 | 9.413 | 1.24E-04 | 1.343 | N |
| 104297_at | Ipo11 | Importin 11 | 5.411 | 2.56E-03 | 1.334 | H, N |
| 96121_at | 1110055N21Rik | RIKEN cDNA 1110055N21 gene | 6.432 | 1.06E-03 | 1.322 |  |
| M12481_3_at | Actb | Actin, beta, cytoplasmic | 11.211 | 4.80E-05 | 1.310 | N, HN |
| 92503_at | Hus1 | Hus1 homolog (S. pombe) | 8.568 | 2.43E-04 | 1.295 | H, N |
| 103651_r_at | Gtf2f2 | General transcription factor IIF, polypeptide 2 | 5.509 | 2.38E-03 | 1.289 | N |
| 100320_at | Kpna4 | Karyopherin (importin) alpha 4 | 5.460 | 2.48E-03 | 1.279 | N |
| 96682_at | St6galnac4 | ST6 (alpha-N-acetyl-neuraminyl-2,3-beta-galactosyl-1,3)-N-acetylgalactosaminide alpha-2,6-sialyltransferase 4 | 6.110 | 1.41E-03 | 1.163 | N, HN |
| 92494_at | Anxa10 | Annexin A10 | 8.866 | 1.90E-04 | 1.149 | N |
| 101209_at | Fcer1a | Fc receptor, IgE, high affinity I, alpha polypeptide | 6.562 | 9.79E-04 | 1.143 | H, N, HN |
| 102990_at | Col3a1 | Procollagen, type III, alpha 1 | -5.651 | 2.11E-03 | 0.132 | H, N |
| 94813_at | Gas1 | Growth arrest specific 1 | -6.194 | 1.31E-03 | 0.152 | N, HN |
| 98478_at | Ccng2 | Cyclin G2 | -6.112 | 1.41E-03 | 0.210 |  |
| 99010_at | Islr | Immunoglobulin superfamily containing leucine-rich repeat | -7.358 | 5.61E-04 | 0.234 | N |
| 102370_at | Dhrs8 | Dehydrogenase/reductase (SDR family) member 8 | -6.501 | 1.01E-03 | 0.283 |  |
| 102292_at | E230016M11Rik | RIKEN cDNA E230016M11 gene | -7.626 | 4.60E-04 | 0.296 |  |
| 95731_at | Sesn1 | Sestrin 1 | -6.956 | 7.62E-04 | 0.299 | N |
| 99024_at | Mxd4 | Max dimerization protein 4 | -9.886 | 9.61E-05 | 0.302 |  |
| 96615_at | Ypel3 | Yippee-like 3 (Drosophila) | -7.158 | 6.45E-04 | 0.315 |  |
| 99188_at | 2010315L10Rik | RIKEN cDNA 2010315L10 gene | -5.999 | 1.55E-03 | 0.337 |  |
| 103504_at | Ssbp2 | Single-stranded DNA binding protein 2 | -7.079 | 6.91E-04 | 0.339 |  |
| 99187_f_at | 2010315L10Rik | RIKEN cDNA 2010315L10 gene | -6.692 | 9.08E-04 | 0.359 |  |
| 93320_at | Cpt1a | Carnitine palmitoyltransferase 1a, liver | -7.256 | 6.04E-04 | 0.360 | N |
| 95161_at | Ctdsp2 | CTD (carboxy-terminal domain, RNA polymerase II, polypeptide A) small phosphatase 2 | -5.719 | 1.99E-03 | 0.378 |  |
| 160522_at | D0H4S114 | DNA segment, human D4S114 | -6.007 | 1.54E-03 | 0.380 | H |
| 96090_g_at | 4931406C07Rik | RIKEN cDNA 4931406C07 gene | -6.120 | 1.39E-03 | 0.396 | N, HN |
| 93007_at | Npy1r | Neuropeptide Y receptor Y1 | -5.229 | 2.92E-03 | 0.401 | N |
| 92958_at | Foxo3a | Forkhead box o3a | -7.115 | 6.61E-04 | 0.411 |  |
| 160547_s_at | Txnip | Thioredoxin interacting protein | -5.808 | 1.81E-03 | 0.413 | N, HN |
| 96728_at | Wdr45 | WD repeat domain 45 | -6.234 | 1.25E-03 | 0.432 |  |
| 94394_at | Rras | Harvey rat sarcoma oncogene, subgroup R | -5.987 | 1.56E-03 | 0.434 |  |
| 160482_at | Acaa1 | Acetyl-Coenzyme A acyltransferase 1 | -5.657 | 2.11E-03 | 0.450 |  |
| 160288_at | Map1lc3b | Microtubule-associated protein 1 light chain 3 beta | -7.626 | 4.58E-04 | 0.458 |  |
| 97398_at | Pck2 | Phosphoenolpyruvate carboxykinase 2 (mitochondrial) | -6.375 | 1.10E-03 | 0.458 | H |
| 161183_at | Dag1 | Dystroglycan 1 | -5.327 | 2.72E-03 | 0.458 | N, HN |
| 101484_at | Nbr1 | Neighbor of Brca1 gene 1 | -5.836 | 1.78E-03 | 0.462 | N |
| 96035_at | Bckdha | Branched chain ketoacid dehydrogenase E1, alpha polypeptide | -13.024 | 2.29E-05 | 0.471 |  |
| 98461_at | Knsl8 | Kinesin-like 8 | -8.301 | 3.04E-04 | 0.473 |  |
| 96240_at | D15Ertd405e | DNA segment, Chr 15, ERATO Doi 405, expressed | -5.967 | 1.59E-03 | 0.473 | HN |
| 92263_at | Leprel2 | Leprecan-like 2 | -5.763 | 1.89E-03 | 0.477 | N, HN |
| 92375_at | Ascc1 | Activating signal cointegrator 1 complex subunit 1 | -6.441 | 1.05E-03 | 0.480 |  |
| 103001_at | Vegfb | Vascular endothelial growth factor B | -6.302 | 1.17E-03 | 0.480 |  |
| 101861_at | Sgce | Sarcoglycan, epsilon | -5.511 | 2.37E-03 | 0.485 | HN |
| 93490_at | Maf1 | MAF1 homolog (S. cerevisiae) | -6.643 | 9.29E-04 | 0.489 |  |
| 100570_at | 6330412F12Rik | RIKEN cDNA 6330412F12 gene | -5.568 | 2.23E-03 | 0.495 | H, N |
| 94976_at | Numa1 | Nuclear mitotic apparatus protein 1 | -5.300 | 2.77E-03 | 0.496 | N, HN |
| MurFAS_at | Fas | Fas (TNF receptor superfamily member) | -8.034 | 3.50E-04 | 0.498 |  |
| 101102_at | Igbp1 | Immunoglobulin (CD79A) binding protein 1 | -6.445 | 1.05E-03 | 0.505 |  |
| 103225_at | Dnase1l1 | Deoxyribonuclease 1-like 1 | -8.684 | 2.24E-04 | 0.510 |  |
| 94982_f_at | Nme3 | Expressed in non-metastatic cells 3 | -6.993 | 7.34E-04 | 0.510 |  |
| 101109_at | Dag1 | Dystroglycan 1 | -5.907 | 1.68E-03 | 0.512 | N, HN |
| 97423_at | 1500035H01Rik | RIKEN cDNA 1500035H01 gene | -6.034 | 1.51E-03 | 0.524 |  |
| 160792_at | Snapap | SNAP-associated protein | -5.739 | 1.94E-03 | 0.524 | N |
| 102094_f_at | Gstm1 | Glutathione S-transferase, mu 1 | -5.583 | 2.21E-03 | 0.525 | H, N |
| 94266_at | Wdr58 | WD repeat domain 58 | -5.768 | 1.88E-03 | 0.526 | N, HN |
| 104409_at | Grik5 | Glutamate receptor, ionotropic, kainate 5 (gamma 2) | -7.563 | 4.83E-04 | 0.530 | N, HN |
| 92348_at | Thra | Thyroid hormone receptor alpha | -5.941 | 1.65E-03 | 0.545 | N |
| 96263_at | Rshl2 | Radial spokehead-like 2 | -6.067 | 1.46E-03 | 0.547 | N |
| 94535_at | Add1 | Adducin 1 (alpha) | -6.547 | 9.91E-04 | 0.549 |  |
| 160602_at | Pde6d | Phosphodiesterase 6D, cgmp-specific, rod, delta | -6.806 | 8.40E-04 | 0.550 | HN |
| 92887_at | Ddah2 | Dimethylarginine dimethylaminohydrolase 2 | -5.478 | 2.45E-03 | 0.552 |  |
| 95095_at | Flot1 | Flotillin 1 | -7.569 | 4.78E-04 | 0.566 | HN |
| 102215_at | Cdv1 | Carnitine deficiency-associated gene expressed in ventricle 1 | -5.411 | 2.56E-03 | 0.568 | HN |
| 103671_at | Htatip2 | HIV-1 tat interactive protein 2, homolog (human) | -5.909 | 1.67E-03 | 0.576 | N, HN |
| 102402_at | Gbas | Glioblastoma amplified sequence | -6.338 | 1.13E-03 | 0.577 | HN |
| 95128_at | Anapc2 | Anaphase promoting complex subunit 2 | -6.469 | 1.03E-03 | 0.583 | N |
| 97224_at | Pnrc1 | Proline-rich nuclear receptor coactivator 1 | -6.280 | 1.20E-03 | 0.585 | N, HN |
| 104372_at | Abhd8 | Abhydrolase domain containing 8 | -5.369 | 2.63E-03 | 0.586 |  |
| 96076_at | Stx5a | Syntaxin 5A | -8.748 | 2.10E-04 | 0.595 | H, N, HN |
| 160287_at | Map1lc3b | Microtubule-associated protein 1 light chain 3 beta | -6.281 | 1.19E-03 | 0.597 | H |
| 92517_at | Rbms2 | RNA binding motif, single stranded interacting protein 2 | -6.017 | 1.53E-03 | 0.597 | N |
| 102059_at | Nicn1 | Nicolin 1 | -5.495 | 2.42E-03 | 0.601 |  |
| 92397_at | Centg2 | Centaurin, gamma 2 | -5.896 | 1.69E-03 | 0.602 | H, N, HN |
| 97928_at | Cln6 | Ceroid-lipofuscinosis, neuronal 6 | -5.239 | 2.89E-03 | 0.605 | N, HN |
| 93972_at | Ndufs2 | NADH dehydrogenase (ubiquinone) Fe-S protein 2 | -9.781 | 1.03E-04 | 0.606 | H, N, HN |
| 93373_at | Naglu | Alpha-N-acetylglucosaminidase (Sanfilippo disease IIIB) | -6.818 | 8.37E-04 | 0.607 | N, HN |
| 95491_at | Park7 | Parkinson disease (autosomal recessive, early onset) 7 | -6.482 | 1.03E-03 | 0.609 | H, N |
| 102836_at | MGI:1194899 | Putative phosphatase | -5.442 | 2.51E-03 | 0.617 | N, HN |
| 103646_at | Crat | Carnitine acetyltransferase | -6.434 | 1.06E-03 | 0.618 |  |
| 104003_at | Phka2 | Phosphorylase kinase alpha 2 | -7.379 | 5.54E-04 | 0.633 | N |
| 98969_at | Abcd1 | ATP-binding cassette, sub-family D (ALD), member 1 | -5.663 | 2.10E-03 | 0.637 |  |
| 94459_at | AI462493 | Expressed sequence AI462493 | -8.173 | 3.32E-04 | 0.643 | N, HN |
| 99938_at | Xrcc1 | X-ray repair complementing defective repair in Chinese hamster cells 1 | -5.619 | 2.16E-03 | 0.648 | HN |
| 96231_at | Bphl | Biphenyl hydrolase-like (serine hydrolase, breast epithelial mucin-associated antigen) | -6.024 | 1.52E-03 | 0.652 | N, HN |
| 103631_at | Zfp688 | Zinc finger protein 688 | -5.942 | 1.64E-03 | 0.656 | HN |
| 101834_at | Mapk3 | Mitogen activated protein kinase 3 | -6.119 | 1.40E-03 | 0.658 | N, HN |
| 160346_at | Wdr6 | WD repeat domain 6 | -6.261 | 1.23E-03 | 0.659 | N, HN |
| 94504_at | 4930570C03Rik | RIKEN cDNA 4930570C03 gene | -5.544 | 2.28E-03 | 0.667 | N, HN |
| 99000_at | Mapk7 | Mitogen activated protein kinase 7 | -7.385 | 5.47E-04 | 0.668 | N, HN |
| 104122_at | D330001F17Rik | RIKEN cDNA D330001F17 gene | -8.411 | 2.72E-04 | 0.669 | N |
| 93539_at | Anapc13 | Anaphase promoting complex subunit 13 | -5.678 | 2.07E-03 | 0.669 |  |
| 160844_at | Pts | 6-pyruvoyl-tetrahydropterin synthase | -5.936 | 1.65E-03 | 0.671 | HN |
| 100122_at | Gnb5 | Guanine nucleotide binding protein, beta 5 | -6.689 | 9.11E-04 | 0.672 |  |
| 96735_at | Stard10 | START domain containing 10 | -5.937 | 1.65E-03 | 0.672 | N |
| 160869_at | Sirt3 | Sirtuin 3 (silent mating type information regulation 2, homolog) 3 (S. Cerevisiae) | -5.482 | 2.44E-03 | 0.676 | HN |
| 95014_at | Fbxo6b | F-box only protein 6b | -7.459 | 5.29E-04 | 0.679 | N |
| 100039_at | Tmem4 | Transmembrane protein 4 | -6.545 | 9.98E-04 | 0.680 | HN |
| 94047_at | 0610031J06Rik | RIKEN cDNA 0610031J06 gene | -5.344 | 2.70E-03 | 0.683 | H, N |
| 94400_at | 1110051M20Rik | RIKEN cDNA 1110051M20 gene | -6.337 | 1.13E-03 | 0.684 | N |
| 104034_at | AI464131 | Expressed sequence AI464131 | -5.682 | 2.05E-03 | 0.684 | H, N |
| 103711_at | Casp9 | Caspase 9 | -5.509 | 2.38E-03 | 0.684 | H, N, HN |
| 161723_at | Wdfy2 | WD repeat and FYVE domain containing 2 | -6.144 | 1.37E-03 | 0.687 | H, N |
| 161286_f_at | Arsa | Arylsulfatase A | -8.378 | 2.79E-04 | 0.692 | HN |
| 160104_at | Hsd3b7 | Hydroxy-delta-5-steroid dehydrogenase, 3 beta- and steroid delta-isomerase 7 | -6.584 | 9.68E-04 | 0.693 | N, HN |
| 92376_at | Rit1 | Ras-like without CAAX 1 | -5.467 | 2.47E-03 | 0.697 | H |
| 96621_at | 1110061L23Rik | RIKEN cDNA 1110061L23 gene | -5.765 | 1.89E-03 | 0.699 | N |
| 92934_at | Zfp90 | Zinc finger protein 90 | -5.573 | 2.22E-03 | 0.702 | H, N, HN |
| 96348_at | Pnpla2 | Patatin-like phospholipase domain containing 2 | -5.586 | 2.20E-03 | 0.712 | N |
| 104482_at | Epim | Epimorphin | -5.810 | 1.81E-03 | 0.716 | H, N, HN |
| 104237_at | Pcbd2 | Pterin 4 alpha carbinolamine dehydratase/dimerization cofactor of hepatocyte nuclear factor 1 alpha (TCF1) 2 | -5.285 | 2.79E-03 | 0.716 | H, N, HN |
| 103811_at | Invs | Inversin | -9.285 | 1.35E-04 | 0.718 | N |
| 160619_at | NA | NA | -6.783 | 8.44E-04 | 0.725 | H, HN |
| 160904_at | B230317C12Rik | RIKEN cDNA B230317C12 gene | -7.683 | 4.44E-04 | 0.728 |  |
| 102802_at | Il18 | Interleukin 18 | -5.610 | 2.17E-03 | 0.728 | N |
| 97533_at | Fcgrt | Fc receptor, IgG, alpha chain transporter | -6.956 | 7.64E-04 | 0.732 | N |
| 102817_at | U2af1-rs1 | U2 small nuclear ribonucleoprotein auxiliary factor (U2AF) 1, related sequence 1 | -7.018 | 7.21E-04 | 0.735 | H |
| 160853_at | Ptdss2 | Phosphatidylserine synthase 2 | -6.490 | 1.02E-03 | 0.736 | N |
| 103408_at | Prkd2 | Protein kinase D2 | -5.673 | 2.08E-03 | 0.741 | N |
| 92411_at | Hs1bp3 | HS1 binding protein 3 | -11.364 | 4.35E-05 | 0.746 | H, N |
| 160685_at | Klhl7 | Kelch-like 7 (Drosophila) | -5.510 | 2.37E-03 | 0.747 | N, HN |
| 92430_at | Chchd5 | Coiled-coil-helix-coiled-coil-helix domain containing 5 | -5.557 | 2.26E-03 | 0.749 | H, N |
| 102991_s_at | H2-Ke6 | H2-K region expressed gene 6 | -6.198 | 1.30E-03 | 0.752 | H, N |
| 93540_at | Adprh | ADP-ribosylarginine hydrolase | -9.490 | 1.12E-04 | 0.753 | H, HN |
| 104439_at | Nat6 | N-acetyltransferase 6 | -6.440 | 1.05E-03 | 0.755 | H, N |
| 161895_s_at | BC054822 | cDNA sequence BC054822 | -7.098 | 6.75E-04 | 0.762 | H, N |
| 102894_g_at | Pou2f1 | POU domain, class 2, transcription factor 1 | -6.044 | 1.49E-03 | 0.771 | H, N, HN |
| 96814_r_at | DXImx46e | DNA segment, Chr X, Immunex 46, expressed | -9.232 | 1.42E-04 | 0.779 | H, N, HN |
| 161018_at | 2310010J17Rik | RIKEN cDNA 2310010J17 gene | -5.526 | 2.33E-03 | 0.780 | N |
| 104609_at | 1110031M08Rik | RIKEN cDNA 1110031M08 gene | -6.161 | 1.34E-03 | 0.785 | H, N, HN |
| 101593_at | Crip2 | Cysteine rich protein 2 | -7.544 | 4.92E-04 | 0.789 | H, N, HN |
| 103988_at | BC023754 | cDNA sequence BC023754 | -5.761 | 1.90E-03 | 0.790 | N, HN |
| 104131_at | Gpr175 | G protein-coupled receptor 175 | -6.013 | 1.53E-03 | 0.798 | H, N, HN |
| 99466_at | Zfp444 | Zinc finger protein 444 | -5.805 | 1.82E-03 | 0.807 | H, N, HN |
| 92318_at | 2010301N04Rik | RIKEN cDNA 2010301N04 gene | -5.504 | 2.40E-03 | 0.807 | N, HN |
| 99905_at | Dub2 | Deubiquitinating enzyme 2 | -6.251 | 1.23E-03 | 0.816 | H, N, HN |
| 95955_at | Ppp2r5e | protein phosphatase 2, regulatory subunit B (B56), epsilon isoform | -5.553 | 2.27E-03 | 0.822 | H, N, HN |
| 93619_at | Per1 | Period homolog 1 (Drosophila) | -5.960 | 1.60E-03 | 0.824 | H, N |
| 161970_f_at | Hmgcl | 3-hydroxy-3-methylglutaryl-Coenzyme A lyase | -8.860 | 1.92E-04 | 0.847 | N, HN |
| 102271_at | D11Bwg0280e | DNA segment, Chr 11, Brigham & Women's Genetics 0280e expressed | -10.448 | 7.32E-05 | 0.849 | N, HN |

**Table S4. Differential gene expression in serum-starved, G0-arrested H-*ras*^-/-^ fibroblast cultures after stimulation with serum for 1 hour.** List of 323 differentially expressed probesets (311 different genes) identified by means of SAM contrast (FDR=0,09) comparing the microarray-generated transcriptional profile of WT fibroblasts that had been serum-starved for 24h to that of similarly starved, H-*ras***^-/-^** fibroblast cultures submitted to subsequent incubation in the presence of 20% FBS for 1 hour. To concentrate on loci exclusively regulated by Ras, genes sharing similar values of differential expression between the WT and H-*ras*^-/-^ fibroblast (ratio of the R-fold values in their respective lists within the range 0.6-1.5) were excluded from this list. The differentially expressed loci are identified by Affymetrix probeset ID, gene name and symbol, and listed according to functional category and degree of overexpression or repression, quantitated by d(i) value. The * and # symbols in the R.fold column denote independent validation of the transcriptional data obtained by means of quantitative RT-PCR or Western immunoblot, respectively. d(i) is a parameter measuring the statistical distance separating the calculated expression value of each gene probeset from the null hypothesis (no-change). p-value is an statistical measure indicating the probability of random expression for that probeset. R fold is the log2 value of the fold change measuring the overexpression or repression of the probesets in the collection of microarrays.

| **Probeset ID** | **Gene symbol** | **Gene name** | **d(i)** | **p-value** | **R.fold** |
| --- | --- | --- | --- | --- | --- |
| *Signal transduction* | |  |  |  |  |
| 93485_at | Ptprd | Protein tyrosine phosphatase, receptor type, D | 7.583 | 7.09E-05 | 2.118 |
| 92205_at | Irs2 | Insulin receptor substrate 2 | 4.915 | 3.64E-04 | 1.936 |
| 94853_at | Gnb1 | Guanine nucleotide binding protein, beta 1 | 4.872 | 3.80E-04 | 2.018 |
| 97319_at | Rrad | Ras-related associated with diabetes | 4.683 | 4.64E-04 | 1.576 |
| 94771_at | Rassf2 | Ras association (RalGDS/AF-6) domain family 2 | 4.248 | 7.09E-04 | 1.774 |
| 98817_at | Fst | Follistatin | 3.948 | 9.54E-04 | 4.834 |
| 92764_at | Ksr | Kinase suppressor of ras | 3.762 | 1.19E-03 | 1.438 |
| 97458_at | Gnb1 | Guanine nucleotide binding protein, beta 1 | 3.384 | 1.97E-03 | 1.863 |
| 92473_at | Mknk2 | MAP kinase-interacting serine/threonine kinase 2 | 3.216 | 2.56E-03 | 1.320 |
| 95721_at | Mapkapk2 | MAP kinase-activated protein kinase 2 | 3.084 | 3.15E-03 | 1.466 |
| 103098_at | Baiap2 | Brain-specific angiogenesis inhibitor 1-associated protein 2 | 2.992 | 3.63E-03 | 1.302 |
| 92362_at | Dusp8 | Dual specificity phosphatase 8 | 2.901 | 4.21E-03 | 1.567 |
| 98403_at | Gna-rs1 | Guanine nucleotide binding protein, related sequence 1 | 2.856 | 4.59E-03 | 1.294 |
| 104108_at | Rab6ip1 | Rab6 interacting protein 1 | -4.888 | 3.73E-04 | 0.533 |
| 98927_at | Rab6 | RAB6, member RAS oncogene family | -4.550 | 5.17E-04 | 0.479 |
| 100122_at | Gnb5 | Guanine nucleotide binding protein, beta 5 | -4.424 | 5.88E-04 | 0.692 |
| 160371_at | Arl6ip1 | ADP-ribosylation factor-like 6 interacting protein 1 | -4.402 | 6.04E-04 | 0.432 |
| 160188_at | Nudt4 | Nudix (nucleoside diphosphate linked moiety X)-type motif 4 | -4.342 | 6.43E-04 | 0.470 |
| 93007_at | Npy1r | Neuropeptide Y receptor Y1 | -4.315 | 6.68E-04 | 0.454 |
| 104461_at | Pik3ca | Phosphatidylinositol 3-kinase, catalytic, alpha polypeptide (p110-alpha) | -3.982 | 9.01E-04 | 0.564 |
| 102196_at | Gna11 | Guanine nucleotide binding protein, alpha 11 | -3.982 | 8.99E-04 | 0.478* |
| 96629_at | Nudt19 | Nudix (nucleoside diphosphate linked moiety X)-type motif 19 | -3.848 | 1.08E-03 | 0.542 |
| 97259_at | Arpp19 | cAMP-regulated phosphoprotein 19 | -3.709 | 1.29E-03 | 0.677 |
| 96728_at | Wdr45 | WD repeat domain 45 | -3.603 | 1.48E-03 | 0.599 |
| 103209_at | Ltbp1 | Latent transforming growth factor beta binding protein 1 | -3.576 | 1.53E-03 | 0.534* |
| 104264_at | Lrba | LPS-responsive beige-like anchor | -3.514 | 1.64E-03 | 0.680 |
| 102374_at | Dscr1l2 | Down syndrome critical region gene 1-like 2 | -3.448 | 1.78E-03 | 0.695 |
| 96751_at | Arl5 | ADP-ribosylation factor-like 5 | -3.330 | 2.13E-03 | 0.493 |
| 99471_at | Stard7 | START domain containing 7 | -3.309 | 2.20E-03 | 0.743* |
| 104300_at | Iqgap1 | IQ motif containing GTPase activating protein 1 | -3.279 | 2.30E-03 | 0.514 |
| 95136_at | Arl6ip4 | ADP-ribosylation factor-like 6 interacting protein 4 | -3.240 | 2.45E-03 | 0.709 |
| 94801_at | Pgrmc2 | Progesterone receptor membrane component 2 | -3.235 | 2.48E-03 | 0.735 |
| 101834_at | Mapk3 | Mitogen activated protein kinase 3 | -3.142 | 2.85E-03 | 0.690* |
| 101113_at | Rhoa | Ras homolog gene family, member A | -3.135 | 2.88E-03 | 0.638 |
| 98993_at | Ppp2r5c | Protein phosphatase 2, regulatory subunit B (B56), gamma isoform | -3.127 | 2.93E-03 | 0.522 |
| 160536_at | **Hras1** | **Harvey rat sarcoma virus oncogene 1** | -3.126 | 2.93E-03 | 0.568 |
| 162447_f_at | Mvp | Major vault protein | -3.019 | 3.48E-03 | 0.811 |
| 93748_at | Grinl1a | Glutamate receptor, ionotropic, N-methyl D-aspartate-like 1A | -2.999 | 3.59E-03 | 0.716 |
| 160189_at | Nudt4 | Nudix (nucleoside diphosphate linked moiety X)-type motif 4 | -2.979 | 3.71E-03 | 0.677 |
| 96103_f_at | Rad23b | RAD23b homolog (S. cerevisiae) | -2.933 | 4.00E-03 | 0.661 |
| 94258_at | Arhgdib | Rho, GDP dissociation inhibitor (GDI) beta | -2.930 | 4.02E-03 | 0.643 |
| *Transcription* | |  |  |  |  |
| 92830_s_at | Zfp36 | Zinc finger protein 36 | 23.120 | 1.37E-05 | 12.085 |
| 99603_g_at | Klf10 | Kruppel-like factor 10 | 5.488 | 2.24E-04 | 2.320 |
| 93013_at | Id2 | Inhibitor of DNA binding 2 | 5.073 | 3.25E-04 | 3.731 |
| 100986_at | Fhl2 | Four and a half LIM domains 2 | 4.805 | 4.10E-04 | 2.067 |
| 99602_at | Klf10 | Kruppel-like factor 10 | 4.472 | 5.58E-04 | 2.365 |
| 103318_at | Gabpb1 | GA repeat binding protein, beta 1 | 4.007 | 8.76E-04 | 1.473 |
| 93669_f_at | Sox11 | SRY-box containing gene 11 | 3.821 | 1.11E-03 | 1.861 |
| 160894_at | Cebpd | CCAAT/enhancer binding protein (C/EBP), delta | 3.735 | 1.23E-03 | 2.598 |
| 93944_r_at | Zfp36l2 | Zinc finger protein 36, C3H type-like 2 | 3.400 | 1.92E-03 | 1.490 |
| 104169_at | Zic1 | Zinc finger protein of the cerebellum 1 | 3.265 | 2.35E-03 | 7.065 |
| 101459_at | Chd1 | Chromodomain helicase DNA binding protein 1 | 3.071 | 3.21E-03 | 1.861 |
| 101727_at | Nfkbie | Nuclear factor of kappa light polypeptide gene enhancer in B-cells inhibitor, epsilon | 3.068 | 3.23E-03 | 1.317 |
| 95750_at | Zc3hc1 | Zinc finger, C3HC type 1 | 2.991 | 3.64E-03 | 1.366 |
| 103437_at | Zfp57 | Zinc finger protein 57 | 2.959 | 3.84E-03 | 1.548 |
| 93878_at | Mllt10 | Myeloid/lymphoid or mixed lineage-leukemia translocation to 10 homolog (Drosophila) | 2.872 | 4.45E-03 | 1.358 |
| 104717_at | Zfp644 | Zinc finger protein 644 | 2.848 | 4.66E-03 | 2.013 |
| 104261_at | Asxl1 | Additional sex combs like 1 (Drosophila) | 3.277 | 2.32E-03 | 1.548 |
| 99024_at | Mxd4 | Max dimerization protein 4 | -8.566 | 5.72E-05 | 0.470 |
| 99052_at | Zfhx1a | Zinc finger homeobox 1a | -4.508 | 5.40E-04 | 0.562 |
| 104228_at | EG668701 | Predicted gene, EG668701 | -4.414 | 5.99E-04 | 0.641 |
| 97893_at | Tbpl1 | TATA box binding protein-like 1 | -4.290 | 6.84E-04 | 0.659 |
| 92554_at | Ctbp2 | C-terminal binding protein 2 | -4.000 | 8.83E-04 | 0.539 |
| 102964_at | Zfp639 | Zinc finger protein 639 | -3.837 | 1.08E-03 | 0.686 |
| 161077_f_at | Smarcd2 | SWI/SNF related, matrix associated, actin dependent regulator of chromatin, subfamily d, member 2 | -3.764 | 1.19E-03 | 0.613 |
| 100148_at | Ctcf | CCCTC-binding factor | -3.606 | 1.47E-03 | 0.610* |
| 100405_at | Cbx3 | Chromobox homolog 3 (Drosophila HP1 gamma) | -3.536 | 1.58E-03 | 0.429 |
| 99111_at | Clpb | ClpB caseinolytic peptidase B homolog (E. coli) | -3.483 | 1.71E-03 | 0.606 |
| 101939_at | Rnf141 | Ring finger protein 141 | -3.431 | 1.83E-03 | 0.710 |
| 95161_at | Ctdsp2 | CTD (carboxy-terminal domain, RNA polymerase II, polypeptide A) small phosphatase 2 | -3.190 | 2.66E-03 | 0.480 |
| 98790_s_at | Meis1 | Myeloid ecotropic viral integration site 1 | -3.157 | 2.80E-03 | 0.689 |
| 161741_r_at | Zfp503 | Zinc finger protein 503 | -3.156 | 2.80E-03 | 0.814 |
| 95755_at | Csda | Cold shock domain protein A | -3.095 | 3.08E-03 | 0.688* |
| 94396_at | Ing1 | Inhibitor of growth family, member 1 | -3.092 | 3.09E-03 | 0.671 |
| 102833_at | Cbx2 | Chromobox homolog 2 (Drosophila Pc class) | -3.083 | 3.16E-03 | 0.724 |
| 98982_at | Tmpo | Thymopoietin | -3.050 | 3.33E-03 | 0.681 |
| 160848_at | Zhx1 | Zinc fingers and homeoboxes protein 1 | -3.022 | 3.46E-03 | 0.473 |
| 96535_at | Zfp499 | Zinc finger protein 499 | -2.921 | 4.11E-03 | 0.715 |
| *Primary cell metabolism* | | |  |  |  |
| 104647_at | Ptgs2 | Prostaglandin-endoperoxide synthase 2 | 3.639 | 1.42E-03 | 11.468 |
| 93824_at | Cps1 | Carbamoyl-phosphate synthetase 1 | 3.266 | 2.35E-03 | 1.311 |
| 160362_at | Mat2a | Methionine adenosyltransferase II, alpha | 3.237 | 2.47E-03 | 2.039* |
| 95738_at | Aldh18a1 | Aldehyde dehydrogenase 18 family, member A1 | 3.168 | 2.75E-03 | 1.637 |
| 92263_at | Leprel2 | Leprecan-like 2 | -5.525 | 2.10E-04 | 0.569 |
| 100574_f_at | Gpi1 | Glucose phosphate isomerase 1 | -4.768 | 4.26E-04 | 0.415 |
| 103367_at | Galgt1 | UDP-N-acetyl-alpha-D-galactosamine:(N-acetylneuraminyl)-galactosylglucosylceramide-beta-1, 4-N-acetylgalactosaminyltransferase | -4.546 | 5.19E-04 | 0.571 |
| 160807_at | Agpat3 | 1-acylglycerol-3-phosphate O-acyltransferase 3 | -4.201 | 7.44E-04 | 0.625 |
| 102993_at | Ggta1 | Glycoprotein galactosyltransferase alpha 1, 3 | -3.921 | 9.93E-04 | 0.607 |
| 102936_at | B4galt6 | UDP-Gal:betaGlcNAc beta 1,4-galactosyltransferase, polypeptide 6 | -3.895 | 1.01E-03 | 0.574 |
| 160204_at | Prr6 | Proline-rich polypeptide 6 | -3.869 | 1.03E-03 | 0.527 |
| 96243_f_at | Aldh9a1 | Aldehyde dehydrogenase 9, subfamily A1 | -3.868 | 1.04E-03 | 0.421* |
| 93582_at | Coq7 | Demethyl-Q 7 | -3.730 | 1.24E-03 | 0.521 |
| 160090_f_at | Aldoa | Aldolase 1, A isoform | -3.714 | 1.27E-03 | 0.490 |
| 94056_at | Scd1 | Stearoyl-Coenzyme A desaturase 1 | -3.650 | 1.39E-03 | 0.408 |
| 161774_f_at | Lypla1 | Lysophospholipase 1 | -3.468 | 1.74E-03 | 0.636 |
| 100540_at | Lta4h | Leukotriene A4 hydrolase | -3.422 | 1.85E-03 | 0.527 |
| 100630_f_at | Gstm5 | Glutathione S-transferase, mu 5 | -3.421 | 1.86E-03 | 0.598 |
| 95066_at | Taldo1 | Transaldolase 1 | -3.256 | 2.40E-03 | 0.466 |
| 104538_at | Ptgis | Prostaglandin I2 (prostacyclin) synthase | -3.157 | 2.80E-03 | 0.372 |
| 161889_f_at | Aldoa | Aldolase 1, A isoform | -3.195 | 2.64E-03 | 0.461 |
| 98984_f_at | Gpd2 | Glycerol phosphate dehydrogenase 2, mitochondrial | -3.192 | 2.65E-03 | 0.644 |
| 101557_at | Bckdk | Branched chain ketoacid dehydrogenase kinase | -3.158 | 2.79E-03 | 0.666 |
| 94428_at | Ilvbl | Ilvb (bacterial acetolactate synthase)-like | -3.154 | 2.81E-03 | 0.725 |
| 101482_at | Ppp1cc | Protein phosphatase 1, catalytic subunit, gamma isoform | -3.099 | 3.06E-03 | 0.676 |
| 95468_at | Egln1 | EGL nine homolog 1 (C. elegans) | -3.082 | 3.16E-03 | 0.674 |
| 104627_at | Cds2 | CDP-diacylglycerol synthase (phosphatidate cytidylyltransferase) 2 | -3.061 | 3.27E-03 | 0.752* |
| 104677_at | Man1b1 | Mannosidase, alpha, class 1B, member 1 | -3.045 | 3.35E-03 | 0.666 |
| 98440_at | Ltb4dh | Leukotriene B4 12-hydroxydehydrogenase | -3.019 | 3.48E-03 | 0.578 |
| 162262_f_at | Gyg1 | Glycogenin 1 | -3.011 | 3.52E-03 | 0.432 |
| 97934_at | Galnt1 | UDP-N-acetyl-alpha-D-galactosamine:polypeptide N-acetylgalactosaminyltransferase 1 | -2.989 | 3.66E-03 | 0.683 |
| *Transport and trafficking processes* | | |  |  |  |
| 97243_at | Slc9a3r1 | Solute carrier family 9 (sodium/hydrogen exchanger), isoform 3 regulator 1 | 4.646 | 4.80E-04 | 1.846* |
| 96919_at | Atp6v0c | ATPase, H+ transporting, V0 subunit C | 3.827 | 1.10E-03 | 1.419 |
| 92288_at | Ap1g1 | Adaptor protein complex AP-1, gamma 1 subunit | 3.248 | 2.43E-03 | 1.566 |
| 92450_at | Slc12a4 | Solute carrier family 12, member 4 | 3.085 | 3.14E-03 | 1.389 |
| 103242_at | Ap1g1 | Adaptor protein complex AP-1, gamma 1 subunit | 2.865 | 4.51E-03 | 1.305 |
| 92582_at | Slc1a5 | Solute carrier family 1 (neutral amino acid transporter), member 5 | -5.393 | 2.47E-04 | 0.520 |
| 99672_at | Clcn4-2 | Chloride channel 4-2 | -5.304 | 2.70E-04 | 0.596 |
| 92220_s_at | Bin1 | Bridging integrator 1 | -4.751 | 4.32E-04 | 0.627 |
| 93626_at | Abcg2 | ATP-binding cassette, sub-family G (WHITE), member 2 | -4.326 | 6.54E-04 | 0.602 |
| 100976_at | Ptpn9 | Protein tyrosine phosphatase, non-receptor type 9 | -4.190 | 7.53E-04 | 0.575 |
| 102670_at | Vps54 | Vacuolar protein sorting 54 (yeast) | -3.950 | 9.47E-04 | 0.723 |
| 103781_at | Stx4a | Syntaxin 4A (placental) | -3.758 | 1.19E-03 | 0.514 |
| 104409_at | Grik5 | Glutamate receptor, ionotropic, kainate 5 (gamma 2) | -3.524 | 1.61E-03 | 0.586* |
| 102835_at | Ap2a2 | Adaptor protein complex AP-2, alpha 2 subunit | -3.414 | 1.87E-03 | 0.692 |
| 160064_at | Stx7 | Syntaxin 7 | -3.278 | 2.30E-03 | 0.651 |
| 99668_at | Bin1 | Bridging integrator 1 | -3.258 | 2.39E-03 | 0.663 |
| 103031_g_at | Dnm1 | Dynamin 1 | -3.232 | 2.48E-03 | 0.581 |
| 100964_at | Vti1b | Vesicle transport through interaction with t-snares 1B homolog | -2.941 | 3.94E-03 | 0.581 |
| *Cell cycle and DNA replication* | | |  |  |  |
| 104639_i_at | Josd3 | Josephin domain containing 3 | 8.813 | 5.26E-05 | 2.587 |
| 104096_at | Orc4l | Origin recognition complex, subunit 4-like (S. Cerevisiae) | 3.961 | 9.33E-04 | 1.538 |
| 102781_at | Ccnl2 | Cyclin L2 | 3.566 | 1.54E-03 | 2.196 |
| 93023_f_at | Hist1h3f | Histone 1, h3f | 3.277 | 2.31E-03 | 1.995* |
| 98478_at | Ccng2 | Cyclin G2 | -6.227 | 1.53E-04 | 0.361 |
| 103501_at | Pura | Purine rich element binding protein A | -5.014 | 3.41E-04 | 0.459 |
| 94819_f_at | Ccni | Cyclin I | -3.727 | 1.25E-03 | 0.482 |
| 98609_at | Sept9 | Septin 9 | -3.640 | 1.41E-03 | 0.611 |
| 95100_at | Anapc5 | Anaphase-promoting complex subunit 5 | -3.394 | 1.93E-03 | 0.660 |
| 92250_s_at | Prcc | Papillary renal cell carcinoma (translocation-associated) | -3.392 | 1.94E-03 | 0.710* |
| 160127_at | Ccng1 | Cyclin G1 | -3.273 | 2.32E-03 | 0.487 |
| 94843_at | Pold4 | Polymerase (DNA-directed), delta 4 | -3.255 | 2.41E-03 | 0.694 |
| 99135_at | Cdc37 | Cell division cycle 37 homolog (S. cerevisiae) | -3.143 | 2.84E-03 | 0.665 |
| 97504_at | Ccnd2 | Cyclin D2 | -3.103 | 3.05E-03 | 0.663 |
| *Immunity and defense* | | |  |  |  |
| 92731_at | Ptx3 | Pentraxin related gene | 4.262 | 6.98E-04 | 3.420* |
| 93861_f_at | Ctse | Cathepsin E | 3.511 | 1.66E-03 | 1.653 |
| 102218_at | Il6 | Interleukin 6 | 3.029 | 3.42E-03 | 6.461* |
| 100362_f_at | Igh | Immunoglobulin heavy chain complex | 2.921 | 4.11E-03 | 1.263 |
| 93860_i_at | Ctse | Cathepsin E | 2.906 | 4.18E-03 | 1.654 |
| 103032_at | Tpst1 | Protein-tyrosine sulfotransferase 1 | -3.277 | 2.31E-03 | 0.594 |
| 95430_f_at | Spg21 | Spastic paraplegia 21 homolog (human) | -3.083 | 3.16E-03 | 0.270 |
| *Response to IFN* | | |  |  |  |
| 94224_s_at | Ifi203 | Interferon activated gene 203 | 3.849 | 1.08E-03 | 3.329 |
| 99509_s_at | Jak3 | Janus kinase 3 | 3.438 | 1.80E-03 | 1.358 |
| 94774_at | Ifi202b | Interferon activated gene 202B | -4.772 | 4.21E-04 | 0.104 |
| *Cell adhesion and migration* | | |  |  |  |
| 98152_at | Ctnnd1 | Catenin (cadherin associated protein), delta 1 | 3.711 | 1.28E-03 | 1.663 |
| 103039_at | Itga5 | Integrin alpha 5 (fibronectin receptor alpha) | 3.195 | 2.63E-03 | 2.583 |
| 103005_s_at | Cd44 | CD44 antigen | 3.051 | 3.32E-03 | 2.247 |
| 94054_at | Cttn | Cortactin | -4.337 | 6.45E-04 | 0.580* |
| 99010_at | Islr | Immunoglobulin superfamily containing leucine-rich repeat | -4.126 | 7.92E-04 | 0.369 |
| 98957_at | Jam3 | Junction adhesion molecule 3 | -3.588 | 1.50E-03 | 0.515 |
| 160312_at | Cklfsf6 | Chemokine-like factor super family 6 | -3.519 | 1.62E-03 | 0.729 |
| 92880_at | Mfge8 | Milk fat globule-EGF factor 8 protein | -3.455 | 1.77E-03 | 0.645 |
| 161183_at | Dag1 | Dystroglycan 1 | -3.373 | 2.00E-03 | 0.528 |
| 96736_at | Lpp | LIM domain containing preferred translocation partner in lipoma | -2.923 | 4.09E-03 | 0.550 |
| *RNA metabolism and processing* | | |  |  |  |
| 98624_at | Rbm38 | RNA binding motif protein 38 | 5.304 | 2.68E-04 | 1.499 |
| 96084_at | Hnrpdl | Heterogeneous nuclear ribonucleoprotein D-like | 4.756 | 4.28E-04 | 1.832 |
| 103525_at | Hnrpll | Heterogeneous nuclear ribonucleoprotein L-like | 3.353 | 2.05E-03 | 1.323 |
| 104152_at | Stk40 | Serine/threonine kinase 40 | 3.303 | 2.22E-03 | 1.345 |
| 100968_at | Cstf3 | Cleavage stimulation factor, 3' pre-RNA, subunit 3 | 3.104 | 3.04E-03 | 1.953 |
| 96188_at | Adar | Adenosine deaminase, RNA-specific | 2.963 | 3.82E-03 | 2.115 |
| 101942_at | Sfrs14 | Splicing factor, arginine/serine-rich 14 | 2.904 | 4.19E-03 | 1.333 |
| 160672_at | Clp1 | CLP1, cleavage and polyadenylation factor I subunit, homolog (S. cerevisiae) | 2.865 | 4.51E-03 | 1.429 |
| 92817_at | Imp3 | IMP3, U3 small nucleolar ribonucleoprotein, homolog (yeast) | -3.964 | 9.22E-04 | 0.604 |
| 92517_at | Rbms2 | RNA binding motif, single stranded interacting protein 2 | -3.964 | 9.20E-04 | 0.566 |
| 97293_at | Rbm10 | RNA binding motif protein 10 | -3.790 | 1.14E-03 | 0.644* |
| 100720_at | Pabpc1 | Poly A binding protein, cytoplasmic 1 | -3.171 | 2.73E-03 | 0.678 |
| 98604_at | Nnp1 | Novel nuclear protein 1 | -3.047 | 3.34E-03 | 0.594 |
| *Cell development and differentiation* | | |  |  |  |
| 102737_at | Edn1 | Endothelin 1 | 5.353 | 2.56E-04 | 7.800 |
| 99964_at | Vdr | Vitamin D receptor | 4.670 | 4.69E-04 | 2.357* |
| 160887_at | Hes1 | Hairy and enhancer of split 1 (Drosophila) | 4.530 | 5.31E-04 | 2.210* |
| 92367_at | Sil | Tal1 interrupting locus | 4.190 | 7.50E-04 | 1.460 |
| 94705_at | Eya1 | Eyes absent 1 homolog (Drosophila) | 3.957 | 9.45E-04 | 1.539 |
| 103623_at | Fbn2 | Fibrillin 2 | 3.947 | 9.59E-04 | 3.253 |
| 100127_at | Crabp2 | Cellular retinoic acid binding protein II | 3.825 | 1.10E-03 | 3.624 |
| 97729_at | Enc1 | Ectodermal-neural cortex 1 | 3.511 | 1.65E-03 | 1.404 |
| 98559_at | Smtn | Smoothelin | 3.593 | 1.49E-03 | 1.555 |
| 100778_at | Cd38 | CD38 antigen | 3.085 | 3.14E-03 | 1.365 |
| 93460_at | Acvr1 | Activin A receptor, type 1 | 2.931 | 4.01E-03 | 1.569 |
| 100024_at | Shrm | Shroom | 2.924 | 4.09E-03 | 2.724* |
| 97520_s_at | Nnat | Neuronatin | 2.870 | 4.46E-03 | 3.182 |
| 93550_at | Csrp2 | Cysteine and glycine-rich protein 2 | 2.870 | 4.46E-03 | 1.832 |
| 94878_at | Btbd1 | BTB (POZ) domain containing 1 | -6.484 | 1.37E-04 | 0.458* |
| 100876_at | Fez1 | Fasciculation and elongation protein zeta 1 (zygin I) | -4.429 | 5.83E-04 | 0.385 |
| 102752_at | Cyfip1 | Cytoplasmic FMR1 interacting protein 1 | -3.791 | 1.14E-03 | 0.485 |
| 103554_at | Adam19 | A disintegrin and metallopeptidase domain 19 (meltrin beta) | -3.666 | 1.35E-03 | 0.437* |
| 160453_at | Ryk | Receptor-like tyrosine kinase | -3.591 | 1.49E-03 | 0.505 |
| 99458_i_at | Mark2 | MAP/microtubule affinity-regulating kinase 2 | -3.578 | 1.52E-03 | 0.511 |
| 94473_at | Pttg1ip | Pituitary tumor-transforming 1 interacting protein | -3.268 | 2.34E-03 | 0.598 |
| 99183_at | Ppp3r1 | Protein phosphatase 3, regulatory subunit B, alpha isoform (calcineurin B, type I) | -3.153 | 2.82E-03 | 0.739 |
| 97930_f_at | Cd151 | CD151 antigen | -3.030 | 3.42E-03 | 0.460 |
| 95405_at | Mesdc2 | Mesoderm development candiate 2 | -2.935 | 3.97E-03 | 0.618 |
| 93810_at | Ctsd | Cathepsin D | -2.883 | 4.34E-03 | 0.675 |
| *Protein biosynthesis and ribosome organization* | | |  |  |  |
| 92855_at | Eif1 | Eukaryotic translation initiation factor 1 | 3.273 | 2.33E-03 | 1.646 |
| 93787_f_at | Mrpl18 | Mitochondrial ribosomal protein L18 | -6.489 | 1.33E-04 | 0.527* |
| 94767_at | Rps11 | Ribosomal protein S11 | -5.173 | 3.04E-04 | 0.377 |
| 161480_i_at | Rplp1 | Ribosomal protein, large, P1 | -3.961 | 9.29E-04 | 0.646 |
| 99336_at | Rps5 | Ribosomal protein S5 | -3.532 | 1.59E-03 | 0.638 |
| 161499_f_at | Rpl7l1 | Ribosomal protein L7-like 1 | -3.455 | 1.77E-03 | 0.496 |
| 96850_at | Eif4g3 | Eukaryotic translation initiation factor 4 gamma, 3 | -3.219 | 2.54E-03 | 0.479 |
| 96626_at | Tufm | Tu translation elongation factor, mitochondrial | -3.058 | 3.28E-03 | 0.520 |
| 94240_i_at | Rpl29 | Ribosomal protein L29 | -2.942 | 3.94E-03 | 0.746 |
| *Apoptosis* | | |  |  |  |
| 93922_g_at | Bat3 | HLA-B-associated transcript 3 | 4.004 | 8.79E-04 | 1.338 |
| 160142_at | Tusc2 | Tumor suppressor candidate 2 | 3.556 | 1.55E-03 | 1.282 |
| 100442_at | Tbrg4 | Transforming growth factor beta regulated gene 4 | 3.393 | 1.94E-03 | 1.483 |
| 103742_at | Unc5c | Unc-5 homolog C (C. Elegans) | 3.024 | 3.45E-03 | 1.515 |
| 99392_at | Tnfaip3 | Tumor necrosis factor, alpha-induced protein 3 | 3.001 | 3.58E-03 | 5.764 |
| 93921_at | Bat3 | HLA-B-associated transcript 3 | 2.998 | 3.59E-03 | 1.305 |
| 160962_at | Bag2 | Bcl2-associated athanogene 2 | 2.976 | 3.72E-03 | 1.729 |
| 99629_at | Ei24 | Etoposide induced 2.4 mrna | -4.355 | 6.36E-04 | 0.442 |
| 96166_at | Tex261 | Testis expressed gene 261 | -3.330 | 2.12E-03 | 0.603 |
| 93064_at | Bnip2 | BCL2/adenovirus E1B 19kDa-interacting protein 1, NIP2 | -3.318 | 2.16E-03 | 0.514 |
| 103216_f_at | Ikbkg | Inhibitor of kappab kinase gamma | -2.879 | 4.40E-03 | 0.733 |
| *Ubiquitin cycle* | |  |  |  |  |
| 102247_at | Ubr1 | Ubiquitin protein ligase E3 component n-recognin 1 | 4.789 | 4.12E-04 | 1.411 |
| 160605_s_at | Usp38 | Ubiquitin specific peptidase 38 | 3.742 | 1.22E-03 | 1.606 |
| 100509_at | Rnf19 | Ring finger protein (C3HC4 type) 19 | 3.121 | 2.94E-03 | 1.608 |
| 98906_at | Fbxo9 | F-box only protein 9 | -5.304 | 2.65E-04 | 0.568 |
| 99106_at | Cops6 | COP9 (constitutive photomorphogenic) homolog, subunit 6 (Arabidopsis thaliana) | -4.061 | 8.26E-04 | 0.529 |
| 101581_at | Ube3a | Ubiquitin protein ligase E3A | -3.495 | 1.68E-03 | 0.568 |
| 160096_at | Spop | Speckle-type POZ protein | -3.429 | 1.84E-03 | 0.657 |
| 101461_f_at | Pja1 | Praja1, RING-H2 motif containing | -3.005 | 3.56E-03 | 0.652 |
| *Electron transport and energy production* | | |  |  |  |
| 95696_at | Txnl2 | Thioredoxin-like 2 | -3.906 | 9.98E-04 | 0.507 |
| 102103_f_at | Txnl2 | Thioredoxin-like 2 | -3.617 | 1.45E-03 | 0.448 |
| 92587_at | Fdx1 | Ferredoxin 1 | -3.530 | 1.59E-03 | 0.500 |
| 97013_f_at | Cyba | Cytochrome b-245, alpha polypeptide | -3.293 | 2.25E-03 | 0.507 |
| 97922_at | Ncb5or | NADPH cytochrome B5 oxidoreductase | -3.220 | 2.54E-03 | 0.669 |
| 102000_f_at | Uqcrc2 | Ubiquinol cytochrome c reductase core protein 2 | -2.938 | 3.95E-03 | 0.471 |
| *Cytoskeleton organization and biogenesis* | | |  |  |  |
| 103574_at | Ablim1 | Actin-binding LIM protein 1 | 3.385 | 1.97E-03 | 2.238 |
| 160065_s_at | Csrp1 | Cysteine and glycine-rich protein 1 | 3.640 | 1.41E-03 | 2.588 |
| 161603_r_at | Epb4.1l4a | Erythrocyte protein band 4.1-like 4a | 2.991 | 3.63E-03 | 2.821 |
| 93716_at | Trim46 | Tripartite motif protein 46 | 2.944 | 3.92E-03 | 1.382 |
| 98454_at | Palm | Paralemmin | -4.665 | 4.76E-04 | 0.723 |
| 93898_at | Sgcb | Sarcoglycan, beta (dystrophin-associated glycoprotein) | -3.876 | 1.02E-03 | 0.609 |
| 93567_at | Pfn2 | Profilin 2 | -3.542 | 1.56E-03 | 0.564 |
| *Cell growth and proliferation* | | |  |  |  |
| 100507_at | Nov | Nephroblastoma overexpressed gene | 3.933 | 9.82E-04 | 2.901 |
| 92232_at | Socs3 | Suppressor of cytokine signaling 3 | 3.871 | 1.03E-03 | 2.111* |
| 93326_at | Tspan7 | Tetraspanin 7 | 3.317 | 2.17E-03 | 1.478 |
| 99532_at | Tob1 | Transducer of erbb-2.1 | 3.070 | 3.22E-03 | 2.204 |
| 98802_at | Ereg | Epiregulin | 3.052 | 3.31E-03 | 8.910 |
| 96920_at | Htra1 | Htra serine peptidase 1 | -6.922 | 9.84E-05 | 0.297 |
| 98587_at | Nap1l1 | Nucleosome assembly protein 1-like 1 | -4.385 | 6.18E-04 | 0.588 |
| 99457_at | Mki67 | Antigen identified by monoclonal antibody Ki 67 | -3.810 | 1.11E-03 | 0.487 |
| 160326_at | Cdv3 | Carnitine deficiency-associated gene expressed in ventricle 3 | -3.271 | 2.33E-03 | 0.709 |
| 99975_at | Prkrir | Protein-kinase, interferon-inducible double stranded RNA dependent inhibitor, repressor of (P58 repressor) | -3.259 | 2.39E-03 | 0.596* |
| 93421_at | Pftk1 | PFTAIRE protein kinase 1 | -3.168 | 2.74E-03 | 0.592 |
| 94951_at | Las1l | LAS1-like (S. cerevisiae) | -3.071 | 3.21E-03 | 0.727 |
| 104390_at | Anp32a | Acidic (leucine-rich) nuclear phosphoprotein 32 family, member A | -2.926 | 4.07E-03 | 0.691 |
| *Angiogenesis* | | |  |  |  |
| 103520_at | Vegfa | Vascular endothelial growth factor A | 5.453 | 2.36E-04 | 1.990* |
| 102738_s_at | Edn1 | Endothelin 1 | 4.643 | 4.85E-04 | 3.736 |
| 92730_at | Hbegf | Heparin-binding EGF-like growth factor | 4.047 | 8.44E-04 | 2.608 |
| 99637_at | Col15a1 | Procollagen, type XV | 3.217 | 2.55E-03 | 1.344 |
| 103671_at | Htatip2 | HIV-1 tat interactive protein 2, homolog (human) | -6.295 | 1.49E-04 | 0.425 |
| 103001_at | Vegfb | Vascular endothelial growth factor B | -4.343 | 6.41E-04 | 0.575 |
| 160484_at | Rtn4 | Reticulon 4 | -3.770 | 1.18E-03 | 0.560 |
| 161983_f_at | Rnh1 | Ribonuclease/angiogenin inhibitor 1 | -2.886 | 4.31E-03 | 0.701 |
| *Proteolysis and peptidolysis* | | |  |  |  |
| 95742_at | Psmd13 | Proteasome (prosome, macropain) 26S subunit, non-atpase, 13 | -3.065 | 3.24E-03 | 0.606 |
| *Protein folding* | | |  |  |  |
| 160395_at | Nudcd2 | Nudc domain containing 2 | -3.040 | 3.38E-03 | 0.579 |
| 94915_at | Ppib | Peptidylprolyl isomerase B | -3.361 | 2.04E-03 | 0.638* |
| 160180_at | Canx | Calnexin | -2.891 | 4.29E-03 | 0.657 |
| *Blood coagulation* | | |  |  |  |
| 95474_at | F2r | Coagulation factor II (thrombin) receptor | -2.887 | 4.31E-03 | 0.500 |
| *Protein binding* | | |  |  |  |
| 104293_at | Klhl21 | Kelch-like 21 (Drosophila) | 3.176 | 2.71E-03 | 1.826 |
| 160685_at | Klhl7 | Kelch-like 7 (Drosophila) | -2.962 | 3.82E-03 | 0.754 |
| *Muscle contraction* | | |  |  |  |
| 99942_s_at | Cnn1 | Calponin 1 | 6.273 | 1.51E-04 | 3.706* |
| *Deoxyribonuclease activity* | | |  |  |  |
| 103225_at | Dnase1l1 | Deoxyribonuclease 1-like 1 | -3.475 | 1.73E-03 | 0.654 |
| *Phospholipid scrambling* | | |  |  |  |
| 102053_at | Plscr2 | Phospholipid scramblase 2 | 3.495 | 1.68E-03 | 1.548 |
| *Unknown* | |  |  |  |  |
| 104640_f_at | 4930553M18Rik | RIKEN cDNA 4930553M18 gene | 6.298 | 1.46E-04 | 2.777 |
| 160773_at | D4Wsu132e | DNA segment, Chr 4, Wayne State University 132, expressed | 3.630 | 1.44E-03 | 1.489 |
| 96156_at | 4930418G15Rik | RIKEN cDNA 4930418G15 gene | 3.253 | 2.41E-03 | 2.014 |
| 93568_i_at | B930046C15Rik | RIKEN cDNA B930046C15 gene | 3.250 | 2.42E-03 | 1.806 |
| 103316_at | Camsap1 | Calmodulin regulated spectrin-associated protein 1 | 3.089 | 3.10E-03 | 1.355 |
| 97933_at | 2010111I01Rik | RIKEN cDNA 2010111I01 gene | 3.095 | 3.08E-03 | 1.518 |
| 95641_at | Plac9 | Placenta specific 9 | 3.079 | 3.18E-03 | 1.294 |
| 95052_at | 1110035L05Rik | RIKEN cDNA 1110035L05 gene | -8.310 | 6.18E-05 | 0.322 |
| 93059_at | 2610204K14Rik | RIKEN cDNA 2610204K14 gene | -6.972 | 9.15E-05 | 0.352 |
| 160963_at | 9630050M13Rik | RIKEN cDNA 9630050M13 gene | -5.039 | 3.34E-04 | 0.429 |
| 97423_at | 1500035H01Rik | RIKEN cDNA 1500035H01 gene | -4.851 | 3.87E-04 | 0.586 |
| 99366_at | Pqlc3 | PQ loop repeat containing | -4.659 | 4.78E-04 | 0.507 |
| 104260_at | AW112037 | Expressed sequence AW112037 | -4.324 | 6.57E-04 | 0.545 |
| 160772_i_at | D11Ertd730e | DNA segment, Chr 11, ERATO Doi 730, expressed | -4.324 | 6.59E-04 | 0.487 |
| 95028_r_at | NA | NA | -4.264 | 6.96E-04 | 0.641 |
| 92884_at | 9130221H12Rik | RIKEN cDNA 9130221H12 gene | -4.251 | 7.07E-04 | 0.484 |
| 95411_at | 1110004F10Rik | RIKEN cDNA 1110004F10 gene | -4.213 | 7.30E-04 | 0.546 |
| 162080_f_at | NA | NA | -4.116 | 7.96E-04 | 0.713 |
| 95045_at | 0610012D09Rik | RIKEN cDNA 0610012D09 gene | -4.059 | 8.31E-04 | 0.522 |
| 104183_at | D2Ertd435e | DNA segment, Chr 2, ERATO Doi 435, expressed | -4.001 | 8.81E-04 | 0.638 |
| 96104_at | 3732413I11Rik | RIKEN cDNA 3732413I11 gene | -3.836 | 1.09E-03 | 0.427 |
| 94426_at | Thumpd1 | THUMP domain containing 1 | -3.763 | 1.19E-03 | 0.668 |
| 104038_at | 4933434E20Rik | RIKEN cDNA 4933434E20 gene | -3.744 | 1.22E-03 | 0.667 |
| 96320_at | 2310044H10Rik | RIKEN cDNA 2310044H10 gene | -3.720 | 1.26E-03 | 0.457 |
| 96917_at | 2410166I05Rik | RIKEN cDNA 2410166I05 gene | -3.717 | 1.26E-03 | 0.761 |
| 96340_at | Tmem50b | Transmembrane protein 50B | -3.503 | 1.67E-03 | 0.679 |
| 101960_at | D10Wsu52e | DNA segment, Chr 10, Wayne State University 52, expressed | -3.389 | 1.95E-03 | 0.583 |
| 160904_at | B230317C12Rik | RIKEN cDNA B230317C12 gene | -3.377 | 1.99E-03 | 0.642 |
| 94022_at | D1Mgi9 | DNA Segment, Chr 1, Mouse Genome Informatics 9 | -3.365 | 2.02E-03 | 0.564 |
| 96090_g_at | 4931406C07Rik | RIKEN cDNA 4931406C07 gene | -3.349 | 2.07E-03 | 0.584 |
| 99139_at | 2310075A12Rik | RIKEN cDNA 2310075A12 gene | -3.332 | 2.12E-03 | 0.720 |
| 103221_at | 5031425D22Rik | RIKEN cDNA 5031425D22 gene | -3.322 | 2.16E-03 | 0.667 |
| 95984_at | C79468 | expressed sequence C79468 | -3.312 | 2.18E-03 | 0.742 |
| 96902_at | 2900091E11Rik | RIKEN cDNA 2900091E11 gene | -3.298 | 2.23E-03 | 0.714 |
| 97797_at | 2210409M21Rik | RIKEN cDNA 2210409M21 gene | -3.291 | 2.25E-03 | 0.695 |
| 98524_f_at | 2210039B01Rik | RIKEN cDNA 2210039B01 gene | -3.253 | 2.42E-03 | 0.648 |
| 95702_at | 1300006C19Rik | RIKEN cDNA 1300006C19 gene | -3.225 | 2.51E-03 | 0.671 |
| 161437_f_at | NA | NA | -3.192 | 2.66E-03 | 0.667 |
| 96943_at | NA | NA | -3.175 | 2.72E-03 | 0.641 |
| 102141_f_at | NA | NA | -3.117 | 2.97E-03 | 0.615 |
| 100587_f_at | 5730403B10Rik | RIKEN cDNA 5730403B10 gene | -3.094 | 3.08E-03 | 0.715 |
| 104252_at | AU020206 | expressed sequence AU020206 | -3.092 | 3.10E-03 | 0.765 |
| 96613_at | 5730536A07Rik | RIKEN cDNA 5730536A07 gene | -3.038 | 3.40E-03 | 0.540 |
| 161214_r_at | BC037034 | cDNA sequence BC037034 | -3.024 | 3.45E-03 | 0.684 |
| 104366_at | BC039093 | cDNA sequence BC039093 | -3.016 | 3.50E-03 | 0.497 |
| 95759_at | 2900092E17Rik | RIKEN cDNA 2900092E17 gene | -2.945 | 3.91E-03 | 0.715 |
| 93986_at | 2410003A14Rik | RIKEN cDNA 2410003A14 gene | -2.933 | 4.00E-03 | 0.574 |
| 98953_at | 1500010M16Rik | RIKEN cDNA 1500010M16 gene | -2.913 | 4.15E-03 | 0.591 |
| 104074_at | 0610025L06Rik | RIKEN cDNA 0610025L06 gene | -2.885 | 4.32E-03 | 0.761 |
| 94923_f_at | D8Ertd69e | DNA segment, Chr 8, ERATO Doi 69, expressed | -2.880 | 4.39E-03 | 0.603 |

**Table S5. Differential gene expression in serum-starved, G0-arrested N-*ras*^-/-^ fibroblasts after incubation of cell cultures in the presence of serum for 1 hour.** List of 696 differentially expressed probesets (649 different genes) identified by means of SAM contrast (FDR=0,09) comparing the microarray-generated transcriptional profile of WT fibroblasts that had been serum-starved for 24h to that of similarly starved, N-*ras***^-/-^** fibroblast cultures submitted to subsequent incubation in the presence of 20% FBS for 1 hour. To concentrate on loci exclusively regulated by Ras, genes sharing similar values of differential expression between the WT and N-*ras*^-/-^ fibroblast (ratio of the R-fold values in their respective lists within the range 0.6-1.5) were excluded from this list. The differentially expressed loci are identified by Affymetrix probeset ID, gene name and symbol, and listed according to functional category and degree of overexpression or repression, quantitated by d(i) value. The * and # symbols in the R.fold column denote independent validation of the transcriptional data obtained by means of quantitative RT-PCR or Western immunoblot, respectively. d(i) is a parameter measuring the statistical distance separating the calculated expression value of each gene probeset from the null hypothesis (no-change). p-value is an statistical measure indicating the probability of random expression for that probeset. R fold is the log2 value of the fold change measuring the overexpression or repression of the probesets in the collection of microarrays.

| **Probeset ID** | **Gene symbol** | **Gene name** | **d(i)** | **p-value** | **R.fold** |
| --- | --- | --- | --- | --- | --- |
| *Signal transduction* | | |  |  |  |
| 94516_f_at | Penk1 | Preproenkephalin 1 | 27.659 | 1.37E-05 | 15.295 |
| 160353_i_at | Mapkapk2 | MAP kinase-activated protein kinase 2 | 16.477 | 4.58E-05 | 2.975 |
| 101561_at | Mt2 | Metallothionein 2 | 12.923 | 8.69E-05 | 6.638* |
| 93974_at | Errfi1 | ERBB receptor feedback inhibitor 1 | 8.747 | 2.72E-04 | 16.374 |
| 95721_at | Mapkapk2 | MAP kinase-activated protein kinase 2 | 7.793 | 4.07E-04 | 1.842 |
| 92362_at | Dusp8 | Dual specificity phosphatase 8 | 7.240 | 5.29E-04 | 2.437 |
| 160747_at | Rgs3 | Regulator of G-protein signaling 3 | 7.051 | 5.95E-04 | 1.923 |
| 93573_at | Mt1 | Metallothionein 1 | 6.654 | 6.96E-04 | 5.765 |
| 94378_at | Rgs16 | Regulator of G-protein signaling 16 | 6.155 | 9.38E-04 | 3.541 |
| 100958_at | Spata13 | Spermatogenesis associated 13 | 6.009 | 1.03E-03 | 1.602 |
| 97106_at | Map3k8 | Mitogen activated protein kinase kinase kinase 8 | 5.869 | 1.13E-03 | 2.193* |
| 101846_r_at | Csprs | Component of Sp100-rs, predicted gene | 5.718 | 1.24E-03 | 1.538 |
| 101435_at | Akap2 | A kinase (PRKA) anchor protein 2 | 5.681 | 1.27E-03 | 1.996 |
| 97962_at | Synj2 | Synaptojanin 2 | 5.528 | 1.40E-03 | 1.372 |
| 93975_at | Errfi1 | ERBB receptor feedback inhibitor 1 | 5.433 | 1.52E-03 | 10.764 |
| 100555_at | Rcan | Regulator of calcineurin 1 | 5.406 | 1.55E-03 | 4.044 |
| 93314_g_at | Map2k3 | Mitogen activated protein kinase kinase 3 | 5.228 | 1.79E-03 | 1.637* |
| 161609_at | Rgs16 | Regulator of G-protein signaling 16 | 5.118 | 1.94E-03 | 2.671 |
| 102663_at | Plaur | Urokinase plasminogen activator receptor | 5.059 | 2.05E-03 | 2.849 |
| 98817_at | Fst | Follistatin | 4.992 | 2.17E-03 | 2.707 |
| 93315_at | Map2k3 | Mitogen activated protein kinase kinase 3 | 4.948 | 2.25E-03 | 2.429* |
| 103098_at | Baiap2 | Brain-specific angiogenesis inhibitor 1-associated protein 2 | 4.367 | 3.79E-03 | 1.613 |
| 94929_at | Ptpn1 | Protein tyrosine phosphatase, non-receptor type 1 | 4.207 | 4.42E-03 | 1.598 |
| 160699_at | Cdca5 | Cell division cycle associated 5 | 4.176 | 4.55E-03 | 1.284 |
| 104180_at | Rac3 | RAS-related C3 botulinum substrate 3 | 4.161 | 4.62E-03 | 1.442 |
| 92205_at | Irs2 | Insulin receptor substrate 2 | 4.159 | 4.63E-03 | 1.494 |
| 97319_at | Rrad | Ras-related associated with diabetes | 4.056 | 5.20E-03 | 1.963* |
| 99960_at | Map2k4 | Mitogen activated protein kinase kinase 4 | 3.922 | 6.07E-03 | 1.802 |
| 161088_r_at | Akap8 | A kinase (PRKA) anchor protein 8 | 3.745 | 7.46E-03 | 1.202 |
| 104416_at | Flt4 | FMS-like tyrosine kinase 4 | 3.693 | 7.90E-03 | 1.193 |
| 101845_s_at | Csprs | Component of Sp100-rs, predicted gene | 3.682 | 8.00E-03 | 2.548 |
| 100883_at | Arfl4 | ADP-ribosylation factor 4-like | 3.663 | 8.18E-03 | 1.323 |
| 94362_at | **Nras** | Neuroblastoma ras oncogene | -12.910 | 8.92E-05 | 0.139 |
| 102759_at | Pik3r2 | Phosphatidylinositol 3-kinase, regulatory subunit, polypeptide 2 (p85 beta) | -11.138 | 1.33E-04 | 0.541* |
| 102374_at | Rcan3 | Regulator of calcineurin 3 | -7.852 | 3.89E-04 | 0.514 |
| 95136_at | Arl6ip4 | ADP-ribosylation factor-like 6 interacting protein 4 | -7.849 | 3.91E-04 | 0.608 |
| 160925_at | **Nras** | Neuroblastoma ras oncogene | -7.370 | 4.85E-04 | 0.398 |
| 93826_at | Ppp2r5a | Protein phosphatase 2, regulatory subunit B (B56), alpha isoform | -7.351 | 4.90E-04 | 0.262* |
| 103592_at | Map2k5 | Mitogen activated protein kinase kinase 5 | -7.175 | 5.58E-04 | 0.630 |
| 160188_at | Nudt4 | Nudix (nucleoside diphosphate linked moiety X)-type motif 4 | -7.100 | 5.81E-04 | 0.350 |
| 96728_at | Wdr45 | WD repeat domain 45 | -6.668 | 6.93E-04 | 0.515 |
| 102308_at | Tulp3 | Tubby-like protein 3 | -6.473 | 7.62E-04 | 0.683 |
| 160189_at | Nudt4 | Nudix (nucleoside diphosphate linked moiety X)-type motif 4 | -6.467 | 7.66E-04 | 0.523 |
| 93962_at | Rap1a | RAS-related protein-1a | -6.445 | 7.85E-04 | 0.693 |
| 160965_at | Rasa4 | RAS p21 protein activator 4 | -6.301 | 8.51E-04 | 0.331 |
| 99471_at | Stard7 | START domain containing 7 | -5.912 | 1.10E-03 | 0.763* |
| 162011_f_at | Rhou | Ras homolog gene family, member U | -5.738 | 1.22E-03 | 0.554 |
| 104461_at | Pik3ca | Phosphatidylinositol 3-kinase, catalytic, alpha polypeptide | -5.368 | 1.59E-03 | 0.616 |
| 93748_at | Grinl1a | Glutamate receptor, ionotropic, N-methyl D-aspartate-like 1A | -5.362 | 1.61E-03 | 0.710 |
| 100684_at | Prkcsh | Protein kinase C substrate 80K-H | -5.255 | 1.76E-03 | 0.694 |
| 104302_f_at | Commd9 | COMM domain containing 9 | -5.246 | 1.76E-03 | 0.729 |
| 94801_at | Pgrmc2 | Progesterone receptor membrane component 2 | -5.193 | 1.85E-03 | 0.629 |
| 160938_at | Mast2 | Microtubule associated serine/threonine kinase 2 | -5.071 | 2.03E-03 | 0.581 |
| 97226_at | Gna12 | Guanine nucleotide binding protein, alpha 12 | -5.009 | 2.13E-03 | 0.425 |
| 96747_at | Rhou | Ras homolog gene family, member U | -4.954 | 2.23E-03 | 0.342 |
| 95022_at | Akap12 | A kinase (PRKA) anchor protein (gravin) 12 | -4.943 | 2.26E-03 | 0.190 |
| 98005_at | Pkia | Protein kinase inhibitor, alpha | -4.917 | 2.30E-03 | 0.427 |
| 160690_at | Csnk2a1 | Casein kinase 2, alpha 1 polypeptide | -4.830 | 2.46E-03 | 0.672 |
| 94403_at | Tbc1d14 | TBC1 domain family, member 14 | -4.823 | 2.47E-03 | 0.648 |
| 100122_at | Gnb5 | Guanine nucleotide binding protein, beta 5 | -4.748 | 2.64E-03 | 0.654 |
| 97058_f_at | Rab33b | RAB33B, member of RAS oncogene family | -4.720 | 2.70E-03 | 0.755 |
| 99596_f_at | Gnai2 | Guanine nucleotide binding protein, alpha inhibiting 2 | -4.658 | 2.88E-03 | 0.635 |
| 98524_f_at | Mtm1 | X-linked myotubular myopathy gene 1 | -4.490 | 3.34E-03 | 0.643 |
| 98503_at | Evi5 | Ecotropic viral integration site 5 | -4.449 | 3.50E-03 | 0.446 |
| 160480_at | Ptprs | Protein tyrosine phosphatase, receptor type, S | -4.330 | 3.93E-03 | 0.703 |
| 103406_at | Xab1 | XPA binding protein 1 | -4.327 | 3.95E-03 | 0.706 |
| 102196_at | Gna11 | Guanine nucleotide binding protein, alpha 11 | -4.258 | 4.22E-03 | 0.543 |
| 103402_at | Tm7sf3 | Transmembrane 7 superfamily member 3 | -4.251 | 4.25E-03 | 0.589 |
| 101113_at | Rhoa | Ras homolog gene family, member A | -4.236 | 4.32E-03 | 0.607 |
| 160373_i_at | Sdpr | Serum deprivation response | -4.177 | 4.54E-03 | 0.490* |
| 160490_at | Def8 | Differentially expressed in FDCP 8 | -4.106 | 4.91E-03 | 0.731 |
| 100908_at | Ptpra | Protein tyrosine phosphatase, receptor type, A | -4.099 | 4.94E-03 | 0.507 |
| 101555_at | Rac1 | RAS-related C3 botulinum substrate 1 | -4.000 | 5.58E-03 | 0.775 |
| 94359_at | Plekha1 | Pleckstrin homology domain containing, family A (phosphoinositide binding specific) member 1 | -3.918 | 6.08E-03 | 0.494 |
| 101505_at | Tbc1d20 | TBC1 domain family, member 20 | -3.879 | 6.31E-03 | 0.711 |
| 160632_at | Prkcn | Protein kinase C, nu | -3.847 | 6.57E-03 | 0.455 |
| 97505_at | Arl1 | ADP-ribosylation factor-like 1 | -3.768 | 7.24E-03 | 0.585 |
| 94398_s_at | Inpp5b | Inositol polyphosphate-5-phosphatase B | -3.757 | 7.36E-03 | 0.739 |
| 160873_at | Ghrl | Ghrelin | -3.755 | 7.38E-03 | 0.614 |
| 104080_at | Pdap1 | PDGFA associated protein 1 | -3.730 | 7.56E-03 | 0.723 |
| 104108_at | Rab6ip1 | Rab6 interacting protein 1 | -3.730 | 7.58E-03 | 0.701 |
| 99978_s_at | Mapk14 | Mitogen activated protein kinase 14 | -3.710 | 7.77E-03 | 0.498 |
| 95454_at | S100a1 | S100 calcium binding protein A1 | -3.699 | 7.85E-03 | 0.737 |
| 100635_at | Sar1a | SAR1a gene homolog 1 (S. cerevisiae) | -3.697 | 7.87E-03 | 0.699 |
| 102014_at | Homer3 | Homer homolog 3 (Drosophila) | -3.688 | 7.95E-03 | 0.708 |
| 98927_at | Rab6 | RAB6, member RAS oncogene family | -3.655 | 8.26E-03 | 0.732 |
| 97958_at | Prkcbp1 | Protein kinase C binding protein 1 | -3.637 | 8.43E-03 | 0.488 |
| 104053_at | Trio | Triple functional domain (PTPRF interacting) | -3.611 | 8.71E-03 | 0.559 |
| 103840_at | Rad17 | RAD17 homolog (S. pombe) | -3.601 | 8.82E-03 | 0.757 |
| 93464_at | Akap9 | A kinase (PRKA) anchor protein (yotiao) 9 | -3.601 | 8.81E-03 | 0.673 |
| 96751_at | Arl5 | ADP-ribosylation factor-like 5 | -3.588 | 8.97E-03 | 0.489 |
| 102117_at | Rabl4 | RAB, member of RAS oncogene family-like 4 | -3.569 | 9.15E-03 | 0.650 |
| 93116_at | Prkacb | Protein kinase, cAMP dependent, catalytic, beta | -3.556 | 9.30E-03 | 0.589 |
| 93747_at | Ptplad1 | Protein tyrosine phosphatase-like A domain containing 1 | -3.553 | 9.34E-03 | 0.489 |
| 94853_at | Gnb1 | Guanine nucleotide binding protein, beta 1 | -3.513 | 9.79E-03 | 0.683* |
| *Transcription* | | |  |  |  |
| 99602_at | Klf10 | Kruppel-like factor 10 | 19.751 | 2.75E-05 | 7.036 |
| 101583_at | Btg2 | B-cell translocation gene 2, anti-proliferative | 15.209 | 5.49E-05 | 19.456 |
| 99835_at | Fosl1 | Fos-like antigen 1 | 11.824 | 1.17E-04 | 13.396* |
| 99535_at | Ccrn4l | CCR4 carbon catabolite repression 4-like (S. cerevisiae) | 11.065 | 1.35E-04 | 15.261* |
| 97813_at | Rela | V-rel reticuloendotheliosis viral oncogene homolog A (avian) | 9.695 | 1.92E-04 | 2.168* |
| 102069_at | Mtf2 | Metal response element binding transcription factor 2 | 8.941 | 2.49E-04 | 1.434 |
| 94187_at | Gsc | Goosecoid | 7.388 | 4.78E-04 | 1.531 |
| 104701_at | Bhlhb2 | Basic helix-loop-helix domain containing, class B2 | 7.217 | 5.40E-04 | 5.145* |
| 160834_at | Sertad1 | SERTA domain containing 1 | 6.831 | 6.45E-04 | 3.803 |
| 104152_at | Stk40 | Serine/threonine kinase 40 | 6.386 | 8.14E-04 | 1.329 |
| 162384_f_at | Ccrn4l | CCR4 carbon catabolite repression 4-like (S. cerevisiae) | 5.349 | 1.63E-03 | 4.461* |
| 98447_at | Cebpa | CCAAT/enhancer binding protein (C/EBP), alpha | 5.173 | 1.87E-03 | 1.386 |
| 98559_at | Smtn | Smoothelin | 5.125 | 1.93E-03 | 2.001 |
| 97704_at | Ell2 | Elongation factor RNA polymerase II 2 | 5.088 | 1.99E-03 | 2.245* |
| 93943_f_at | Zfp36l2 | Zinc finger protein 36, C3H type-like 2 | 4.927 | 2.28E-03 | 2.486 |
| 92249_g_at | Nr4a2 | Nuclear receptor subfamily 4, group A, member 2 | 4.704 | 2.75E-03 | 2.329* |
| 92248_at | Nr4a2 | Nuclear receptor subfamily 4, group A, member 2 | 4.703 | 2.75E-03 | 2.317* |
| 98534_at | Sap18 | Sin3-associated polypeptide 18 | 4.702 | 2.75E-03 | 1.373 |
| 160894_at | Cebpd | CCAAT/enhancer binding protein (C/EBP), delta | 4.444 | 3.53E-03 | 3.475 |
| 103546_at | Fosl2 | Fos-like antigen 2 | 4.332 | 3.91E-03 | 1.720* |
| 103757_at | Mizf | MBD2-interacting zinc finger | 4.322 | 3.96E-03 | 1.198 |
| 102996_at | Ell | Elongation factor RNA polymerase II | 4.241 | 4.29E-03 | 1.269 |
| 101694_f_at | Myst2 | MYST histone acetyltransferase 2 | 4.019 | 5.47E-03 | 1.284 |
| 92300_at | Mnt | Max binding protein | 3.890 | 6.25E-03 | 1.362 |
| 98030_at | Trim30 | Tripartite motif protein 30 | 3.855 | 6.50E-03 | 4.845 |
| 102657_at | Hlx1 | H2.0-like homeo box 1 (Drosophila) | 3.840 | 6.62E-03 | 1.307 |
| 92925_at | Cebpb | CCAAT/enhancer binding protein (C/EBP), beta | 3.831 | 6.68E-03 | 3.347* |
| 93878_at | Mllt10 | Myeloid/lymphoid or mixed lineage-leukemia translocation to 10 homolog (Drosophila) | 3.825 | 6.74E-03 | 1.223 |
| 101502_at | Tgif | TG interacting factor | 3.786 | 7.07E-03 | 2.158* |
| 92562_at | Nfe2l2 | Nuclear factor, erythroid derived 2, like 2 | 3.758 | 7.36E-03 | 2.078 |
| 92251_f_at | AI607873 | Expressed sequence AI607873 | 3.655 | 8.25E-03 | 2.811 |
| 93490_at | Maf1 | MAF1 homolog (S. cerevisiae) | -9.264 | 2.31E-04 | 0.471 |
| 161308_f_at | Yap1 | Yes-associated protein 1 | -9.174 | 2.36E-04 | 0.663 |
| 99024_at | Mxd4 | Max dimerization protein 4 | -8.983 | 2.45E-04 | 0.331 |
| 93693_at | Hmbox1 | Homeobox containing 1 | -7.656 | 4.26E-04 | 0.458 |
| 103092_at | Trim37 | Tripartite motif protein 37 | -7.156 | 5.65E-04 | 0.562 |
| 101081_at | Ctbp1 | C-terminal binding protein 1 | -7.125 | 5.74E-04 | 0.483 |
| 160449_at | Dr1 | Down-regulator of transcription 1 | -6.072 | 9.77E-04 | 0.623* |
| 102048_at | Ankrd1 | Ankyrin repeat domain 1 (cardiac muscle) | -5.933 | 1.09E-03 | 0.095* |
| 104279_at | Polr2f | Polymerase (RNA) II (DNA directed) polypeptide F | -5.866 | 1.14E-03 | 0.725 |
| 94015_at | Th1l | TH1-like homolog (Drosophila) | -5.580 | 1.36E-03 | 0.755 |
| 94295_at | Gtf2i | General transcription factor II I | -5.563 | 1.38E-03 | 0.426 |
| 100941_at | Pias2 | Protein inhibitor of activated STAT2 | -5.550 | 1.39E-03 | 0.770 |
| 103762_at | Gtf2f1 | General transcription factor IIF, polypeptide 1 | -5.483 | 1.46E-03 | 0.623 |
| 103081_at | Baz1b | Bromodomain adjacent to zinc finger domain, 1B | -5.457 | 1.48E-03 | 0.725 |
| 102833_at | Cbx2 | Chromobox homolog 2 (Drosophila Pc class) | -5.400 | 1.56E-03 | 0.706 |
| 100461_at | Polr2j | Polymerase (RNA) II (DNA directed) polypeptide J | -5.334 | 1.65E-03 | 0.603 |
| 92934_at | Zfp90 | Zinc finger protein 90 | -5.129 | 1.93E-03 | 0.592 |
| 95883_at | Phf17 | PHD finger protein 17 | -5.032 | 2.09E-03 | 0.553 |
| 93071_at | Trim28 | Tripartite motif protein 28 | -4.885 | 2.35E-03 | 0.698* |
| 95522_i_at | Zfp68 | Zinc finger protein 68 | -4.816 | 2.48E-03 | 0.421* |
| 98982_at | Tmpo | Thymopoietin | -4.733 | 2.68E-03 | 0.791 |
| 98981_s_at | Tcf12 | Transcription factor 12 | -4.724 | 2.69E-03 | 0.370 |
| 95161_at | Ctdsp2 | CTD (carboxy-terminal domain, RNA polymerase II, polypeptide A) small phosphatase 2 | -4.585 | 3.08E-03 | 0.473 |
| 102235_at | Mycl1 | v-myc myelocytomatosis viral oncogene homolog 1, lung carcinoma derived (avian) | -4.569 | 3.12E-03 | 0.652 |
| 92375_at | Ascc1 | Activating signal cointegrator 1 complex subunit 1 | -4.512 | 3.29E-03 | 0.712 |
| 160109_at | Sox4 | SRY-box containing gene 4 | -4.434 | 3.56E-03 | 0.436 |
| 160245_at | Zfp740 | Zinc finger protein 740 | -4.397 | 3.69E-03 | 0.572 |
| 98767_at | Yy1 | YY1 transcription factor | -4.313 | 4.02E-03 | 0.504 |
| 104462_at | Hic1 | Hypermethylated in cancer 1 | -4.164 | 4.60E-03 | 0.604 |
| 160138_at | Mxi1 | Max interacting protein 1 | -4.161 | 4.62E-03 | 0.434 |
| 96940_at | Tead2 | TEA domain family member 2 | -4.148 | 4.72E-03 | 0.604 |
| 104554_at | Nr2f6 | Nuclear receptor subfamily 2, group F, member 6 | -4.108 | 4.90E-03 | 0.686 |
| 104437_at | Zfp30 | Zinc finger protein 30 | -4.105 | 4.91E-03 | 0.453 |
| 160129_at | Eef1d | Eukaryotic translation elongation factor 1 delta (guanine nucleotide 27olester protein) | -4.086 | 5.03E-03 | 0.742 |
| 160483_at | Tcf4 | Transcription factor 4 | -4.078 | 5.08E-03 | 0.434 |
| 99950_at | Tbp | TATA box binding protein | -4.004 | 5.56E-03 | 0.737 |
| 94973_at | Jmjd1b | Jumonji domain containing 1B | -4.000 | 5.58E-03 | 0.641 |
| 161077_f_at | Smarcd2 | SWI/SNF related, matrix associated, actin dependent regulator of chromatin, subfamily d, member 2 | -3.949 | 5.90E-03 | 0.574 |
| 94396_at | Ing1 | Inhibitor of growth family, member 1 | -3.930 | 6.02E-03 | 0.617 |
| 161741_r_at | Zfp503 | Zinc finger protein 503 | -3.886 | 6.28E-03 | 0.788 |
| 104471_at | Hdac6 | Histone deacetylase 6 | -3.839 | 6.63E-03 | 0.818* |
| 94821_at | Xbp1 | X-box binding protein 1 | -3.768 | 7.22E-03 | 0.556 |
| 102271_at | Zmiz2 | Zinc finger, MIZ-type containing 2 | -3.739 | 7.50E-03 | 0.801 |
| 104453_at | Sap30l | Similar to SAP30-like | -3.699 | 7.85E-03 | 0.622 |
| 162138_s_at | Cbx6 | Chromobox homolog 6 | -3.677 | 8.06E-03 | 0.351 |
| 160848_at | Zhx1 | Zinc fingers and homeoboxes protein 1 | -3.534 | 9.57E-03 | 0.487 |
| *Primary cell metabolism* | | |  |  |  |
| 104509_at | Ch25h | Cholesterol 25-hydroxylase | 7.055 | 5.90E-04 | 3.173* |
| 104647_at | Ptgs2 | Prostaglandin-endoperoxide synthase 2 | 6.534 | 7.39E-04 | 27.027* |
| 160832_at | Ldlr | Low density lipoprotein receptor | 5.967 | 1.06E-03 | 2.150* |
| 99425_at | Hmgcr | 3-hydroxy-3-methylglutaryl-Coenzyme A reductase | 5.743 | 1.21E-03 | 4.587 |
| 95392_at | Gcnt2 | Glucosaminyl (N-acetyl) transferase 2, I-branching enzyme | 5.727 | 1.23E-03 | 1.886* |
| 94461_at | Pbef1 | Pre-B-cell colony-enhancing factor 1 | 5.200 | 1.84E-03 | 2.521 |
| 99820_f_at | Cecr5 | Cat eye syndrome chromosome region, candidate 5 homolog (human) | 4.907 | 2.32E-03 | 1.211 |
| 104285_at | Hmgcr | 3-hydroxy-3-methylglutaryl-Coenzyme A reductase | 4.736 | 2.67E-03 | 2.863 |
| 94177_at | Hsd17b7 | Hydroxysteroid (17-beta) dehydrogenase 7 | 4.586 | 3.08E-03 | 2.262 |
| 161629_i_at | Afp | Alpha fetoprotein | 4.554 | 3.17E-03 | 1.802* |
| 93868_at | Nsdhl | NAD(P) dependent steroid dehydrogenase-like | 4.356 | 3.82E-03 | 2.151 |
| 103066_at | Tyki | Thymidylate kinase family LPS-inducible member | 4.273 | 4.17E-03 | 2.965 |
| 98631_g_at | Nsdhl | NAD(P) dependent steroid dehydrogenase-like | 4.235 | 4.33E-03 | 2.312* |
| 104519_at | Itih2 | Inter-alpha trypsin inhibitor, heavy chain 2 | 4.172 | 4.56E-03 | 2.103 |
| 94367_at | Uck2 | Uridine-cytidine kinase 2 | 4.086 | 5.03E-03 | 1.652 |
| 94325_at | Hmgcs1 | 3-hydroxy-3-methylglutaryl-Coenzyme A synthase 1 | 4.073 | 5.12E-03 | 2.776 |
| 103914_at | Pcyt2 | Phosphate cytidylyltransferase 2, ethanolamine | 4.024 | 5.44E-03 | 1.586 |
| 95420_at | Pgd | Phosphogluconate dehydrogenase | 4.012 | 5.50E-03 | 1.355 |
| 160289_s_at | Dlst | Dihydrolipoamide S-succinyltransferase (E2 component of 2-oxo-glutarate complex) | 3.864 | 6.44E-03 | 1.425 |
| 96533_at | Tor3a | Torsin family 3, member A | 3.853 | 6.52E-03 | 2.026 |
| 95978_at | Atp13a3 | ATPase type 13A3 | 3.813 | 6.83E-03 | 1.539 |
| 101587_at | Ephx1 | Epoxide hydrolase 1, microsomal | 3.800 | 6.94E-03 | 1.932* |
| 101173_at | Pctp | Phosphatidylcholine transfer protein | 3.763 | 7.29E-03 | 1.180 |
| 160137_at | B3gnt1 | UDP-GlcNAc:betaGal beta-1,3-N-acetylglucosaminyltransferase 1 | 3.716 | 7.72E-03 | 1.300 |
| 104372_at | Abhd8 | Abhydrolase domain containing 8 | -10.189 | 1.72E-04 | 0.435 |
| 104677_at | Man1b1 | Mannosidase, alpha, class 1B, member 1 | -8.097 | 3.57E-04 | 0.508 |
| 161243_f_at | Abhd8 | Abhydrolase domain containing 8 | -6.240 | 8.76E-04 | 0.567 |
| 102993_at | Ggta1 | Glycoprotein galactosyltransferase alpha 1, 3 | -5.852 | 1.15E-03 | 0.550 |
| 103739_at | Glce | Glucuronyl C5-epimerase | -5.719 | 1.24E-03 | 0.409 |
| 92557_at | Hsd17b1 | Hydroxysteroid (17-beta) dehydrogenase 1 | -5.631 | 1.31E-03 | 0.804 |
| 101482_at | Ppp1cc | Protein phosphatase 1, catalytic subunit, gamma isoform | -5.523 | 1.41E-03 | 0.504 |
| 97797_at | Asnsd1 | Asparagine synthetase domain containing 1 | -5.508 | 1.42E-03 | 0.683 |
| 100576_at | Pafah1b3 | Platelet-activating factor acetylhydrolase, isoform 1b, alpha1 subunit | -5.439 | 1.50E-03 | 0.533 |
| 94312_at | Pdxdc1 | Pyridoxal-dependent decarboxylase domain containing 1 | -5.211 | 1.82E-03 | 0.646 |
| 96948_at | Qdpr | Quininoid dihydropteridine reductase | -4.997 | 2.16E-03 | 0.654 |
| 103646_at | Crat | Carnitine acetyltransferase | -4.926 | 2.29E-03 | 0.682 |
| 98960_s_at | B3galt3 | UDP-Gal:betaGlcNAc beta 1,3-galactosyltransferase, polypeptide 3 | -4.841 | 2.44E-03 | 0.712 |
| 101000_at | Oaz2 | Ornithine decarboxylase antizyme 2 | -4.798 | 2.53E-03 | 0.501 |
| 93320_at | Cpt1a | Carnitine palmitoyltransferase 1a, liver | -4.747 | 2.64E-03 | 0.451 |
| 97398_at | Pck2 | Phosphoenolpyruvate carboxykinase 2 (mitochondrial) | -4.478 | 3.39E-03 | 0.672 |
| 160195_at | 1200013P24Rik | RIKEN cDNA 1200013P24 gene | -4.395 | 3.70E-03 | 0.661 |
| 94818_at | Ogt | O-linked N-acetylglucosamine (GlcNAc) transferase (UDP-N-acetylglucosamine:polypeptide-N-acetylglucosaminyl transferase) | -4.349 | 3.84E-03 | 0.325* |
| 161349_f_at | Dpm2 | Dolichol-phosphate (beta-D) mannosyltransferase 2 | -4.348 | 3.86E-03 | 0.685 |
| 95754_at | Mbtps1 | Membrane-bound transcription factor peptidase, site 1 | -4.303 | 4.05E-03 | 0.753 |
| 161774_f_at | Lypla1 | Lysophospholipase 1 | -4.237 | 4.31E-03 | 0.664 |
| 93600_at | Leprot | Leptin receptor overlapping transcript | -4.190 | 4.49E-03 | 0.497 |
| 100042_at | Hagh | Hydroxyacyl glutathione hydrolase | -4.172 | 4.56E-03 | 0.695 |
| 104627_at | Cds2 | CDP-diacylglycerol synthase (phosphatidate cytidylyltransferase) 2 | -4.163 | 4.60E-03 | 0.775* |
| 97247_at | Isoc1 | Isochorismatase domain containing 1 | -4.097 | 4.97E-03 | 0.744 |
| 101074_at | Ddost | Dolichyl-di-phosphooligosaccharide-protein glycotransferase | -4.033 | 5.38E-03 | 0.505 |
| 95064_at | Acaa2 | Acetyl-Coenzyme A acyltransferase 2 (mitochondrial 3-oxoacyl-Coenzyme A thiolase) | -3.963 | 5.81E-03 | 0.442 |
| 102639_at | Chst2 | Carbohydrate sulfotransferase 2 | -3.948 | 5.91E-03 | 0.551 |
| 92887_at | Ddah2 | Dimethylarginine dimethylaminohydrolase 2 | -3.904 | 6.14E-03 | 0.508 |
| 100068_at | Aldh1a1 | Aldehyde dehydrogenase family 1, subfamily A1 | -3.900 | 6.18E-03 | 0.763 |
| 160327_at | Dctn6 | Dynactin 6 | -3.898 | 6.20E-03 | 0.668 |
| 101557_at | Bckdk | Branched chain ketoacid dehydrogenase kinase | -3.896 | 6.21E-03 | 0.767 |
| 93540_at | Adprh | ADP-ribosylarginine hydrolase | -3.870 | 6.39E-03 | 0.693* |
| 97110_at | Chst2 | Carbohydrate sulfotransferase 2 | -3.816 | 6.82E-03 | 0.686 |
| 103637_at | Naga | N-acetyl galactosaminidase, alpha | -3.809 | 6.87E-03 | 0.569 |
| 160187_at | Pafah1b2 | Platelet-activating factor acetylhydrolase, isoform 1b, alpha2 subunit | -3.807 | 6.88E-03 | 0.716 |
| 93258_at | Hmbs | Hydroxymethylbilane synthase | -3.791 | 7.01E-03 | 0.730 |
| 96338_at | Egln2 | EGL nine homolog 2 (C. elegans) | -3.790 | 7.03E-03 | 0.651 |
| 104343_f_at | Pla2g12a | Phospholipase A2, group XIIA | -3.766 | 7.27E-03 | 0.413 |
| 160337_at | Isyna1 | Myo-inositol 1-phosphate synthase A1 | -3.759 | 7.35E-03 | 0.496 |
| 96243_f_at | Aldh9a1 | Aldehyde dehydrogenase 9, subfamily A1 | -3.739 | 7.50E-03 | 0.467* |
| 95468_at | Egln1 | EGL nine homolog 1 (C. elegans) | -3.680 | 8.03E-03 | 0.684 |
| 93500_at | Alas1 | Aminolevulinic acid synthase 1 | -3.669 | 8.13E-03 | 0.730 |
| 160482_at | Acaa1 | Acetyl-Coenzyme A acyltransferase 1 | -3.661 | 8.19E-03 | 0.630 |
| 102370_at | Hsd17b11 | Hydroxysteroid (17-beta) dehydrogenase 11 | -3.659 | 8.21E-03 | 0.407 |
| 103663_at | Pomgnt1 | Protein O-linked mannose beta1,2-N-acetylglucosaminyltransferase | -3.643 | 8.37E-03 | 0.652 |
| 161694_f_at | Ptgs1 | Prostaglandin-endoperoxide synthase 1 | -3.636 | 8.44E-03 | 0.641 |
| 94438_at | Pfkm | Phosphofructokinase, muscle | -3.631 | 8.50E-03 | 0.737 |
| 93007_at | Npy1r | Neuropeptide Y receptor Y1 | -3.631 | 8.50E-03 | 0.445 |
| *Transport and trafficking processes* | | |  |  |  |
| 101877_at | Slc31a1 | Solute carrier family 31, member 1 | 10.397 | 1.58E-04 | 1.542 |
| 93330_at | Aqp1 | Aquaporin 1 | 8.670 | 2.81E-04 | 3.253 |
| 93586_at | Syngr2 | Synaptogyrin 2 | 6.199 | 9.06E-04 | 1.366 |
| 103035_at | Tap1 | Transporter 1, ATP-binding cassette, sub-family B (MDR/TAP) | 6.106 | 9.68E-04 | 3.730 |
| 92736_at | Slc7a2 | Solute carrier family 7 (cationic amino acid transporter, y+ system), member 2 | 4.610 | 3.00e-03 | 2.080 |
| 100618_f_at | Slc25a5 | Solute carrier family 25 (mitochondrial carrier, adenine nucleotide translocator), member 5 | 4.532 | 3.23E-03 | 1.332 |
| 99619_at | Tmem41b | Transmembrane protein 41B | 3.984 | 5.67E-03 | 1.332 |
| 93471_at | Slc4a7 | Solute carrier family 4, sodium bicarbonate cotransporter, member 7 | 3.887 | 6.27e-03 | 2.731 |
| 95733_at | Slc29a1 | Solute carrier family 29 (nucleoside transporters), member 1 | 3.860 | 6.47e-03 | 1.703 |
| 100499_at | Stx3 | Syntaxin 3 | 3.666 | 8.15E-03 | 1.596 |
| 103467_g_at | Cyhr1 | Cysteine and histidine rich 1 | -14.184 | 6.86E-05 | 0.420 |
| 102099_f_at | Kctd17 | Potassium channel tetramerisation domain containing 17 | -9.570 | 2.08E-04 | 0.583 |
| 93045_at | Abcd3 | ATP-binding cassette, sub-family D (ALD), member 3 | -7.846 | 3.96E-04 | 0.352 |
| 103466_at | Cyhr1 | Cysteine and histidine rich 1 | -7.160 | 5.63E-04 | 0.679 |
| 102670_at | Vps54 | Vacuolar protein sorting 54 (yeast) | -6.824 | 6.47E-04 | 0.794 |
| 162080_f_at | Kctd17 | Potassium channel tetramerisation domain containing 17 | -5.630 | 1.32E-03 | 0.717 |
| 94549_at | Mfsd1 | Major facilitator superfamily domain containing 1 | -5.347 | 1.64E-03 | 0.623 |
| 93626_at | Abcg2 | ATP-binding cassette, sub-family G (WHITE), member 2 | -5.347 | 1.64E-03 | 0.573 |
| 100951_at | Pkd2 | Polycystic kidney disease 2 | -5.338 | 1.65E-03 | 0.437 |
| 96894_at | Tmed4 | Transmembrane emp24 protein transport domain containing 4 | -5.080 | 2.00E-03 | 0.635 |
| 103726_at | Yipf5 | Yip1 domain family, member 5 | -4.949 | 2.24E-03 | 0.626 |
| 93736_at | Tcn2 | Transcobalamin 2 | -4.797 | 2.54E-03 | 0.601 |
| 93440_at | Sec22b | SEC22 vesicle trafficking protein homolog B (S. cerevisiae) | -4.794 | 2.54E-03 | 0.696 |
| 96675_at | Unc50 | Unc-50 homolog (C. Elegans) | -4.754 | 2.62E-03 | 0.792 |
| 93362_at | Ap2m1 | Adaptor protein complex AP-2, mu1 | -4.649 | 2.90E-03 | 0.674 |
| 104071_at | Tnpo2 | Transportin 2 (importin 3, karyopherin beta 2b) | -4.557 | 3.16E-03 | 0.774 |
| 100943_at | Slc1a4 | Solute carrier family 1 (glutamate/neutral amino acid transporter), member 4 | -4.360 | 3.81E-03 | 0.667 |
| 93819_at | Tmed2 | Transmembrane emp24 domain trafficking protein 2 | -4.320 | 3.98E-03 | 0.588 |
| 94548_at | Mfsd1 | Major facilitator superfamily domain containing 1 | -4.248 | 4.26E-03 | 0.625 |
| 161613_at | Chmp7 | CHMP family, member 7 | -4.237 | 4.32E-03 | 0.604 |
| 93983_at | Derl1 | Der1-like domain family, member 1 | -4.160 | 4.62E-03 | 0.780 |
| 96674_at | Tnpo3 | Transportin 3 | -4.153 | 4.67E-03 | 0.631 |
| 160064_at | Stx7 | Syntaxin 7 | -4.064 | 5.16E-03 | 0.678 |
| 99672_at | Clcn4-2 | Chloride channel 4-2 | -4.063 | 5.17E-03 | 0.676 |
| 103459_at | Slc39a6 | Solute carrier family 39 (metal ion transporter), member 6 | -4.035 | 5.37e-03 | 0.585 |
| 98483_at | Cacnb3 | Calcium channel, voltage-dependent, beta 3 subunit | -4.027 | 5.42E-03 | 0.620 |
| 160291_at | Sec61a1 | Sec61 alpha 1 subunit (S. Cerevisiae) | -3.985 | 5.66E-03 | 0.595 |
| 160270_at | Lman1 | Lectin, mannose-binding, 1 | -3.954 | 5.87E-03 | 0.666 |
| 96832_at | Slc39a1 | Solute carrier family 39 (zinc transporter), member 1 | -3.774 | 7.14e-03 | 0.616 |
| 93341_r_at | Copb2 | Coatomer protein complex, subunit beta 2 (beta prime) | -3.705 | 7.81E-03 | 0.776 |
| 93818_g_at | Tmed2 | Transmembrane emp24 domain trafficking protein 2 | -3.654 | 8.27E-03 | 0.661 |
| 104514_at | Epn1 | Epsin 1 | -3.527 | 9.65E-03 | 0.740 |
| 97957_at | Slc27a4 | Solute carrier family 27 (fatty acid transporter), member 4 | -3.518 | 9.73e-03 | 0.726 |
| *Cell cycle and DNA replication* | | |  |  |  |
| 104639_i_at | Josd3 | Josephin domain containing 3 | 12.360 | 1.03E-04 | 2.400 |
| 102292_at | Gadd45a | Growth arrest and DNA-damage-inducible 45 alpha | 10.352 | 1.60E-04 | 4.522* |
| 161666_f_at | Gadd45b | Growth arrest and DNA-damage-inducible 45 beta | 8.405 | 3.13E-04 | 6.979 |
| 94288_at | Hist1h1c | Histone 1, h1c | 7.202 | 5.49E-04 | 3.448* |
| 104640_f_at | Josd3 | Josephin domain containing 3 | 6.556 | 7.32E-04 | 2.424 |
| 93023_f_at | Hist1h3f | Histone 1, h3f | 6.496 | 7.55E-04 | 2.009* |
| 99803_at | Hist1h1b | Histone 1, h1b | 6.130 | 9.59E-04 | 1.434* |
| 94805_f_at | Hist3h2a | Histone 3, h2a | 5.948 | 1.08E-03 | 5.054 |
| 96416_f_at | Hist1h3d | Histone1, h3d | 5.466 | 1.47E-03 | 1.719 |
| 160261_i_at | Lats2 | Large tumor suppressor 2 | 4.603 | 3.02E-03 | 2.769 |
| 93833_s_at | Hist1h2bc | Histone 1, h2bc | 4.555 | 3.16E-03 | 3.673 |
| 104690_at | Polm | Polymerase (DNA directed), mu | 4.120 | 4.85E-03 | 1.271 |
| 103443_at | Aim1 | Absent in melanoma 1 | 4.076 | 5.09E-03 | 1.800 |
| 94476_at | Josd3 | Josephin domain containing 3 | 4.051 | 5.23E-03 | 1.415 |
| 94638_at | Hist2h2bb | Histone 2, h2bb | 3.717 | 7.71E-03 | 1.303 |
| 93022_i_at | Hist1h3f | Histone 1, h3f | 3.684 | 7.98E-03 | 1.549* |
| 94819_f_at | Ccni | Cyclin I | -8.347 | 3.23E-04 | 0.317 |
| 98478_at | Ccng2 | Cyclin G2 | -8.109 | 3.52E-04 | 0.298 |
| 97504_at | Ccnd2 | Cyclin D2 | -8.059 | 3.64E-04 | 0.357* |
| 99563_at | Spin | Spindlin | -7.353 | 4.87E-04 | 0.436* |
| 95100_at | Anapc5 | Anaphase-promoting complex subunit 5 | -7.309 | 5.03E-04 | 0.551 |
| 98789_at | Cdkn2a | Cyclin-dependent kinase inhibitor 2A | -6.344 | 8.42E-04 | 0.135# |
| 94954_at | Anapc4 | Anaphase promoting complex subunit 4 | -5.730 | 1.23E-03 | 0.633 |
| 104482_at | Stx2 | Syntaxin2 | -5.726 | 1.23E-03 | 0.671 |
| 98609_at | Sept9 | Septin 9 | -5.687 | 1.26E-03 | 0.591 |
| 92788_f_at | Cetn3 | Centrin 3 | -5.685 | 1.26E-03 | 0.693* |
| 161010_r_at | Cdkn1b | Cyclin-dependent kinase inhibitor 1B (P27) | -5.066 | 2.05E-03 | 0.674 |
| 92350_at | Mapre1 | Microtubule-associated protein, RP/EB family, member 1 | -4.986 | 2.18E-03 | 0.720 |
| 92770_at | S100a6 | S100 calcium binding protein A6 (calcyclin) | -4.936 | 2.27E-03 | 0.626 |
| 103736_at | Sash1 | SAM and SH3 domain containing 1 | -4.177 | 4.54E-03 | 0.386 |
| 103501_at | Pura | Purine rich element binding protein A | -4.078 | 5.09E-03 | 0.481 |
| 98075_at | Cdc123 | Cell division cycle 123 homolog (S. cerevisiae) | -3.948 | 5.91E-03 | 0.800 |
| 101367_at | Dctn1 | Dynactin 1 | -3.914 | 6.10E-03 | 0.768 |
| 99135_at | Cdc37 | Cell division cycle 37 homolog (S. Cerevisiae) | -3.903 | 6.15E-03 | 0.780 |
| 96236_at | Cdc16 | CDC16 cell division cycle 16 homolog (S. cerevisiae) | -3.690 | 7.93E-03 | 0.716 |
| 160538_at | Cdk4 | Cyclin-dependent kinase 4 | -3.656 | 8.24E-03 | 0.587 |
| 99068_at | Anapc1 | Anaphase promoting complex subunit 1 | -3.515 | 9.77E-03 | 0.847 |
| *Immunity and defense* | | |  |  |  |
| 93858_at | Cxcl10 | Chemokine (C-X-C motif) ligand 10 | 15.074 | 6.18E-05 | 12.385* |
| 98501_at | Il1rl1 | Interleukin 1 receptor-like 1 | 12.979 | 8.47E-05 | 10.300* |
| 93861_f_at | Ctse | Cathepsin E | 12.742 | 9.61E-05 | 2.536 |
| 102218_at | Il6 | Interleukin 6 | 10.240 | 1.65E-04 | 20.438* |
| 101160_at | Cxcl2 | Chemokine (C-X-C motif) ligand 2 | 9.616 | 2.01E-04 | 6.452* |
| 102699_at | Mx2 | Myxovirus (influenza virus) resistance 2 | 8.756 | 2.70E-04 | 14.932* |
| 101464_at | Timp1 | Tissue inhibitor of metalloproteinase 1 | 8.703 | 2.75E-04 | 2.798 |
| 98417_at | Mx1 | Myxovirus (influenza virus) resistance 1 | 8.592 | 2.93E-04 | 3.830 |
| 103005_s_at | Cd44 | CD44 antigen | 7.600 | 4.35E-04 | 4.194 |
| 104177_at | Rsad2 | Radical S-adenosyl methionine domain containing 2 | 6.926 | 6.20E-04 | 22.089 |
| 92731_at | Ptx3 | Pentraxin related gene | 6.235 | 8.88E-04 | 3.069* |
| 104014_at | Hfe | Hemochromatosis | 5.653 | 1.29E-03 | 1.294 |
| 102717_at | Oas1g | 2’-5’ oligoadenylate synthetase 1G | 5.641 | 1.29E-03 | 1.235 |
| 93860_i_at | Ctse | Cathepsin E | 5.564 | 1.37E-03 | 2.408 |
| 98500_at | Il1rl1 | Interleukin 1 receptor-like 1 | 5.316 | 1.67E-03 | 10.974* |
| 98008_at | Cx3cl1 | Chemokine (C-X3-C motif) ligand 1 | 4.927 | 2.28E-03 | 2.919 |
| 99379_f_at | LOC56628 | Similar to H-2 class I histocompatibility antigen, L-D alpha chain precursor | 4.731 | 2.69E-03 | 2.237 |
| 101658_f_at | H2-Q8 | Histocompatibility 2, Q region locus 8 | 4.634 | 2.95E-03 | 2.001 |
| 101681_f_at | H2-Bl | Histocompatibility 2, blastocyst | 4.510 | 3.29E-03 | 1.441 |
| 102430_at | Myd88 | Myeloid differentiation primary response gene 88 | 4.492 | 3.34E-03 | 1.425 |
| 161023_at | Il15ra | Interleukin 15 receptor, alpha chain | 4.208 | 4.42E-03 | 1.366 |
| 97173_f_at | EG630499 | Predicted gene, EG630499 | 3.872 | 6.37E-03 | 2.104 |
| 98776_at | Igh-6 | Immunoglobulin heavy chain 6 (heavy chain of igm) | 3.818 | 6.79E-03 | 1.215 |
| 102161_f_at | H2-Q2 | Histocompatibility 2, Q region locus 2 | 3.793 | 7.00E-03 | 2.228 |
| 98813_at | Rel | Reticuloendotheliosis oncogene | 3.772 | 7.18E-03 | 1.292 |
| 93087_r_at | Igk-C | Immunoglobulin kappa chain, constant region | 3.742 | 7.47E-03 | 1.240 |
| 96221_at | Traf3ip2 | Traf3 interacting protein 2 | 3.657 | 8.23E-03 | 1.645 |
| 96912_s_at | Ctla2a | Cytotoxic T lymphocyte-associated protein 2 alpha | -5.545 | 1.39E-03 | 0.196 |
| 103032_at | Tpst1 | Protein-tyrosine sulfotransferase 1 | -4.614 | 2.99E-03 | 0.492 |
| 102960_at | Rga | Recombination activating gene 1 gene activation | -4.443 | 3.53E-03 | 0.681 |
| 103422_at | Cd1d1 | CD1d1 antigen | -4.426 | 3.60E-03 | 0.526* |
| 104501_at | Vapb | Vesicle-associated membrane protein, associated protein B and C | -4.331 | 3.92E-03 | 0.624 |
| 101102_at | Igbp1 | Immunoglobulin (CD79A) binding protein 1 | -4.205 | 4.43E-03 | 0.586 |
| 93546_s_at | Cbfb | Core binding factor beta | -3.827 | 6.72E-03 | 0.632* |
| 95466_at | Cotl1 | Coactosin-like 1 (Dictyostelium) | -3.565 | 9.22E-03 | 0.690 |
| *Response to interferon* | | |  |  |  |
| 102401_at | Irf1 | Interferon regulatory factor 1 | 13.448 | 7.32E-05 | 8.326 |
| 93085_at | Psmb9 | Proteosome (prosome, macropain) subunit, beta type 9 (large multifunctional peptidase 2) | 11.280 | 1.28E-04 | 2.651 |
| 98410_at | Iigp2 | Interferon inducible GTPase 2 | 5.822 | 1.17E-03 | 4.114 |
| 162202_f_at | Irf7 | Interferon regulatory factor 7 | 5.293 | 1.69E-03 | 1.449 |
| 98465_f_at | Ifi204 | Interferon activated gene 204 | 5.073 | 2.02E-03 | 5.611 |
| 103963_f_at | Iigp1 | Interferon inducible gtpase 1 | 5.069 | 2.03E-03 | 5.818 |
| 95303_at | Ifitm6 | Interferon induced transmembrane protein 6 | 4.994 | 2.16E-03 | 1.365 |
| 96764_at | Iigp1 | Interferon inducible GTPase 1 | 4.810 | 2.50E-03 | 8.259 |
| 160933_at | Igtp | Interferon gamma induced GTPase | 4.682 | 2.83E-03 | 3.523 |
| 102791_at | Psmb8 | Proteosome (prosome, macropain) subunit, beta type 8 (large multifunctional peptidase 7) | 4.657 | 2.88E-03 | 3.602 |
| 99099_at | Stat3 | Signal transducer and activator of transcription 3 | 4.568 | 3.13E-03 | 1.686 |
| 94774_at | Ifi202b | Interferon activated gene 202B | 4.557 | 3.16E-03 | 5.256 |
| 99100_at | Stat5a | Signal transducer and activator of transcription 5A | 4.421 | 3.62E-03 | 1.583 |
| 102906_at | Tgtp | T-cell specific GTPase | 4.370 | 3.78E-03 | 8.042* |
| 101465_at | Stat1 | Signal transducer and activator of transcription 1 | 4.289 | 3.88E-03 | 3.803*# |
| 94224_s_at | Ifi203 | Interferon activated gene 203 | 4.286 | 4.11E-03 | 3.446 |
| 100013_at | Ifi35 | Interferon-induced protein 35 | 4.158 | 4.63E-03 | 2.700 |
| 161173_f_at | Ifi202b | Interferon activated gene 202B | 4.076 | 5.10E-03 | 3.309 |
| 98822_at | G1p2 | Interferon, alpha-inducible protein | 3.981 | 5.69E-03 | 4.839 |
| 161511_f_at | G1p2 | Interferon, alpha-inducible protein | 3.849 | 6.55E-03 | 4.757 |
| 103446_at | Ifih1 | Interferon induced with helicase C domain 1 | 3.777 | 7.13E-03 | 3.101 |
| 104669_at | Irf7 | Interferon regulatory factor 7 | 3.725 | 7.63E-03 | 3.433 |
| 92232_at | Socs3 | Suppressor of cytokine signaling 3 | 3.707 | 7.80E-03 | 3.556* |
| 99509_s_at | Jak3 | Janus kinase 3 | 3.669 | 8.12E-03 | 1.405 |
| 160694_at | Ifngr2 | Interferon gamma receptor 2 | -4.064 | 5.16E-03 | 0.568 |
| 99975_at | Prkrir | Protein-kinase, interferon-inducible double stranded RNA dependent inhibitor, repressor of (P58 repressor) | -3.771 | 7.20E-03 | 0.688* |
| 160875_at | Psmb1 | Proteasome (prosome, macropain) subunit, beta type 1 | -3.674 | 8.07E-03 | 0.694 |
| *Cell adhesion and migration* | | |  |  |  |
| 100484_at | Mmp13 | Matrix metallopeptidase 13 | 11.564 | 1.24E-04 | 25.898* |
| 101982_at | Vasp | Vasodilator-stimulated phosphoprotein | 8.814 | 2.61E-04 | 1.908 |
| 103039_at | Itga5 | Integrin alpha 5 (fibronectin receptor alpha) | 7.454 | 4.67E-04 | 7.328 |
| 94964_at | Vcl | Vinculin | 7.024 | 5.99E-04 | 3.346 |
| 94643_at | Pvr | Poliovirus receptor | 6.853 | 6.43E-04 | 8.025 |
| 98833_at | Mmp3 | 32ntrax metallopeptidase 3 | 6.697 | 6.86E-04 | 11.415 |
| 102280_at | Pcdh7 | Protocadherin 7 | 4.621 | 2.98E-03 | 2.098 |
| 100428_at | Lamc2 | Laminin, gamma 2 | 4.569 | 3.12E-03 | 2.494* |
| 103434_at | Pscd3 | Pleckstrin homology, Sec7 and coiled-coil domains 3 | 3.775 | 7.14E-03 | 2.155 |
| 95141_at | Capzb | Capping protein (actin filament) muscle Z-line, beta | 3.761 | 7.32E-03 | 1.184 |
| 104469_at | Pdpn | Podoplanin | -12.417 | 9.84E-05 | 0.119 |
| 101551_s_at | Tes | Testis derived transcript | -7.063 | 5.88E-04 | 0.383 |
| 162263_f_at | Lamb1-1 | Laminin B1 subunit 1 | -6.754 | 6.75E-04 | 0.432 |
| 97750_at | Lamr1 | Laminin receptor 1 (ribosomal protein SA) | -5.857 | 1.15E-03 | 0.576 |
| 104761_at | Antxr2 | 32ntrax toxin receptor 2 | -5.448 | 1.49E-03 | 0.474 |
| 95643_at | Wdr6 | WD repeat domain 6 | -5.097 | 1.98E-03 | 0.647* |
| 102852_at | Cdh2 | Cadherin 2 | -5.049 | 2.07E-03 | 0.207 |
| 161247_f_at | Dgcr2 | DiGeorge syndrome critical region gene 2 | -4.834 | 2.45E-03 | 0.619 |
| 96742_at | Dpt | Dermatopontin | -4.663 | 2.87E-03 | 0.069 |
| 96187_at | Pkp4 | Plakophilin 4 | -4.634 | 2.95E-03 | 0.672 |
| 101861_at | Sgce | Sarcoglycan, epsilon | -4.578 | 3.10E-03 | 0.519 |
| 101948_at | Lamb1-1 | Laminin B1 subunit 1 | -4.538 | 3.21E-03 | 0.480 |
| 98428_at | Spast | Spastin | -4.516 | 3.28E-03 | 0.632 |
| 160607_at | Pard3 | Par-3 (partitioning defective 3) homolog (C. Elegans) | -4.129 | 4.81E-03 | 0.708 |
| 102656_at | Itga4 | Integrin alpha 4 | -4.071 | 5.13E-03 | 0.825 |
| 162034_r_at | Antxr2 | 33ntrax toxin receptor 2 | -3.869 | 6.41E-03 | 0.800 |
| 96692_at | Fgfr1op2 | FGFR1 oncogene partner 2 | -3.781 | 7.10E-03 | 0.769 |
| 94236_at | Nisch | Nischarin | -3.715 | 7.73E-03 | 0.577 |
| 104602_at | Wipf1 | WAS/WASL interacting protein family, member 1 | -3.697 | 7.86E-03 | 0.386 |
| 100457_at | Glg1 | Golgi apparatus protein 1 | -3.651 | 8.29E-03 | 0.653 |
| 160667_at | Evl | Ena-vasodilator stimulated phosphoprotein | -3.611 | 8.72E-03 | 0.587 |
| *RNA metabolism and processing* | | |  |  |  |
| 160208_at | Sf3b3 | Splicing factor 3b, subunit 3 | 4.736 | 2.67E-03 | 1.401 |
| 102678_at | Trim21 | Tripartite motif protein 21 | 4.409 | 3.67E-03 | 1.658 |
| 99923_at | Pcf11 | Cleavage and polyadenylation factor subunit homolog (S. cerevisiae) | 4.334 | 3.91E-03 | 1.759 |
| 96188_at | Adar | Adenosine deaminase, RNA-specific | 4.244 | 4.28E-03 | 3.214 |
| 102031_at | Rnaseh1 | Ribonuclease H1 | 3.798 | 6.96E-03 | 1.241 |
| 102741_at | Adar | Adenosine deaminase, RNA-specific | 3.654 | 8.27E-03 | 1.591 |
| 100534_at | Tsnax | Translin-associated factor X | -7.947 | 3.78E-04 | 0.638* |
| 102399_at | Rbpms | RNA binding protein gene with multiple splicing | -6.885 | 6.31E-04 | 0.356 |
| 92775_at | Pabpc4 | Poly A binding protein, cytoplasmic 4 | -6.363 | 8.26E-04 | 0.465 |
| 103449_at | Lsm14b | LSM14 homolog B (SCD6, S. cerevisiae) | -6.142 | 9.45E-04 | 0.582 |
| 161432_f_at | Sart3 | Squamous cell carcinoma antigen recognized by T-cells 3 | -6.060 | 9.79E-04 | 0.784 |
| 97293_at | Rbm10 | RNA binding motif protein 10 | -6.005 | 1.03E-03 | 0.644* |
| 100720_at | Pabpc1 | Poly A binding protein, cytoplasmic 1 | -5.226 | 1.80E-03 | 0.569 |
| 95910_f_at | Rbed1 | RNA binding motif and ELMO domain 1 | -4.945 | 2.25E-03 | 0.685 |
| 160772_i_at | Slu7 | SLU7 splicing factor homolog (S. cerevisiae) | -4.821 | 2.48E-03 | 0.519 |
| 100559_at | Dhx16 | DEAH (Asp-Glu-Ala-His) box polypeptide 16 | -4.607 | 3.00E-03 | 0.595 |
| 93130_at | Rrp1b | Ribosomal RNA processing 1 homolog B (S. cerevisiae) | -4.586 | 3.08E-03 | 0.675 |
| 160079_i_at | Wac | WW domain containing adaptor with coiled-coil | -4.427 | 3.59E-03 | 0.753 |
| 162424_f_at | Ddx17 | DEAD (Asp-Glu-Ala-Asp) box polypeptide 17 | -4.427 | 3.59E-03 | 0.497 |
| 103888_at | Rbpms | RNA binding protein gene with multiple splicing | -4.153 | 4.68E-03 | 0.504 |
| 160352_at | Pcbp4 | Poly(rc) binding protein 4 | -4.147 | 4.73E-03 | 0.778 |
| 92623_at | Csde1 | Cold shock domain containing E1, RNA binding | -4.114 | 4.88E-03 | 0.592 |
| 96621_at | Pih1d1 | PIH1 domain containing 1 | -4.090 | 5.00E-03 | 0.812 |
| 94509_at | Ncbp2 | Nuclear cap binding protein subunit 2 | -4.085 | 5.05E-03 | 0.593* |
| 103414_at | Skiv2l | Superkiller viralicidic activity 2-like (S. Cerevisiae ) | -4.084 | 5.05E-03 | 0.730 |
| 104609_at | Hnrpul2 | Heterogeneous nuclear ribonucleoprotein U-like 2 | -3.981 | 5.69E-03 | 0.721 |
| 92517_at | Rbms2 | RNA binding motif, single stranded interacting protein 2 | -3.973 | 5.75E-03 | 0.655 |
| 97329_at | Ints3 | Integrator complex subunit 3 | -3.564 | 9.23E-03 | 0.735 |
| 103678_at | Sbno1 | Sno, strawberry notch homolog 1 (Drosophila) | -3.558 | 9.28E-03 | 0.640 |
| 94067_at | Dcps | Decapping enzyme, scavenger | -3.525 | 9.68E-03 | 0.679 |
| *Cell development and differentiation* | | |  |  |  |
| 100127_at | Crabp2 | Cellular retinoic acid binding protein II | 19.265 | 3.20E-05 | 16.587 |
| 160162_at | Tagln2 | Transgelin 2 | 11.584 | 1.21E-04 | 3.114* |
| 92367_at | Sil | Tal1 interrupting locus | 5.924 | 1.09E-03 | 1.445 |
| 103839_at | Sphk1 | Sphingosine kinase 1 | 5.553 | 1.38E-03 | 2.606 |
| 94489_at | Ptp4a1 | Protein tyrosine phosphatase 4a1 | 5.236 | 1.77E-03 | 2.457 |
| 92399_at | Runx1 | Runt related transcription factor 1 | 4.736 | 2.67E-03 | 3.050 |
| 97487_at | Serpine2 | Serine (or cysteine) peptidase inhibitor, clade E, member 2 | 4.532 | 3.23E-03 | 4.611 |
| 94804_at | Pbx1 | Pre B-cell leukemia transcription factor 1 | 4.335 | 3.90E-03 | 1.258 |
| 93866_s_at | Mgp | Matrix Gla protein | 4.321 | 3.97E-03 | 25.407 |
| 92507_at | Utrn | Utrophin | 4.206 | 4.42E-03 | 1.576 |
| 93873_s_at | Hoxa11s | Homeo box A11, opposite strand transcript | 4.165 | 4.59E-03 | 1.691 |
| 98796_at | Mip | Major intrinsic protein of eye lens fiber | 4.156 | 4.65E-03 | 1.258 |
| 103397_at | Hrb | HIV-1 Rev binding protein | 3.929 | 6.03E-03 | 1.469 |
| 98525_f_at | Edr1 | Erythroid differentiation regulator 1 | 3.789 | 7.04E-03 | 3.021 |
| 104021_at | Hoxa11 | Homeo box A11 | 3.666 | 8.15E-03 | 2.240 |
| 100992_at | Phc1 | Polyhomeotic-like 1 (Drosophila) | -5.762 | 1.20E-03 | 0.547 |
| 95557_at | Bmp1 | Bone morphogenetic protein 1 | -5.357 | 1.62E-03 | 0.597 |
| 101484_at | Nbr1 | Neighbor of Brca1 gene 1 | -4.715 | 2.72E-03 | 0.615 |
| 101095_at | Mfap2 | Microfibrillar-associated protein 2 | -4.713 | 2.72E-03 | 0.163 |
| 99458_i_at | Mark2 | MAP/microtubule affinity-regulating kinase 2 | -4.687 | 2.82E-03 | 0.514 |
| 96684_at | Grsf1 | G-rich RNA sequence binding factor 1 | -4.592 | 3.05E-03 | 0.619 |
| 93951_at | Golga3 | Golgi autoantigen, golgin subfamily a, 3 | -4.517 | 3.27E-03 | 0.676 |
| 103356_at | Dock7 | Dedicator of cytokinesis 7 | -4.355 | 3.83E-03 | 0.671 |
| 100876_at | Fez1 | Fasciculation and elongation protein zeta 1 (zygin I) | -4.344 | 3.87E-03 | 0.419 |
| 160819_at | Ndrg4 | N-myc downstream regulated gene 4 | -4.195 | 4.47E-03 | 0.606 |
| 160453_at | Ryk | Receptor-like tyrosine kinase | -3.843 | 6.60E-03 | 0.587 |
| 92643_at | Nf2 | Neurofibromatosis 2 | -3.731 | 7.56E-03 | 0.554 |
| 98555_at | Ttc3 | Tetratricopeptide repeat domain 3 | -3.696 | 7.87E-03 | 0.623 |
| 160498_at | Ldb1 | LIM domain binding 1 | -3.691 | 7.92E-03 | 0.703 |
| 101858_at | Rfng | Radical fringe gene homolog (Drosophila) | -3.588 | 8.97E-03 | 0.743 |
| *Protein biosynthesis and ribosome organization* | | |  |  |  |
| 161961_at | Rpl19 | Ribosomal protein L19 | 5.170 | 1.87E-03 | 1.269 |
| 99849_at | Wdr92 | WD repeat domain 92 | 3.663 | 8.18E-03 | 2.330 |
| 93787_f_at | Mrpl18 | Mitochondrial ribosomal protein L18 | -9.408 | 2.17E-04 | 0.560 |
| 93859_at | Mtif2 | Mitochondrial translational initiation factor 2 | -8.898 | 2.54E-04 | 0.530* |
| 161480_i_at | Rplp1 | Ribosomal protein, large, P1 | -8.219 | 3.41E-04 | 0.619 |
| 160365_at | Eif2s2 | Eukaryotic translation initiation factor 2, subunit 2 (beta) | -4.712 | 2.72E-03 | 0.539 |
| 101213_at | Arbp | Acidic ribosomal phosphoprotein P0 | -4.681 | 2.83E-03 | 0.718 |
| 160345_at | Mrpl34 | Mitochondrial ribosomal protein L34 | -4.399 | 3.69E-03 | 0.737 |
| 96942_at | Eif3s6ip | Eukaryotic translation initiation factor 3, subunit 6 interacting protein | -4.100 | 4.93E-03 | 0.699 |
| 100557_g_at | Eif4b | Eukaryotic translation initiation factor 4B | -4.038 | 5.34E-03 | 0.594 |
| 94766_at | Eef1a1 | Eukaryotic translation elongation factor 1 alpha 1 | -3.858 | 6.48E-03 | 0.807 |
| 160071_at | Rpp30 | Ribonuclease P/MRP 30 subunit (human) | -3.840 | 6.62E-03 | 0.827 |
| 98936_at | Sars1 | Seryl-aminoacyl-tRNA synthetase 1 | -3.838 | 6.63E-03 | 0.697 |
| 101072_at | Eif4ebp2 | Eukaryotic translation initiation factor 4E binding protein 2 | -3.831 | 6.68E-03 | 0.611 |
| 102058_at | Mrpl9 | Mitochondrial ribosomal protein L9 | -3.766 | 7.27E-03 | 0.630 |
| 96864_at | Mrps26 | Mitochondrial ribosomal protein S26 | -3.599 | 8.84E-03 | 0.724 |
| 160956_r_at | Guf1 | GUF1 GTPase homolog (S. cerevisiae) | -3.598 | 8.84E-03 | 0.779 |
| 98085_f_at | Rps28 | Ribosomal protein S28 | -3.585 | 8.98E-03 | 0.688 |
| 97083_at | Eif2s2 | Eukaryotic translation initiation factor 2, subunit 2 (beta) | -3.572 | 9.12E-03 | 0.730 |
| 101129_at | Rpl5 | Ribosomal protein L5 | -3.564 | 9.23E-03 | 0.679 |
| 96158_at | Paip2b | Poly(A) binding protein interacting protein 2B | -3.527 | 9.65E-03 | 0.733 |
| 103537_at | Eif2ak3 | Eukaryotic translation initiation factor 2 alpha kinase 3 | -3.523 | 9.68E-03 | 0.680 |
| 101137_at | Rps3 | Ribosomal protein S3 | -3.516 | 9.75E-03 | 0.770 |
| *Apoptosis* | | |  |  |  |
| 99392_at | Tnfaip3 | Tumor necrosis factor, alpha-induced protein 3 | 18.398 | 3.89E-05 | 45.832 |
| 102313_at | Gch1 | GTP cyclohydrolase 1 | 9.481 | 2.15E-04 | 3.179 |
| 98433_at | Bid | BH3 interacting domain death agonist | 7.631 | 4.32E-04 | 2.445# |
| 98056_at | Phlda3 | Pleckstrin homology-like domain, family A, member 3 | 6.804 | 6.61E-04 | 2.704 |
| 93536_at | Bax | Bcl2-associated X protein | 6.134 | 9.54E-04 | 1.695# |
| 160829_at | Phlda1 | Pleckstrin homology-like domain, family A, member 1 | 5.855 | 1.15E-03 | 12.759* |
| 93093_at | Mcl1 | Myeloid cell leukemia sequence 1 | 4.187 | 4.50E-03 | 1.909 |
| 94196_at | Ikbkg | Inhibitor of kappab kinase gamma | 4.057 | 5.19E-03 | 1.315 |
| 160489_at | Tnfaip2 | Tumor necrosis factor, alpha-induced protein 2 | 3.964 | 5.80E-03 | 1.847 |
| 93416_at | Tnfsf11 | Tumor necrosis factor (ligand) superfamily, member 11 | 3.873 | 6.35E-03 | 1.927* |
| 97091_at | Ripk1 | Receptor (TNFRSF)-interacting serine-threonine kinase 1 | 3.836 | 6.65E-03 | 1.262 |
| 101995_at | Sqstm1 | Sequestosome 1 | 3.700 | 7.84E-03 | 1.892* |
| 96166_at | Tex261 | Testis expressed gene 261 | -6.237 | 8.81E-04 | 0.621* |
| 101517_at | Tex261 | Testis expressed gene 261 | -6.025 | 1.01E-03 | 0.678* |
| 100115_at | Qrich1 | Glutamine-rich 1 | -4.504 | 3.31E-03 | 0.694 |
| 162490_f_at | Pdcd6 | Programmed cell death 6 | -4.392 | 3.71E-03 | 0.778 |
| 98424_at | Ptpn13 | Protein tyrosine phosphatase, non-receptor type 13 | -4.325 | 3.96E-03 | 0.499* |
| 160931_at | Dnm1l | Dynamin 1-like | -4.154 | 4.67E-03 | 0.738 |
| 93591_at | Pef1 | Penta-EF hand domain containing 1 | -4.046 | 5.26E-03 | 0.738 |
| 94458_at | Casp6 | Caspase 6 | -3.686 | 7.96E-03 | 0.658 |
| 101892_f_at | Fts | Fused toes | -3.680 | 8.02E-03 | 0.405 |
| 100988_at | Bcl2l11 | BCL2-like 11 (apoptosis facilitator) | -3.649 | 8.31E-03 | 0.668 |
| *Ubiquitin cycle* | | |  |  |  |
| 95024_at | Usp18 | Ubiquitin specific peptidase 18 | 4.391 | 3.72E-03 | 7.320 |
| 104715_at | Ubap2 | Ubiquitin-associated protein 2 | 4.087 | 5.02E-03 | 1.135 |
| 97729_at | Enc1 | Ectodermal-neural cortex 1 | 4.081 | 5.07E-03 | 1.653 |
| 161992_at | Ppm1m | Protein phosphatase 1M | 4.080 | 5.08E-03 | 1.327 |
| 160096_at | Spop | Speckle-type POZ protein | -5.210 | 1.82E-03 | 0.657 |
| 103713_at | Usp9x | Ubiquitin specific peptidase 9, X chromosome | -4.953 | 2.24E-03 | 0.586 |
| 94457_at | Rbck1 | RanBP-type and C3HC4-type zinc finger containing 1 | -4.776 | 2.59E-03 | 0.693 |
| 98906_at | Fbxo9 | F-box only protein 9 | -4.692 | 2.79E-03 | 0.616 |
| 104525_at | Ppm1m | Protein phosphatase 1M | -4.660 | 2.87E-03 | 0.658 |
| 101461_f_at | Pja1 | Praja1, RING-H2 motif containing | -4.658 | 2.88E-03 | 0.564 |
| 95057_at | Herpud1 | Homocysteine-inducible, endoplasmic reticulum stress-inducible, ubiquitin-like domain member 1 | -4.658 | 2.88E-03 | 0.543 |
| 102646_at | Usp34 | Ubiquitin specific peptidase 34 | -4.605 | 3.02E-03 | 0.674 |
| 102813_f_at | Ube1dc1 | Ubiquitin-activating enzyme E1-domain containing 1 | -4.598 | 3.04E-03 | 0.566 |
| 99106_at | Cops6 | COP9 (constitutive photomorphogenic) homolog, subunit 6 (Arabidopsis thaliana) | -4.425 | 3.60E-03 | 0.567 |
| 97530_at | Ube2i | Ubiquitin-conjugating enzyme E2I | -4.380 | 3.75E-03 | 0.670 |
| 96232_at | Cul2 | Cullin 2 | -4.288 | 4.11E-03 | 0.733 |
| 94878_at | Btbd1 | BTB (POZ) domain containing 1 | -4.285 | 4.12E-03 | 0.667* |
| 102812_i_at | Ube1dc1 | Ubiquitin-activating enzyme E1-domain containing 1 | -4.085 | 5.05E-03 | 0.474 |
| 101462_r_at | Pja1 | Praja1, RING-H2 motif containing | -3.926 | 6.04E-03 | 0.718 |
| 104109_at | Fbxo21 | F-box only protein 21 | -3.746 | 7.45E-03 | 0.610 |
| 101581_at | Ube3a | Ubiquitin protein ligase E3A | -3.655 | 8.25E-03 | 0.631 |
| 95682_at | Ddb1 | Damage specific DNA binding protein 1 | -3.580 | 9.05E-03 | 0.556 |
| 93101_s_at | Nedd4 | Neural precursor cell expressed, developmentally down-regulted gene 4 | -3.557 | 9.30E-03 | 0.547* |
| *Electron transport and energy production* | | |  |  |  |
| 96878_at | Cyb5b | Cytochrome b5 type B | 6.530 | 7.41E-04 | 1.622 |
| 93187_at | Trim47 | Tripartite motif protein 47 | 6.236 | 8.83E-04 | 2.135 |
| 96603_at | Qsox1 | Quiescin Q6 | 3.876 | 6.33E-03 | 1.938 |
| 101370_at | Kpna1 | Karyopherin (importin) alpha 1 | 3.653 | 8.28E-03 | 1.540 |
| 96831_at | Pdia5 | Protein disulfide isomerase associated 5 | -15.272 | 5.26E-05 | 0.629 |
| 160734_at | Ap3s1 | Adaptor-related protein complex 3, sigma 1 subunit | -4.738 | 2.67E-03 | 0.441 |
| 97013_f_at | Cyba | Cytochrome b-245, alpha polypeptide | -4.551 | 3.17E-03 | 0.576 |
| 103018_at | Uso1 | USO1 homolog, vesicle docking protein (yeast) | -4.444 | 3.53E-03 | 0.640 |
| 92587_at | Fdx1 | Ferredoxin 1 | -4.365 | 3.80E-03 | 0.623 |
| 160088_at | Fmo5 | Flavin containing monooxygenase 5 | -4.343 | 3.87E-03 | 0.801 |
| 93820_at | Cox7a2 | Cytochrome c oxidase, subunit viia 2 | -4.206 | 4.42E-03 | 0.583 |
| 99979_at | Cyp1b1 | Cytochrome P450, family 1, subfamily b, polypeptide 1 | -4.011 | 5.51E-03 | 0.227* |
| 160383_at | Cox7a2l | Cytochrome c oxidase subunit viia polypeptide 2-like | -3.896 | 6.21E-03 | 0.585 |
| 160076_at | Mtx2 | Metaxin 2 | -3.777 | 7.13E-03 | 0.735 |
| 96280_at | Ndufa2 | NADH dehydrogenase (ubiquinone) 1 alpha subcomplex, 2 | -3.629 | 8.54E-03 | 0.595 |
| 101989_at | Uqcrc1 | Ubiquinol-cytochrome c reductase core protein 1 | -3.520 | 9.72E-03 | 0.677 |
| 97485_at | Pcyox1 | Prenylcysteine oxidase 1 | -3.515 | 9.77E-03 | 0.605 |
| *Cytoskeleton organization and biogenesis* | | |  |  |  |
| 92608_at | Csrp1 | Cysteine and glycine-rich protein 1 | 5.439 | 1.50E-03 | 3.749 |
| 160065_s_at | Csrp1 | Cysteine and glycine-rich protein 1 | 5.286 | 1.70E-03 | 3.618 |
| 103234_at | Nefh | Neurofilament, heavy polypeptide | 4.480 | 3.38E-03 | 1.193 |
| 102331_at | St5 | Suppression of tumorigenicity 5 | 4.082 | 5.06E-03 | 1.417 |
| 98409_at | Myo1b | Myosin IB | -9.647 | 1.99E-04 | 0.335 |
| 98129_at | Tmsb10 | Thymosin, beta 10 | -6.049 | 9.91E-04 | 0.583 |
| 98454_at | Palm | Paralemmin | -5.150 | 1.90E-03 | 0.721 |
| 98402_at | Macf1 | Microtubule-actin crosslinking factor 1 | -5.094 | 1.98E-03 | 0.532 |
| 95151_at | Plekho1 | Pleckstrin homology domain containing, family O member 1 | -4.187 | 4.50E-03 | 0.693 |
| 95150_at | Plekho1 | Pleckstrin homology domain containing, family O member 1 | -4.061 | 5.18E-03 | 0.513 |
| 93567_at | Pfn2 | Profilin 2 | -3.945 | 5.93E-03 | 0.513 |
| M12481_3_st | Actb | Actin, beta, cytoplasmic | -3.817 | 6.80E-03 | 0.566 |
| 95152_g_at | Plekho1 | Pleckstrin homology domain containing, family O member 1 | -3.811 | 6.84E-03 | 0.715 |
| 161698_f_at | Mtap7d1 | Microtubule-associated protein 7 domain containing 1 | -3.771 | 7.19E-03 | 0.684 |
| 94535_at | Add1 | Adducin 1 (alpha) | -3.526 | 9.66E-03 | 0.503* |
| *Cell growth and proliferation* | | |  |  |  |
| 92472_f_at | Slfn2 | Schlafen 2 | 5.251 | 1.76E-03 | 4.328 |
| 92471_i_at | Slfn2 | Schlafen 2 | 4.764 | 2.60E-03 | 3.406 |
| 94704_at | Wisp2 | WNT1 inducible signaling pathway protein 2 | 4.344 | 3.87E-03 | 2.102 |
| 96920_at | Htra1 | Htra serine peptidase 1 | -11.030 | 1.37E-04 | 0.126 |
| 104390_at | Anp32a | Acidic (leucine-rich) nuclear phosphoprotein 32 family, member A | -9.166 | 2.38E-04 | 0.452 |
| 104367_at | Eps15 | Epidermal growth factor receptor pathway substrate 15 | -6.295 | 8.53E-04 | 0.387 |
| 104118_at | Armc10 | Armadillo repeat containing 10 | -5.601 | 1.34E-03 | 0.495 |
| 97953_g_at | Tsc2 | Tuberous sclerosis 2 | -4.931 | 2.27E-03 | 0.429 |
| 93372_at | Anp32a | Acidic (leucine-rich) nuclear phosphoprotein 32 family, member A | -4.648 | 2.91E-03 | 0.262 |
| 104006_at | Eps15 | Epidermal growth factor receptor pathway substrate 15 | -4.474 | 3.40E-03 | 0.742 |
| 97951_s_at | Tsc2 | Tuberous sclerosis 2 | -3.792 | 7.01E-03 | 0.609 |
| 97474_r_at | Ptn | Pleiotrophin | -3.746 | 7.44E-03 | 0.265 |
| 160527_at | Igfbp7 | Insulin-like growth factor binding protein 7 | -3.589 | 8.95E-03 | 0.666 |
| *Angiogenesis* | | |  |  |  |
| 102737_at | Edn1 | Endothelin 1 | 13.768 | 7.09E-05 | 10.073 |
| 102738_s_at | Edn1 | Endothelin 1 | 12.327 | 1.05E-04 | 4.823 |
| 94147_at | Serpine1 | Serine (or cysteine) peptidase inhibitor, clade E, member 1 | 11.678 | 1.19E-04 | 31.094* |
| 98802_at | Ereg | Epiregulin | 10.699 | 1.53E-04 | 21.713* |
| 92730_at | Hbegf | Heparin-binding EGF-like growth factor | 8.636 | 2.86E-04 | 7.410 |
| 103520_at | Vegfa | Vascular endothelial growth factor A | 8.109 | 3.50E-04 | 1.891* |
| 101882_s_at | Col18a1 | Procollagen, type XVIII, alpha 1 | 7.280 | 5.10E-04 | 2.897 |
| 101881_g_at | Col18a1 | Procollagen, type XVIII, alpha 1 | 7.258 | 5.22E-04 | 5.575 |
| 99638_at | Col18a1 | Procollagen, type XVIII, alpha 1 | 5.006 | 2.14E-03 | 2.024 |
| 162483_f_at | Col18a1 | Procollagen, type XVIII, alpha 1 | 3.801 | 6.93E-03 | 1.798 |
| 104551_at | Cul7 | Cullin 7 | 3.712 | 7.75E-03 | 1.234 |
| 103671_at | Htatip2 | HIV-1 tat interactive protein 2, homolog (human) | -5.908 | 1.11E-03 | 0.575 |
| 103001_at | Vegfb | Vascular endothelial growth factor B | -3.883 | 6.29E-03 | 0.676 |
| *Proteolysis and peptidolysis* | | |  |  |  |
| 97933_at | 2010111I01Rik | RIKEN cDNA 2010111I01 gene | 8.558 | 2.97E-04 | 1.940 |
| 98976_at | Adamdec1 | ADAM-like, decysin 1 | 4.385 | 3.74E-03 | 1.229 |
| 102407_at | Cma1 | Chymase 1, mast cell | 4.249 | 4.26E-03 | 1.551 |
| 95662_at | X83328 | EST X83328 | -5.587 | 1.35E-03 | 0.577 |
| 96211_at | Dpp8 | Dipeptidylpeptidase 8 | -5.586 | 1.36E-03 | 0.596 |
| 160328_at | Lonp1 | lon peptidase 1, mitochondrial | -4.455 | 3.48E-03 | 0.532 |
| 96541_at | X83328 | EST X83328 | -3.923 | 6.06E-03 | 0.610 |
| *Microtubule dynamics* | | |  |  |  |
| 100342_i_at | Tuba1 | Tubulin, alpha 1 | -7.468 | 4.62E-04 | 0.535 |
| 103315_at | Tnrc6a | Trinucleotide repeat containing 6a | -5.373 | 1.59E-03 | 0.449 |
| 97908_at | Rmnd5a | Required for meiotic nuclear division 5 homolog A (S. cerevisiae) | -4.844 | 2.44E-03 | 0.533 |
| 98461_at | Knsl8 | Kinesin-like 8 | -4.115 | 4.87E-03 | 0.688 |
| 94863_r_at | Dynlrb1 | Dynein light chain roadblock-type 1 | -3.761 | 7.32E-03 | 0.727 |
| *Protein folding* | | |  |  |  |
| 99082_at | Fkbp10 | FK506 binding protein 10 | 5.439 | 1.50E-03 | 1.347 |
| 100353_g_at | Hspa4 | Heat shock protein 4 | 4.064 | 5.15E-03 | 1.545 |
| 97868_at | Dnaja3 | DnaJ (Hsp40) homolog, subfamily A, member 3 | -5.634 | 1.31E-03 | 0.717 |
| 95112_f_at | Ppil2 | Peptidylprolyl isomerase (cyclophilin)-like 2 | -4.546 | 3.18E-03 | 0.719 |
| *Coagulation* | | |  |  |  |
| 97689_at | F3 | Coagulation factor III | 7.218 | 5.38E-04 | 6.059* |
| 160649_at | Gp1bb | Glycoprotein Ib, beta polypeptide | -4.281 | 4.13E-03 | 0.769 |
| 95474_at | F2r | Coagulation factor II (thrombin) receptor | -3.870 | 6.40E-03 | 0.516 |
| *DNA repair* | | |  |  |  |
| 103564_at | Uvrag | UV radiation resistance associated gene | 7.441 | 4.69E-04 | 1.323 |
| 93019_at | H2afx | H2A histone family, member X | 3.709 | 7.78E-03 | 2.152* |
| 99938_at | Xrcc1 | X-ray repair complementing defective repair in Chinese hamster cells 1 | -3.872 | 6.37E-03 | 0.691 |
| *Nucleosome assembly* | | |  |  |  |
| 104045_at | Tspyl4 | TSPY-like 4 | -4.543 | 3.19E-03 | 0.561 |
| *Response to oxidative stress* | | |  |  |  |
| 160547_s_at | Txnip | Thioredoxin interacting protein | 4.509 | 3.30E-03 | 2.023* |
| *Autophagy* | | |  |  |  |
| 95138_at | Wipi2 | WD repeat domain, phosphoinositide interacting 2 | -3.555 | 9.31E-03 | 0.738 |
| *Chemotaxis* | | |  |  |  |
| 100704_at | Cmtm4 | CKLF-like MARVEL transmembrane domain containing 4 | 4.733 | 2.68E-03 | 1.231 |
| *Extracellular matrix organization and biogenesis* | | |  |  |  |
| 96835_at | Matn4 | Matrilin 4 | 3.670 | 8.12E-03 | 1.514 |
| *Regulation of blood pressure* | | |  |  |  |
| 95355_at | Agtrap | Angiotensin II, type I receptor-associated protein | -4.121 | 4.84E-03 | 0.613 |
| *Epithelial to mesenchymal transition* | | |  |  |  |
| 99183_at | Ppp3r1 | Protein phosphatase 3, regulatory subunit B, alpha isoform (calcineurin B, type I) | -3.588 | 8.97E-03 | 0.764 |
| *Muscle contraction* | | |  |  |  |
| 100605_at | Tpm2 | Tropomyosin 2, beta | 5.733 | 1.22E-03 | 1.665 |
| *Telomere maintenance* | | |  |  |  |
| 102912_at | Tnks2 | Tankyrase, TRF1-interacting ankyrin-related ADP-ribose polymerase 2 | -5.287 | 1.70E-03 | 0.403 |
| *Unknown* | | |  |  |  |
| 100471_at | 1110036O03Rik | RIKEN cDNA 1110036O03 gene | 13.106 | 7.78E-05 | 1.639 |
| 160773_at | D4Wsu132e | DNA segment, Chr 4, Wayne State University 132, expressed | 6.491 | 7.57E-04 | 1.751 |
| 94192_at | Gdap10 | Ganglioside-induced differentiation-associated-protein 10 | 6.381 | 8.21E-04 | 1.709 |
| 160682_at | 6430706D22Rik | RIKEN cDNA 6430706D22 gene | 5.969 | 1.06E-03 | 1.393 |
| 162260_at | 6330407G11Rik | RIKEN cDNA 6330407G11 gene | 4.853 | 2.41E-03 | 1.545 |
| 161742_r_at | NA | NA | 4.016 | 5.49E-03 | 1.250 |
| 94667_at | AA408396 | Expressed sequence AA408396 | 3.898 | 6.20E-03 | 1.157 |
| 99366_at | Pqlc3 | PQ loop repeat containing | 3.888 | 6.26E-03 | 2.410 |
| 100442_at | LOC100046166 | Similar to transforming growth factor beta regulated gene 4 | 3.882 | 6.30E-03 | 1.381 |
| 96614_at | 4933426M11Rik | RIKEN cDNA 4933426M11 gene | 3.873 | 6.35E-03 | 1.795 |
| 100322_at | NA | NA | 3.771 | 7.20E-03 | 1.374 |
| 161822_at | Svp2 | Seminal vesicle protein 2 | 3.739 | 7.50E-03 | 1.122 |
| 160563_at | Serf2 | Small EDRK-rich factor 2 | -8.656 | 2.84E-04 | 0.713 |
| 104434_at | Unc119b | Unc-119 homolog B (C. elegans) | -8.596 | 2.91E-04 | 0.674 |
| 97423_at | 1500035H01Rik | RIKEN cDNA 1500035H01 gene | -8.391 | 3.18E-04 | 0.638 |
| 94047_at | 0610031J06Rik | RIKEN cDNA 0610031J06 gene | -6.854 | 6.41E-04 | 0.637 |
| 97401_at | Josd1 | Josephin domain containing 1 | -6.404 | 8.03E-04 | 0.585 |
| 99139_at | 2310075A12Rik | RIKEN cDNA 2310075A12 gene | -6.239 | 8.79E-04 | 0.682 |
| 160685_at | Klhl7 | Kelch-like 7 (Drosophila) | -6.122 | 9.61E-04 | 0.692 |
| 104038_at | 4933434E20Rik | RIKEN cDNA 4933434E20 gene | -6.040 | 9.95E-04 | 0.612 |
| 95593_at | Golm1 | Golgi membrana protein 1 | -5.902 | 1.11E-03 | 0.733 |
| 95478_at | Deb1 | Differentially expressed in B16F10 1 | -5.887 | 1.12E-03 | 0.650 |
| 160904_at | B230317C12Rik | RIKEN cDNA B230317C12 gene | -5.865 | 1.14E-03 | 0.631 |
| 96917_at | 2410166I05Rik | RIKEN cDNA 2410166I05 gene | -5.803 | 1.18E-03 | 0.712 |
| 94106_at | NA | NA | -5.485 | 1.44E-03 | 0.436 |
| 103545_at | 2610019E17Rik | RIKEN cDNA 2610019E17 gene | -5.455 | 1.48E-03 | 0.587 |
| 161214_r_at | BC037034 | cDNA sequence BC037034 | -5.295 | 1.69E-03 | 0.589 |
| 95045_at | 0610012D09Rik | RIKEN cDNA 0610012D09 gene | -5.047 | 2.07E-03 | 0.524 |
| 92268_at | 2700007P21Rik | RIKEN cDNA 2700007P21 gene | -4.930 | 2.27E-03 | 0.667 |
| 93059_at | 2610204K14Rik | RIKEN cDNA 2610204K14 gene | -4.781 | 2.57E-03 | 0.590 |
| 100465_i_at | Iqcf4 | IQ motif containing F4 | -4.760 | 2.61E-03 | 0.340 |
| 98908_at | Usmg3 | Upregulated during skeletal muscle growth 3 | -4.747 | 2.65E-03 | 0.490 |
| 102233_at | 4833442J19Rik | RIKEN cDNA 4833442J19 gene | -4.725 | 2.69E-03 | 0.648 |
| 102137_f_at | NA | NA | -4.707 | 2.74E-03 | 0.728 |
| 104228_at | NA | NA | -4.677 | 2.85E-03 | 0.717 |
| 100054_s_at | D2Wsu81e | DNA segment, Chr 2, Wayne State University 81, expressed | -4.618 | 2.98E-03 | 0.748 |
| 96686_i_at | 2010100O12Rik | RIKEN cDNA 2010100O12 gene | -4.515 | 3.28E-03 | 0.536 |
| 93165_at | Lysmd1 | LysM, putative peptidoglycan-binding, domain containing 1 | -4.458 | 3.47E-03 | 0.746 |
| 97897_at | C78339 | Expressed sequence C78339 | -4.333 | 3.91E-03 | 0.538 |
| 97115_at | NA | NA | -4.242 | 4.29E-03 | 0.721 |
| 160709_at | 1110001A16Rik | RIKEN cDNA 1110001A16 gene | -4.235 | 4.32E-03 | 0.800 |
| 98039_at | 2410015M20Rik | RIKEN cDNA 2410015M20 gene | -4.184 | 4.52E-03 | 0.703 |
| 95002_at | D17Wsu92e | DNA segment, Chr 17, Wayne State University 92, expressed | -4.127 | 4.82E-03 | 0.580 |
| 160549_at | Tmem165 | Transmembrane protein 165 | -4.088 | 5.02E-03 | 0.705 |
| 103780_at | 1700021F05Rik | RIKEN cDNA 1700021F05 gene | -4.072 | 5.12E-03 | 0.740 |
| 161770_f_at | Ankzf1 | Ankyrin repeat and zinc finger domain containing 1 | -4.065 | 5.15E-03 | 0.729 |
| 100742_at | AA409749 | Expressed sequence AA409749 | -3.916 | 6.09E-03 | 0.771 |
| 96240_at | Tmem106c | Transmembrane protein 106C | -3.883 | 6.30E-03 | 0.588 |
| 104076_at | 1190017O12Rik | RIKEN cDNA 1190017O12 gene | -3.844 | 6.60E-03 | 0.751 |
| 97935_at | LOC100047441 | Similar to RIKEN cDNA 4121402D02 gene | -3.820 | 6.77E-03 | 0.739 |
| 100039_at | Tmem4 | Transmembrane protein 4 | -3.810 | 6.86E-03 | 0.649* |
| 96090_g_at | 4931406C07Rik | RIKEN cDNA 4931406C07 gene | -3.779 | 7.11E-03 | 0.514 |
| 104195_at | 1810009A15Rik | RIKEN cDNA 1810009A15 gene | -3.759 | 7.33E-03 | 0.771 |
| 96340_at | Tmem50b | Transmembrane protein 50B | -3.758 | 7.36E-03 | 0.793 |
| 94955_at | Ankrd40 | Ankyrin repeat domain 40 | -3.717 | 7.72E-03 | 0.748 |
| 100466_f_at | Iqcf4 | IQ motif containing F4 | -3.714 | 7.73E-03 | 0.528 |
| 103584_at | 5830471E12Rik | RIKEN cDNA 5830471E12 gene | -3.679 | 8.04E-03 | 0.457 |
| 95984_at | C79468 | Expressed sequence C79468 | -3.606 | 8.77E-03 | 0.804 |
| 160704_at | 1110067D22Rik | RIKEN cDNA 1110067D22 gene | -3.590 | 8.93E-03 | 0.306 |
| 95396_at | NA | DNA cytosine methyltransferase mRNA | -3.558 | 9.28E-03 | 0.187 |
| 102141_f_at | 4933434E20Rik | RIKEN cDNA 4933434E20 gene | -3.525 | 9.67E-03 | 0.572 |
| 101023_f_at | 0610010E21Rik | RIKEN cDNA 0610010E21 gene | -3.523 | 9.69E-03 | 0.770 |

**Table S6. Differential gene expression in serum-starved, G0-arrested H-*ras*^-/-^/N-*ras*^-/-^ fibroblasts after incubation of cell cultures in the presence of serum for 1 hour.** List of 593 differentially expressed probesets (570 different genes) identified by means of SAM contrast (FDR=0,09) comparing the microarray-generated transcriptional profile of WT fibroblasts that had been serum-starved for 24h to that of similarly starved, H-*ras*^-/-^/N-*ras*^-/-^ fibroblast cultures submitted to subsequent incubation in the presence of 20% FBS for 1 hour. To concentrate on loci exclusively regulated by Ras, genes sharing similar values of differential expression between the WT and H-*ras*^-/-^/N-*ras*^-/-^ fibroblast (ratio of the R-fold values in their respective lists within the range 0.6-1.5) were excluded from this list. The differentially expressed loci are identified by Affymetrix probeset ID, gene name and symbol, and listed according to functional category and degree of overexpression or repression, quantitated by d(i) value. The * and # symbols in the R.fold column denote independent validation of the transcriptional data obtained by means of quantitative RT-PCR or Western immunoblot, respectively. d(i) is a parameter measuring the statistical distance separating the calculated expression value of each gene probeset from the null hypothesis (no-change). p-value is an statistical measure indicating the probability of random expression for that probeset. R fold is the log2 value of the fold change measuring the overexpression or repression of the probesets in the collection of microarrays.

| **Probeset ID** | **Genename** | **Description** | **d(i)** | **p-value** | **R.fold** |
| --- | --- | --- | --- | --- | --- |
| *Signal transduction* | |  |  |  |  |
| 101030_at | Rhob | Ras homolog gene family, member B | 15.134 | 5.26E-05 | 6.408 |
| 101561_at | Mt2 | Metallothionein 2 | 9.325 | 2.10E-04 | 4.211* |
| 161609_at | Rgs16 | Regulator of G-protein signaling 16 | 9.249 | 2.13E-04 | 9.392* |
| 94378_at | Rgs16 | Regulator of G-protein signaling 16 | 8.408 | 2.88E-04 | 10.940* |
| 104152_at | Stk40 | Serine/threonine kinase 40 | 7.216 | 4.76E-04 | 1.573 |
| 97890_at | Sgk | Serum/glucocorticoid regulated kinase | 7.154 | 5.01E-04 | 8.665* |
| 104697_at | Rhoj | Ras homolog gene family, member J | 7.074 | 5.19E-04 | 1.999 |
| 92362_at | Dusp8 | Dual specificity phosphatase 8 | 6.486 | 7.34E-04 | 2.282 |
| 93573_at | Mt1 | Metallothionein 1 | 6.418 | 7.71E-04 | 4.380 |
| 93974_at | Errfi1 | ERBB receptor feedback inhibitor 1 | 6.344 | 8.05E-04 | 10.626 |
| 101502_at | Tgif | TG interacting factor | 5.779 | 1.26E-03 | 2.792* |
| 97979_at | Ppp1r7 | Protein phosphatase 1, regulatory (inhibitor) subunit 7 | 5.773 | 1.27E-03 | 1.362 |
| 100555_at | Rcan1 | 40ouse40a40s of calcineurin 1 | 5.675 | 1.34E-03 | 2.398* |
| 98916_at | Ppp2r2a | Protein phosphatase 2 (formerly 2A), regulatory subunit B (PR 52), alpha isoform | 5.239 | 1.91E-03 | 1.387 |
| 95547_at | Rhod | Ras homolog gene family, member D | 5.020 | 2.35E-03 | 1.512 |
| 93975_at | Errfi1 | ERBB receptor feedback inhibitor 1 | 5.008 | 2.39E-03 | 8.637 |
| 95721_at | Mapkapk2 | MAP kinase-activated protein kinase 2 | 4.894 | 2.65E-03 | 1.777 |
| 101435_at | Akap2 | A kinase (PRKA) anchor protein 2 | 4.841 | 2.78E-03 | 1.680 |
| 160779_at | Saps3 | SAPS domain family, member 3 | 4.795 | 2.91E-03 | 1.390 |
| 99960_at | Map2k4 | Mitogen activated protein kinase kinase 4 | 4.663 | 3.27E-03 | 1.802 |
| 95609_at | Ppp1r15b | Protein phosphatase 1, regulatory (inhibitor) subunit 15b | 4.620 | 3.42E-03 | 2.000 |
| 104498_at | Homer1 | Homer homolog 1 (Drosophila) | 4.491 | 3.81E-03 | 2.567 |
| 104673_at | Epha4 | Eph receptor A4 | 4.331 | 4.50E-03 | 5.336 |
| 93314_g_at | Map2k3 | Mitogen activated protein kinase kinase 3 | 4.250 | 4.82E-03 | 1.408 |
| 104180_at | Rac3 | RAS-related C3 botulinum substrate 3 | 4.209 | 5.00E-03 | 1.318 |
| 99337_at | Baiap2 | Brain-specific angiogenesis inhibitor 1-associated protein 2 | 4.171 | 5.21E-03 | 1.659 |
| 99532_at | Tob1 | Transducer of erbb-2.1 | 4.165 | 5.24E-03 | 2.488 |
| 160082_s_at | Arf4 | ADP-ribosylation factor 4 | 4.159 | 5.28E-03 | 1.935 |
| 93676_at | Rad51ap1 | RAD51 associated protein 1 | 4.106 | 5.58E-03 | 1.439 |
| 100155_at | Ddr1 | Discoidin domain receptor family, member 1 | 4.092 | 5.64E-03 | 1.700 |
| 97991_at | Kras | v-Ki-ras2 Kirsten rat sarcoma viral oncogene homolog | 4.039 | 5.91E-03 | 1.715 |
| 93285_at | Dusp6 | Dual specificity phosphatase 6 | 4.030 | 5.96E-03 | 4.426 |
| 99176_at | Fibp | Fibroblast growth factor (acidic) intracellular binding protein | 3.975 | 6.29E-03 | 1.557 |
| 160840_at | Arhgef3 | Rho guanine nucleotide exchange factor (GEF) 3 | 3.910 | 6.77E-03 | 1.535 |
| 101571_g_at | Igfbp4 | Insulin-like growth factor binding protein 4 | 3.825 | 7.41E-03 | 2.588 |
| 161171_at | Dusp8 | Dual specificity phosphatase 8 | 3.767 | 7.91E-03 | 1.396 |
| 102759_at | Pik3r2 | Phosphatidylinositol 3-kinase, regulatory subunit, polypeptide 2 (p85 beta) | -14.921 | 5.49E-05 | 0.532* |
| 160925_at | Nras | Neuroblastoma ras oncogene | -8.566 | 2.72E-04 | 0.386 |
| 97058_f_at | Rab33b | RAB33B, member of RAS oncogene family | -8.552 | 2.75E-04 | 0.731 |
| 94362_at | Nras | Neuroblastoma ras oncogene | -8.199 | 3.09E-04 | 0.185 |
| 104461_at | Pik3ca | Phosphatidylinositol 3-kinase, catalytic, alpha polypeptide | -6.973 | 5.58E-04 | 0.536 |
| 95136_at | Arl6ip4 | ADP-ribosylation factor-like 6 interacting protein 4 | -6.789 | 5.90E-04 | 0.640 |
| 96132_at | Apcdd1 | Adenomatosis polyposis coli down-regulated 1 | -6.657 | 6.34E-04 | 0.321 |
| 96728_at | Wdr45 | WD repeat domain 45 | -6.526 | 7.09E-04 | 0.566 |
| 94258_at | Arhgdib | Rho, GDP dissociation inhibitor (GDI) beta | -6.475 | 7.46E-04 | 0.334 |
| 100976_at | Ptpn9 | Protein tyrosine phosphatase, non-receptor type 9 | -6.366 | 8.01E-04 | 0.623 |
| 102374_at | Rcan3 | 41ouse41a41s of calcineurin 3 | -6.305 | 8.42E-04 | 0.622 |
| 102308_at | Tulp3 | Tubby-like protein 3 | -6.206 | 8.81E-04 | 0.712 |
| 94403_at | Tbc1d14 | TBC1 domain family, member 14 | -5.994 | 1.05E-03 | 0.535 |
| 93826_at | Ppp2r5a | Protein phosphatase 2, regulatory subunit B (B56), alpha isoform | -5.872 | 1.17E-03 | 0.277* |
| 93939_at | Sh2b3 | SH2B adaptor protein 3, similar to lymphocyte-specific adaptor protein Lnk | -5.749 | 1.29E-03 | 0.642 |
| 103833_at | Hipk2 | Homeodomain interacting protein kinase 2 | -5.684 | 1.33E-03 | 0.688 |
| 160892_at | Dlgh3 | Discs, large homolog 3 (Drosophila) | -5.619 | 1.40E-03 | 0.751 |
| 92585_at | Map2k1 | Mitogen activated protein kinase kinase 1 | -5.555 | 1.47E-03 | 0.585 |
| 98927_at | Rab6 | RAB6, member RAS oncogene family | -5.378 | 1.70E-03 | 0.707 |
| 104525_at | Ppm1m | Protein phosphatase 1M | -5.345 | 1.74E-03 | 0.649 |
| 102710_at | Apbb1ip | Amyloid beta (A4) precursor protein-binding, family B, member 1 interacting protein | -5.307 | 1.83E-03 | 0.206 |
| 94394_at | Rras | Harvey rat sarcoma oncogene, subgroup R | -5.088 | 2.22E-03 | 0.553 |
| 100122_at | Gnb5 | Guanine nucleotide binding protein, beta 5 | -4.954 | 2.49E-03 | 0.593 |
| 103592_at | Map2k5 | Mitogen activated protein kinase kinase 5 | -4.907 | 2.60E-03 | 0.665 |
| 96751_at | Arl5 | ADP-ribosylation factor-like 5 | -4.815 | 2.85E-03 | 0.403 |
| 103330_at | Rabgap1 | RAB GTPase activating protein 1 | -4.775 | 2.97E-03 | 0.562 |
| 160708_at | Schip1 | Schwannomin interacting protein 1 | -4.709 | 3.16E-03 | 0.439 |
| 100958_at | Spata13 | Spermatogenesis associated 13 | -4.698 | 3.19E-03 | 0.687 |
| 101584_at | Rsu1 | Ras 41ouse41a41 protein 1 | -4.689 | 3.21E-03 | 0.795 |
| 94398_s_at | Inpp5b | Inositol polyphosphate-5-phosphatase B | -4.688 | 3.21E-03 | 0.744 |
| 162092_f_at | Ihpk1 | Inositol hexaphosphate kinase 1 | -4.657 | 3.28E-03 | 0.803 |
| 160527_at | Igfbp7 | Insulin-like growth factor binding protein 7 | -4.634 | 3.37E-03 | 0.585 |
| 99953_at | Rgl2 | Ral guanine nucleotide dissociation stimulator-like 2 | -4.486 | 3.83E-03 | 0.760 |
| 96572_at | Azi2 | 5-azacytidine induced gene 2 | -4.434 | 4.05E-03 | 0.607 |
| 92452_at | Pik3ca | Phosphatidylinositol 3-kinase, catalytic, alpha polypeptide | -4.405 | 4.16E-03 | 0.606 |
| 161521_at | Ppp1r12c | Protein phosphatase 1, regulatory (inhibitor) subunit 12C | -4.364 | 4.34E-03 | 0.819 |
| 104302_f_at | Commd9 | COMM domain containing 9 | -4.309 | 4.59E-03 | 0.644 |
| 94006_at | Azi2 | 5-azacytidine induced gene 2 | -4.304 | 4.61E-03 | 0.530 |
| 96818_at | Dtx2 | Deltex 2 homolog (Drosophila) | -4.295 | 4.64E-03 | 0.716 |
| 99596_f_at | Gnai2 | Guanine nucleotide binding protein, alpha inhibiting 2 | -4.210 | 4.99E-03 | 0.673 |
| 94801_at | Pgrmc2 | 42ouse42a42s42na receptor 42ouse42a42 component 2 | -4.189 | 5.11E-03 | 0.568 |
| 96911_at | Gnb2 | Guanine nucleotide binding protein, beta 2 | -4.175 | 5.18E-03 | 0.694 |
| 92698_at | Mertk | C-mer proto-oncogene tyrosine kinase | -4.174 | 5.19E-03 | 0.747 |
| 97505_at | Arl1 | ADP-ribosylation factor-like 1 | -4.171 | 5.21E-03 | 0.634 |
| 93627_at | Ankrd28 | Ankyrin repeat domain 28 | -4.152 | 5.33E-03 | 0.395 |
| 102196_at | Gna11 | Guanine nucleotide binding protein, alpha 11 | -4.132 | 5.44E-03 | 0.558* |
| 103209_at | Ltbp1 | Latent transforming growth factor beta binding protein 1 | -4.126 | 5.48E-03 | 0.529* |
| 95701_at | Cxxc5 | CXXC finger 5 | -4.064 | 5.77E-03 | 0.382 |
| 101834_at | Mapk3 | Mitogen activated protein kinase 3 | -4.054 | 5.82E-03 | 0.705* |
| 100908_at | Ptpra | Protein tyrosine phosphatase, receptor type, A | -4.017 | 6.04E-03 | 0.531 |
| 101442_f_at | Itpr2 | Inositol 1,4,5-triphosphate receptor 2 | -3.962 | 6.40E-03 | 0.681 |
| 160536_at | Hras1 | Harvey rat sarcoma virus oncogene 1 | -3.946 | 6.49E-03 | 0.689 |
| 104607_at | Rtn2 | Reticulon 2 (Z-band associated protein) | -3.864 | 7.10E-03 | 0.550 |
| 97226_at | Gna12 | Guanine nucleotide binding protein, alpha 12 | -3.855 | 7.16E-03 | 0.470 |
| 161067_at | Trib3 | 42ouse42a42 homolog 3 (Drosophila) | -3.825 | 7.41E-03 | 0.455 |
| 104725_at | Rhoq | Ras homolog gene family, member Q | -3.815 | 7.52E-03 | 0.528 |
| *Transcription* | |  |  |  |  |
| 102661_at | Egr2 | Early growth response 2 | 44.994 | 4.58E-06 | 56.783 |
| 94010_at | Tceb1 | Transcription elongation factor B (SIII), polypeptide 1 | 27.007 | 1.60E-05 | 2.935 |
| 94009_at | Tceb1 | Transcription elongation factor B (SIII), polypeptide 1 | 19.680 | 3.20E-05 | 2.874 |
| 99602_at | Klf10 | Kruppel-like factor 10 | 13.263 | 6.86E-05 | 10.980 |
| 99603_g_at | Klf10 | Kruppel-like factor 10 | 13.058 | 7.55E-05 | 10.505 |
| 99835_at | Fosl1 | Fos-like antigen 1 | 12.398 | 8.47E-05 | 18.120* |
| 99535_at | Ccrn4l | CCR4 carbon catabolite repression 4-like (S. cerevisiae) | 12.186 | 9.84E-05 | 14.836 |
| 94064_at | Zfp91 | Zinc finger protein 91 | 11.496 | 1.21E-04 | 1.652 |
| 92925_at | Cebpb | CCAAT/enhancer binding protein (C/EBP), beta | 10.368 | 1.56E-04 | 3.896* |
| 98083_at | Klf6 | Kruppel-like factor 6 | 10.346 | 1.58E-04 | 6.893 |
| 94008_at | Tceb1 | Transcription elongation factor B (SIII), polypeptide 1 | 9.125 | 2.27E-04 | 4.80 |
| 96109_at | Klf2 | Kruppel-like factor 2 (lung) | 8.353 | 2.93E-04 | 2.499 |
| 94109_at | Zfp281 | Zinc finger protein 281 | 7.819 | 3.68E-04 | 2.443 |
| 104156_r_at | Atf3 | Activating transcription factor 3 | 6.655 | 6.36E-04 | 18.640 |
| 160894_at | Cebpd | CCAAT/enhancer binding protein (C/EBP), delta | 6.402 | 7.85E-04 | 3.363 |
| 92923_g_at | Tcfap2a | Transcription factor AP-2, alpha | 6.388 | 7.89E-04 | 1.246 |
| 103393_at | Pspc1 | Paraspeckle protein 1 | 6.073 | 9.91E-04 | 1.975 |
| 103318_at | Gabpb1 | GA repeat binding protein, beta 1 | 6.067 | 1.00E-03 | 1.654 |
| 95360_at | Zcchc6 | Zinc finger, CCHC domain containing 6 | 5.468 | 1.57E-03 | 1.989 |
| 104712_at | Myc | Myelocytomatosis oncogene | 5.463 | 1.58E-03 | 7.941* |
| 104645_at | Klf7 | Kruppel-like factor 7 (ubiquitous) | 5.304 | 1.83E-03 | 3.462* |
| 93324_at | Zfp36l1 | Zinc finger protein 36, C3H type-like 1 | 5.281 | 1.86E-03 | 2.839* |
| 92300_at | Mnt | Max binding protein | 4.872 | 2.70E-03 | 1.870 |
| 93619_at | Per1 | Period homolog 1 (Drosophila) | 4.770 | 2.99E-03 | 5.523* |
| 97386_at | Zfp703 | Zinc finger protein 703 | 4.522 | 3.71E-03 | 1.926 |
| 160834_at | Sertad1 | SERTA domain containing 1 | 4.499 | 3.77E-03 | 3.383 |
| 101727_at | Nfkbie | Nuclear factor of kappa light polypeptide gene enhancer in B-cells inhibitor, epsilon | 4.274 | 4.73E-03 | 2.050 |
| 161716_at | Fos | FBJ osteosarcoma oncogene | 4.166 | 5.23E-03 | 1.311 |
| 92339_at | Taf1a | TATA box binding protein (Tbp)-associated factor, RNA polymerase I, A | 4.125 | 5.48E-03 | 1.566* |
| 100125_at | Pa2g4 | Proliferation-associated 2G4 | 3.992 | 6.20E-03 | 1.509* |
| 104701_at | Bhlhb2 | Basic helix-loop-helix domain containing, class B2 | 3.990 | 6.20E-03 | 5.590* |
| 102209_at | Nfatc1 | Nuclear factor of activated T-cells, cytoplasmic, calcineurin-dependent 1 | 3.887 | 6.94E-03 | 1.286* |
| 103891_i_at | Ell2 | Elongation factor RNA polymerase II 2 | 3.878 | 7.00E-03 | 3.047 |
| 92564_at | Lrrfip1 | Leucine rich repeat (in FLII) interacting protein 1 | 3.838 | 7.30E-03 | 2.036 |
| 93490_at | Maf1 | MAF1 homolog (S. cerevisiae) | -12.127 | 1.01E-04 | 0.397 |
| 97893_at | Tbpl1 | TATA box binding protein-like 1 | -11.626 | 1.14E-04 | 0.532 |
| 99024_at | Mxd4 | Max dimerization protein 4 | -10.676 | 1.46E-04 | 0.419 |
| 100475_at | Trim25 | Tripartite motif protein 25 | -8.085 | 3.23E-04 | 0.165 |
| 98790_s_at | Meis1 | Myeloid ecotropic viral integration site 1 | -7.902 | 3.48E-04 | 0.555 |
| 93656_g_at | Usf1 | Upstream transcription factor 1 | -6.745 | 6.02E-04 | 0.712 |
| 101081_at | Ctbp1 | C-terminal binding protein 1 | -6.334 | 8.17E-04 | 0.532 |
| 160449_at | Dr1 | Down-regulator of transcription 1 | -6.231 | 8.69E-04 | 0.641* |
| 95161_at | Ctdsp2 | CTD (carboxy-terminal domain, RNA polymerase II, polypeptide A) small phosphatase 2 | -6.203 | 8.88E-04 | 0.377 |
| 98070_at | Suds3 | Suppressor of defective silencing 3 homolog (S. cerevisiae) | -5.935 | 1.11E-03 | 0.692 |
| 103466_at | Cyhr1 | Cysteine and 43ouse43a43s rich 1 | -5.928 | 1.12E-03 | 0.570 |
| 100461_at | Polr2j | Polymerase (RNA) II (DNA directed) polypeptide J | -5.888 | 1.16E-03 | 0.585 |
| 160245_at | Zfp740 | Zinc finger protein 740 | -5.829 | 1.21E-03 | 0.532 |
| 161077_f_at | Smarcd2 | SWI/SNF related, matrix associated, actin dependent regulator of chromatin, subfamily d, member 2 | -5.098 | 2.19E-03 | 0.516 |
| 94295_at | Gtf2i | General transcription factor II I | -5.040 | 2.32E-03 | 0.567 |
| 98981_s_at | Tcf12 | Transcription factor 12 | -4.993 | 2.42E-03 | 0.378 |
| 96940_at | Tead2 | TEA domain family member 2 | -4.844 | 2.76E-03 | 0.697 |
| 95883_at | Phf17 | PHD finger protein 17 | -4.816 | 2.84E-03 | 0.536 |
| 103006_at | Atf5 | Activating transcription factor 5 | -4.809 | 2.87E-03 | 0.448 |
| 160138_at | Mxi1 | Max interacting protein 1 | -4.733 | 3.10E-03 | 0.386 |
| 103467_g_at | Cyhr1 | Cysteine and 43ouse43a43s rich 1 | -4.628 | 3.40E-03 | 0.402 |
| 102833_at | Cbx2 | Chromobox homolog 2 (Drosophila Pc class) | -4.465 | 3.91E-03 | 0.688 |
| 101939_at | Rnf141 | Ring finger protein 141 | -4.380 | 4.26E-03 | 0.800 |
| 103762_at | Gtf2f1 | General transcription factor IIF, polypeptide 1 | -4.349 | 4.42E-03 | 0.779 |
| 92344_at | Hltf | Helicase-like transcription factor | -4.172 | 5.20E-03 | 0.611 |
| 161080_f_at | Gtf2ird2 | GTF2I repeat domain containing 2 | -4.096 | 5.62E-03 | 0.649 |
| 96793_at | Dmap1 | DNA methyltransferase 1-associated protein 1 | -4.087 | 5.66E-03 | 0.748 |
| 95315_at | Zfp14 | Zinc finger protein 14 | -4.033 | 5.95E-03 | 0.820 |
| 160220_at | Zfp110 | Zinc finger protein 110 | -4.005 | 6.12E-03 | 0.673 |
| 95522_i_at | Zfp68 | Zinc finger protein 68 | -3.986 | 6.23E-03 | 0.510* |
| 93615_at | Pbx3 | Pre B-cell 43ouse43a43 transcription factor 3 | -3.844 | 7.25E-03 | 0.604 |
| 102256_at | Tbx15 | T-box 15 | -3.830 | 7.36E-03 | 0.309 |
| 104437_at | Zfp30 | Zinc finger protein 30 | -3.805 | 7.60E-03 | 0.469 |
| 162138_s_at | Cbx6 | Chromobox homolog 6 | -3.804 | 7.61E-03 | 0.309 |
| *Primary cell metabolism* | |  |  |  |  |
| 94177_at | Hsd17b7 | Hydroxysteroid (17-beta) dehydrogenase 7 | 11.457 | 1.24E-04 | 2.627 |
| 160580_at | Man1a | Mannosidase 1, alpha | 10.095 | 1.65E-04 | 4.735* |
| 104647_at | Ptgs2 | Prostaglandin-endoperoxide synthase 2 | 7.741 | 3.80E-04 | 35.217* |
| 94367_at | Uck2 | Uridine-cytidine kinase 2 | 6.499 | 7.14E-04 | 2.370* |
| 101990_at | Ldh2 | Lactate dehydrogenase 2, B chain | 5.493 | 1.53E-03 | 2.396 |
| 92731_at | Ptx3 | Pentraxin related gene | 5.177 | 2.02E-03 | 3.452* |
| 160107_at | Hprt1 | Hypoxanthine guanine phosphoribosyl transferase 1 | 5.146 | 2.08E-03 | 2.074* |
| 103271_at | Lrp6 | Low density lipoprotein receptor-related protein 6 | 5.090 | 2.21E-03 | 1.322 |
| 160362_at | Mat2a | Methionine adenosyltransferase II, alpha | 4.978 | 2.44E-03 | 1.330 |
| 94216_at | Sdhc | Succinate dehydrogenase complex, subunit C, integral membrane protein | 4.918 | 2.58E-03 | 1.367 |
| 160579_at | Man1a | Mannosidase 1, alpha | 4.821 | 2.83E-03 | 2.862* |
| 98892_at | Lpin1 | Lipin 1 | 4.559 | 3.58E-03 | 2.357* |
| 160648_at | Fignl1 | Fidgetin-like 1 | 4.436 | 4.05E-03 | 1.904 |
| 98632_at | Ada | Adenosine deaminase | 4.229 | 4.91E-03 | 1.559 |
| 98989_at | Dhcr7 | 7-dehydrocholesterol reductase | 4.068 | 5.75E-03 | 2.169 |
| 103341_at | Ctps | Cytidine 5’-triphosphate synthase | 4.060 | 5.79E-03 | 1.643 |
| 96231_at | Bphl | Biphenyl hydrolase-like (serine hydrolase, breast epithelial mucin-associated antigen) | 4.042 | 5.89E-03 | 1.301 |
| 93264_at | Srebf1 | Sterol regulatory element binding factor 1 | 3.990 | 6.21E-03 | 1.216 |
| 104285_at | Hmgcr | 3-hydroxy-3-methylglutaryl-Coenzyme A reductase | 3.932 | 6.58E-03 | 2.893 |
| 102352_at | Galnt10 | UDP-N-acetyl-alpha-D-galactosamine:polypeptide N-acetylgalactosaminyltransferase 10 | 3.807 | 7.59E-03 | 1.411 |
| 93496_at | Elovl5 | ELOVL family member 5, elongation of long chain fatty acids (yeast) | 3.796 | 7.68E-03 | 1.440 |
| 102049_at | Pdk4 | Pyruvate dehydrogenase kinase, isoenzyme 4 | 3.781 | 7.80E-03 | 2.127 |
| 160388_at | Sc4mol | Sterol-C4-methyl oxidase-like | 3.774 | 7.85E-03 | 2.459 |
| 97398_at | Pck2 | Phosphoenolpyruvate carboxykinase 2 (mitochondrial) | -9.846 | 1.78E-04 | 0.433 |
| 104372_at | Abhd8 | Abhydrolase domain containing 8 | -8.647 | 2.63E-04 | 0.421 |
| 92887_at | Ddah2 | Dimethylarginine dimethylaminohydrolase 2 | -7.365 | 4.44E-04 | 0.561 |
| 101482_at | Ppp1cc | Protein phosphatase 1, catalytic subunit, gamma isoform | -6.343 | 8.08E-04 | 0.532 |
| 101473_at | Nnmt | Nicotinamide N-methyltransferase | -6.153 | 9.24E-04 | 0.474 |
| 98960_s_at | B3galt3 | UDP-Gal:betaGlcNAc beta 1,3-galactosyltransferase, polypeptide 3 | -6.097 | 9.70E-04 | 0.560 |
| 97343_at | Mepce | Methylphosphate 44ouse44a enzyme | -5.970 | 1.08E-03 | 0.725 |
| 93320_at | Cpt1a | Carnitine palmitoyltransferase 1ª, liver | -5.907 | 1.14E-03 | 0.405 |
| 93940_at | Pon3 | Paraoxonase 3 | -5.356 | 1.72E-03 | 0.570 |
| 104677_at | Man1b1 | Mannosidase, alpha, class 1B, member 1 | -5.259 | 1.88E-03 | 0.584 |
| 101000_at | Oaz2 | Ornithine decarboxylase antizyme 2 | -5.233 | 1.93E-03 | 0.453 |
| 160482_at | Acaa1 | Acetyl-Coenzyme A acyltransferase 1 | -5.153 | 2.07E-03 | 0.548 |
| 96338_at | Egln2 | EGL nine homolog 2 (C. elegans) | -5.094 | 2.20E-03 | 0.661 |
| 101074_at | Ddost | Dolichyl-di-phosphooligosaccharide-protein glycotransferase | -5.017 | 2.36E-03 | 0.537 |
| 161243_f_at | Abhd8 | Abhydrolase domain containing 8 | -4.952 | 2.50E-03 | 0.526 |
| 102936_at | B4galt6 | UDP-Gal:betaGlcNAc beta 1,4-galactosyltransferase, polypeptide 6 | -4.730 | 3.11E-03 | 0.642 |
| 92263_at | Leprel2 | 44ouse44a44-like 2 | -4.655 | 3.29E-03 | 0.685 |
| 94381_at | Uck1 | Uridine-cytidine kinase 1 | -4.648 | 3.33E-03 | 0.758 |
| 103739_at | MGI:2136405 | Glucuronyl C5-epimerase | -4.552 | 3.60E-03 | 0.438 |
| 103646_at | Crat | Carnitine acetyltransferase | -4.440 | 4.02E-03 | 0.715 |
| 99847_at | St3gal1 | ST3 beta-galactoside alpha-2,3-sialyltransferase 1 | -4.285 | 4.68E-03 | 0.475 |
| 95133_at | Asns | Asparagine synthetase | -4.234 | 4.89E-03 | 0.593 |
| 93978_at | B3galnt2 | UDP-GalNAc:betaGlcNAc beta 1,3-galactosaminyltransferase, polypeptide 2 | -4.164 | 5.24E-03 | 0.774 |
| 99571_at | Acaa1 | Acetyl-Coenzyme A acyltransferase 1 | -4.134 | 5.43E-03 | 0.747 |
| 99184_at | Csad | Cysteine sulfinic acid decarboxylase | -4.105 | 5.58E-03 | 0.605* |
| 104342_i_at | Pla2g12a | Phospholipase A2, group XIIA | -4.014 | 6.06E-03 | 0.591 |
| 161694_f_at | Ptgs1 | Prostaglandin-endoperoxide synthase 1 | -4.007 | 6.10E-03 | 0.634 |
| 103637_at | Naga | N-acetyl galactosaminidase, alpha | -3.853 | 7.17E-03 | 0.550 |
| 95468_at | Egln1 | EGL nine homolog 1 (C. elegans) | -3.834 | 7.32E-03 | 0.730 |
| 102035_at | Tpmt | Thiopurine methyltransferase | -3.784 | 7.77E-03 | 0.792 |
| *Transport and trafficking processes* | | |  |  |  |
| 93466_at | Exoc4 | Exocyst complex component 4 | 8.602 | 2.68E-04 | 2.139 |
| 96930_at | Ehd1 | EH-domain containing 1 | 6.916 | 5.70E-04 | 1.621* |
| 95978_at | Atp13a3 | ATPase type 13A3 | 6.755 | 5.97E-04 | 2.870 |
| 103218_at | Slc10a3 | Solute carrier family 10 (sodium/bile acid cotransporter family), member 3 | 5.536 | 1.49E-03 | 1.486 |
| 94860_at | Timm17a | Translocator of inner mitochondrial membrane 17a | 4.958 | 2.48E-03 | 1.612 |
| 93738_at | Slc2a1 | Solute carrier family 2 (facilitated glucose transporter), member 1 | 4.902 | 2.62E-03 | 2.871 |
| 103667_at | Atp13a3 | ATPase type 13A3 | 4.793 | 2.91E-03 | 2.565 |
| 98944_at | Sec23b | Sec23b (s. Cerevisiae) | 4.174 | 5.19E-03 | 1.291 |
| 103988_at | Atg2a | ATG2 autophagy related 2 homolog A (S. cerevisiae) | 4.155 | 5.31E-03 | 1.135 |
| 95735_at | Nolc1 | Nucleolar and coiled-body phosphoprotein 1 | 4.112 | 5.54E-03 | 1.599 |
| 101370_at | Kpna1 | Karyopherin (importin) alpha 1 | 4.056 | 5.81E-03 | 1.817 |
| 103642_at | G3bp1 | Ras-GTPase-activating protein SH3-domain binding protein | 3.949 | 6.48E-03 | 1.998 |
| 93471_at | Slc4a7 | Solute carrier family 4, sodium bicarbonate cotransporter, member 7 | 3.942 | 6.52E-03 | 2.338 |
| 95733_at | Slc29a1 | Solute carrier family 29 (nucleoside transporters), member 1 | 3.890 | 6.91E-03 | 1.485 |
| 100943_at | Slc1a4 | Solute carrier family 1 (glutamate/neutral amino acid transporter), member 4 | -7.342 | 4.48E-04 | 0.579 |
| 93819_at | Tmed2 | Transmembrane emp24 domain trafficking protein 2 | -6.539 | 6.96E-04 | 0.562 |
| 104464_s_at | Kdelr3 | KDEL (Lys-Asp-Glu-Leu) endoplasmic reticulum protein retention receptor 3 | -6.336 | 8.12E-04 | 0.336 |
| 96894_at | Tmed4 | Transmembrane emp24 protein transport domain containing 4 | -6.213 | 8.76E-04 | 0.734 |
| 93045_at | Abcd3 | ATP-binding cassette, sub-family D (ALD), member 3 | -5.943 | 1.10E-03 | 0.444 |
| 93362_at | Ap2m1 | Adaptor protein complex AP-2, mu1 | -5.604 | 1.42E-03 | 0.579 |
| 95095_at | Flot1 | Flotillin 1 | -5.506 | 1.51E-03 | 0.636* |
| 102835_at | Ap2a2 | Adaptor protein complex AP-2, alpha 2 subunit | -5.236 | 1.92E-03 | 0.604 |
| 160344_at | Npc2 | Niemann Pick type C2 | -5.159 | 2.06E-03 | 0.693 |
| 162148_r_at | Apom | Apolipoprotein M | -4.875 | 2.69E-03 | 0.880 |
| 161951_f_at | Mcart1 | Mitochondrial carrier triple repeat 1 | -4.871 | 2.70E-03 | 0.758 |
| 94256_at | Clic4 | Chloride intracellular channel 4 (mitochondrial) | -4.848 | 2.76E-03 | 0.469 |
| 162080_f_at | Kctd17 | Potassium channel tetramerisation domain containing 17 | -4.834 | 2.79E-03 | 0.680 |
| 93705_at | Chrnb1 | Cholinergic receptor, nicotinic, beta polypeptide 1 (muscle) | -4.710 | 3.15E-03 | 0.745 |
| 94549_at | Mfsd1 | Major facilitator superfamily domain containing 1 | -4.687 | 3.22E-03 | 0.685 |
| 160076_at | Mtx2 | Metaxin 2 | -4.673 | 3.25E-03 | 0.659 |
| 102099_f_at | Kctd17 | Potassium channel tetramerisation domain containing 17 | -4.282 | 4.70E-03 | 0.734 |
| 94876_f_at | Gorasp2 | Golgi reassembly stacking protein 2 | -4.079 | 5.71E-03 | 0.679 |
| 93818_g_at | Tmed2 | Transmembrane emp24 domain trafficking protein 2 | -3.857 | 7.14E-03 | 0.611 |
| 93341_r_at | Copb2 | Coatomer protein complex, subunit beta 2 (beta prime) | -3.783 | 7.79E-03 | 0.742 |
| 103031_g_at | Dnm1 | Dynamin 1 | -3.775 | 7.85E-03 | 0.371 |
| 100492_at | Ap2a2 | Adaptor protein complex AP-2, alpha 2 subunit | -3.771 | 7.88E-03 | 0.554 |
| 93982_at | Derl1 | Der1-like domain family, member 1 | -3.771 | 7.87E-03 | 0.700 |
| *Cell cycle and DNA replication* | |  |  |  |  |
| 93023_f_at | Hist1h3f | Histone 1, h3f | 8.718 | 2.54E-04 | 2.881* |
| 94805_f_at | Hist3h2a | Histone 3, h2a | 7.343 | 4.46E-04 | 5.305 |
| 96416_f_at | Hist1h3d | Histone1, h3d | 7.011 | 5.45E-04 | 2.018 |
| 99541_at | Kif11 | Kinesin family member 11 | 6.742 | 6.04E-04 | 1.583 |
| 94288_at | Hist1h1c | Histone 1, h1c | 6.341 | 8.10E-04 | 3.373* |
| 92310_at | Plk2 | Polo-like kinase 2 (Drosophila) | 6.109 | 9.61E-04 | 5.232 |
| 93067_f_at | Hist2h2aa1 | Histone 2, h2aa1 | 6.042 | 1.02E-03 | 2.322 |
| 104690_at | Polm | Polymerase (DNA directed), mu | 5.861 | 1.18E-03 | 1.394 |
| 103821_at | Cdc6 | Cell division cycle 6 homolog (S. Cerevisiae) | 5.860 | 1.18E-03 | 2.085 |
| 104096_at | Orc4l | Origin recognition complex, subunit 4-like (S. Cerevisiae) | 5.826 | 1.21E-03 | 1.539 |
| 160127_at | Ccng1 | Cyclin G1 | 5.448 | 1.59E-03 | 4.992 |
| 98006_at | Pola2 | Polymerase (DNA directed), alpha 2 | 5.310 | 1.82E-03 | 1.772 |
| 94489_at | Ptp4a1 | Protein tyrosine phosphatase 4ª1 | 5.302 | 1.83E-03 | 2.969 |
| 96168_at | Kif23 | Kinesin family member 23 | 5.171 | 2.03E-03 | 1.676 |
| 103069_at | Lin9 | Lin-9 homolog (C. Elegans) | 5.150 | 2.08E-03 | 1.472 |
| 98110_at | Mdm2 | Transformed 46ouse 3T3 cell double minute 2 | 5.084 | 2.23E-03 | 6.825# |
| 100413_at | Ylpm1 | YLP motif containing 1 | 5.013 | 2.37E-03 | 1.300 |
| 95032_at | Prc1 | Protein regulator of cytokinesis 1 | 4.980 | 2.44E-03 | 1.730 |
| 93758_at | Incenp | Inner centromere protein | 4.856 | 2.74E-03 | 2.091 |
| 93889_f_at | Hist1h2ba | Histone 1, h2ba | 4.842 | 2.78E-03 | 2.327* |
| 93019_at | H2afx | H2A histone family, member X | 4.553 | 3.60E-03 | 2.408* |
| 102632_at | Aspm | Asp (abnormal spindle)-like, microcephaly associated (Drosophila) | 4.405 | 4.16E-03 | 1.838 |
| 160496_s_at | Mcm3 | Minichromosome maintenance deficient 3 (S. Cerevisiae) | 4.388 | 4.23E-03 | 2.108 |
| 93356_at | Mcm7 | Minichromosome maintenance deficient 7 (S. Cerevisiae) | 4.340 | 4.45E-03 | 1.459 |
| 97327_at | Fen1 | Flap structure specific endonuclease 1 | 4.259 | 4.78E-03 | 2.017 |
| 93833_s_at | Hist1h2bc | Histone 1, h2bc | 4.244 | 4.84E-03 | 3.310 |
| 100156_at | Mcm5 | Minichromosome maintenance deficient 5, cell division cycle 46 (S. Cerevisiae) | 4.237 | 4.88E-03 | 2.000* |
| 103207_at | Pola1 | Polymerase (DNA directed), alpha 1 | 4.056 | 5.81E-03 | 1.971 |
| 102292_at | Gadd45a | Growth arrest and DNA-damage-inducible 45 alpha | 3.781 | 7.81E-03 | 2.588 |
| 95100_at | Anapc5 | Anaphase-promoting complex subunit 5 | -8.502 | 2.77E-04 | 0.605 |
| 104537_at | Rmnd1 | Required for meiotic nuclear division 1 homolog (S. cerevisiae) | -7.999 | 3.34E-04 | 0.734 |
| 98478_at | Ccng2 | Cyclin G2 | -7.165 | 4.96E-04 | 0.263 |
| 103736_at | Sash1 | SAM and SH3 domain containing 1 | -6.151 | 9.27E-04 | 0.293 |
| 103501_at | Pura | Purine rich element binding protein A | -6.042 | 1.02E-03 | 0.437 |
| 99135_at | Cdc37 | Cell division cycle 37 homolog (S. Cerevisiae) | -5.160 | 2.06E-03 | 0.753 |
| 94819_f_at | Ccni | Cyclin I | -4.656 | 3.29E-03 | 0.360 |
| 102772_at | Abl1 | V-abl Abelson murine leukemia oncogene 1 | -4.365 | 4.33E-03 | 0.786 |
| 97482_at | Cab39l | Calcium binding protein 39-like | -4.271 | 4.74E-03 | 0.810 |
| 98609_at | Sept9 | Septin 9 | -4.228 | 4.91E-03 | 0.677 |
| 94084_at | Cdc26 | Cell division cycle 26 | -3.972 | 6.31E-03 | 0.730 |
| 99558_at | Ccnc | Cyclin C | -3.957 | 6.43E-03 | 0.560 |
| 101969_at | Nbl1 | Neuroblastoma, suppression of tumorigenicity 1 | -3.814 | 7.52E-03 | 0.710 |
| *Immunity and defense* | |  |  |  |  |
| 102218_at | Il6 | Interleukin 6 | 11.509 | 1.19E-04 | 30.055* |
| 101851_at | Cd200 | Cd200 antigen | 8.030 | 3.25E-04 | 2.982 |
| 101054_at | Cd74 | CD74 antigen (invariant polypeptide of major histocompatibility complex, class II antigen-associated) | 6.610 | 6.61E-04 | 2.064 |
| 92948_at | Csf2 | Colony stimulating factor 2 (granulocyte-macrophage) | 4.686 | 3.23E-03 | 1.603 |
| 101160_at | Cxcl2 | Chemokine (C-X-C motif) ligand 2 | 4.234 | 4.89E-03 | 3.074* |
| 98501_at | Il1rl1 | Interleukin 1 receptor-like 1 | 3.850 | 7.21E-03 | 4.931 |
| 95593_at | Golm1 | Golgi membrane protein 1 | -5.550 | 1.48E-03 | 0.782 |
| 93874_s_at | Il11ra2 | Interleukin 11 receptor, alpha chain 2 | -5.019 | 2.35E-03 | 0.261 |
| 97737_f_at | Nlrx1 | NLR family member X1 | -4.793 | 2.91E-03 | 0.724 |
| 160387_at | Cd302 | CD302 antigen | -4.654 | 3.30E-03 | 0.325 |
| 104501_at | Vapb | Vesicle-associated membrane protein, associated protein B and C | -4.584 | 3.51E-03 | 0.638 |
| 99584_at | Cd82 | CD82 antigen | -4.506 | 3.75E-03 | 0.656 |
| 99491_at | Il10rb | Interleukin 10 receptor, beta | -4.054 | 5.83E-03 | 0.637 |
| 97718_at | Ctla4 | Cytotoxic T-lymphocyte-associated protein 4 | -4.004 | 6.13E-03 | 0.807 |
| 160347_at | Ncaph2 | Non-SMC condensin II complex, subunit H2 | -3.874 | 7.02E-03 | 0.641 |
| 99010_at | Islr | Immunoglobulin superfamily containing leucine-rich repeat | -3.860 | 7.12E-03 | 0.259 |
| 103611_at | Cd47 | CD47 antigen (Rh-related antigen, integrin-associated signal transducer) | -3.815 | 7.51E-03 | 0.383 |
| *Response to interferon* | |  |  |  |  |
| 99100_at | Stat5a | Signal transducer and activator of transcription 5A | 5.969 | 1.08E-03 | 1.645* |
| 102401_at | Irf1 | Interferon regulatory factor 1 | 5.326 | 1.78E-03 | 6.039 |
| 99099_at | Stat3 | Signal transducer and activator of transcription 3 | 5.250 | 1.89E-03 | 1.625 |
| 160694_at | Ifngr2 | Interferon gamma receptor 2 | -4.754 | 3.04E-03 | 0.541 |
| 162447_f_at | Mvp | Major vault protein | -4.520 | 3.71E-03 | 0.730 |
| *Cell adhesion and migration* | |  |  |  |  |
| 101464_at | Timp1 | Tissue inhibitor of metalloproteinase 1 | 8.019 | 3.29E-04 | 2.785 |
| 103039_at | Itga5 | Integrin alpha 5 (fibronectin receptor alpha) | 5.144 | 2.10E-03 | 3.120 |
| 101450_at | Csf1 | Colony stimulating factor 1 (macrophage) | 4.305 | 4.60E-03 | 1.894 |
| 94643_at | Pvr | Poliovirus receptor | 4.294 | 4.64E-03 | 1.829 |
| 98280_at | Mmp16 | Matrix metallopeptidase 16 | 4.284 | 4.68E-03 | 1.652 |
| 99663_g_at | Nup85 | Nucleoporin 85 | 4.194 | 5.07E-03 | 1.391 |
| 99933_at | Pvrl2 | Poliovirus receptor-related 2 | 3.935 | 6.55E-03 | 1.902 |
| 98152_at | Ctnnd1 | Catenin (cadherin associated protein), delta 1 | 3.927 | 6.64E-03 | 1.769 |
| 99662_at | Nup85 | Nucleoporin 85 | 3.878 | 6.99E-03 | 1.504 |
| 97875_at | Adrm1 | Adhesion regulating molecule 1 | 3.828 | 7.38E-03 | 1.274 |
| 101110_at | Col6a3 | Procollagen, type VI, alpha 3 | -7.115 | 5.12E-04 | 0.199* |
| 99014_at | Apbb1 | Amyloid beta (A4) precursor protein-binding, family B, member 1 | -6.189 | 8.97E-04 | 0.737 |
| 92880_at | Mfge8 | Milk fat globule-EGF factor 8 protein | -6.112 | 9.56E-04 | 0.506 |
| 160346_at | Wdr6 | WD repeat domain 6 | -5.240 | 1.91E-03 | 0.755 |
| 97750_at | Lamr1 | Laminin receptor 1 (ribosomal protein SA) | -4.925 | 2.57E-03 | 0.602 |
| 96766_s_at | Tyro3 | TYRO3 protein tyrosine kinase 3 | -4.831 | 2.81E-03 | 0.618 |
| 104602_at | Waspip | Wiskott-Aldrich syndrome protein interacting protein | -4.721 | 3.13E-03 | 0.298 |
| 100142_at | Dgcr2 | DiGeorge syndrome critical region gene 2 | -4.574 | 3.53E-03 | 0.806 |
| 96742_at | Dpt | Dermatopontin | -4.559 | 3.58E-03 | 0.077 |
| 101359_at | Lamb2 | Laminin, beta 2 | -4.379 | 4.27E-03 | 0.459 |
| 104761_at | Antxr2 | Anthrax toxin receptor 2 | -4.314 | 4.57E-03 | 0.496 |
| 94236_at | Nisch | Nischarin | -4.227 | 4.91E-03 | 0.654 |
| 100990_g_at | Itgb1bp1 | Integrin beta 1 binding protein 1 | -4.170 | 5.22E-03 | 0.560 |
| 100457_at | Glg1 | Golgi apparatus protein 1 | -4.040 | 5.91E-03 | 0.647 |
| 103984_at | Pkhd1 | Polycystic kidney and hepatic disease 1 | -3.946 | 6.50E-03 | 0.832 |
| 104743_at | Cdh13 | Cadherin 13 | -3.867 | 7.07E-03 | 0.428 |
| *RNA metabolism and processing* | |  |  |  |  |
| 160208_at | Sf3b3 | Splicing factor 3b, subunit 3 | 7.439 | 4.35E-04 | 1.474 |
| 101936_at | Clk4 | CDC like kinase 4 | 7.415 | 4.37E-04 | 2.597* |
| 160351_at | Rnpep | Arginyl aminopeptidase (aminopeptidase B) | 6.436 | 7.64E-04 | 1.315 |
| 160273_at | Zfp36l2 | Zinc finger protein 36, C3H type-like 2 | 5.403 | 1.65E-03 | 2.030 |
| 101942_at | Sfrs14 | Splicing factor, arginine/serine-rich 14 | 5.124 | 2.13E-03 | 1.306 |
| 160182_at | Sfrs6 | Splicing factor, arginine/serine-rich 6 | 4.695 | 3.20E-03 | 1.583 |
| 102385_at | Wdr43 | WD repeat domain 43 | 4.452 | 3.99E-03 | 1.372 |
| 95791_s_at | Sfrs2 | Splicing factor, arginine/serine-rich 2 (SC-35) | 4.529 | 3.69E-03 | 1.917 |
| 99620_at | Sfpq | Splicing factor proline/glutamine rich (polypyrimidine tract binding protein associated) | 4.469 | 3.89E-03 | 1.583 |
| 100547_at | Rbm4b | RNA binding motif protein 4B | 4.399 | 4.19E-03 | 1.462 |
| 161002_at | Rbm15 | RNA binding motif protein 15 | 4.365 | 4.33E-03 | 1.277 |
| 99923_at | Pcf11 | Cleavage and polyadenylation factor subunit homolog (S. cerevisiae) | 4.239 | 4.87E-03 | 1.905 |
| 101003_at | Sfrs3 | Splicing factor, arginine/serine-rich 3 (srp20) | 4.235 | 4.88E-03 | 1.472 |
| 92317_at | Elavl2 | ELAV (embryonic lethal, abnormal vision, Drosophila)-like 2 (Hu antigen B) | 4.199 | 5.05E-03 | 1.224 |
| 97808_at | Sf3b1 | Splicing factor 3b, subunit 1 | 4.171 | 5.21E-03 | 1.946 |
| 103525_at | Hnrpll | Heterogeneous nuclear ribonucleoprotein L-like | 4.116 | 5.52E-03 | 1.469 |
| 96027_at | Sf3a1 | Splicing factor 3a, subunit 1 | 3.915 | 6.74E-03 | 1.358 |
| 104586_at | Sfrs1 | Splicing factor, arginine/serine-rich 1 (ASF/SF2) | 3.829 | 7.38E-03 | 1.760 |
| 98923_at | Rcl1 | RNA terminal phosphate cyclase-like 1 | 3.786 | 7.76E-03 | 1.535 |
| 93990_at | Hnrph1 | Heterogeneous nuclear ribonucleoprotein H1 | 3.780 | 7.82E-03 | 1.576 |
| 102399_at | Rbpms | RNA binding protein gene with multiple splicing | -5.926 | 1.12E-03 | 0.357 |
| 97517_at | Exosc4 | Exosome component 4 | -5.318 | 1.81E-03 | 0.802 |
| 92817_at | Imp3 | IMP3, U3 small nucleolar ribonucleoprotein, homolog (yeast) | -4.790 | 2.92E-03 | 0.658 |
| 97293_at | Rbm10 | RNA binding motif protein 10 | -4.260 | 4.77E-03 | 0.704* |
| 103678_at | Sbno1 | Sno, strawberry notch homolog 1 (Drosophila) | -4.200 | 5.04E-03 | 0.766 |
| 102356_at | Wdr23 | WD repeat domain 23 | -4.050 | 5.85E-03 | 0.646 |
| 103449_at | Lsm14b | LSM14 homolog B (SCD6, S. cerevisiae) | -4.040 | 5.91E-03 | 0.659 |
| 101524_at | Hnrpa3 | Heterogeneous nuclear ribonucleoprotein A3 | -4.030 | 5.96E-03 | 0.805 |
| 97536_at | Wdtc1 | WD and tetratricopeptide repeats 1 | -3.982 | 6.26E-03 | 0.664 |
| 99866_at | Rbms1 | RNA binding motif, single stranded interacting protein 1 | -3.843 | 7.26E-03 | 0.770 |
| *Cell Development and differentiation* | | |  |  |  |
| 100127_at | Crabp2 | Cellular retinoic acid binding protein II | 32.668 | 6.86E-06 | 10.735 |
| 101583_at | Btg2 | B-cell translocation gene 2, anti-proliferative | 17.686 | 3.89E-05 | 27.089 |
| 103830_at | Snai1 | Snail homolog 1 (Drosophila) | 13.279 | 6.63E-05 | 3.835 |
| 97813_at | Rela | V-rel reticuloendotheliosis viral oncogene homolog A (avian) | 11.095 | 1.30E-04 | 2.005* |
| 160367_at | Pdlim7 | PDZ and LIM domain 7 | 9.353 | 2.08E-04 | 2.135 |
| 93866_s_at | Mgp | 48ouse48 Gla protein | 6.763 | 5.95E-04 | 9.817 |
| 102737_at | Edn1 | Endothelin 1 | 6.615 | 6.59E-04 | 7.968 |
| 160162_at | Tagln2 | Transgelin 2 | 6.584 | 6.80E-04 | 2.483* |
| 95637_at | Flnb | Filamin, beta | 6.124 | 9.45E-04 | 2.572 |
| 161050_at | Nav2 | Neuron navigator 2 | 6.074 | 9.88E-04 | 1.713 |
| 103397_at | Hrb | HIV-1 Rev binding protein | 5.676 | 1.33E-03 | 1.816 |
| 100302_at | Maff | v-maf musculoaponeurotic fibrosarcoma oncogene family, protein F (avian) | 5.464 | 1.58E-03 | 6.847 |
| 99964_at | Vdr | Vitamin D receptor | 4.877 | 2.68E-03 | 3.932* |
| 103614_at | Nfkb2 | Nuclear factor of kappa light polypeptide gene enhancer in B-cells 2, p49/p100 | 4.869 | 2.71E-03 | 1.646 |
| 92399_at | Runx1 | Runt related transcription factor 1 | 4.830 | 2.81E-03 | 3.500 |
| 98817_at | Fst | Follistatin | 4.766 | 3.00E-03 | 4.121 |
| 99021_at | Prrx1 | Paired related homeobox 1 | 4.743 | 3.07E-03 | 4.544 |
| 160606_r_at | Adamts1 | A disintegrin-like and metallopeptidse (reprolysin type) with thrombospondin type 1 motif, 1 | 4.402 | 4.18E-03 | 1.332 |
| 102043_at | Tuft1 | Tuftelin 1 | 4.221 | 4.93E-03 | 1.492 |
| 97487_at | Serpine2 | Serine (or cysteine) peptidase inhibitor, clade E, member 2 | 4.101 | 5.60E-03 | 4.444 |
| 102956_at | Msx2 | Homeo box, msh-like 2 | 4.034 | 5.94E-03 | 2.199 |
| 93228_at | Hells | Helicase, lymphoid specific | 3.995 | 6.18E-03 | 2.007 |
| 94804_at | Pbx1 | Pre B-cell 49ouse49a49 transcription factor 1 | 3.937 | 6.55E-03 | 1.330 |
| 92722_f_at | Six1 | Sine oculis-related homeobox 1 homolog (Drosophila) | 3.882 | 6.97E-03 | 3.697 |
| 103342_at | Eed | Embryonic ectoderm development | 3.943 | 6.51E-03 | 2.491 |
| 160532_at | Tpm1 | Tropomyosin 1, alpha | 3.847 | 7.23E-03 | 1.505 |
| 96263_at | Rshl2 | Radial spokehead-like 2 | -7.276 | 4.55E-04 | 0.526 |
| 101484_at | Nbr1 | Neighbor of Brca1 gene 1 | -6.610 | 6.66E-04 | 0.555 |
| 93951_at | Golga3 | Golgi autoantigen, golgin subfamily a, 3 | -6.420 | 7.69E-04 | 0.631 |
| 99929_at | Ext2 | 49ouse49a49s (multiple) 2 | -6.033 | 1.03E-03 | 0.786 |
| 95706_at | Lgals3 | Lectin, galactose binding, soluble 3 | -5.930 | 1.11E-03 | 0.169 |
| 92348_at | Thra | Thyroid 49ouse49a receptor alpha | -5.788 | 1.25E-03 | 0.571 |
| 100876_at | Fez1 | Fasciculation and elongation protein zeta 1 (zygin I) | -5.324 | 1.79E-03 | 0.195 |
| 98078_at | Aph1a | Anterior pharynx defective 1ª homolog (C. Elegans) | -4.796 | 2.90E-03 | 0.836 |
| 97885_at | Tmem176b | Transmembrane protein 176B | -4.576 | 3.53E-03 | 0.494 |
| 100992_at | Phc1 | Polyhomeotic-like 1 (Drosophila) | -4.383 | 4.25E-03 | 0.727 |
| 96684_at | Grsf1 | G-rich RNA sequence binding factor 1 | -4.259 | 4.78E-03 | 0.610 |
| 103811_at | Invs | Inversin | -4.151 | 5.33E-03 | 0.801* |
| 160453_at | Ryk | Receptor-like tyrosine kinase | -4.111 | 5.54E-03 | 0.506 |
| 99374_at | Epo | Erythropoietin | -4.088 | 5.66E-03 | 0.769 |
| 160757_at | Rnf38 | Ring finger protein 38 | -4.075 | 5.72E-03 | 0.735 |
| 95557_at | Bmp1 | Bone morphogenetic protein 1 | -4.009 | 6.09E-03 | 0.675 |
| 103875_at | Ngrn | Neugrin, neurite outgrowth associated | -3.942 | 6.52E-03 | 0.584 |
| 162347_f_at | Bgn | Biglycan | -3.882 | 6.98E-03 | 0.634 |
| 92726_at | Sox6 | SRY-box containing gene 6 | -3.875 | 7.01E-03 | 0.865 |
| 97953_g_at | Tsc2 | Tuberous sclerosis 2 | -3.853 | 7.18E-03 | 0.572 |
| 160939_at | Twist2 | Twist homolog 2 (Drosophila) | -3.820 | 7.46E-03 | 0.361 |
| 102389_s_at | Gap43 | Growth associated protein 43 | -3.782 | 7.80E-03 | 0.740 |
| *Protein biosynthesis and ribosome biogenesis* | | |  |  |  |
| 99140_at | Mrpl16 | Mitochondrial ribosomal protein L16 | 7.005 | 5.51E-04 | 1.416 |
| 93488_at | TSR1 | TSR1, 20S rRNA accumulation, homolog (yeast) | 6.169 | 9.06E-04 | 2.527 |
| 96628_at | Eprs | Glutamyl-prolyl-tRNA synthetase | 3.947 | 6.49E-03 | 1.764 |
| 95070_at | Nars | Asparaginyl-tRNA synthetase | -12.323 | 9.15E-05 | 0.464 |
| 94327_at | Mrps18a | Mitochondrial ribosomal protein S18A | -6.415 | 7.73E-04 | 0.869 |
| 96138_at | Mars | Methionine-tRNA synthetase | -5.564 | 1.46E-03 | 0.729 |
| 101213_at | Arbp | Acidic ribosomal phosphoprotein P0 | -4.970 | 2.46E-03 | 0.721 |
| 94837_at | Eif3s8 | Eukaryotic translation initiation factor 3, subunit 8 | -4.952 | 2.50E-03 | 0.742 |
| 98936_at | Sars1 | Seryl-aminoacyl-tRNA synthetase 1 | -4.780 | 2.95E-03 | 0.664 |
| 100636_at | Eif4ebp1 | Eukaryotic translation initiation factor 4E binding protein 1 | -4.456 | 3.96E-03 | 0.627 |
| 93787_f_at | Mrpl18 | Mitochondrial ribosomal protein L18 | -4.412 | 4.13E-03 | 0.603* |
| 93564_at | Yars | Tyrosyl-tRNA synthetase | -4.046 | 5.86E-03 | 0.734 |
| 161480_i_at | Rplp1 | Ribosomal protein, large, P1 | -4.041 | 5.90E-03 | 0.695 |
| 96942_at | Eif3s6ip | Eukaryotic translation initiation factor 3, subunit 6 interacting protein | -3.974 | 6.30E-03 | 0.727 |
| 96604_at | Dus1l | Dihydrouridine synthase 1-like (S. Cerevisiae) | -3.872 | 7.04E-03 | 0.773 |
| 97647_at | Rps16 | Ribosomal protein S16 | -3.805 | 7.60E-03 | 0.730 |
| *Apoptosis* | |  |  |  |  |
| MurFAS_at | Fas | Fas (TNF receptor superfamily member) | 13.147 | 7.09E-05 | 2.737*# |
| 99392_at | Tnfaip3 | Tumor necrosis factor, alpha-induced protein 3 | 10.085 | 1.67E-04 | 38.585 |
| 161666_f_at | Gadd45b | Growth arrest and DNA-damage-inducible 45 beta | 9.205 | 2.20E-04 | 6.995 |
| 160829_at | Phlda1 | Pleckstrin homology-like domain, family A, member 1 | 7.632 | 4.10E-04 | 22.030* |
| 102921_s_at | Fas | Fas (TNF receptor superfamily member) | 6.051 | 1.01E-03 | 2.815* |
| 102734_at | Birc2 | Baculoviral IAP repeat-containing 2 | 5.824 | 1.21E-03 | 2.149 |
| 102887_at | Tnfrsf11b | Tumor necrosis factor receptor superfamily, member 11b (osteoprotegerin) | 5.730 | 1.29E-03 | 3.642 |
| 97825_at | Perp | PERP, TP53 apoptosis effector | 5.492 | 1.54E-03 | 10.824 |
| 101407_at | Fxn | Frataxin | 5.341 | 1.75E-03 | 1.315 |
| 101995_at | Sqstm1 | Sequestosome 1 | 4.477 | 3.86E-03 | 1.997* |
| 160268_at | Pdcl3 | Phosducin-like 3 | 4.404 | 4.16E-03 | 1.463 |
| 100005_at | Traf4 | Tnf receptor associated factor 4 | 4.043 | 5.89E-03 | 1.732 |
| 98056_at | Phlda3 | Pleckstrin homology-like domain, family A, member 3 | 3.993 | 6.19E-03 | 5.091 |
| 99070_at | Chuk | Conserved helix-loop-helix ubiquitous kinase | 3.769 | 7.90E-03 | 1.737 |
| 93064_at | Bnip2 | BCL2/adenovirus E1B 19kda-interacting protein 1, NIP2 | -5.315 | 1.82E-03 | 0.513 |
| 103216_f_at | Ikbkg | Inhibitor of kappab kinase gamma | -4.518 | 3.72E-03 | 0.690 |
| 103338_at | Parp6 | Poly (ADP-ribose) polymerase family, member 6 | -4.486 | 3.83E-03 | 0.645 |
| 160920_at | Bcl2l2 | Bcl2-like 2 | -4.476 | 3.86E-03 | 0.608 |
| 95660_at | Ethe1 | Ethylmalonic encephalopathy 1 | -4.251 | 4.81E-03 | 0.485 |
| 160489_at | Tnfaip2 | Tumor necrosis factor, alpha-induced protein 2 | -4.102 | 5.59E-03 | 0.580 |
| 161112_at | Parp16 | Poly (ADP-ribose) polymerase family, member 16 | -3.988 | 6.22E-03 | 0.746 |
| 104189_at | Traf6 | Tnf receptor-associated factor 6 | -3.985 | 6.24E-03 | 0.800 |
| 103671_at | Htatip2 | HIV-1 tat interactive protein 2, homolog (human) | -3.861 | 7.11E-03 | 0.517 |
| *Ubiquitin cycle* | |  |  |  |  |
| 160605_s_at | Usp38 | Ubiquitin specific peptidase 38 | 6.702 | 6.22E-04 | 2.092 |
| 99149_at | Trim59 | Tripartite motif-containing 59 | 6.124 | 9.43E-04 | 2.151 |
| 103906_f_at | Nedd4l | Neural precursor cell expressed, developmentally down-regulated gene 4-like | 5.821 | 1.22E-03 | 1.279 |
| 160110_at | Wwp2 | WW domain containing E3 ubiquitin protein ligase 2 | 4.463 | 3.91E-03 | 1.333 |
| 98906_at | Fbxo9 | F-box only protein 9 | -7.939 | 3.45E-04 | 0.579 |
| 99085_at | Usp3 | Ubiquitin specific peptidase 3 | -6.210 | 8.79E-04 | 0.624 |
| 93101_s_at | Nedd4 | Neural precursor cell expressed, developmentally down-regulted gene 4 | -5.013 | 2.37E-03 | 0.497* |
| 94457_at | Rbck1 | RanBP-type and C3HC4-type zinc finger containing 1 | -4.900 | 2.62E-03 | 0.725 |
| 160096_at | Spop | Speckle-type POZ protein | -4.286 | 4.67E-03 | 0.701 |
| 101421_at | Rnf5 | Ring finger protein 5 | -4.244 | 4.84E-03 | 0.699 |
| 95424_at | Sumo3 | SMT3 suppressor of mif two 3 homolog 3 (yeast) | -4.204 | 5.02E-03 | 0.636 |
| 102812_i_at | Ube1dc1 | Ubiquitin-activating enzyme E1-domain containing 1 | -4.103 | 5.59E-03 | 0.516 |
| 99086_g_at | Usp3 | Ubiquitin specific peptidase 3 | -4.067 | 5.76E-03 | 0.611 |
| 96244_at | Uchl1 | Ubiquitin carboxy-terminal hydrolase L1 | -3.984 | 6.25E-03 | 0.491 |
| *Electron transport and energy production* | | |  |  |  |
| 103619_at | Cyb5b | Cytochrome b5 type B | 3.962 | 6.39E-03 | 1.342 |
| 97013_f_at | Cyba | Cytochrome b-245, alpha polypeptide | -7.473 | 4.30E-04 | 0.168 |
| 100059_at | Cyba | Cytochrome b-245, alpha polypeptide | -7.052 | 5.26E-04 | 0.178 |
| 160088_at | Fmo5 | Flavin containing monooxygenase 5 | -5.444 | 1.59E-03 | 0.717 |
| 103850_at | Loxl1 | Lysyl oxidase-like 1 | -5.349 | 1.73E-03 | 0.553 |
| 92587_at | Fdx1 | Ferredoxin 1 | -4.915 | 2.59E-03 | 0.587 |
| 97922_at | Ncb5or | NADPH cytochrome B5 oxidoreductase | -4.638 | 3.36E-03 | 0.603 |
| 99019_at | Por | P450 (cytochrome) oxidoreductase | -4.474 | 3.87E-03 | 0.573 |
| 161083_at | Taok3 | TAO kinase 3 | -4.421 | 4.09E-03 | 0.583 |
| 96831_at | Pdia5 | Protein disulfide isomerase associated 5 | -4.376 | 4.27E-03 | 0.542 |
| 94831_at | Ctsb | Cathepsin B | -4.375 | 4.29E-03 | 0.455 |
| 101031_at | Surf1 | Surfeit gene 1 | -4.337 | 4.47E-03 | 0.597 |
| 160611_at | Cyp4v3 | Cytochrome P450, family 4, subfamily v, polypeptide 3 | -4.254 | 4.80E-03 | 0.476 |
| 98533_at | Cyb5 | Cytochrome b-5 | -4.154 | 5.31E-03 | 0.424* |
| 96542_at | Surf4 | Surfeit gene 4 | -3.902 | 6.83E-03 | 0.704 |
| *Cytoskeleton organization and biogenesis* | | |  |  |  |
| 92608_at | Csrp1 | Cysteine and glycine-rich protein 1 | 5.819 | 1.22E-03 | 4.181 |
| 160065_s_at | Csrp1 | Cysteine and glycine-rich protein 1 | 5.331 | 1.77E-03 | 4.255 |
| 98454_at | Palm | Paralemmin | -5.323 | 1.80E-03 | 0.804 |
| *Cell growth and proliferation* | |  |  |  |  |
| 94510_at | Wdr77 | WD repeat domain 77 | 5.718 | 1.30E-03 | 1.301 |
| 99915_at | Areg | Amphiregulin | 5.393 | 1.66E-03 | 2.163* |
| 103220_at | Ndnl2 | Necdin-like 2 | 4.877 | 2.68E-03 | 1.434 |
| 98531_g_at | Gas5 | Growth arrest specific 5 | 3.962 | 6.40E-03 | 1.659 |
| 97951_s_at | Tsc2 | Tuberous sclerosis 2 | -7.209 | 4.78E-04 | 0.677 |
| 103243_at | Emp2 | Epithelial membrane protein 2 | -7.205 | 4.80E-04 | 0.652* |
| 104390_at | Anp32a | Acidic (leucine-rich) nuclear phosphoprotein 32 family, member A | -6.099 | 9.68E-04 | 0.503 |
| 97426_at | Emp1 | Epithelial membrane protein 1 | -5.284 | 1.86E-03 | 0.514* |
| 102966_at | Cnot6l | CCR4-NOT transcription complex, subunit 6-like | -4.906 | 2.61E-03 | 0.658 |
| 93780_at | Them2 | Thioesterase superfamily member 2 | -4.768 | 3.00E-03 | 0.704 |
| 104367_at | Eps15 | Epidermal growth factor receptor pathway substrate 15 | -4.383 | 4.25E-03 | 0.524 |
| 96289_at | Stoml2 | Stomatin (Epb7.2)-like 2 | -3.961 | 6.42E-03 | 0.586 |
| 160108_at | Nupr1 | Nuclear protein 1 | -3.924 | 6.67E-03 | 0.288 |
| 97429_at | Snrk | SNF related kinase | -3.850 | 7.20E-03 | 0.569 |
| *Angiogenesis* | |  |  |  |  |
| 94147_at | Serpine1 | Serine (or cysteine) peptidase inhibitor, clade E, member 1 | 11.090 | 1.33E-04 | 25.915* |
| 101973_at | Cited2 | Cbp/p300-interacting transactivator, with Glu/Asp-rich carboxy-terminal domain, 2 | 9.432 | 1.94E-04 | 6.698 |
| 103520_at | Vegfa | Vascular endothelial growth factor A | 7.652 | 4.05E-04 | 5.785* |
| 98802_at | Ereg | Epiregulin | 6.740 | 6.09E-04 | 6.361 |
| 92559_at | Vcam1 | Vascular cell adhesion molecule 1 | -5.328 | 1.78E-03 | 0.356 |
| 93574_at | Serpinf1 | Serine (or cysteine) peptidase inhibitor, clade F, member 1 | -4.002 | 6.14E-03 | 0.332 |
| 95016_at | Nrp1 | Neuropilin 1 | -3.826 | 7.40E-03 | 0.691* |
| *Proteolysis and peptidolysis* | |  |  |  |  |
| 98976_at | Adamdec1 | ADAM-like, decysin 1 | 4.303 | 4.61E-03 | 1.162 |
| 104467_at | Cpd | Carboxypeptidase D | 4.037 | 5.93E-03 | 1.290 |
| 93039_at | Pgcp | Plasma glutamate carboxypeptidase | -6.532 | 7.07E-04 | 0.147 |
| 96211_at | Dpp8 | Dipeptidylpeptidase 8 | -6.167 | 9.08E-04 | 0.458 |
| 95662_at | X83328 | EST X83328 | -5.574 | 1.44E-03 | 0.650 |
| 93261_at | Lgmn | Legumain | -5.050 | 2.29E-03 | 0.362 |
| 160328_at | Lonp1 | Lon peptidase 1, mitochondrial | -4.907 | 2.61E-03 | 0.642 |
| 93349_at | Pcolce | Procollagen C-endopeptidase enhancer protein | -3.776 | 7.83E-03 | 0.522 |
| *Microtubule dynamics* | |  |  |  |  |
| 96298_f_at | Dynll1 | Dynein light chain LC8-type1 | 4.184 | 5.13E-03 | 1.201 |
| 94835_f_at | Tubb2 | Tubulin, beta 2 | 4.167 | 5.23E-03 | 1.880 |
| 95135_at | Mid1ip1 | Mid1 interacting protein 1 (gastrulation specific G12-like (zebrafish)) | -7.201 | 4.83E-04 | 0.156 |
| 93609_at | Spg20 | Spastic paraplegia 20, spartin (Troyer syndrome) homolog (human) | -5.836 | 1.20E-03 | 0.779 |
| 160288_at | Map1lc3b | Microtubule-associated protein 1 light chain 3 beta | -4.814 | 2.85E-03 | 0.658* |
| 101480_at | Dnalc4 | Dynein, axonemal, light chain 4 | -3.939 | 6.54E-03 | 0.661 |
| *Protein folding* | |  |  |  |  |
| 92571_at | Hspa4 | Heat shock protein 4 | 4.939 | 2.54E-03 | 2.479* |
| 100353_g_at | Hspa4 | Heat shock protein 4 | 4.654 | 3.31E-03 | 2.017* |
| 94915_at | Ppib | Peptidylprolyl isomerase B | -8.002 | 3.32E-04 | 0.520* |
| 97868_at | Dnaja3 | DnaJ (Hsp40) homolog, subfamily A, member 3 | -6.912 | 5.72E-04 | 0.702 |
| 96249_at | Sep15 | Selenoprotein | -5.964 | 1.09E-03 | 0.659 |
| 160144_at | Dnajc10 | DnaJ (Hsp40) homolog, subfamily C, member 10 | -4.363 | 4.34E-03 | 0.637 |
| *Blood coagulation* | |  |  |  |  |
| 97689_at | F3 | Coagulation factor III | 8.141 | 3.13E-04 | 7.387* |
| 95355_at | Agtrap | Angiotensin II, type I receptor-associated protein | -5.818 | 1.23E-03 | 0.542 |
| *DNA repair* | |  |  |  |  |
| 102001_at | Rrm2 | Ribonucleotide reductase M2 | 6.690 | 6.27E-04 | 3.077 |
| 93138_at | 2410012H22Rik | RIKEN cDNA 2410012H22 gene | 5.013 | 2.37E-03 | 1.712 |
| 102853_at | Smc3 | Structural maintenace of chromosomes 3 | 4.337 | 4.46E-03 | 1.448 |
| 102976_at | Brca1 | Breast cancer 1 | 3.792 | 7.71E-03 | 1.392 |
| 94420_f_at | Cry1 | Cryptochrome 1 (photolyase-like) | -4.610 | 3.46E-03 | 0.481 |
| 99938_at | Xrcc1 | X-ray repair complementing defective repair in Chinese hamster cells 1 | -4.166 | 5.23E-03 | 0.713 |
| 92318_at | 2010301N04Rik | RIKEN cDNA 2010301N04 gene | -4.025 | 5.99E-03 | 0.836 |
| *Peroxisome organization and biogenesis* | | |  |  |  |
| 99469_at | Pex6 | Peroxisomal biogenesis factor 6 | -4.032 | 5.95E-03 | 0.665 |
| 103814_at | Pex11b | Peroxisomal biogenesis factor 11b | -3.827 | 7.39E-03 | 0.765 |
| 103815_at | Pex11b | Peroxisomal biogenesis factor 11b | -3.812 | 7.54E-03 | 0.761 |
| *Protein modification* | |  |  |  |  |
| 93985_at | Tiparp | TCDD-inducible poly(ADP-ribose) polymerase | 4.389 | 4.22E-03 | 8.684 |
| 95057_at | Herpud1 | Homocysteine-inducible, endoplasmic reticulum stress-inducible, ubiquitin-like domain member 1 | -9.401 | 2.04E-04 | 0.442 |
| *Autophagy* | |  |  |  |  |
| 95138_at | Wipi2 | WD repeat domain, phosphoinositide interacting 2 | -5.328 | 1.78E-03 | 0.639 |
| *GPI anchor biosynthetic process* | |  |  |  |  |
| 160444_at | Pigx | Phosphatidylinositol glycan, class X | -7.874 | 3.57E-04 | 0.768 |
| *Response to oxidative stress* | |  |  |  |  |
| 103607_at | Gpx7 | Glutathione peroxidase 7 | -3.962 | 6.40E-03 | 0.311 |
| *Unknown* | |  |  |  |  |
| 94829_at | Tmem70 | Transmembrane protein 70 | 14.397 | 6.18E-05 | 2.932 |
| 160682_at | 6430706D22Rik | RIKEN cDNA 6430706D22 gene | 10.004 | 1.72E-04 | 1.991 |
| 160707_at | D10Wsu102e | DNA segment, Chr 10, Wayne State University 102, expressed | 7.672 | 4.00E-04 | 1.530 |
| 96156_at | Snhg6 | Small nucleolar RNA host gene (non-protein coding) 6 | 6.104 | 9.63E-04 | 2.596 |
| 100348_at | X83313 | EST X83313 | 5.344 | 1.75E-03 | 3.478 |
| 96785_at | Ankrd47 | Ankyrin repeat domain 47 | 5.101 | 2.18E-03 | 3.442 |
| 97258_at | Lactb2 | Lactamase, beta 2 | 5.076 | 2.25E-03 | 1.842 |
| 99366_at | Pqlc3 | PQ loop repeat containing | 4.936 | 2.54E-03 | 2.427 |
| 160768_at | D030056L22Rik | RIKEN cDNA D030056L22 gene | 4.884 | 2.66E-03 | 1.702 |
| 93426_at | Atad2b | ATPase family, AAA domain containing 2B | 4.551 | 3.61E-03 | 1.472 |
| 100442_at | LOC100046166 | Similar to transforming growth factor beta regulated gene 4 | 4.007 | 6.09E-03 | 1.426* |
| 160310_at | D19Bwg1357e | DNA segment, Chr 19, Brigham & Women's Genetics 1357 expressed | 3.918 | 6.72E-03 | 1.376 |
| 96352_at | 2400001E08Rik | RIKEN cDNA 2400001E08 gene | 3.797 | 7.67E-03 | 1.174 |
| 96614_at | 4933426M11Rik | RIKEN cDNA 4933426M11 gene | 3.779 | 7.82E-03 | 1.757 |
| 92268_at | 2700007P21Rik | RIKEN cDNA 2700007P21 gene | -8.805 | 2.45E-04 | 0.483 |
| 99139_at | 2310075A12Rik | RIKEN cDNA 2310075A12 gene | -6.747 | 5.99E-04 | 0.565 |
| 160904_at | B230317C12Rik | RIKEN cDNA B230317C12 gene | -6.480 | 7.41E-04 | 0.620 |
| 95052_at | C1qdc2 | C1q domain containing 2 | -6.272 | 8.53E-04 | 0.324 |
| 104434_at | Unc119b | Unc-119 homolog B (C. elegans) | -6.156 | 9.20E-04 | 0.702 |
| 97423_at | 1500035H01Rik | RIKEN cDNA 1500035H01 gene | -5.610 | 1.41E-03 | 0.546 |
| 95119_at | 1110038D17Rik | RIKEN cDNA 1110038D17 gene | -5.568 | 1.45E-03 | 0.709 |
| 94504_at | 4930570C03Rik | RIKEN cDNA 4930570C03 gene | -5.454 | 1.59E-03 | 0.708 |
| 160513_at | 2210016F16Rik | RIKEN cDNA 2210016F16 gene | -5.342 | 1.75E-03 | 0.525 |
| 96240_at | Tmem106c | Transmembrane protein 106C | -5.279 | 1.86E-03 | 0.550 |
| 161997_f_at | NA | NA | -5.104 | 2.18E-03 | 0.697 |
| 96014_at | D4Ertd196e | DNA segment, Chr 4, ERATO Doi 196, expressed | -5.102 | 2.18E-03 | 0.775 |
| 100039_at | Tmem4 | Transmembrane protein 4 | -5.041 | 2.32E-03 | 0.699* |
| 160963_at | 9630050M13Rik | RIKEN cDNA 9630050M13 gene | -4.917 | 2.59E-03 | 0.493 |
| 97143_at | NA | NA | -4.808 | 2.87E-03 | 0.765 |
| 96615_at | Ypel3 | Yippee-like 3 (Drosophila) | -4.749 | 3.05E-03 | 0.507 |
| 104122_at | D330001F17Rik | RIKEN cDNA D330001F17 gene | -4.635 | 3.37E-03 | 0.798 |
| 102233_at | 4833442J19Rik | RIKEN cDNA 4833442J19 gene | -4.572 | 3.54E-03 | 0.615 |
| 96917_at | 2410166I05Rik | RIKEN cDNA 2410166I05 gene | -4.553 | 3.60E-03 | 0.712 |
| 92250_s_at | Prcc | Papillary renal cell carcinoma (translocation-associated) | -4.518 | 3.72E-03 | 0.629* |
| 94958_at | 1110013L07Rik | RIKEN cDNA 1110013L07 gene | -4.380 | 4.26E-03 | 0.599 |
| 100054_s_at | D2Wsu81e | DNA segment, Chr 2, Wayne State University 81, expressed | -4.357 | 4.37E-03 | 0.723 |
| 93059_at | 2610204K14Rik | RIKEN cDNA 2610204K14 gene | -4.231 | 4.90E-03 | 0.662 |
| 94951_at | Las1l | LAS1-like (S. Cerevisiae) | -4.198 | 5.06E-03 | 0.796 |
| 97798_at | 4930504E06Rik | RIKEN cDNA 4930504E06 gene | -4.085 | 5.68E-03 | 0.613 |
| 160906_i_at | NA | NA | -3.986 | 6.23E-03 | 0.563 |
| 95045_at | 0610012D09Rik | RIKEN cDNA 0610012D09 gene | -3.888 | 6.92E-03 | 0.638 |
| 162081_f_at | NA | NA | -3.887 | 6.94E-03 | 0.715 |
| 160563_at | Serf2 | Small EDRK-rich factor 2 | -3.853 | 7.18E-03 | 0.760 |
| 104038_at | 4933434E20Rik | RIKEN cDNA 4933434E20 gene | -3.847 | 7.23E-03 | 0.744 |
| 104446_f_at | 4933428G09Rik | RIKEN cDNA 4933428G09 gene | -3.832 | 7.34E-03 | 0.781 |
| 97554_at | BC005624 | cDNA sequence BC005624 | -3.809 | 7.56E-03 | 0.707 |

**Table S7. Differential gene expression in serum-starved, G0-arrested H-ras^-/-^ fibroblasts after incubation of cell cultures in the presence of serum for 8 hours**. List of 862 differentially expressed probesets (808 different genes) identified by means of SAM contrast (FDR=0,09) comparing the microarray-generated transcriptional profile of WT fibroblasts that had been serum-starved for 24h to that of similarly starved, H-*ras***^-/-^** fibroblast cultures submitted to subsequent incubation in the presence of 20% FBS for 8 hours. To concentrate on loci exclusively regulated by Ras, genes sharing similar values of differential expression between the WT and H-*ras*^-/-^ fibroblast (ratio of the R-fold values in their respective lists within the range 0.6-1.5) were excluded from this list. The differentially expressed loci are identified by Affymetrix probeset ID, gene name and symbol, and listed according to functional category and degree of overexpression or repression, quantitated by d(i) value. The * and # symbols in the R.fold column denote independent validation of the transcriptional data obtained by means of quantitative RT-PCR or Western immunoblot, respectively. d(i) is a parameter measuring the statistical distance separating the calculated expression value of each gene probeset from the null hypothesis (no-change). p-value is an statistical measure indicating the probability of random expression for that probeset. R fold is the log2 value of the fold change measuring the overexpression or repression of the probesets in the collection of microarrays. **.**

| **Probeset ID** | **Gene symbol** | **Gene name** | **d.value** | **p.value** | **R.fold** |
| --- | --- | --- | --- | --- | --- |
| *Signal transduction* | | |  |  |  |
| 160353_i_at | Mapkapk2 | MAP kinase-activated protein kinase 2 | 20.544 | 2.52E-05 | 2.215 |
| 102663_at | Plaur | Urokinase plasminogen activator receptor | 11.987 | 2.10E-04 | 5.412 |
| 98603_s_at | Rangap1 | RAN GTPase activating protein 1 | 10.178 | 4.30E-04 | 1.687* |
| 97991_at | Kras | v-Ki-ras2 Kirsten rat sarcoma viral oncogene homolog | 9.259 | 7.16E-04 | 2.210 |
| 94378_at | Rgs16 | Regulator of G-protein signaling 16 | 9.158 | 7.55E-04 | 6.044* |
| 94853_at | Gnb1 | Guanine nucleotide binding protein, beta 1 | 8.712 | 9.20E-04 | 3.199* |
| 98403_at | Gna-rs1 | Guanine nucleotide binding protein, related sequence 1 | 8.315 | 1.08E-03 | 1.898 |
| 161609_at | Rgs16 | Regulator of G-protein signaling 16 | 8.082 | 1.19E-03 | 5.003* |
| 103605_g_at | Rgs19 | Regulator of G-protein signaling 19 | 8.009 | 1.24E-03 | 1.991 |
| 97458_at | Gnb1 | Guanine nucleotide binding protein, beta 1 | 7.748 | 1.39E-03 | 3.179* |
| 95721_at | Mapkapk2 | MAP kinase-activated protein kinase 2 | 7.542 | 1.56E-03 | 1.820 |
| 98602_at | Rangap1 | RAN GTPase activating protein 1 | 7.206 | 1.83E-03 | 1.721* |
| 93949_at | Gnb4 | Guanine nucleotide binding protein, beta 4 | 7.173 | 1.86E-03 | 1.690* |
| 93602_at | Rps6ka4 | Ribosomal protein S6 kinase, polypeptide 4 | 7.015 | 2.00E-03 | 1.941* |
| 94828_at | Oprs1 | Opioid receptor, sigma 1 | 6.793 | 2.26E-03 | 1.892 |
| 94482_at | Csnk2a2 | Casein kinase II, alpha 2, polypeptide | 6.555 | 2.56E-03 | 1.520 |
| 104761_at | Antxr2 | Anthrax toxin receptor 2 | 6.523 | 2.60E-03 | 2.161 |
| 93254_at | Mapk1 | Mitogen activated protein kinase 1 | 6.428 | 2.78E-03 | 1.363 |
| 94889_at | Vapa | Vesicle-associated membrane protein, associated protein A | 6.354 | 2.92E-03 | 1.604 |
| 94483_at | Csnk2a2 | Casein kinase II, alpha 2, polypeptide | 6.072 | 3.51E-03 | 1.710 |
| 102787_at | Gpr56 | G protein-coupled receptor 56 | 6.055 | 3.56E-03 | 1.945 |
| 102896_at | Dok1 | Docking protein 1 | 5.758 | 4.22E-03 | 3.159 |
| 102821_s_at | Rasl2-9 | RAS-like, family 2, locus 9 | 5.721 | 4.31E-03 | 2.340* |
| 104339_at | Pygo2 | Pygopus 2 | 5.590 | 4.73E-03 | 1.474 |
| 103511_at | Arhgap8 | Rho GTPase activating protein 8 | 5.564 | 4.83E-03 | 2.000 |
| 96102_i_at | Rad23b | RAD23b homolog (S. cerevisiae) | 5.525 | 4.94E-03 | 1.764* |
| 103606_r_at | Rgs19 | Regulator of G-protein signaling 19 | 5.473 | 5.10E-03 | 1.664 |
| 95358_at | Pip5k2a | Phosphatidylinositol-4-phosphate 5-kinase, type II, alpha | 5.417 | 5.29E-03 | 1.606 |
| 102379_at | Rassf1 | Ras association (RalGDS/AF-6) domain family 1 | 5.309 | 5.66E-03 | 2.256* |
| 102299_at | Prkca | Protein kinase C, alpha | 5.269 | 5.82E-03 | 2.035 |
| 95666_at | Cops8 | COP9 (constitutive photomorphogenic) homolog, subunit 8 (Arabidopsis thaliana) | 5.215 | 6.03E-03 | 1.814 |
| 103416_at | Mapk6 | Mitogen-activated protein kinase 6 | 5.196 | 6.12E-03 | 2.583* |
| 96881_at | Commd6 | COMM domain containing 6 | 5.135 | 6.39E-03 | 1.499 |
| 99978_s_at | Mapk14 | Mitogen activated protein kinase 14 | 5.089 | 6.57E-03 | 1.742 |
| 94854_g_at | Gnb1 | Guanine nucleotide binding protein, beta 1 | 5.079 | 6.61E-03 | 2.711* |
| 93070_at | Ranbp5 | RAN binding protein 5 | 5.063 | 6.71E-03 | 3.395* |
| 98916_at | Ppp2r2a | Protein phosphatase 2 (formerly 2A), regulatory subunit B (PR 52), alpha isoform | 4.992 | 7.03E-03 | 1.342 |
| 93315_at | Map2k3 | Mitogen activated protein kinase kinase 3 | 4.946 | 7.24E-03 | 2.874* |
| 101977_at | Nudt3 | Nudix (nucleotide diphosphate linked moiety X)-type motif 3 | 4.924 | 7.35E-03 | 1.381 |
| 96733_at | Rap1gds1 | RAP1, GTP-GDP dissociation stimulator 1 | 4.875 | 7.60E-03 | 1.458 |
| 97509_f_at | Fgfr1 | Fibroblast growth factor receptor 1 | 4.850 | 7.74E-03 | 2.199 |
| 94913_at | G3bp2 | GTPase activating protein (SH3 domain) binding protein 2 | 4.829 | 7.86E-03 | 1.617 |
| 104047_at | Mapk8 | Mitogen activated protein kinase 8 | 4.815 | 7.95E-03 | 1.537 |
| 97535_at | Ywhah | Tyrosine 3-monooxygenase/tryptophan 5-monooxygenase activation protein, eta polypeptide | 4.760 | 8.30E-03 | 2.039 |
| 101034_at | Grb2 | Growth factor receptor bound protein 2 | 4.757 | 8.33E-03 | 1.530 |
| 96637_at | Tbc1d1 | TBC1 domain family, member 1 | 4.731 | 8.48E-03 | 2.033 |
| 102850_at | Tnk2 | Tyrosine kinase, non-receptor, 2 | 4.725 | 8.51E-03 | 1.814 |
| 104254_at | Ralgps2 | Ral GEF with PH domain and SH3 binding motif 2 | 4.679 | 8.77E-03 | 1.318 |
| 104697_at | Rhoj | Ras homolog gene family, member J | 4.634 | 9.08E-03 | 1.496 |
| 97339_at | Git1 | G protein-coupled receptor kinase-interactor 1 | 4.632 | 9.09E-03 | 1.241 |
| 101254_at | Ran | RAN, member RAS oncogene family | 4.606 | 9.30E-03 | 1.864 |
| 93872_at | Gfra1 | Glial cell line derived neurotrophic factor family receptor alpha 1 | 4.595 | 9.38E-03 | 3.077 |
| 101582_at | Gnl2 | Guanine nucleotide binding protein-like 2 (nucleolar) | 4.551 | 9.74E-03 | 1.599 |
| 93314_g_at | Map2k3 | Mitogen activated protein kinase kinase 3 | 4.526 | 9.95E-03 | 1.602* |
| 96206_at | Phldb1 | Pleckstrin homology-like domain, family B, member 1 | 4.455 | 1.05E-02 | 1.456 |
| 98948_at | Gnl3 | Guanine nucleotide binding protein-like 3 (nucleolar) | 4.411 | 1.08E-02 | 1.989 |
| 94377_at | Gng11 | Guanine nucleotide binding protein (G protein), gamma 11 | 4.356 | 1.14E-02 | 1.907 |
| 103642_at | RP23-336J1.4 | Ras-GTPase-activating protein SH3-domain binding protein | 4.264 | 1.22E-02 | 1.825 |
| 93285_at | Dusp6 | Dual specificity phosphatase 6 | 4.237 | 1.25E-02 | 4.579 |
| 94503_at | Rab8a | RAB8A, member RAS oncogene family | 4.092 | 1.39E-02 | 1.244 |
| 160100_at | Efhd2 | EF hand domain containing 2 | 4.058 | 1.43E-02 | 1.431 |
| 92185_at | Arl4C | ADP-ribosylation factor-like 4C | 4.045 | 1.45E-02 | 3.350 |
| 103402_at | Tm7sf3 | Transmembrane 7 superfamily member 3 | -9.437 | 6.45E-04 | 0.357 |
| 93614_at | Rragd | Ras-related GTP binding D | -8.886 | 8.47E-04 | 0.884 |
| 104108_at | Rab6ip1 | Rab6 interacting protein 1 | -7.633 | 1.46E-03 | 0.458 |
| 97106_at | Map3k8 | Mitogen activated protein kinase kinase kinase 8 | -7.582 | 1.52E-03 | 0.461* |
| 92619_at | Wbp1 | WW domain binding protein 1 | -7.349 | 1.72E-03 | 0.734 |
| 103793_at | Mvp | Major vault protein | -6.470 | 2.70E-03 | 0.587 |
| 103592_at | Map2k5 | Mitogen activated protein kinase kinase 5 | -5.843 | 4.02E-03 | 0.699 |
| 104699_at | Pik3c3 | Phosphoinositide-3-kinase, class 3 | -5.736 | 4.27E-03 | 0.710 |
| 102117_at | Rabl4 | RAB, member of RAS oncogene family-like 4 | -5.659 | 4.51E-03 | 0.569 |
| 99164_at | Mapbpip | Mitogen activated protein binding protein interacting protein | -5.435 | 5.23E-03 | 0.776 |
| 160900_at | Gkap1 | G kinase anchoring protein 1 | -5.404 | 5.33E-03 | 0.435 |
| 93011_at | Gabarapl1 | Gamma-aminobutyric acid (GABA(A)) receptor-associated protein-like 1 | -5.320 | 5.62E-03 | 0.405 |
| 97888_at | Rhog | Ras homolog gene family, member G | -5.104 | 6.50E-03 | 0.535 |
| 94036_at | Cdc42ep4 | CDC42 effector protein (Rho GTPase binding) 4 | -5.070 | 6.65E-03 | 0.711 |
| 93748_at | Grinl1a | Glutamate receptor, ionotropic, N-methyl D-aspartate-like 1A | -4.988 | 7.05E-03 | 0.803 |
| 160373_i_at | Sdpr | Serum deprivation response | -4.922 | 7.37E-03 | 0.335* |
| 104427_at | Sbf2 | SET binding factor 2 | -4.916 | 7.41E-03 | 0.479 |
| 160747_at | Rgs3 | Regulator of G-protein signaling 3 | -4.775 | 8.21E-03 | 0.707* |
| 104053_at | Trio | Triple functional domain (PTPRF interacting) | -4.705 | 8.60E-03 | 0.487 |
| 93936_at | Ptprg | Protein tyrosine phosphatase, receptor type, G | -4.615 | 9.24E-03 | 0.708 |
| 92698_at | Mertk | c-mer proto-oncogene tyrosine kinase | -4.515 | 1.01E-02 | 0.706 |
| *cAMP dependent signalling* | | |  |  |  |
| 93214_at | Camk2d | Calcium/calmodulin-dependent protein kinase II, delta | 5.707 | 4.36E-03 | 1.996 |
| 97096_at | Prkar2a | Protein kinase, cAMP dependent regulatory, type II alpha | 5.306 | 5.67E-03 | 2.523 |
| 103316_at | Camsap1 | Calmodulin regulated spectrin-associated protein 1 | 4.606 | 9.30E-03 | 1.769 |
| 95001_at | Akap8 | A kinase (PRKA) anchor protein 8 | 4.355 | 1.14E-02 | 1.451* |
| 94390_at | Akap8 | A kinase (PRKA) anchor protein 8 | 4.102 | 1.38E-02 | 1.568* |
| *Transcription* | | |  |  |  |
| 100935_at | Mlx | MAX-like protein X | 17.340 | 4.58E-05 | 1.569* |
| 99602_at | Klf10 | Kruppel-like factor 10 | 13.705 | 1.21E-04 | 3.236 |
| 98021_at | Praf1 | Polymerase (RNA) I associated factor 1 | 12.084 | 1.97E-04 | 2.827 |
| 102363_r_at | Junb | Jun-B oncogene | 11.015 | 3.18E-04 | 2.056 |
| 99610_at | Ss18 | Synovial sarcoma translocation, Chromosome 18 | 10.527 | 3.64E-04 | 1.962* |
| 102362_i_at | Junb | Jun-B oncogene | 10.341 | 3.96E-04 | 2.313 |
| 99109_at | Ier2 | Immediate early response 2 | 9.979 | 4.78E-04 | 3.676* |
| 161881_f_at | Zfp259 | Zinc finger protein 259 | 8.087 | 1.19E-03 | 1.417 |
| 95433_at | Ddx54 | DEAD (Asp-Glu-Ala-Asp) box polypeptide 54 | 7.758 | 1.38E-03 | 2.261 |
| 103643_at | Zdhhc16 | Zinc finger, DHHC domain containing 16 | 7.661 | 1.44E-03 | 1.901 |
| 96083_s_at | Hnrpdl | Heterogeneous nuclear ribonucleoprotein d-like | 7.359 | 1.71e-03 | 1.789 |
| 160654_at | Rfxank | Regulatory factor X-associated ankyrin-containing protein | 7.206 | 1.83E-03 | 2.655 |
| 104169_at | Zic1 | Zinc finger protein of the cerebellum 1 | 6.976 | 2.05E-03 | 4.383 |
| 92979_at | Etv4 | Ets variant gene 4 (E1A enhancer binding protein, E1AF) | 6.865 | 2.18E-03 | 2.204 |
| 160898_at | Abt1 | Activator of basal transcription | 6.826 | 2.23E-03 | 1.407 |
| 98430_at | Surf5 | Surfeit gene 5 | 6.592 | 2.51E-03 | 1.355 |
| 94466_f_at | Cebpz | CCAAT/enhancer binding protein zeta | 6.586 | 2.51E-03 | 2.194 |
| 99095_at | Max | Max protein | 6.480 | 2.68E-03 | 2.212 |
| 101086_f_at | Cnbp1 | Cellular nucleic acid binding protein 1 | 6.476 | 2.68E-03 | 1.745 |
| 103318_at | Gabpb1 | GA repeat binding protein, beta 1 | 6.434 | 2.76E-03 | 1.380 |
| 92654_at | Polr3a | Polymerase (RNA) III (DNA directed) polypeptide A | 6.418 | 2.79E-03 | 1.584 |
| 99535_at | Ccrn4l | CCR4 carbon catabolite repression 4-like (S. cerevisiae) | 6.384 | 2.86E-03 | 3.554 |
| 102800_at | Foxc2 | Forkhead box C2 | 6.238 | 3.16E-03 | 4.035* |
| 160244_at | Fem1a | Feminization 1 homolog a (C. Elegans) | 6.095 | 3.45E-03 | 1.222 |
| 100302_at | Maff | V-maf musculoaponeurotic fibrosarcoma oncogene family, protein F (avian) | 6.047 | 3.58E-03 | 1.784 |
| 94968_at | Nfyc | Nuclear transcription factor-Y gamma | 5.967 | 3.76E-03 | 1.384 |
| 101088_f_at | Cnbp1 | Cellular nucleic acid binding protein 1 | 5.900 | 3.91E-03 | 1.675 |
| 102382_at | Arntl | Aryl hydrocarbon receptor nuclear translocator-like | 5.869 | 3.98E-03 | 1.953 |
| 94073_at | Polr2g | Polymerase (RNA) II (DNA directed) polypeptide G | 5.800 | 4.13E-03 | 1.581 |
| 95755_at | Csda | Cold shock domain protein A | 5.752 | 4.23E-03 | 1.506* |
| 95659_at | Actl6a | Actin-like 6A | 5.590 | 4.72E-03 | 2.305 |
| 93782_at | Rnf4 | Ring finger protein 4 | 5.590 | 4.72E-03 | 1.657 |
| 102069_at | Mtf2 | Metal response element binding transcription factor 2 | 5.166 | 6.24E-03 | 1.406 |
| 100130_at | Jun | Jun oncogene | 5.098 | 6.53E-03 | 1.618 |
| 104303_i_at | Polr3k | Polymerase (RNA) III (DNA directed) polypeptide K | 5.096 | 6.55E-03 | 1.899 |
| 104304_r_at | Polr3k | Polymerase (RNA) III (DNA directed) polypeptide K | 5.080 | 6.60E-03 | 1.713 |
| 96305_at | Bud31 | BUD31 homolog (yeast) | 5.059 | 6.72E-03 | 1.702 |
| 97394_at | Smarca5 | SWI/SNF related, matrix associated, actin dependent regulator of chromatin, subfamily a, member 5 | 5.006 | 6.96E-03 | 1.912* |
| 102371_at | Nr4a1 | Nuclear receptor subfamily 4, group A, member 1 | 4.958 | 7.19E-03 | 1.260 |
| 160449_at | Dr1 | Down-regulator of transcription 1 | 4.918 | 7.41E-03 | 1.394* |
| 102661_at | Egr2 | Early growth response 2 | 4.915 | 7.42E-03 | 1.700 |
| 99950_at | Tbp | TATA box binding protein | 4.912 | 7.43E-03 | 1.412 |
| 97705_at | Snapc3 | Small nuclear RNA activating complex, polypeptide 3 | 4.868 | 7.64E-03 | 1.347 |
| 162016_f_at | Foxc2 | Forkhead box C2 | 4.864 | 7.66E-03 | 2.468* |
| 104349_at | Snip1 | Smad nuclear interacting protein 1 | 4.806 | 8.01E-03 | 1.271 |
| 93701_at | Smarca5 | SWI/SNF related, matrix associated, actin dependent regulator of chromatin, subfamily a, member 5 | 4.760 | 8.30E-03 | 1.512* |
| 93911_at | Tgs1 | Trimethylguanosine synthase homolog (S. cerevisiae) | 4.750 | 8.36E-03 | 1.479 |
| 92332_at | Dlx2 | Distal-less homeobox 2 | 4.689 | 8.71E-03 | 5.057 |
| 103964_at | Esrra | Estrogen related receptor, alpha | 4.622 | 9.17E-03 | 1.209 |
| 92257_at | Clock | Circadian locomoter output cycles kaput | 4.595 | 9.38E-03 | 1.947* |
| 100553_at | Trim27 | Tripartite motif protein 27 | 4.556 | 9.70E-03 | 1.630* |
| 99058_at | Hmga2 | High mobility group AT-hook 2 | 4.549 | 9.76E-03 | 4.673 |
| 94303_at | Hnrpd | Heterogeneous nuclear ribonucleoprotein d | 4.539 | 9.86e-03 | 1.713 |
| 95129_at | Ncor2 | Nuclear receptor co-repressor 2 | 4.488 | 1.02E-02 | 1.945 |
| 94332_at | Ets1 | E26 avian leukemia oncogene 1, 5' domain | 4.441 | 1.06E-02 | 2.930 |
| 97315_at | Polr2d | Polymerase (RNA) II (DNA directed) polypeptide D | 4.436 | 1.07E-02 | 1.488 |
| 102018_at | Cnot3 | CCR4-NOT transcription complex, subunit 3 | 4.436 | 1.07E-02 | 1.282 |
| 100482_at | Zfp598 | Zinc finger protein 598 | 4.432 | 1.07E-02 | 1.591 |
| 93620_at | Rpo1-4 | RNA polymerase 1-4 | 4.405 | 1.09E-02 | 2.361 |
| 98515_at | Gtf2e2 | General transcription factor II E, polypeptide 2 (beta subunit) | 4.356 | 1.14E-02 | 1.749 |
| 103807_at | Wiz | Widely-interspaced zinc finger motifs | 4.356 | 1.14E-02 | 1.495 |
| 92804_at | Polr2h | Polymerase (RNA) II (DNA directed) polypeptide H | 4.237 | 1.25E-02 | 1.628 |
| 104610_at | Pprc1 | Peroxisome proliferative activated receptor, gamma, coactivator-related 1 | 4.230 | 1.25E-02 | 2.062 |
| 93669_f_at | Sox11 | SRY-box containing gene 11 | 4.229 | 1.25E-02 | 1.694 |
| 95596_at | Foxk2 | Forkhead box K2 | 4.205 | 1.28E-02 | 2.177 |
| 101959_r_at | Tfdp1 | Transcription factor Dp 1 | 4.187 | 1.29E-02 | 1.823 |
| 104279_at | Polr2f | Polymerase (RNA) II (DNA directed) polypeptide F | 4.185 | 1.30E-02 | 1.553 |
| 92225_f_at | Rpo1-2 | RNA polymerase 1-2 | 4.182 | 1.30E-02 | 1.264 |
| 93943_f_at | Zfp36l2 | Zinc finger protein 36, C3H type-like 2 | 4.096 | 1.39E-02 | 2.094 |
| 95155_at | Znrf1 | Zinc and ring finger 1 | 4.086 | 1.40E-02 | 1.179 |
| 96711_at | Znrd1 | Zinc ribbon domain containing, 1 | 4.052 | 1.44E-02 | 1.477 |
| 97154_f_at | MGC117846 | Similar to zinc finger protein 665 | 4.044 | 1.45E-02 | 1.873 |
| 93753_at | Litaf | LPS-induced TN factor | 4.010 | 1.49E-02 | 1.838 |
| 103504_at | Ssbp2 | Single-stranded DNA binding protein 2 | -15.382 | 8.01E-05 | 0.178 |
| 161427_f_at | E2f1 | E2F transcription factor 1 | -10.005 | 4.74E-04 | 0.851 |
| 95059_at | Pnrc2 | Proline-rich nuclear receptor coactivator 2 | -8.994 | 8.01E-04 | 0.324* |
| 99052_at | Zfhx1a | Zinc finger homeobox 1a | -8.312 | 1.09E-03 | 0.481 |
| 96940_at | Tead2 | TEA domain family member 2 | -8.129 | 1.17E-03 | 0.542 |
| 95326_at | Tspyl1 | Testis-specific protein, Y-encoded-like 1 | -7.414 | 1.66E-03 | 0.726 |
| 101939_at | Rnf141 | Ring finger protein 141 | -7.302 | 1.74E-03 | 0.681 |
| 161308_f_at | Yap1 | Yes-associated protein 1 | -7.246 | 1.79E-03 | 0.799 |
| 104740_at | Tada1l | Transcriptional adaptor 1 (HFI1 homolog, yeast) like | -7.015 | 2.00E-03 | 0.599 |
| 160780_at | Tcf3 | Transcription factor 3 | -6.663 | 2.42E-03 | 0.501 |
| 160483_at | Tcf4 | Transcription factor 4 | -6.399 | 2.82E-03 | 0.333 |
| 95033_at | Jmjd1a | Jumonji domain containing 1A | -6.366 | 2.89E-03 | 0.510 |
| 94973_at | Jmjd1b | Jumonji domain containing 1B | -5.776 | 4.18E-03 | 0.545 |
| 94261_at | Thrap3 | Thyroid hormone receptor associated protein 3 | -5.702 | 4.37E-03 | 0.731 |
| 96703_at | Maged1 | Melanoma antigen, family D, 1 | -5.578 | 4.78E-03 | 0.594 |
| 92935_at | Runx1t1 | Runt-related transcription factor 1; translocated to, 1 (cyclin D-related) | -5.567 | 4.82E-03 | 0.418 |
| 104010_at | Zfp99 | Zinc finger protein 99 | -5.484 | 5.07E-03 | 0.650 |
| 97917_at | Bloc1s1 | Biogenesis of lysosome-related organelles complex-1, subunit 1 | -5.326 | 5.60E-03 | 0.567 |
| 92990_at | Zfp93 | Zinc finger protein 93 | -5.232 | 5.95E-03 | 0.629 |
| 92974_at | Zfp37 | Zinc finger protein 37 | -5.231 | 5.96E-03 | 0.690 |
| 161741_r_at | Zfp503 | Zinc finger protein 503 | -5.212 | 6.04E-03 | 0.791 |
| 104070_at | Pcaf | p300/CBP-associated factor | -5.191 | 6.13E-03 | 0.203 |
| 94406_at | Phtf1 | Putative homeodomain transcription factor 1 | -5.181 | 6.17E-03 | 0.634 |
| 99034_at | Irx3 | Iroquois related homeobox 3 (Drosophila) | -5.168 | 6.23E-03 | 0.734 |
| 160848_at | Zhx1 | Zinc fingers and homeoboxes protein 1 | -5.162 | 6.26E-03 | 0.329* |
| 94295_at | Gtf2i | General transcription factor II I | -5.136 | 6.38E-03 | 0.591 |
| 98024_at | Nfyb | Nuclear transcription factor-Y beta | -5.014 | 6.91E-03 | 0.687 |
| 92300_at | Mnt | Max binding protein | -4.852 | 7.72E-03 | 0.695 |
| 103450_at | Bcas3 | Breast carcinoma amplified sequence 3 | -4.840 | 7.79E-03 | 0.866 |
| 99076_at | Nr1d2 | Nuclear receptor subfamily 1, group D, member 2 | -4.761 | 8.30E-03 | 0.413 |
| 99465_at | Mecp2 | Methyl CpG binding protein 2 | -4.735 | 8.44E-03 | 0.579 |
| 160138_at | Mxi1 | Max interacting protein 1 | -4.670 | 8.82E-03 | 0.410 |
| 101483_at | Ccndbp1 | Cyclin D-type binding-protein 1 | -4.667 | 8.84E-03 | 0.712 |
| *Primary cell metabolism* | | |  |  |  |
| 100046_at | Mthfd2 | Methylenetetrahydrofolate dehydrogenase (NAD+ dependent), methenyltetrahydrofolate cyclohydrolase | 11.819 | 2.29E-04 | 2.292 |
| 100622_at | Prdx6 | Peroxiredoxin 6 | 8.481 | 1.01E-03 | 1.532 |
| 94367_at | Uck2 | Uridine-cytidine kinase 2 | 8.468 | 1.01E-03 | 3.092* |
| 100026_at | Bcat1 | Branched chain aminotransferase 1, cytosolic | 8.018 | 1.23E-03 | 3.669* |
| 98429_at | Lypla2 | Lysophospholipase 2 | 7.850 | 1.32E-03 | 1.598 |
| 160765_at | Ptges2 | Prostaglandin E synthase 2 | 7.666 | 1.44E-03 | 1.688 |
| 104620_at | Tmem68 | Transmembrane protein 68 | 7.658 | 1.44E-03 | 2.326 |
| 94276_at | Hsd17b12 | Hydroxysteroid (17-beta) dehydrogenase 12 | 7.316 | 1.74E-03 | 2.481* |
| 96827_at | Cad | Carbamoyl-phosphate synthetase 2, aspartate transcarbamylase, and dihydroorotase | 6.982 | 2.04E-03 | 1.615 |
| 99038_at | Adss | Adenylosuccinate synthetase, non muscle | 6.834 | 2.22E-03 | 1.857 |
| 98390_at | Impdh1 | Inosine 5'-phosphate dehydrogenase 1 | 6.560 | 2.55E-03 | 1.745 |
| 160767_at | Soat1 | Sterol O-acyltransferase 1 | 6.200 | 3.22E-03 | 6.105* |
| 95887_at | Soat1 | Sterol O-acyltransferase 1 | 6.189 | 3.25E-03 | 4.235* |
| 94052_at | Dpm2 | Dolichol-phosphate (beta-D) mannosyltransferase 2 | 6.155 | 3.32E-03 | 1.399 |
| 96139_at | Gcs1 | Glucosidase 1 | 6.080 | 3.49E-03 | 1.546 |
| 160289_s_at | Dlst | Dihydrolipoamide S-succinyltransferase (E2 component of 2-oxo-glutarate complex) | 5.792 | 4.14E-03 | 1.593 |
| 93991_at | Mdh2 | Malate dehydrogenase 2, NAD (mitochondrial) | 5.729 | 4.28E-03 | 1.602 |
| 92553_at | Esd | Esterase D/formylglutathione hydrolase | 5.497 | 5.02E-03 | 1.660 |
| 94260_at | Larp1 | La ribonucleoprotein domain family, member 1 | 5.364 | 5.47E-03 | 1.570 |
| 96295_at | Psat1 | Phosphoserine aminotransferase 1 | 5.335 | 5.56E-03 | 1.934 |
| 99039_g_at | Adss | Adenylosuccinate synthetase, non muscle | 5.293 | 5.71E-03 | 2.235 |
| 160385_at | Gcsh | Glycine cleavage system protein H (aminomethyl carrier) | 5.242 | 5.91E-03 | 2.550 |
| 93097_at | Arg1 | Arginase 1, liver | 5.049 | 6.77E-03 | 1.417 |
| 92589_at | Psph | Phosphoserine phosphatase | 5.023 | 6.87E-03 | 2.347 |
| 92335_at | Ltbp2 | Latent transforming growth factor beta binding protein 2 | 5.014 | 6.90E-03 | 3.364 |
| 95133_at | Asns | Asparagine synthetase | 5.005 | 6.98E-03 | 1.629 |
| 101489_at | Amd1 | S-adenosylmethionine decarboxylase 1 | 4.846 | 7.78E-03 | 2.434 |
| 160084_at | Odc1 | Ornithine decarboxylase, structural 1 | 4.806 | 8.01E-03 | 2.193 |
| 101002_at | Azin1 | Antizyme inhibitor 1 | 4.704 | 8.62E-03 | 1.913* |
| 97924_at | Gne | Glucosamine | 4.698 | 8.65E-03 | 1.265 |
| 160850_at | Fpgs | Folylpolyglutamyl synthetase | 4.622 | 9.18E-03 | 1.828 |
| 104647_at | Ptgs2 | Prostaglandin-endoperoxide synthase 2 | 4.620 | 9.19E-03 | 6.601* |
| 95404_at | Pafah1b2 | Platelet-activating factor acetylhydrolase, isoform 1b, alpha2 subunit | 4.614 | 9.25E-03 | 1.699 |
| 160107_at | Hprt1 | Hypoxanthine guanine phosphoribosyl transferase 1 | 4.601 | 9.32e-03 | 1.764* |
| 101466_at | Pigf | Phosphatidylinositol glycan, class F | 4.543 | 9.82E-03 | 1.407 |
| 98372_at | Aldh1a3 | Aldehyde dehydrogenase family 1, subfamily A3 | 4.364 | 1.13E-02 | 4.122 |
| 97880_at | Dlst | Dihydrolipoamide S-succinyltransferase (E2 component of 2-oxo-glutarate complex) | 4.339 | 1.15E-02 | 1.687 |
| 99078_at | Dohh | Deoxyhypusine hydroxylase/monooxygenase | 4.268 | 1.22E-02 | 1.822 |
| 98071_f_at | Dck | Deoxycytidine kinase | 4.161 | 1.32E-02 | 1.494 |
| 94375_at | Hk2 | Hexokinase 2 | 4.155 | 1.33E-02 | 3.015* |
| 98593_at | Cmas | Cytidine monophospho-N-acetylneuraminic acid synthetase | 4.152 | 1.33E-02 | 1.419 |
| 160798_at | Mecr | Mitochondrial trans-2-enoyl-CoA reductase | 4.135 | 1.35E-02 | 1.351 |
| 103924_at | Agpat5 | 1-acylglycerol-3-phosphate O-acyltransferase 5 (lysophosphatidic acid acyltransferase, epsilon) | 4.080 | 1.41E-02 | 1.420 |
| 160134_at | Adipor1 | Adiponectin receptor 1 | 4.055 | 1.43E-02 | 1.342 |
| 101426_at | Cerk | Ceramide kinase | 4.027 | 1.47E-02 | 1.118 |
| 94850_at | Acot10 | Acyl-CoA thioesterase 10 | 4.007 | 1.49E-02 | 1.753 |
| 161997_f_at | Aldh2 | Aldehyde dehydrogenase 2, mitochondrial | -10.265 | 4.16E-04 | 0.559 |
| 94365_at | Hint2 | Histidine triad nucleotide binding protein 2 | -9.447 | 6.41E-04 | 0.648 |
| 161738_f_at | Ilvbl | Ilvb (bacterial acetolactate synthase)-like | -6.987 | 2.04E-03 | 0.664 |
| 92261_at | Coq4 | Coenzyme Q4 homolog (yeast) | -6.786 | 2.27E-03 | 0.802 |
| 100042_at | Hagh | Hydroxyacyl glutathione hydrolase | -6.509 | 2.63E-03 | 0.583 |
| 93026_at | Mgst1 | Microsomal glutathione S-transferase 1 | -6.441 | 2.75E-03 | 0.162 |
| 161243_f_at | Abhd8 | Abhydrolase domain containing 8 | -6.435 | 2.75E-03 | 0.616 |
| 160482_at | Acaa1 | Acetyl-Coenzyme A acyltransferase 1 | -6.395 | 2.83E-03 | 0.446 |
| 94428_at | Ilvbl | Ilvb (bacterial acetolactate synthase)-like | -6.243 | 3.15E-03 | 0.589 |
| 93754_at | Ech1 | Enoyl coenzyme A hydratase 1, peroxisomal | -6.138 | 3.35E-03 | 0.273 |
| 160807_at | Agpat3 | 1-acylglycerol-3-phosphate O-acyltransferase 3 | -6.059 | 3.54E-03 | 0.636 |
| 97515_at | Hsd17b4 | Hydroxysteroid (17-beta) dehydrogenase 4 | -5.808 | 4.11E-03 | 0.428* |
| 160204_at | Prr6 | Proline-rich polypeptide 6 | -5.806 | 4.12E-03 | 0.535 |
| 92341_at | B3galt2 | UDP-Gal:betaGlcNAc beta 1,3-galactosyltransferase, polypeptide 2 | -5.778 | 4.18E-03 | 0.863 |
| 98527_at | Dci | Dodecenoyl-Coenzyme A delta isomerase (3,2 trans-enoyl-Coenyme A isomerase) | -5.716 | 4.32E-03 | 0.543 |
| 104509_at | Ch25h | Cholesterol 25-hydroxylase | -5.646 | 4.55E-03 | 0.465* |
| 93308_s_at | Pcx | Pyruvate carboxylase | -5.641 | 4.56E-03 | 0.479 |
| 102026_s_at | Chkb | Choline kinase beta | -5.634 | 4.58E-03 | 0.788 |
| 102004_at | Acaa1 | Acetyl-Coenzyme A acyltransferase 1 | -5.511 | 4.99E-03 | 0.788 |
| 100630_f_at | Gstm5 | Glutathione s-transferase, mu 5 | -5.483 | 5.07e-03 | 0.533 |
| 162262_f_at | Gyg1 | Glycogenin 1 | -5.370 | 5.46E-03 | 0.286 |
| L09192_3_at | Pcx | Pyruvate carboxylase | -5.367 | 5.46E-03 | 0.462 |
| 103068_at | Akr1e1 | Aldo-keto reductase family 1, member E1 | -5.297 | 5.70E-03 | 0.811 |
| 97971_at | Phkg2 | Phosphorylase kinase, gamma 2 (testis) | -5.275 | 5.78E-03 | 0.815 |
| 98588_at | Fah | Fumarylacetoacetate hydrolase | -5.207 | 6.06E-03 | 0.582 |
| 100099_at | Smpd1 | Sphingomyelin phosphodiesterase 1, acid lysosomal | -5.190 | 6.14E-03 | 0.535 |
| 101000_at | Oaz2 | Ornithine decarboxylase antizyme 2 | -5.177 | 6.19E-03 | 0.410 |
| 93868_at | Nsdhl | NAD(P) dependent steroid dehydrogenase-like | -5.177 | 6.19E-03 | 0.369 |
| 93933_at | Ppp1r3c | Protein phosphatase 1, regulatory (inhibitor) subunit 3C | -5.057 | 6.73E-03 | 0.769 |
| 92581_at | Acadm | Acetyl-Coenzyme A dehydrogenase, medium chain | -5.052 | 6.75E-03 | 0.509 |
| 99543_s_at | Dguok | Deoxyguanosine kinase | -4.941 | 7.26E-03 | 0.766 |
| 95426_at | Echs1 | Enoyl Coenzyme A hydratase, short chain, 1, mitochondrial | -4.837 | 7.80E-03 | 0.491 |
| 94485_at | Peci | Peroxisomal delta3, delta2-enoyl-Coenzyme A isomerase | -4.793 | 8.09E-03 | 0.464 |
| 94438_at | Pfkm | Phosphofructokinase, muscle | -4.789 | 8.11E-03 | 0.675 |
| 97450_s_at | Aldh7a1 | Aldehyde dehydrogenase family 7, member A1 | -4.739 | 8.42E-03 | 0.668 |
| 93642_at | Gdf1 | Growth differentiation factor 1 | -4.696 | 8.66E-03 | 0.687 |
| 100629_at | Gstm5 | Glutathione s-transferase, mu 5 | -4.634 | 9.07e-03 | 0.428 |
| 160652_at | Ctps2 | Cytidine 5'-triphosphate synthase 2 | -4.551 | 9.75E-03 | 0.385 |
| 101515_at | Acox1 | Acyl-Coenzyme A oxidase 1, palmitoyl | -4.548 | 9.77E-03 | 0.746 |
| 99498_at | Glul | Glutamate-ammonia ligase (glutamine synthase) | -4.547 | 9.78E-03 | 0.808 |
| 97385_at | Nagk | N-acetylglucosamine kinase | -4.531 | 9.92E-03 | 0.635 |
| 160579_at | Man1a | Mannosidase 1, alpha | -4.523 | 9.98E-03 | 0.371 |
| *Transport and trafficking processes* | | |  |  |  |
| 160409_at | Pitpna | Phosphatidylinositol transfer protein, alpha | 12.196 | 1.88E-04 | 1.376 |
| 95889_at | Arf3 | ADP-ribosylation factor 3 | 9.904 | 4.96E-04 | 1.339 |
| 103286_at | Stam2 | Signal transducing adaptor molecule (SH3 domain and ITAM motif) 2 | 9.439 | 6.43E-04 | 1.466 |
| 104221_at | Slc7a5 | Solute carrier family 7 (cationic amino acid transporter, y+ system), member 5 | 9.006 | 7.94E-04 | 2.310 |
| 92398_at | Vps37b | Vacuolar protein sorting 37B (yeast) | 8.805 | 8.74E-04 | 1.843 |
| 101370_at | Kpna1 | Karyopherin (importin) alpha 1 | 8.170 | 1.14E-03 | 2.482 |
| 97551_at | Hip1r | Huntingtin interacting protein 1 related | 7.688 | 1.42E-03 | 1.589* |
| 103527_at | Slc35e4 | Solute carrier family 35, member E4 | 7.614 | 1.48E-03 | 1.880 |
| 99329_at | Abcc1 | ATP-binding cassette, sub-family C (CFTR/MRP), member 1 | 7.150 | 1.88E-03 | 1.842 |
| 104510_at | Cacna2d1 | Calcium channel, voltage-dependent, alpha2/delta subunit 1 | 7.121 | 1.91E-03 | 1.323 |
| 104145_at | Tcof1 | Treacher Collins Franceschetti syndrome 1, homolog | 7.088 | 1.94E-03 | 2.448 |
| 94419_at | Slc19a1 | Solute carrier family 19 (sodium/hydrogen exchanger), member 1 | 6.622 | 2.47E-03 | 2.048* |
| 96760_at | Timm9 | Translocase of inner mitochondrial membrane 9 homolog (yeast) | 6.461 | 2.72E-03 | 2.210 |
| 95432_f_at | Tomm70a | Translocase of outer mitochondrial membrane 70 homolog A (yeast) | 6.417 | 2.80E-03 | 2.474 |
| 103681_at | Atp6v0a2 | ATPase, H+ transporting, lysosomal V0 subunit a isoform 2 | 6.212 | 3.21E-03 | 2.255* |
| 100568_at | Abce1 | ATP-binding cassette, sub-family E (OABP), member 1 | 6.151 | 3.32E-03 | 2.003 |
| 160543_at | Snx3 | Sorting nexin 3 | 5.699 | 4.38E-03 | 1.443* |
| 92840_at | Nup54 | Nucleoporin 54 | 5.614 | 4.65E-03 | 1.548 |
| 93993_at | Lman2 | Lectin, mannose-binding 2 | 5.535 | 4.91E-03 | 1.498 |
| 103394_at | Fxyd5 | FXYD domain-containing ion transport regulator 5 | 5.438 | 5.21E-03 | 2.603* |
| 96919_at | Atp6v0c | ATPase, H+ transporting, V0 subunit C | 5.434 | 5.23E-03 | 1.684 |
| 93845_at | Abcf2 | ATP-binding cassette, sub-family F (GCN20), member 2 | 5.353 | 5.51E-03 | 1.842 |
| 96174_at | Pom121 | Nuclear pore membrane protein 121 | 5.314 | 5.64E-03 | 1.495* |
| 100948_at | Ank | Progressive ankylosis | 5.153 | 6.31E-03 | 3.475* |
| 98104_at | Atp6v0b | ATPase, H+ transporting, V0 subunit B | 5.106 | 6.50E-03 | 1.571 |
| 97471_at | Armc1 | Armadillo repeat containing 1 | 4.950 | 7.22E-03 | 1.589 |
| 102273_at | Atp6v0a2 | ATPase, H+ transporting, lysosomal V0 subunit a isoform 2 | 4.924 | 7.36E-03 | 1.661* |
| 98975_at | Nup93 | Nucleoporin 93 | 4.823 | 7.89E-03 | 1.812 |
| 96010_at | Kpna3 | Karyopherin (importin) alpha 3 | 4.704 | 8.61E-03 | 1.530 |
| 92428_at | Asna1 | Arsa (bacterial) arsenite transporter, ATP-binding, homolog 1 | 4.681 | 8.76E-03 | 1.762 |
| 95075_at | Nup35 | Nucleoporin 35 | 4.592 | 9.40E-03 | 1.552 |
| 104007_at | Slc25a15 | Solute carrier family 25 (mitochondrial carrier ornithine transporter), member 15 | 4.377 | 1.12E-02 | 1.403 |
| 99579_at | Atp1b3 | ATPase, Na+/K+ transporting, beta 3 polypeptide | 4.185 | 1.30E-02 | 1.523 |
| 99146_at | Stx6 | Syntaxin 6 | 4.142 | 1.34E-02 | 1.566 |
| 92872_at | Necap1 | NECAP endocytosis associated 1 | 4.142 | 1.34E-02 | 1.454 |
| 160960_at | Atp6v0a2 | ATPase, H+ transporting, lysosomal V0 subunit a isoform 2 | 4.129 | 1.35E-02 | 1.626* |
| 94254_at | Clic4 | Chloride intracellular channel 4 (mitochondrial) | 4.114 | 1.37E-02 | 1.783 |
| 98386_s_at | Cacna1c | Calcium channel, voltage-dependent, L type, alpha 1C subunit | 4.060 | 1.43E-02 | 1.413 |
| 93466_at | Exoc4 | Exocyst complex component 4 | -14.849 | 8.92E-05 | 0.534 |
| 99187_f_at | Use1 | Unconventional SNARE in the ER 1 homolog (S. cerevisiae) | -10.978 | 3.20E-04 | 0.339 |
| 103854_at | Arl2 | ADP-ribosylation factor-like 2 | -10.082 | 4.53E-04 | 0.591 |
| 93609_at | Spg20 | Spastic paraplegia 20, spartin (Troyer syndrome) homolog (human) | -9.491 | 6.27E-04 | 0.797 |
| 95490_at | Kdelr1 | KDEL (Lys-Asp-Glu-Leu) endoplasmic reticulum protein retention receptor 1 | -8.831 | 8.63E-04 | 0.599 |
| 104264_at | Lrba | LPS-responsive beige-like anchor | -7.782 | 1.37E-03 | 0.577 |
| 162426_f_at | Scamp1 | Secretory carrier membrane protein 1 | -7.324 | 1.73E-03 | 0.763 |
| 96894_at | Tmed4 | Transmembrane emp24 protein transport domain containing 4 | -7.088 | 1.94E-03 | 0.593 |
| 102786_at | Clcn3 | Chloride channel 3 | -6.606 | 2.49E-03 | 0.658 |
| 99188_at | Use1 | Unconventional SNARE in the ER 1 homolog (S. cerevisiae) | -6.251 | 3.13E-03 | 0.330 |
| 94556_at | Snx10 | Sorting nexin 10 | -5.822 | 4.09E-03 | 0.532 |
| 92582_at | Slc1a5 | Solute carrier family 1 (neutral amino acid transporter), member 5 | -5.774 | 4.19E-03 | 0.478 |
| 160371_at | Arl6ip1 | ADP-ribosylation factor-like 6 interacting protein 1 | -5.765 | 4.21E-03 | 0.421 |
| 94465_g_at | Clcn3 | Chloride channel 3 | -4.933 | 7.30E-03 | 0.728 |
| 104514_at | Epn1 | Epsin 1 | -4.757 | 8.33E-03 | 0.683 |
| 94244_at | Ihpk1 | Inositol hexaphosphate kinase 1 | -4.588 | 9.44E-03 | 0.611 |
| 162092_f_at | Ihpk1 | Inositol hexaphosphate kinase 1 | -5.520 | 4.96E-03 | 0.778 |
| 95602_at | Trpc4ap | Transient receptor potential cation channel, subfamily C, member 4 associated protein | -5.456 | 5.14E-03 | 0.800 |
| 102670_at | Vps54 | Vacuolar protein sorting 54 (yeast) | -5.288 | 5.73E-03 | 0.777 |
| 99845_at | Slc1a6 | Solute carrier family 1 (high affinity aspartate/glutamate transporter), member 6 | -5.250 | 5.89E-03 | 0.524 |
| 104648_at | Pacs1 | Phosphofurin acidic cluster sorting protein 1 | -5.046 | 6.78E-03 | 0.750 |
| 96529_at | Ap1gbp1 | AP1 gamma subunit binding protein 1 | -4.809 | 7.99E-03 | 0.794 |
| 103745_at | Snx13 | Sorting nexin 13 | -4.740 | 8.41E-03 | 0.777 |
| *Cell cycle and DNA replication* | | |  |  |  |
| 96726_at | Cdk8 | Cyclin-dependent kinase 8 | 11.847 | 2.27E-04 | 2.284* |
| 99138_at | Rcc1 | Regulator of chromosome condensation 1 | 8.863 | 8.56E-04 | 3.291* |
| 95122_g_at | Pole4 | Polymerase (DNA-directed), epsilon 4 (p12 subunit) | 8.165 | 1.14E-03 | 1.546 |
| 92879_at | Ppm1g | Protein phosphatase 1G (formerly 2C), magnesium-dependent, gamma isoform | 7.984 | 1.25E-03 | 2.039 |
| 95121_at | Pole4 | Polymerase (DNA-directed), epsilon 4 (p12 subunit) | 6.975 | 2.05E-03 | 2.057 |
| 95063_at | Cdca7 | Cell division cycle associated 7 | 6.897 | 2.14E-03 | 3.200 |
| 98532_at | Cdk2ap1 | CDK2 (cyclin-dependent kinase 2)-associated protein 1 | 6.244 | 3.15E-03 | 1.769 |
| 101492_at | Pin1 | Protein (peptidyl-prolyl cis/trans isomerase) NIMA-interacting 1 | 5.984 | 3.71E-03 | 1.603* |
| 100413_at | Ylpm1 | YLP motif containing 1 | 5.782 | 4.17E-03 | 1.234 |
| 96200_at | Cdca4 | Cell division cycle associated 4 | 4.544 | 9.81E-03 | 1.578* |
| 95712_at | Orc6l | Origin recognition complex, subunit 6-like (S. Cerevisiae) | 5.528 | 4.93E-03 | 1.732 |
| 160842_at | Kin | Antigenic determinant of rec-A protein | 5.289 | 5.72E-03 | 1.260 |
| 97327_at | Fen1 | Flap structure specific endonuclease 1 | 4.805 | 8.02E-03 | 2.316 |
| 104096_at | Orc4l | Origin recognition complex, subunit 4-like (S. Cerevisiae) | 4.597 | 9.35E-03 | 1.751 |
| 103821_at | Cdc6 | Cell division cycle 6 homolog (S. Cerevisiae) | 4.396 | 1.10E-02 | 1.776 |
| 161651_f_at | Orc2l | Origin recognition complex, subunit 2-like (S. Cerevisiae) | 4.348 | 1.15E-02 | 1.226 |
| 93112_at | Mcm2 | Minichromosome maintenance deficient 2 mitotin (S. Cerevisiae) | 4.014 | 1.48E-02 | 1.712 |
| 99541_at | Kif11 | Kinesin family member 11 | -11.468 | 2.61E-04 | 0.528 |
| 94294_at | Ccnb2 | Cyclin B2 | -8.958 | 8.19E-04 | 0.360 |
| 95100_at | Anapc5 | Anaphase-promoting complex subunit 5 | -7.575 | 1.53E-03 | 0.646 |
| 96710_at | H2afv | H2A histone family, member V | -7.486 | 1.61E-03 | 0.355 |
| 100278_at | Cdkn1b | Cyclin-dependent kinase inhibitor 1B (P27) | -6.742 | 2.32E-03 | 0.737 |
| 93635_at | Kif3c | Kinesin family member 3C | -5.792 | 4.14E-03 | 0.744 |
| 95032_at | Prc1 | Protein regulator of cytokinesis 1 | -5.392 | 5.38E-03 | 0.624 |
| 93699_at | Polg2 | Polymerase (DNA directed), gamma 2, accessory subunit | -5.161 | 6.26E-03 | 0.825 |
| 161856_f_at | Kif20a | Kinesin family member 20A | -5.010 | 6.95E-03 | 0.561 |
| 102395_at | Pmp22 | Peripheral myelin protein | -4.908 | 7.45E-03 | 0.252 |
| 160501_at | Kif20a | Kinesin family member 20A | -4.775 | 8.21E-03 | 0.534 |
| 95419_at | H1f0 | H1 histone family, member 0 | -4.756 | 8.34E-03 | 0.265* |
| 100612_at | Rrm1 | Ribonucleotide reductase M1 | -4.641 | 9.02E-03 | 0.809 |
| *Immunity and defense* | | |  |  |  |
| 98501_at | Il1rl1 | Interleukin 1 receptor-like 1 | 15.838 | 6.63E-05 | 19.662 |
| 102209_at | Nfatc1 | Nuclear factor of activated T-cells, cytoplasmic, calcineurin-dependent 1 | 10.368 | 3.82E-04 | 2.193* |
| 103975_at | Grem2 | Gremlin 2 homolog, cysteine knot superfamily (Xenopus laevis) | 5.530 | 4.92E-03 | 8.400* |
| 161046_at | Crlf1 | Cytokine receptor-like factor 1 | 4.934 | 7.29E-03 | 5.397 |
| 93861_f_at | Ctse | Cathepsin E | 4.627 | 9.12E-03 | 1.401 |
| 93860_i_at | Ctse | Cathepsin E | 4.446 | 1.06E-02 | 1.374 |
| 103563_at | Ibrdc3 | IBR domain containing 3 | 4.262 | 1.22E-02 | 2.054 |
| 99010_at | Islr | Immunoglobulin superfamily containing leucine-rich repeat | -13.054 | 1.35E-04 | 0.098 |
| 101495_at | Cd81 | CD 81 antigen | -7.232 | 1.81E-03 | 0.472 |
| 99491_at | Il10rb | Interleukin 10 receptor, beta | -5.673 | 4.47E-03 | 0.559 |
| 97718_at | Ctla4 | Cytotoxic T-lymphocyte-associated protein 4 | -5.618 | 4.64E-03 | 0.843 |
| 93874_s_at | Il11ra2 | Interleukin 11 receptor, alpha chain 2 | -5.355 | 5.50E-03 | 0.297 |
| 94345_at | Il6st | Interleukin 6 signal transducer | -5.318 | 5.63E-03 | 0.286 |
| 98349_at | Il6st | Interleukin 6 signal transducer | -4.566 | 9.62E-03 | 0.371 |
| 160657_at | Ilf3 | Interleukin enhancer binding factor 3 | -4.557 | 9.69E-03 | 0.575 |
| *Response to IFN* | | |  |  |  |
| 92287_at | Socs5 | Suppressor of cytokine signaling 5 | 5.282 | 5.75E-03 | 1.844 |
| 94774_at | Ifi202b | Interferon activated gene 202B | -5.479 | 5.08E-03 | 0.104 |
| 101014_at | Ifnar2 | Interferon (alpha and beta) receptor 2 | -4.795 | 8.07E-03 | 0.473 |
| *Cell adhesion and migration* | | |  |  |  |
| 94724_at | Mmp10 | Matrix metallopeptidase 10 | 9.024 | 7.92E-04 | 1.540 |
| 96187_at | Pkp4 | Plakophilin 4 | 8.244 | 1.11E-03 | 2.235 |
| 94896_at | Hnrpab | Heterogeneous nuclear ribonucleoprotein A/B | 7.898 | 1.30E-03 | 1.498 |
| 94643_at | Pvr | Poliovirus receptor | 7.688 | 1.42E-03 | 2.864 |
| 99663_g_at | Nup85 | Nucleoporin 85 | 7.261 | 1.77E-03 | 1.609 |
| 93383_at | Col7a1 | Procollagen, type VII, alpha 1 | 7.107 | 1.93E-03 | 4.160 |
| 94826_at | Itgb4bp | Integrin beta 4 binding protein | 6.922 | 2.10E-03 | 2.166* |
| 97875_at | Adrm1 | Adhesion regulating molecule 1 | 6.622 | 2.47E-03 | 1.943 |
| 99996_at | Pkp1 | Plakophilin 1 | 6.527 | 2.60E-03 | 2.012 |
| 160227_s_at | Bysl | Bystin-like | 6.386 | 2.85E-03 | 2.449 |
| 101993_at | Tnc | Tenascin C | 6.251 | 3.13E-03 | 5.467* |
| 101040_at | Capn2 | Calpain 2 | 5.644 | 4.55E-03 | 2.157 |
| 103039_at | Itga5 | Integrin alpha 5 (fibronectin receptor alpha) | 5.639 | 4.56E-03 | 6.002 |
| 92414_at | Adam12 | A disintegrin and metallopeptidase domain 12 (meltrin alpha) | 5.458 | 5.14E-03 | 2.171* |
| 97822_at | Pak2 | p21 (CDKN1A)-activated kinase 2 | 5.225 | 5.98E-03 | 1.639 |
| 97832_at | Cd97 | CD97 antigen | 5.117 | 6.46E-03 | 1.336 |
| 100124_r_at | Itgb1 | Integrin beta 1 (fibronectin receptor beta) | 5.095 | 6.55E-03 | 1.830 |
| 97904_at | Actr3 | ARP3 actin-related protein 3 homolog (yeast) | 4.805 | 8.02E-03 | 2.102 |
| 99662_at | Nup85 | Nucleoporin 85 | 4.757 | 8.32E-03 | 1.879 |
| 97823_g_at | Pak2 | p21 (CDKN1A)-activated kinase 2 | 4.313 | 1.18E-02 | 1.454 |
| 95643_at | Wdr6 | WD repeat domain 6 | -7.383 | 1.68E-03 | 0.501 |
| 98614_at | Nphp1 | Nephronophthisis 1 (juvenile) homolog (human) | -7.136 | 1.90E-03 | 0.650 |
| 101843_at | Sh2b1 | SH2-B adaptor protein 1 | -6.121 | 3.38E-03 | 0.657 |
| 99014_at | Apbb1 | Amyloid beta (A4) precursor protein-binding, family B, member 1 | -5.876 | 3.96E-03 | 0.605 |
| 161247_f_at | Dgcr2 | DiGeorge syndrome critical region gene 2 | -4.937 | 7.29E-03 | 0.684 |
| 160932_at | Nck1 | Non-catalytic region of tyrosine kinase adaptor protein 1 | -4.690 | 8.70E-03 | 0.843 |
| 98848_at | Sorbs3 | Sorbin and SH3 domain containing 3 | -4.606 | 9.30E-03 | 0.589 |
| 96865_at | Marcks | Myristoylated alanine rich protein kinase C substrate | -4.550 | 9.76E-03 | 0.202 |
| 100142_at | Dgcr2 | DiGeorge syndrome critical region gene 2 | -4.535 | 9.90E-03 | 0.791 |
| *RNA metabolism and processing* | | |  |  |  |
| 94450_at | Nsun2 | NOL1/NOP2/Sun domain family 2 | 16.267 | 5.26E-05 | 3.904 |
| 94452_g_at | Nsun2 | NOL1/NOP2/Sun domain family 2 | 16.085 | 5.95E-05 | 3.631 |
| 93518_at | Rnps1 | Ribonucleic acid binding protein S1 | 12.914 | 1.40E-04 | 1.791 |
| 160492_at | Ddx18 | DEAD (Asp-Glu-Ala-Asp) box polypeptide 18 | 12.866 | 1.42E-04 | 2.497 |
| 99954_at | Rnu3ip2 | RNA, U3 small nucleolar interacting protein 2 | 11.888 | 2.22E-04 | 2.265 |
| 101371_at | Cpsf4 | Cleavage and polyadenylation specific factor 4 | 10.194 | 4.26E-04 | 1.669* |
| 95685_at | Wdr74 | WD repeat domain 74 | 9.772 | 5.40E-04 | 1.833 |
| 102385_at | Wdr43 | WD repeat domain 43 | 8.599 | 9.49E-04 | 2.743 |
| 104766_at | Nola1 | Nucleolar protein family A, member 1 (H/ACA small nucleolar rnps) | 8.137 | 1.16E-03 | 2.643* |
| 98923_at | Rcl1 | RNA terminal phosphate cyclase-like 1 | 7.683 | 1.43E-03 | 2.232 |
| 101114_at | Srprb | Signal recognition particle receptor, B subunit | 7.482 | 1.62E-03 | 1.484 |
| 94292_at | Strap | Serine/threonine kinase receptor associated protein | 7.205 | 1.83E-03 | 2.110 |
| 103056_at | Wdr50 | WD repeat domain 50 | 7.152 | 1.88E-03 | 2.468 |
| 97486_at | U2af1 | U2 small nuclear ribonucleoprotein auxiliary factor (U2AF) 1 | 7.143 | 1.89E-03 | 2.302 |
| 104586_at | Sfrs1 | Splicing factor, arginine/serine-rich 1 (ASF/SF2) | 7.057 | 1.97E-03 | 2.690 |
| 161060_i_at | Ddx51 | DEAD (Asp-Glu-Ala-Asp) box polypeptide 51 | 7.003 | 2.02E-03 | 2.175 |
| 104212_at | Lrpprc | Leucine-rich PPR-motif containing | 6.872 | 2.17E-03 | 2.337* |
| 92839_f_at | Snrpb2 | U2 small nuclear ribonucleoprotein B | 6.513 | 2.62E-03 | 1.372 |
| 95428_at | Imp4 | IMP4, U3 small nucleolar ribonucleoprotein, homolog (yeast) | 6.342 | 2.95E-03 | 1.674 |
| 103932_at | Wdsof1 | WD repeats and SOF domain containing 1 | 6.175 | 3.28E-03 | 2.209 |
| 100917_at | Wdr55 | WD repeat domain 55 | 6.054 | 3.56E-03 | 2.002 |
| 96245_at | Ddx56 | DEAD (Asp-Glu-Ala-Asp) box polypeptide 56 | 6.038 | 3.59E-03 | 1.641 |
| 95649_at | Phf5a | PHD finger protein 5A | 5.653 | 4.53E-03 | 1.893 |
| 99182_at | Rpp14 | Ribonuclease P 14 subunit (human) | 5.650 | 4.54E-03 | 1.631 |
| 97845_at | Wdr5 | WD repeat domain 5 | 5.620 | 4.63E-03 | 1.337 |
| 102409_at | Lsm8 | LSM8 homolog, U6 small nuclear RNA associated (S. Cerevisiae) | 5.444 | 5.19E-03 | 1.878 |
| 100577_at | Snrpd1 | Small nuclear ribonucleoprotein D1 | 5.436 | 5.22E-03 | 1.599* |
| 94972_at | Rbms1 | RNA binding motif, single stranded interacting protein 1 | 5.399 | 5.35E-03 | 1.461 |
| 95756_at | Ftsj3 | Ftsj homolog 3 (E. Coli) | 5.342 | 5.55E-03 | 2.809 |
| 160550_i_at | Magoh | Mago-nashi homolog, proliferation-associated (Drosophila) | 5.311 | 5.65E-03 | 1.820 |
| 99621_s_at | Sfpq | Splicing factor proline/glutamine rich (polypyrimidine tract binding protein associated) | 5.268 | 5.82E-03 | 2.167* |
| 96946_at | Lsm1 | LSM1 homolog, U6 small nuclear RNA associated (S. Cerevisiae) | 5.186 | 6.15E-03 | 1.297 |
| 160847_at | Trnt1 | tRNA nucleotidyl transferase, CCA-adding, 1 | 5.093 | 6.56E-03 | 1.786 |
| 102400_at | AU014645 | Expressed sequence AU014645 | 5.075 | 6.63E-03 | 2.050 |
| 104276_at | Glipr2 | GLI pathogenesis-related 2 | 5.050 | 6.76E-03 | 1.306 |
| 99151_at | Exosc7 | Exosome component 7 | 4.891 | 7.53E-03 | 1.292 |
| 93630_at | Cugbp1 | CUG triplet repeat, RNA binding protein 1 | 4.806 | 8.01E-03 | 1.623 |
| 160296_at | Wsb2 | WD repeat and SOCS box-containing 2 | 4.741 | 8.41E-03 | 2.312 |
| 96136_at | Exosc10 | Exosome component 10 | 4.637 | 9.05E-03 | 1.369 |
| 97517_at | Exosc4 | Exosome component 4 | 4.543 | 9.82E-03 | 1.215 |
| 93309_at | Ddx3x | DEAD/H (Asp-Glu-Ala-Asp/His) box polypeptide 3, X-linked | 4.535 | 9.89E-03 | 2.159 |
| 104399_at | Cstf2 | Cleavage stimulation factor, 3' pre-RNA subunit 2 | 4.477 | 1.03E-02 | 1.866 |
| 95650_at | Nhp2l1 | NHP2 non-histone chromosome protein 2-like 1 (S. Cerevisiae) | 4.476 | 1.03E-02 | 1.902 |
| 160531_at | Emg1 | EMG1 nucleolar protein homolog (S. cerevisiae) | 4.419 | 1.08E-02 | 1.782 |
| 100037_at | Ddx18 | DEAD (Asp-Glu-Ala-Asp) box polypeptide 18 | 4.385 | 1.11E-02 | 2.623 |
| 96027_at | Sf3a1 | Splicing factor 3a, subunit 1 | 4.356 | 1.14E-02 | 1.575 |
| 98048_at | Fusip1 | FUS interacting protein (serine-arginine rich) 1 | 4.297 | 1.19E-02 | 1.532 |
| 97166_at | Elac2 | Elac homolog 2 (E. Coli) | 4.224 | 1.26E-02 | 1.409 |
| 95677_at | Wdr57 | WD repeat domain 57 (U5 snRNP specific) | 4.127 | 1.36E-02 | 1.787 |
| 160078_at | Ppp1r14b | Protein phosphatase 1, regulatory (inhibitor) subunit 14B | 4.047 | 1.45E-02 | 1.812 |
| 103525_at | Hnrpll | Heterogeneous nuclear ribonucleoprotein l-like | 4.024 | 1.48e-02 | 1.297 |
| 97903_at | Tsen34 | tRNA splicing endonuclease 34 homolog (SEN34, S. cerevisiae) | -6.283 | 3.05E-03 | 0.555 |
| 104231_at | Hnrph3 | Heterogeneous nuclear ribonucleoprotein h3 | -5.805 | 4.12e-03 | 0.609 |
| 97536_at | Wdtc1 | WD and tetratricopeptide repeats 1 | -5.308 | 5.66E-03 | 0.674 |
| 94067_at | Dcps | Decapping enzyme, scavenger | -5.119 | 6.45E-03 | 0.613 |
| 96650_at | Auh | AU RNA binding protein/enoyl-coenzyme A hydratase | -5.093 | 6.56E-03 | 0.593 |
| 97848_at | Rbmx | RNA binding motif protein, X chromosome | -4.685 | 8.72E-03 | 0.704 |
| 103414_at | Skiv2l | Superkiller viralicidic activity 2-like (S. Cerevisiae ) | -4.521 | 9.99E-03 | 0.699 |
| *Cell development and differentiation* | | |  |  |  |
| 97273_at | MGI:1933527 | Arsenate resistance protein 2 | 11.262 | 2.95E-04 | 1.368 |
| 94121_at | Sprr2h | Small proline-rich protein 2H | 9.687 | 5.70E-04 | 4.126* |
| 103830_at | Snai1 | Snail homolog 1 (Drosophila) | 9.373 | 6.68E-04 | 4.898 |
| 104614_at | Gpc1 | Glypican 1 | 8.412 | 1.04E-03 | 2.821 |
| 102375_at | Smyd5 | SET and MYND domain containing 5 | 8.212 | 1.13E-03 | 1.656 |
| 93460_at | Acvr1 | Activin A receptor, type 1 | 7.431 | 1.65E-03 | 2.056 |
| 104523_at | Lrrc8c | Leucine rich repeat containing 8 family, member C | 7.222 | 1.81E-03 | 1.823 |
| 100323_at | Amd2 | S-adenosylmethionine decarboxylase 2 | 7.068 | 1.96E-03 | 2.735 |
| 104557_at | Pitpnb | Phosphatidylinositol transfer protein, beta | 7.060 | 1.97E-03 | 1.963 |
| 98817_at | Fst | Follistatin | 6.969 | 2.06E-03 | 4.431 |
| 92901_at | Rara | Retinoic acid receptor, alpha | 6.842 | 2.21E-03 | 1.661 |
| 93228_at | Hells | Helicase, lymphoid specific | 6.578 | 2.53E-03 | 1.897 |
| 93550_at | Csrp2 | Cysteine and glycine-rich protein 2 | 6.540 | 2.58E-03 | 3.272 |
| 93184_at | Tbl2 | Transducin (beta)-like 2 | 5.996 | 3.68E-03 | 1.350 |
| 99633_at | Ncdn | Neurochondrin | 5.833 | 4.05E-03 | 1.958 |
| 95486_at | Jmjd6 | Jumonji domain containing 6 | 5.659 | 4.51E-03 | 1.785 |
| 103980_at | Epha2 | Eph receptor A2 | 5.656 | 4.52E-03 | 1.968 |
| 95637_at | Flnb | Filamin, beta | 5.514 | 4.97E-03 | 2.654 |
| 95050_at | Chordc1 | Cysteine and histidine-rich domain (CHORD)-containing, zinc-binding protein 1 | 5.418 | 5.29E-03 | 1.368* |
| 103824_at | Wfs1 | Wolfram syndrome 1 homolog (human) | 5.380 | 5.43E-03 | 1.775 |
| 98589_at | Adfp | Adipose differentiation related protein | 4.846 | 7.77E-03 | 1.791* |
| 103876_at | Crlz1 | Charged amino acid rich leucine zipper 1 | 4.786 | 8.15E-03 | 1.376 |
| 100555_at | Dscr1 | Down syndrome critical region homolog 1 (human) | 4.711 | 8.58E-03 | 1.491 |
| 97500_g_at | Fhl1 | Four and a half LIM domains 1 | 4.682 | 8.75E-03 | 1.489 |
| 92317_at | Elavl2 | ELAV (embryonic lethal, abnormal vision, Drosophila)-like 2 (Hu antigen B) | 4.576 | 9.54E-03 | 1.236 |
| 92867_at | Phc2 | Polyhomeotic-like 2 (Drosophila) | 4.571 | 9.57E-03 | 1.986* |
| 95738_at | Aldh18a1 | Aldehyde dehydrogenase 18 family, member A1 | 4.538 | 9.86E-03 | 1.738 |
| 92399_at | Runx1 | Runt related transcription factor 1 | 4.532 | 9.91E-03 | 2.389 |
| 93013_at | Id2 | Inhibitor of DNA binding 2 | 4.469 | 1.04E-02 | 2.583* |
| 100962_at | Nab2 | Ngfi-A binding protein 2 | 4.466 | 1.04E-02 | 1.619 |
| 100277_at | Inhba | Inhibin beta-A | 4.459 | 1.04E-02 | 11.472 |
| 103791_at | Narg1 | NMDA receptor-regulated gene 1 | 4.458 | 1.05E-02 | 1.723 |
| 93546_s_at | Cbfb | Core binding factor beta | 4.432 | 1.07E-02 | 1.508* |
| 97520_s_at | Nnat | Neuronatin | 4.408 | 1.09E-02 | 2.629 |
| 94387_at | Spata5 | Spermatogenesis associated 5 | 4.376 | 1.12E-02 | 2.143 |
| 160330_at | Chordc1 | Cysteine and histidine-rich domain (CHORD)-containing, zinc-binding protein 1 | 4.271 | 1.22E-02 | 1.344* |
| 98090_at | Krr1 | KRR1, small subunit (SSU) processome component, homolog (yeast) | 4.246 | 1.24E-02 | 1.379 |
| 160119_at | Creld2 | Cysteine-rich with EGF-like domains 2 | 4.169 | 1.31E-02 | 1.967 |
| 103839_at | Sphk1 | Sphingosine kinase 1 | 4.062 | 1.43E-02 | 2.680 |
| 102798_at | Adm | Adrenomedullin | -7.536 | 1.57E-03 | 0.071* |
| 99988_at | Dym | Dymeclin | -7.388 | 1.68E-03 | 0.690 |
| 160215_at | Aes | Amino-terminal enhancer of split | -7.086 | 1.95E-03 | 0.601* |
| 94878_at | Btbd1 | BTB (POZ) domain containing 1 | -7.007 | 2.01E-03 | 0.563* |
| 93534_at | Dcn | Decorin | -6.706 | 2.38E-03 | 0.292 |
| 104312_at | Abhd14a | Abhydrolase domain containing 14A | -6.483 | 2.67E-03 | 0.594 |
| 100992_at | Phc1 | Polyhomeotic-like 1 (Drosophila) | -6.378 | 2.87E-03 | 0.649 |
| 98555_at | Ttc3 | Tetratricopeptide repeat domain 3 | -6.215 | 3.20E-03 | 0.535 |
| 161183_at | Dag1 | Dystroglycan 1 | -5.885 | 3.95E-03 | 0.336 |
| 103007_at | Efna1 | Ephrin A1 | -5.670 | 4.48E-03 | 0.741 |
| 102398_at | Rxrb | Retinoid X receptor beta | -5.285 | 5.74E-03 | 0.829 |
| 160311_at | Plxnb2 | Plexin B2 | -5.093 | 6.56E-03 | 0.557 |
| 102266_at | Inha | Inhibin alpha | -5.087 | 6.58E-03 | 0.833 |
| 101858_at | Rfng | Radical fringe gene homolog (drosophila) | -4.919 | 7.39e-03 | 0.689 |
| 99929_at | Ext2 | Exostoses (multiple) 2 | -4.861 | 7.67E-03 | 0.689 |
| 96672_at | Hod | Homeobox only domain | -4.746 | 8.37E-03 | 0.794 |
| 102579_f_at | Hoxa6 | Homeo box a6 | -4.625 | 9.14e-03 | 0.655 |
| 103804_at | Reck | Reversion-inducing-cysteine-rich protein with kazal motifs | -4.571 | 9.57e-03 | 0.219 |
| 103025_at | Mov10 | Moloney leukemia virus 10 | -4.559 | 9.68E-03 | 0.746 |
| *Protein biosynthesis and ribosome biogenesis* | | |  |  |  |
| 98606_s_at | Wars | Tryptophanyl-tRNA synthetase | 20.802 | 2.06E-05 | 2.240 |
| 95054_at | Tars | Threonyl-tRNA synthetase | 12.831 | 1.44E-04 | 2.895* |
| 95070_at | Nars | Asparaginyl-tRNA synthetase | 12.143 | 1.92E-04 | 1.862 |
| 93752_at | Iars | Isoleucine-tRNA synthetase | 12.140 | 1.94E-04 | 1.795 |
| 100636_at | Eif4ebp1 | Eukaryotic translation initiation factor 4E binding protein 1 | 10.016 | 4.71E-04 | 2.165 |
| 94875_at | Mrpl20 | Mitochondrial ribosomal protein L20 | 10.612 | 3.52E-04 | 2.467 |
| 96755_at | Etf1 | Eukaryotic translation termination factor 1 | 8.592 | 9.56E-04 | 2.343* |
| 98608_at | Etf1 | Eukaryotic translation termination factor 1 | 8.541 | 9.77E-04 | 2.149* |
| 96754_s_at | Etf1 | Eukaryotic translation termination factor 1 | 7.953 | 1.27E-03 | 1.929* |
| 96663_at | Surf6 | Surfeit gene 6 | 7.925 | 1.28E-03 | 2.903 |
| 93488_at | Tsr1 | TSR1, 20S rRNA accumulation, homolog (yeast) | 7.808 | 1.34E-03 | 3.983 |
| 103708_at | Eif1a | Eukaryotic translation initiation factor 1A | 7.753 | 1.39E-03 | 1.775 |
| 97824_at | Nola2 | Nucleolar protein family A, member 2 | 7.581 | 1.52E-03 | 3.962 |
| 95109_at | Nol5a | Nucleolar protein 5A | 7.536 | 1.57E-03 | 3.354* |
| 103926_at | Eif4g1 | Eukaryotic translation initiation factor 4, gamma 1 | 6.650 | 2.44E-03 | 1.953 |
| 94363_at | Bms1l | BMS1-like, ribosome assembly protein (yeast) | 6.604 | 2.49E-03 | 1.917 |
| 94253_at | Eif2s1 | Eukaryotic translation initiation factor 2, subunit 1 alpha | 6.597 | 2.51E-03 | 2.488* |
| 160341_at | Jtv1 | JTV1 gene | 6.352 | 2.93E-03 | 2.312 |
| 96693_at | Rars | Arginyl-tRNA synthetase | 6.350 | 2.94E-03 | 2.744 |
| 93058_at | Eif1a | Eukaryotic translation initiation factor 1A | 6.068 | 3.51E-03 | 2.007 |
| 93564_at | Yars | Tyrosyl-tRNA synthetase | 6.009 | 3.65E-03 | 1.507 |
| 93270_at | Gars | Glycyl-tRNA synthetase | 5.937 | 3.83E-03 | 2.012 |
| 97414_at | Noc4l | Nucleolar complex associated 4 homolog (S. Cerevisiae) | 5.814 | 4.10E-03 | 2.244 |
| 95159_at | Mrps18b | Mitochondrial ribosomal protein S18B | 5.768 | 4.20E-03 | 2.179 |
| 94462_at | Eif2b1 | Eukaryotic translation initiation factor 2B, subunit 1 (alpha) | 5.733 | 4.27E-03 | 1.764 |
| 104702_at | Wdr46 | WD repeat domain 46 | 5.669 | 4.48E-03 | 2.010 |
| 160283_at | Rsl1d1 | Ribosomal L1 domain containing 1 | 5.431 | 5.25E-03 | 2.104 |
| 93787_f_at | Mrpl18 | Mitochondrial ribosomal protein L18 | 5.296 | 5.70E-03 | 1.606* |
| 160266_r_at | Bxdc2 | Brix domain containing 2 | 5.220 | 6.01E-03 | 1.743 |
| 98138_at | Rpl7l1 | Ribosomal protein L7-like 1 | 5.162 | 6.25E-03 | 1.638 |
| 160226_at | Gfm1 | G elongation factor 1 | 5.144 | 6.34E-03 | 1.588 |
| 93973_at | Eif3s9 | Eukaryotic translation initiation factor 3, subunit 9 (eta) | 4.920 | 7.38E-03 | 1.661 |
| 94327_at | Mrps18a | Mitochondrial ribosomal protein S18A | 4.876 | 7.59E-03 | 1.462 |
| 96604_at | Dus1l | Dihydrouridine synthase 1-like (S. cerevisiae) | 4.833 | 7.84E-03 | 1.447 |
| 95046_s_at | Eif2b4 | Eukaryotic translation initiation factor 2B, subunit 4 delta | 4.780 | 8.19E-03 | 1.302 |
| 101530_at | Eftud2 | Elongation factor Tu GTP binding domain containing 2 | 4.719 | 8.54E-03 | 1.639 |
| 99101_at | Eif3s7 | Eukaryotic translation initiation factor 3, subunit 7 (zeta) | 4.637 | 9.06E-03 | 1.700 |
| 160431_at | Mrpl12 | Mitochondrial ribosomal protein L12 | 4.612 | 9.26E-03 | 1.724 |
| 94494_at | Farslb | Phenylalanine-tRNA synthetase-like, beta subunit | 4.586 | 9.45E-03 | 2.297 |
| 160231_at | Farsla | Phenylalanine-tRNA synthetase-like, alpha subunit | 4.071 | 1.42E-02 | 1.820 |
| 96883_at | Eif3s4 | Eukaryotic translation initiation factor 3, subunit 4 (delta) | 4.064 | 1.42E-02 | 1.474 |
| 99185_at | Mrpl41 | Mitochondrial ribosomal protein L41 | 4.327 | 1.16E-02 | 1.325 |
| 93929_s_at | Mrpplf3 | Mitogen regulated protein, proliferin 3 | 4.324 | 1.17E-02 | 4.616 |
| 93786_i_at | Mrpl18 | Mitochondrial ribosomal protein L18 | 4.014 | 1.49E-02 | 1.929* |
| 97559_at | Eef2 | Eukaryotic translation elongation factor 2 | -7.006 | 2.02E-03 | 0.712 |
| 94767_at | Rps11 | Ribosomal protein S11 | -6.790 | 2.27E-03 | 0.441 |
| 97318_at | Dtd1 | D-tyrosyl-tRNA deacylase 1 homolog (S. cerevisiae) | -5.995 | 3.69E-03 | 0.693 |
| 99599_s_at | Ptov1 | Prostate tumor over expressed gene 1 | -5.948 | 3.80E-03 | 0.504 |
| 96059_at | Mrpl48 | Mitochondrial ribosomal protein L48 | -5.780 | 4.17E-03 | 0.549 |
| 100727_at | Rpl28 | Ribosomal protein L28 | -5.437 | 5.22E-03 | 0.751 |
| 101573_f_at | Rpl27a | Ribosomal protein l27a | -5.049 | 6.76E-03 | 0.741 |
| 98564_f_at | Rps26 | Ribosomal protein S26 | -4.923 | 7.36E-03 | 0.700 |
| *Apoptosis* | | |  |  |  |
| 94225_at | Atg5l | Autophagy-related 5-like (yeast) | 7.614 | 1.48E-03 | 1.552* |
| 100442_at | Tbrg4 | Transforming growth factor beta regulated gene 4 | 7.598 | 1.51E-03 | 1.898* |
| 93215_at | Tnfaip1 | Tumor necrosis factor, alpha-induced protein 1 (endothelial) | 6.033 | 3.60E-03 | 1.434 |
| 101035_at | Api5 | Apoptosis inhibitor 5 | 5.046 | 6.78E-03 | 1.723 |
| 160794_at | Smyd2 | SET and MYND domain containing 2 | 4.906 | 7.47E-03 | 1.645 |
| 104710_at | Bak1 | BCL2-antagonist/killer 1 | 4.866 | 7.65E-03 | 2.249 |
| 96008_at | Dad1 | Defender against cell death 1 | 4.614 | 9.25E-03 | 1.398 |
| 99608_at | Prdx2 | Peroxiredoxin 2 | 4.576 | 9.54E-03 | 1.500 |
| 104157_at | Fastkd5 | FAST kinase domains 5 | 4.542 | 9.82E-03 | 1.673 |
| 97284_at | Bcl2l13 | BCL2-like 13 (apoptosis facilitator) | 4.539 | 9.85E-03 | 1.370* |
| 96252_at | Pdcd6ip | Programmed cell death 6 interacting protein | 4.370 | 1.13E-02 | 1.528 |
| 98056_at | Phlda3 | Pleckstrin homology-like domain, family A, member 3 | 4.342 | 1.15E-02 | 2.747 |
| 102921_s_at | Fas | Fas (TNF receptor superfamily member) | -15.289 | 8.47E-05 | 0.304 |
| 102860_at | Serpina3g | Serine (or cysteine) peptidase inhibitor, clade A, member 3G | -7.614 | 1.48E-03 | 0.307 |
| 103029_at | Pdcd4 | Programmed cell death 4 | -7.496 | 1.60E-03 | 0.215 |
| 101554_at | Nfkbia | Nuclear factor of kappa light chain gene enhancer in B-cells inhibitor, alpha | -6.748 | 2.31E-03 | 0.538 |
| 101965_at | Rnf13 | Ring finger protein 13 | -6.712 | 2.37E-03 | 0.531 |
| 96765_at | Peg3 | Paternally expressed 3 | -6.188 | 3.25E-03 | 0.088* |
| 98424_at | Ptpn13 | Protein tyrosine phosphatase, non-receptor type 13 | -5.826 | 4.07E-03 | 0.396 |
| 100988_at | Bcl2l11 | BCL2-like 11 (apoptosis facilitator) | -5.786 | 4.15E-03 | 0.643 |
| 101451_at | Peg3 | Paternally expressed 3 | -5.576 | 4.79E-03 | 0.155 |
| 160645_at | Birc3 | Baculoviral IAP repeat-containing 3 | -5.496 | 5.03E-03 | 0.629 |
| 95910_f_at | Rbed1 | RNA binding motif and ELMO domain 1 | -5.053 | 6.74E-03 | 0.745 |
| *Ubiquitin cycle* | | |  |  |  |
| 97479_at | Ube2l3 | Ubiquitin-conjugating enzyme E2L 3 | 12.664 | 1.56E-04 | 1.281 |
| 104715_at | Ubap2 | Ubiquitin-associated protein 2 | 10.147 | 4.42E-04 | 1.288 |
| 95600_at | Arih2 | Ariadne homolog 2 (Drosophila) | 9.588 | 5.95E-04 | 1.611 |
| 97906_at | Siah2 | Seven in absentia 2 | 8.513 | 9.98E-04 | 1.445 |
| 103350_at | Psmd7 | Proteasome (prosome, macropain) 26S subunit, non-ATPase, 7 | 6.986 | 2.04E-03 | 1.650 |
| 99575_at | Ubqln1 | Ubiquilin 1 | 6.857 | 2.19E-03 | 1.798 |
| 92547_at | Hip2 | Huntingtin interacting protein 2 | 6.310 | 2.99E-03 | 1.559 |
| 102776_at | Usp38 | Ubiquitin specific peptidase 38 | 6.065 | 3.52E-03 | 1.555 |
| 160534_at | Psmc5 | Protease (prosome, macropain) 26S subunit, ATPase 5 | 5.816 | 4.09E-03 | 1.468 |
| 96563_at | Usp24 | Ubiquitin specific peptidase 24 | 5.545 | 4.88E-03 | 1.657 |
| 95448_at | Psmc2 | Proteasome (prosome, macropain) 26S subunit, ATPase 2 | 5.183 | 6.16E-03 | 1.888 |
| 92769_at | Psmd3 | Proteasome (prosome, macropain) 26S subunit, non-ATPase, 3 | 5.038 | 6.82E-03 | 1.620 |
| 96061_at | Usp14 | Ubiquitin specific peptidase 14 | 4.977 | 7.11E-03 | 1.784* |
| 160605_s_at | Usp38 | Ubiquitin specific peptidase 38 | 4.960 | 7.17E-03 | 2.235 |
| 160856_at | Ubl4 | Ubiquitin-like 4 | 4.924 | 7.36E-03 | 1.287 |
| 93069_at | Ube2d2 | Ubiquitin-conjugating enzyme E2D 2 | 4.719 | 8.55E-03 | 1.494 |
| 160209_at | Rnf44 | Ring finger protein 44 | 4.663 | 8.87E-03 | 1.816 |
| 99102_at | Usp9x | Ubiquitin specific peptidase 9, X chromosome | 4.633 | 9.09E-03 | 1.464* |
| 103906_f_at | Nedd4l | Neural precursor cell expressed, developmentally down-regulated gene 4-like | 4.661 | 8.89E-03 | 1.973 |
| 94219_at | Psmb2 | Proteasome (prosome, macropain) subunit, beta type 2 | 4.645 | 9.00E-03 | 1.635 |
| 103907_at | Nedd4l | Neural precursor cell expressed, developmentally down-regulated gene 4-like | 4.386 | 1.11E-02 | 1.561 |
| 97274_at | Psmd14 | Proteasome (prosome, macropain) 26S subunit, non-ATPase, 14 | 4.361 | 1.13E-02 | 1.946 |
| 96959_at | Ube2n | Ubiquitin-conjugating enzyme E2N | 4.195 | 1.29E-02 | 1.778* |
| 93988_at | Psma7 | Proteasome (prosome, macropain) subunit, alpha type 7 | 4.125 | 1.36E-02 | 1.337 |
| 104348_at | Bre | Brain and reproductive organ-expressed protein | -7.608 | 1.49E-03 | 0.596 |
| 93101_s_at | Nedd4 | Neural precursor cell expressed, developmentally down-regulted gene 4 | -7.365 | 1.70E-03 | 0.385 |
| 97171_f_at | Ube2l6 | Ubiquitin-conjugating enzyme E2L 6 | -7.123 | 1.91E-03 | 0.778 |
| *Electron transport and energy production* | | |  |  |  |
| 98130_at | Txn2 | Thioredoxin 2 | 9.801 | 5.35E-04 | 1.804* |
| 160339_at | Pycr2 | Pyrroline-5-carboxylate reductase family, member 2 | 5.539 | 4.90E-03 | 2.081 |
| 99019_at | Por | P450 (cytochrome) oxidoreductase | 5.288 | 5.73E-03 | 1.905 |
| 93187_at | Trim47 | Tripartite motif protein 47 | 4.512 | 1.01E-02 | 1.846 |
| 98533_at | Cyb5 | Cytochrome b-5 | -6.834 | 2.22E-03 | 0.289* |
| 92898_at | Cyp7b1 | Cytochrome P450, family 7, subfamily b, polypeptide 1 | -6.077 | 3.49E-03 | 0.536 |
| 99979_at | Cyp1b1 | Cytochrome P450, family 1, subfamily b, polypeptide 1 | -6.013 | 3.64E-03 | 0.123* |
| 92587_at | Fdx1 | Ferredoxin 1 | -5.976 | 3.74E-03 | 0.514 |
| 160611_at | Cyp4v3 | Cytochrome P450, family 4, subfamily v, polypeptide 3 | -5.505 | 5.00E-03 | 0.351 |
| 104153_at | Ivd | Isovaleryl coenzyme A dehydrogenase | -4.990 | 7.04E-03 | 0.508 |
| 160194_at | Gcdh | Glutaryl-Coenzyme A dehydrogenase | -4.948 | 7.23E-03 | 0.745 |
| 160383_at | Cox7a2l | Cytochrome c oxidase subunit viia polypeptide 2-like | -4.931 | 7.31E-03 | 0.563 |
| 96915_f_at | Ndufa3 | NADH dehydrogenase (ubiquinone) 1 alpha subcomplex, 3 | -4.926 | 7.35E-03 | 0.534 |
| *Cytoskeleton organization and biogenesis* | | |  |  |  |
| 100923_at | Myo10 | Myosin X | 11.750 | 2.36E-04 | 3.360 |
| 98559_at | Smtn | Smoothelin | 9.433 | 6.47E-04 | 1.772 |
| 93427_at | Myo1d | Myosin ID | 7.319 | 1.74E-03 | 2.123 |
| 98107_at | Coro1c | Coronin, actin binding protein 1C | 7.249 | 1.79E-03 | 2.949 |
| M12481_5_at | Actb | Actin, beta, cytoplasmic | 7.190 | 1.84E-03 | 1.537* |
| 94835_f_at | Tubb2 | Tubulin, beta 2 | 6.965 | 2.06E-03 | 2.069 |
| 96033_at | Sdc1 | Syndecan 1 | 6.559 | 2.56E-03 | 2.334 |
| 160094_at | Arpc4 | Actin related protein 2/3 complex, subunit 4 | 6.285 | 3.04E-03 | 1.893 |
| 95543_at | Tpm4 | Tropomyosin 4 | 5.852 | 4.00E-03 | 1.889 |
| M12481_3_at | Actb | Actin, beta, cytoplasmic | 5.454 | 5.16E-03 | 1.240* |
| 93937_at | Gas8 | Growth arrest specific 8 | 5.200 | 6.09E-03 | 1.326 |
| 101543_f_at | Tuba6 | Tubulin, alpha 6 | 5.057 | 6.72E-03 | 1.288 |
| 93805_at | Ssna1 | Sjogren's syndrome nuclear autoantigen 1 | 5.054 | 6.74E-03 | 1.409 |
| 101855_at | Mtap6 | Microtubule-associated protein 6 | 4.531 | 9.92E-03 | 1.432 |
| 99119_at | Cfl1 | Cofilin 1, non-muscle | 4.506 | 1.01E-02 | 1.673 |
| 103281_at | Cd2ap | CD2-associated protein | 4.461 | 1.04E-02 | 1.466 |
| 98402_at | Macf1 | Microtubule-actin crosslinking factor 1 | -6.898 | 2.13E-03 | 0.552 |
| 97803_at | Mpp1 | Membrane protein, palmitoylated | -6.489 | 2.66E-03 | 0.650 |
| 102331_at | St5 | Suppression of tumorigenicity 5 | -5.915 | 3.88E-03 | 0.608 |
| 98454_at | Palm | Paralemmin | -5.551 | 4.86E-03 | 0.685 |
| 162245_f_at | Myo6 | Myosin VI | -4.866 | 7.65E-03 | 0.709 |
| 160379_at | Epb4.1 | Erythrocyte protein band 4.1 | -4.850 | 7.74E-03 | 0.592 |
| 93898_at | Sgcb | Sarcoglycan, beta (dystrophin-associated glycoprotein) | -4.568 | 9.60E-03 | 0.563 |
| 94699_at | Ipp | IAP promoted placental gene | -4.552 | 9.73E-03 | 0.802 |
| *Cell growth and proliferation* | | |  |  |  |
| 99915_at | Areg | Amphiregulin | 14.423 | 1.03E-04 | 4.913* |
| 97426_at | Emp1 | Epithelial membrane protein 1 | 7.362 | 1.71E-03 | 2.237* |
| 160326_at | Cdv3 | Carnitine deficiency-associated gene expressed in ventricle 3 | 7.062 | 1.97E-03 | 2.016 |
| 96632_at | Morf4l2 | Mortality factor 4 like 2 | 6.399 | 2.82E-03 | 1.349 |
| 101918_at | Tgfb1 | Transforming growth factor, beta 1 | 6.028 | 3.61E-03 | 4.196* |
| 160788_at | Pes1 | Pescadillo homolog 1, containing BRCT domain (zebrafish) | 5.973 | 3.75E-03 | 1.929 |
| 95148_at | Ak2 | Adenylate kinase 2 | 5.934 | 3.84E-03 | 2.320* |
| 96907_at | Cherp | Calcium homeostasis endoplasmic reticulum protein | 5.020 | 6.88E-03 | 1.381 |
| 99537_at | Ruvbl1 | Ruvb-like protein 1 | 4.366 | 1.13E-02 | 1.705 |
| 93326_at | Tspan7 | Tetraspanin 7 | 4.093 | 1.39E-02 | 1.635 |
| 99457_at | Mki67 | Antigen identified by monoclonal antibody Ki 67 | -13.899 | 1.14E-04 | 0.371 |
| 103536_at | Tmeff2 | Transmembrane protein with EGF-like and two follistatin-like domains 2 | -10.335 | 4.00E-04 | 0.521 |
| 104390_at | Anp32a | Acidic (leucine-rich) nuclear phosphoprotein 32 family, member A | -7.479 | 1.62E-03 | 0.509 |
| 100457_at | Glg1 | Golgi apparatus protein 1 | -5.853 | 4.00E-03 | 0.597 |
| 97892_at | Brd8 | Bromodomain containing 8 | -5.730 | 4.28E-03 | 0.700 |
| 97951_s_at | Tsc2 | Tuberous sclerosis 2 | -5.677 | 4.45E-03 | 0.751 |
| 96920_at | Htra1 | Htra serine peptidase 1 | -5.546 | 4.88E-03 | 0.493 |
| 93994_at | Chpt1 | Choline phosphotransferase 1 | -4.938 | 7.28E-03 | 0.372 |
| 93284_at | Cirbp | Cold inducible RNA binding protein | -4.852 | 7.72E-03 | 0.662 |
| 102224_at | Igf1r | Insulin-like growth factor I receptor | -4.708 | 8.59E-03 | 0.401 |
| *Angiogenesis* | | |  |  |  |
| 104110_at | Gatad2a | GATA zinc finger domain containing 2A | 9.943 | 4.83E-04 | 2.224 |
| 93216_at | Fgf2 | Fibroblast growth factor 2 | 8.741 | 8.99E-04 | 1.250 |
| 103520_at | Vegfa | Vascular endothelial growth factor A | 4.699 | 8.65E-03 | 3.731* |
| 92560_g_at | Vcam1 | Vascular cell adhesion molecule 1 | -16.206 | 5.49E-05 | 0.407 |
| 92365_at | Figf | C-fos induced growth factor | -5.711 | 4.35E-03 | 0.076* |
| 92558_at | Vcam1 | Vascular cell adhesion molecule 1 | -5.347 | 5.53E-03 | 0.312 |
| 95531_at | Amot | Angiomotin | -4.766 | 8.26E-03 | 0.339 |
| 92559_at | Vcam1 | Vascular cell adhesion molecule 1 | -4.653 | 8.93E-03 | 0.419 |
| *Proteolysis and peptidolysis* | | |  |  |  |
| 100581_at | Cstb | Cystatin B | 6.716 | 2.36E-03 | 1.481 |
| 104025_at | Thop1 | Thimet oligopeptidase 1 | 4.763 | 8.28E-03 | 1.867* |
| 100983_at | Prep | Prolyl endopeptidase | 4.095 | 1.39E-02 | 1.773 |
| 96259_at | Npepps | Aminopeptidase puromycin sensitive | -4.772 | 8.22E-03 | 0.663 |
| *Microtubule dynamics* | | |  |  |  |
| 96298_f_at | Dynll1 | Dynein light chain 1 LC8-type1 | 5.529 | 4.93E-03 | 1.532 |
| *Protein folding* | | |  |  |  |
| 104738_at | Dnajc2 | DnaJ (Hsp40) homolog, subfamily C, member 2 | 8.547 | 9.75E-04 | 2.784 |
| 101890_f_at | Dnajc2 | DnaJ (Hsp40) homolog, subfamily C, member 2 | 7.603 | 1.50E-03 | 3.280 |
| 104057_at | Grpel1 | GrpE-like 1, mitochondrial | 6.937 | 2.08E-03 | 2.130 |
| 96254_at | Dnajb1 | DnaJ (Hsp40) homolog, subfamily B, member 1 | 6.106 | 3.43E-03 | 1.569 |
| 92571_at | Hspa4 | Heat shock protein 4 | 6.034 | 3.60e-03 | 2.740* |
| 100352_at | Hspa4 | Heat shock protein 4 | 5.635 | 4.57e-03 | 1.299* |
| 92806_at | Pdrg1 | P53 and DNA damage regulated 1 | 5.226 | 5.97E-03 | 2.670 |
| 100353_g_at | Hspa4 | Heat shock protein 4 | 5.142 | 6.35e-03 | 1.926* |
| 160102_at | Cct8 | Chaperonin subunit 8 (theta) | 5.024 | 6.87E-03 | 2.194 |
| 102761_at | Grpel2 | GrpE-like 2, mitochondrial | 4.408 | 1.09E-02 | 1.439 |
| 96594_at | Hspa4 | Heat shock protein 4 | 4.390 | 1.11e-02 | 1.730* |
| 99082_at | Fkbp10 | FK506 binding protein 10 | 4.312 | 1.18E-02 | 1.776 |
| 92829_at | Hspe1 | Heat shock protein 1 (chaperonin 10) | 4.274 | 1.21e-02 | 1.592 |
| 101562_at | Hspa14 | Heat shock 70kda protein 14 | 4.255 | 1.23e-02 | 1.934 |
| 94218_at | Tcp1 | T-complex protein 1 | 4.238 | 1.24E-02 | 1.316 |
| 97914_at | Hspa9a | Heat shock protein 9a | 4.071 | 1.42e-02 | 1.295 |
| 101955_at | Hspa5 | Heat shock 70kd protein 5 (glucose-regulated protein) | 4.002 | 1.50e-02 | 2.376 |
| 99111_at | Clpb | ClpB caseinolytic peptidase B homolog (E. coli) | -5.973 | 3.75E-03 | 0.644 |
| *Blood coagulation* | | |  |  |  |
| 92978_s_at | Serpinb2 | Serine (or cysteine) peptidase inhibitor, clade B, member 2 | 17.409 | 4.35E-05 | 26.142 |
| 101393_at | Anxa3 | Annexin A3 | 6.937 | 2.08E-03 | 2.492 |
| 94147_at | Serpine1 | Serine (or cysteine) peptidase inhibitor, clade E, member 1 | 6.700 | 2.39E-03 | 36.112* |
| 95313_at | F2r | Coagulation factor II (thrombin) receptor | 4.428 | 1.07E-02 | 2.096 |
| 95709_at | Vkorc1 | Vitamin K epoxide reductase complex, subunit 1 | -5.701 | 4.38E-03 | 0.494 |
| *DNA repair* | | |  |  |  |
| 101540_at | Tdg | Thymine DNA glycosylase | 5.128 | 6.41E-03 | 2.046 |
| 102792_at | Ung | Uracil DNA glycosylase | 4.550 | 9.76E-03 | 1.424 |
| *Nuclear stability, chromatin structure, and gene expression* | | |  |  |  |
| 97366_at | Lemd2 | LEM domain containing 2 | 5.305 | 5.67E-03 | 1.511 |
| 101459_at | Chd1 | Chromodomain helicase DNA binding protein 1 | 5.275 | 5.78E-03 | 2.169 |
| 101414_at | Lmnb2 | Lamin B2 | 4.322 | 1.17E-02 | 1.266 |
| 100616_at | Cenpa | Centromere autoantigen A | -5.200 | 6.09E-03 | 0.757 |
| 97809_at | Ehmt2 | Euchromatic histone lysine N-methyltransferase 2 | -4.544 | 9.81E-03 | 0.693 |
| *Peroxisomal organization and biogenesis* | | |  |  |  |
| 100027_s_at | Pex14 | Peroxisomal biogenesis factor 14 | 5.829 | 4.07E-03 | 1.366 |
| 99469_at | Pex6 | Peroxisomal biogenesis factor 6 | -6.148 | 3.33E-03 | 0.650 |
| 94491_at | Pex13 | Peroxisomal biogenesis factor 13 | -4.656 | 8.91E-03 | 0.712 |
| *Muscle contraction* | | |  |  |  |
| 99942_s_at | Cnn1 | Calponin 1 | 6.254 | 3.12E-03 | 5.065* |
| 100605_at | Tpm2 | Tropomyosin 2, beta | 4.762 | 8.28E-03 | 3.330 |
| *Protein binding* | | |  |  |  |
| 104293_at | Klhl21 | Kelch-like 21 (Drosophila) | 6.949 | 2.08E-03 | 3.363 |
| 93190_at | Heatr2 | HEAT repeat containing 2 | 4.667 | 8.84E-03 | 1.364 |
| *Spermidine biosynthetic process* | | |  |  |  |
| 92540_f_at | Srm | Spermidine synthase | 6.296 | 3.02E-03 | 4.023* |
| 101291_at | Srm | Spermidine synthase | 4.806 | 8.00E-03 | 1.509* |
| *Unknown* | | |  |  |  |
| 160067_at | 2310057D15Rik | RIKEN cDNA 2310057D15 gene | 10.016 | 4.69E-04 | 1.780 |
| 160801_at | Pqlc1 | PQ loop repeat containing 1 | 9.251 | 7.23E-04 | 1.808 |
| 95657_f_at | D13Wsu177e | DNA segment, Chr 13, Wayne State University 177, expressed | 8.370 | 1.06E-03 | 3.231 |
| 95139_at | 2510001I10Rik | RIKEN cDNA 2510001I10 gene | 8.222 | 1.12E-03 | 1.563 |
| 103619_at | 1810044O22Rik | RIKEN cDNA 1810044O22 gene | 8.034 | 1.23E-03 | 1.612 |
| 96878_at | 1810044O22Rik | RIKEN cDNA 1810044O22 gene | 7.870 | 1.32E-03 | 2.210 |
| 160990_r_at | BC016198 | cDNA sequence BC016198 | 7.792 | 1.36E-03 | 2.637 |
| 98509_at | BC002199 | cDNA sequence BC002199 | 7.526 | 1.58E-03 | 1.831 |
| 103312_f_at | 2610101J03Rik | RIKEN cDNA 2610101J03 gene | 4.732 | 8.47E-03 | 2.361 |
| 104229_at | 2610200G18Rik | RIKEN cDNA 2610200G18 gene | 4.710 | 8.58E-03 | 2.426 |
| 97252_at | 2610012O22Rik | RIKEN cDNA 2610012O22 gene | 6.973 | 2.05E-03 | 2.094 |
| 95648_at | NA | NA | 6.896 | 2.14E-03 | 1.374 |
| 160279_at | 4930588M11Rik | RIKEN cDNA 4930588M11 gene | 6.725 | 2.35E-03 | 2.010 |
| 103601_at | 2210013M04Rik | RIKEN cDNA 2210013M04 gene | 6.638 | 2.45E-03 | 1.424 |
| 98890_at | 1700012G19Rik | RIKEN cDNA 1700012G19 gene | 6.543 | 2.57E-03 | 1.603 |
| 94502_at | D13Wsu50e | DNA segment, Chr 13, Wayne State University 50, expressed | 6.529 | 2.59E-03 | 2.023 |
| 93579_at | Jagn1 | Jagunal homolog 1 (Drosophila) | 6.456 | 2.72E-03 | 2.053 |
| 95451_at | 2810405J04Rik | RIKEN cDNA 2810405J04 gene | 6.386 | 2.85E-03 | 2.312 |
| 104475_at | AA408278 | Expressed sequence AA408278 | 6.363 | 2.91E-03 | 1.716 |
| 104284_at | 5031439G07Rik | RIKEN cDNA 5031439G07 gene | 6.296 | 3.02E-03 | 1.668 |
| 101741_at | 2810433D01Rik | RIKEN cDNA 2810433D01 gene | 6.099 | 3.44E-03 | 1.480 |
| 97462_at | 3110006P09Rik | RIKEN cDNA 3110006P09 gene | 6.031 | 3.61E-03 | 1.951 |
| 96871_at | 2310042G06Rik | RIKEN cDNA 2310042G06 gene | 6.001 | 3.68E-03 | 2.339 |
| 94536_s_at | 2900073G15Rik | RIKEN cDNA 2900073G15 gene | 5.965 | 3.77E-03 | 1.336 |
| 95409_at | 1110019J04Rik | RIKEN cDNA 1110019J04 gene | 5.964 | 3.77E-03 | 2.252 |
| 97992_at | BC024683 | cDNA sequence BC024683 | 5.870 | 3.98E-03 | 1.541 |
| 102427_at | Fyttd1 | Forty-two-three domain containing 1 | 5.813 | 4.10E-03 | 1.909 |
| 102387_at | BC003885 | cDNA sequence BC003885 | 5.777 | 4.18E-03 | 1.680 |
| 97404_at | 1500034J01Rik | RIKEN cDNA 1500034J01 gene | 5.758 | 4.22E-03 | 1.834 |
| 104037_at | BC016198 | cDNA sequence BC016198 | 5.661 | 4.50E-03 | 2.004 |
| 94360_at | 2700029M09Rik | RIKEN cDNA 2700029M09 gene | 5.549 | 4.87E-03 | 1.842 |
| 102920_at | 9130422G05Rik | RIKEN cDNA 9130422G05 gene | 5.455 | 5.15E-03 | 1.640 |
| 160223_at | 2610511O17Rik | RIKEN cDNA 2610511O17 gene | 5.452 | 5.17E-03 | 1.797 |
| 94111_r_at | D10627 | cDNA sequence D10627 | 5.443 | 5.20E-03 | 1.464 |
| 160156_at | 0910001A06Rik | RIKEN cDNA 0910001A06 gene | 5.410 | 5.32E-03 | 2.450 |
| 99948_at | Ccdc95 | Coiled-coil domain containing 95 | 5.326 | 5.60E-03 | 1.227 |
| 104341_at | 2310014H01Rik | RIKEN cDNA 2310014H01 gene | 5.268 | 5.82E-03 | 1.648 |
| 100895_at | D16Bwg1547e | DNA segment, Chr 16, Brigham & Women's Genetics 1547 expressed | 5.279 | 5.77E-03 | 1.852 |
| 99432_at | NA | NA | 5.277 | 5.78E-03 | 1.315 |
| 93795_at | NA | NA | 5.189 | 6.14E-03 | 1.737 |
| 96212_at | 2310061I04Rik | RIKEN cDNA 2310061I04 gene | 4.966 | 7.15E-03 | 2.211 |
| 93478_at | 4930461P20Rik | RIKEN cDNA 4930461P20 gene | 4.949 | 7.23E-03 | 1.569 |
| 99949_at | AI225782 | Expressed sequence AI225782 | 4.948 | 7.24E-03 | 1.847 |
| 92388_at | BC038311 | cDNA sequence BC038311 | 4.931 | 7.31E-03 | 1.353 |
| 96709_at | 1110008P14Rik | RIKEN cDNA 1110008P14 gene | 4.912 | 7.42E-03 | 2.714 |
| 104060_at | 2700088M22Rik | RIKEN cDNA 2700088M22 gene | 4.879 | 7.57E-03 | 1.448 |
| 104643_at | BC037006 | cDNA sequence BC037006 | 4.833 | 7.83E-03 | 1.950 |
| 96677_at | 2410195B05Rik | RIKEN cDNA 2410195B05 gene | 4.818 | 7.92E-03 | 1.970 |
| 104141_at | D15Wsu75e | DNA segment, Chr 15, Wayne State University 75, expressed | 4.791 | 8.10E-03 | 1.625 |
| 96904_at | 2310075G12Rik | RIKEN cDNA 2310075G12 gene | 4.757 | 8.32E-03 | 1.363 |
| 103863_at | 5630401J11Rik | RIKEN cDNA 5630401J11 gene | 4.669 | 8.83E-03 | 1.553 |
| 104463_at | 3830408P06Rik | RIKEN cDNA 3830408P06 gene | 4.688 | 8.71E-03 | 1.503 |
| 160768_at | D030056L22Rik | RIKEN cDNA D030056L22 gene | 4.687 | 8.71E-03 | 1.465 |
| 96135_at | 3110003A17Rik | RIKEN cDNA 3110003A17 gene | 4.684 | 8.73E-03 | 2.521 |
| 160620_at | Gt(ROSA)26Sor | Gene trap ROSA 26, Philippe Soriano | 4.613 | 9.26E-03 | 1.566 |
| 98518_f_at | 2300002G24Rik | RIKEN cDNA 2300002G24 gene | 4.521 | 1.00E-02 | 1.282 |
| 98959_at | 0610016J10Rik | RIKEN cDNA 0610016J10 gene | 4.502 | 1.01E-02 | 1.450 |
| 97205_at | 6030465E24Rik | RIKEN cDNA 6030465E24 gene | 4.496 | 1.02E-02 | 2.316 |
| 96156_at | 4930418G15Rik | RIKEN cDNA 4930418G15 gene | 4.483 | 1.03E-02 | 2.786 |
| 99677_at | 2610042O14Rik | RIKEN cDNA 2610042O14 gene | 4.454 | 1.05E-02 | 1.232 |
| 103768_at | D19Ertd678e | DNA segment, Chr 19, ERATO Doi 678, expressed | 4.416 | 1.08E-02 | 1.896 |
| 103017_at | LOC664862 | Similar to transmembrane 7 superfamily member 1 | 4.342 | 1.15E-02 | 1.568 |
| 161541_r_at | AI597080 | Expressed sequence AI597080 | 4.329 | 1.16E-02 | 1.089 |
| 160835_i_at | 1110007C09Rik | RIKEN cDNA 1110007C09 gene | 4.327 | 1.17E-02 | 2.186 |
| 100306_at | 2700007P21Rik | RIKEN cDNA 2700007P21 gene | 4.306 | 1.18E-02 | 1.610 |
| 102072_g_at | 1500034J01Rik | RIKEN cDNA 1500034J01 gene | 4.273 | 1.21E-02 | 1.383 |
| 96113_at | D18Wsu98e | DNA segment, Chr 18, Wayne State University 98, expressed | 4.264 | 1.22E-02 | 1.571 |
| 104400_at | 0610042I15Rik | RIKEN cDNA 0610042I15 gene | 4.213 | 1.27E-02 | 2.242 |
| 160166_r_at | 2810409H07Rik | RIKEN cDNA 2810409H07 gene | 4.211 | 1.27E-02 | 1.539 |
| 94286_at | 9130011J15Rik | RIKEN cDNA 9130011J15 gene | 4.201 | 1.28E-02 | 1.506 |
| 160831_at | AI838661 | Expressed sequence AI838661 | 4.191 | 1.29E-02 | 1.595 |
| 160773_at | D4Wsu132e | DNA segment, Chr 4, Wayne State University 132, expressed | 4.147 | 1.33E-02 | 1.528 |
| 161092_at | NA | NA | 4.124 | 1.36E-02 | 1.425 |
| 98013_at | D15Ertd621e | DNA segment, Chr 15, ERATO Doi 621, expressed | 4.063 | 1.42E-02 | 1.770 |
| 160251_at | 2610312B22Rik | RIKEN cDNA 2610312B22 gene | 4.043 | 1.45E-02 | 1.626 |
| 95052_at | 1110035L05Rik | RIKEN cDNA 1110035L05 gene | -12.756 | 1.49E-04 | 0.336 |
| 98495_at | 5033414D02Rik | RIKEN cDNA 5033414D02 gene | -10.159 | 4.35E-04 | 0.703 |
| 162479_f_at | NA | NA | -8.844 | 8.58E-04 | 0.555 |
| 93437_f_at | 4632419I22Rik | RIKEN cDNA 4632419I22 gene | -8.119 | 1.17E-03 | 0.619 |
| 95693_at | NA | NA | -8.102 | 1.18E-03 | 0.423 |
| 104631_g_at | BC021611 | cDNA sequence BC021611 | -7.832 | 1.33E-03 | 0.701 |
| 102421_at | 2400010G15Rik | RIKEN cDNA 2400010G15 gene | -7.801 | 1.35E-03 | 0.684 |
| 104358_at | D10Ertd610e | DNA segment, Chr 10, ERATO Doi 610, expressed | -7.752 | 1.39E-03 | 0.540 |
| 160883_at | NA | NA | -7.148 | 1.89E-03 | 0.782 |
| 100527_at | D11Ertd99e | DNA segment, Chr 11, ERATO Doi 99, expressed | -7.112 | 1.92E-03 | 0.561 |
| 95442_at | BC010787 | cDNA sequence BC010787 | -6.846 | 2.20E-03 | 0.567 |
| 94022_at | D1Mgi9 | DNA Segment, Chr 1, Mouse Genome Informatics 9 | -6.773 | 2.29E-03 | 0.561 |
| 95523_at | 6530401D17Rik | RIKEN cDNA 6530401D17 gene | -6.638 | 2.45E-03 | 0.492 |
| 95877_at | 2610020H08Rik | RIKEN cDNA 2610020H08 gene | -6.590 | 2.51E-03 | 0.765 |
| 96902_at | 2900091E11Rik | RIKEN cDNA 2900091E11 gene | -6.427 | 2.78E-03 | 0.464 |
| 102233_at | 4833442J19Rik | RIKEN cDNA 4833442J19 gene | -6.340 | 2.95E-03 | 0.624 |
| 96089_at | 4931406C07Rik | RIKEN cDNA 4931406C07 gene | -6.163 | 3.30E-03 | 0.472 |
| 103221_at | 5031425D22Rik | RIKEN cDNA 5031425D22 gene | -5.979 | 3.73E-03 | 0.679 |
| 93059_at | 2610204K14Rik | RIKEN cDNA 2610204K14 gene | -5.927 | 3.86E-03 | 0.440 |
| 162350_at | D330001F17Rik | RIKEN cDNA D330001F17 gene | -5.880 | 3.95E-03 | 0.816 |
| 100963_at | NA | NA | -5.774 | 4.19E-03 | 0.609 |
| 93992_at | 5430437P03Rik | RIKEN cDNA 5430437P03 gene | -5.669 | 4.48E-03 | 0.730 |
| 95090_at | 2410001H17Rik | RIKEN cDNA 2410001H17 gene | -5.515 | 4.97E-03 | 0.675 |
| 95396_at | NA | NA | -5.477 | 5.08E-03 | 0.093 |
| 97738_r_at | BC034204 | cDNA sequence BC034204 | -5.271 | 5.80E-03 | 0.767 |
| 160271_at | 0610007C21Rik | RIKEN cDNA 0610007C21 gene | -5.212 | 6.05E-03 | 0.368 |
| 95045_at | 0610012D09Rik | RIKEN cDNA 0610012D09 gene | -5.184 | 6.16E-03 | 0.532 |
| 161872_f_at | 1110049G11Rik | RIKEN cDNA 1110049G11 gene | -5.086 | 6.58E-03 | 0.672 |
| 104333_at | D17H6S56E-5 | DNA segment, Chr 17, human D6S56E 5 | -5.073 | 6.63E-03 | 0.283 |
| 103481_at | 6720457D02Rik | RIKEN cDNA 6720457D02 gene | -5.059 | 6.72E-03 | 0.680 |
| 104537_at | 0610042C05Rik | RIKEN cDNA 0610042C05 gene | -5.057 | 6.72E-03 | 0.652 |
| 98934_at | 0610007P06Rik | RIKEN cDNA 0610007P06 gene | -5.016 | 6.90E-03 | 0.581 |
| 104183_at | D2Ertd435e | DNA segment, Chr 2, ERATO Doi 435, expressed | -4.981 | 7.07E-03 | 0.708 |
| 97324_at | 5133401N09Rik | RIKEN cDNA 5133401N09 gene | -4.779 | 8.19E-03 | 0.750 |
| 99154_s_at | 1810020D17Rik | RIKEN cDNA 1810020D17 gene | -4.689 | 8.70E-03 | 0.685 |
| 102099_f_at | NA | NA | -4.687 | 8.72E-03 | 0.776 |
| 94784_at | AI593484 | Expressed sequence AI593484 | -4.643 | 9.01E-03 | 0.743 |
| 102960_at | Rag1ap1 | Recombination activating gene 1 gene activation | -4.615 | 9.24E-03 | 0.682 |
| 97797_at | 2210409M21Rik | RIKEN cDNA 2210409M21 gene | -4.534 | 9.90E-03 | 0.756 |

**Table S8. Differential gene expression in serum-starved, G0-arrested N-*ras*^-/-^ fibroblasts after incubation of cell cultures in the presence of serum for 8 hours.** List of 294 differentially expressed probesets (275 different genes) identified by means of SAM contrast (FDR=0,09) comparing the microarray-generated transcriptional profile of WT fibroblasts that had been serum-starved for 24h to that of similarly starved, N-*ras***^-/-^** fibroblast cultures submitted to subsequent incubation in the presence of 20% FBS for 8 hours. To concentrate on loci exclusively regulated by Ras, genes sharing similar values of differential expression between the WT and N-*ras*^-/-^ fibroblast (ratio of the R-fold values in their respective lists within the range 0.6-1.5) were excluded from this list. The differentially expressed loci are identified by Affymetrix probeset ID, gene name and symbol, and listed according to functional category and degree of overexpression or repression, quantitated by d(i) value. The * and # symbols in the R.fold column denote independent validation of the transcriptional data obtained by means of quantitative RT-PCR or Western immunoblot, respectively. d(i) is a parameter measuring the statistical distance separating the calculated expression value of each gene probeset from the null hypothesis (no-change). p-value is an statistical measure indicating the probability of random expression for that probeset. R fold is the log2 value of the fold change measuring the overexpression or repression of the probesets in the collection of microarrays.

| **Probeset ID** | **Gene symbol** | **Gene name** | **d(i)** | **p-value** | **R.fold** |
| --- | --- | --- | --- | --- | --- |
| *Signal transduction* | | |  |  |  |
| 93602_at | Rps6ka4 | Ribosomal protein S6 kinase, polypeptide 4 | 10.701 | 9.84E-05 | 2.743 |
| 94828_at | Oprs1 | Opioid receptor, sigma 1 | 8.733 | 2.45E-04 | 1.882 |
| 94516_f_at | Penk1 | Preproenkephalin 1 | 8.006 | 3.50E-04 | 17.553 |
| 93314_g_at | Map2k3 | Mitogen activated protein kinase kinase 3 | 6.750 | 7.28E-04 | 1.617* |
| 93178_at | Ngef | Neuronal guanine nucleotide exchange factor | 6.410 | 9.29E-04 | 1.663 |
| 101561_at | Mt2 | Metallothionein 2 | 6.041 | 1.21E-03 | 3.462* |
| 97890_at | Sgk | Serum/glucocorticoid regulated kinase | 5.886 | 1.37E-03 | 3.829* |
| 98817_at | Fst | Follistatin | 5.560 | 1.70E-03 | 3.389 |
| 98603_s_at | Rangap1 | RAN gtpase activating protein 1 | 5.283 | 2.11E-03 | 2.515 |
| 92368_at | Ramp3 | Receptor (calcitonin) activity modifying protein 3 | 5.240 | 2.20E-03 | 1.755 |
| 94257_at | Rraga | Ras-related GTP binding A | 5.224 | 2.23E-03 | 1.681 |
| 101030_at | Rhob | Ras homolog gene family, member B | 5.153 | 2.37E-03 | 1.884 |
| 95721_at | Mapkapk2 | MAP kinase-activated protein kinase 2 | 4.909 | 2.92E-03 | 1.612 |
| 103642_at | G3bp1 | Ras-GTPase-activating protein SH3-domain binding protein | 4.871 | 3.02E-03 | 2.197 |
| 93315_at | Map2k3 | Mitogen activated protein kinase kinase 3 | 4.795 | 3.26E-03 | 2.538* |
| 94913_at | G3bp2 | GTPase activating protein (SH3 domain) binding protein 2 | 4.753 | 3.38E-03 | 1.596 |
| 96700_r_at | Rap1b | RAS related protein 1b | 4.662 | 3.66E-03 | 1.389 |
| 94362_at | **Nras** | **Neuroblastoma ras oncogene** | -13.502 | 2.97E-05 | 0.138 |
| 160925_at | **Nras** | **Neuroblastoma ras oncogene** | -9.014 | 2.13E-04 | 0.375 |
| 102759_at | Pik3r2 | Phosphatidylinositol 3-kinase, regulatory subunit, polypeptide 2 (p85 beta) | -8.277 | 3.02E-04 | 0.714 |
| 94022_at | Gltscr2 | Glioma tumor suppressor candidate region gene 2 | -7.697 | 4.07E-04 | 0.663 |
| 103592_at | Map2k5 | Mitogen activated protein kinase kinase 5 | -7.599 | 4.30E-04 | 0.602 |
| 97058_f_at | Rab33b | RAB33B, member of RAS oncogene family | -6.519 | 8.63E-04 | 0.801 |
| 160480_at | Ptprs | Protein tyrosine phosphatase, receptor type, S | -6.023 | 1.22E-03 | 0.656 |
| 96911_at | Gnb2 | Guanine nucleotide binding protein, beta 2 | -5.791 | 1.47E-03 | 0.750 |
| 96132_at | Apcdd1 | Adenomatosis polyposis coli down-regulated 1 | -5.734 | 1.52E-03 | 0.369 |
| 98950_at | Rragc | Ras-related GTP binding C | -5.711 | 1.55E-03 | 0.599 |
| 101947_at | Akap8l | A kinase (PRKA) anchor protein 8-like | -5.310 | 2.07E-03 | 0.389 |
| 103656_at | Lancl1 | LanC (bacterial lantibiotic synthetase component C)-like 1 | -5.307 | 2.08E-03 | 0.471 |
| 93591_at | Pef1 | Penta-EF hand domain containing 1 | -4.994 | 2.67E-03 | 0.661 |
| 93826_at | Ppp2r5a | Protein phosphatase 2, regulatory subunit B (B56), alpha isoform | -4.983 | 2.73E-03 | 0.314* |
| 92698_at | Mertk | C-mer proto-oncogene tyrosine kinase | -4.971 | 2.76E-03 | 0.767 |
| 162011_f_at | Rhou | Ras homolog gene family, member U | -4.910 | 2.92E-03 | 0.590 |
| 100530_at | Ralgds | Ral guanine nucleotide dissociation stimulator | -4.872 | 3.02E-03 | 0.341* |
| 92410_at | Rad23a | RAD23a homolog (S. Cerevisiae) | -4.860 | 3.05E-03 | 0.725 |
| 97710_f_at | Mpv17l | Mpv17 transgene, kidney disease mutant-like | -4.765 | 3.34E-03 | 0.591 |
| 100963_at | Pnkd | Paroxysmal nonkinesiogenic dyskinesia | -4.705 | 3.54E-03 | 0.540 |
| 160892_at | Dlgh3 | Discs, large homolog 3 (Drosophila) | -4.667 | 3.65E-03 | 0.821 |
| *Transcription* | | |  |  |  |
| 102363_r_at | Junb | Jun-B oncogene | 13.693 | 2.75E-05 | 1.834 |
| 102362_i_at | Junb | Jun-B oncogene | 11.464 | 7.09E-05 | 2.123 |
| 98021_at | Praf1 | Polymerase (RNA) I associated factor 1 | 9.058 | 2.04E-04 | 1.880 |
| 98447_at | Cebpa | CCAAT/enhancer binding protein (C/EBP), alpha | 6.710 | 7.55E-04 | 1.445 |
| 100935_at | Tcfl4 | Transcription factor-like 4 | 5.754 | 1.50E-03 | 1.369* |
| 96083_s_at | Hnrpdl | Heterogeneous nuclear ribonucleoprotein D-like | 5.013 | 2.63E-03 | 1.545 |
| 99109_at | Ier2 | Immediate early response 2 | 4.674 | 3.62E-03 | 2.201* |
| 161287_f_at | Mybbp1a | MYB binding protein (P160) 1a | 4.607 | 3.86E-03 | 1.252 |
| 161504_i_at | Zcd1 | Zinc finger, CDGSH-type domain 1 | 4.605 | 3.88E-03 | 1.428 |
| 93708_at | Pias3 | Protein inhibitor of activated STAT 3 | -8.087 | 3.29E-04 | 0.561 |
| 96785_at | Ankrd47 | Ankyrin repeat domain 47 | -8.069 | 3.36E-04 | 0.464 |
| 161308_f_at | Yap1 | Yes-associated protein 1 | -7.650 | 4.23E-04 | 0.768 |
| 102048_at | Ankrd1 | Ankyrin repeat domain 1 (cardiac muscle) | -6.905 | 6.66E-04 | 0.079* |
| 160483_at | Tcf4 | Transcription factor 4 | -5.872 | 1.39E-03 | 0.324 |
| 98790_s_at | Meis1 | Myeloid ecotropic viral integration site 1 | -5.827 | 1.43E-03 | 0.529 |
| 99552_at | Snai2 | Snail homolog 2 (Drosophila) | -5.537 | 1.73E-03 | 0.517 |
| 103466_at | Cyhr1 | Cysteine and histidine rich 1 | -5.480 | 1.81E-03 | 0.684 |
| 160402_at | Tceb2 | Transcription elongation factor B (SIII), polypeptide 2 | -5.460 | 1.83E-03 | 0.588 |
| 103467_g_at | Cyhr1 | Cysteine and histidine rich 1 | -5.420 | 1.88E-03 | 0.519 |
| 97892_at | Brd8 | Bromodomain containing 8 | -5.366 | 1.97E-03 | 0.650 |
| 160619_at | Bsdc1 | BSD domain containing 1 | -5.179 | 2.32E-03 | 0.737 |
| 160245_at | Zfp740 | Zinc finger protein 740 | -4.874 | 3.01E-03 | 0.601 |
| 104458_at | Tcf3 | Transcription factor 3 | -4.788 | 3.28E-03 | 0.711 |
| 160138_at | Mxi1 | Max interacting protein 1 | -4.777 | 3.32E-03 | 0.348 |
| 104209_at | Cyhr1 | Cysteine and histidine rich 1 | -4.740 | 3.43E-03 | 0.812 |
| 96707_at | Zscan21 | Zinc finger and SCAN domain containing 21 | -4.663 | 3.66E-03 | 0.508 |
| 100941_at | Miz1 | Msx-interacting-zinc finger | -4.654 | 3.68E-03 | 0.783 |
| *Primary cell metabolism* | | |  |  |  |
| 94815_at | Bpgm | 2,3-bisphosphoglycerate mutase | 9.395 | 1.69E-04 | 1.958 |
| 98890_at | 1700012G19Rik | RIKEN cDNA 1700012G19 gene | 7.904 | 3.59E-04 | 1.497 |
| 99038_at | Adss | Adenylosuccinate synthetase, non muscle | 6.947 | 6.41E-04 | 1.783 |
| 160492_at | Ddx18 | DEAD (Asp-Glu-Ala-Asp) box polypeptide 18 | 6.418 | 9.24E-04 | 1.653 |
| 95887_at | Soat1 | Sterol O-acyltransferase 1 | 6.246 | 1.03E-03 | 2.099 |
| 92553_at | Esd | Esterase D/formylglutathione hydrolase | 6.027 | 1.22E-03 | 2.325 |
| 160289_s_at | Dlst | Dihydrolipoamide S-succinyltransferase (E2 component of 2-oxo-glutarate complex) | 5.941 | 1.30E-03 | 1.553 |
| 104351_at | Gmppb | GDP-mannose pyrophosphorylase B | 5.885 | 1.38E-03 | 1.730* |
| 160648_at | Fignl1 | Fidgetin-like 1 | 5.545 | 1.71E-03 | 2.010 |
| 92794_f_at | Nme1 | Expressed in non-metastatic cells 1, protein | 5.397 | 1.93E-03 | 2.244 |
| 100324_g_at | Amd2 | S-adenosylmethionine decarboxylase 2 | 5.341 | 2.02E-03 | 2.045 |
| 100030_at | Upp1 | Uridine phosphorylase 1 | 5.138 | 2.40E-03 | 1.730 |
| 99039_g_at | Adss | Adenylosuccinate synthetase, non muscle | 5.037 | 2.59E-03 | 1.994 |
| 100608_at | Sptlc1 | Serine palmitoyltransferase, long chain base subunit 1 | 5.011 | 2.64E-03 | 1.400 |
| 160568_at | Eno1 | Enolase 1, alpha non-neuron | 5.010 | 2.64E-03 | 2.316 |
| 96139_at | Gcs1 | Glucosidase 1 | 4.986 | 2.71E-03 | 1.452 |
| 92540_f_at | Srm | Spermidine synthase | 4.773 | 3.32E-03 | 2.570* |
| 96827_at | Cad | Carbamoyl-phosphate synthetase 2, aspartate transcarbamylase, and dihydroorotase | 4.698 | 3.55E-03 | 1.382 |
| 92824_at | Nme6 | Expressed in non-metastatic cells 6, protein | 4.622 | 3.80E-03 | 1.223 |
| 100576_at | Pafah1b3 | Platelet-activating factor acetylhydrolase, isoform 1b, alpha1 subunit | -7.299 | 5.31E-04 | 0.436 |
| 98527_at | Dci | Dodecenoyl-Coenzyme A delta isomerase (3,2 trans-enoyl-Coenyme A isomerase) | -6.844 | 6.86E-04 | 0.553 |
| 101000_at | Oaz2 | Ornithine decarboxylase antizyme 2 | -6.535 | 8.47E-04 | 0.380 |
| L09192_3_at | Pcx | Pyruvate carboxylase | -6.499 | 8.72E-04 | 0.408 |
| 161997_f_at | Aldh2 | Aldehyde dehydrogenase 2, mitochondrial | -6.330 | 9.77E-04 | 0.657 |
| 102035_at | Tpmt | Thiopurine methyltransferase | -6.033 | 1.21E-03 | 0.707 |
| 104509_at | Ch25h | Cholesterol 25-hydroxylase | -5.930 | 1.32E-03 | 0.511* |
| 100042_at | Hagh | Hydroxyacyl glutathione hydrolase | -5.650 | 1.61E-03 | 0.628 |
| 161243_f_at | Abhd8 | Abhydrolase domain containing 8 | -5.436 | 1.86E-03 | 0.594 |
| 101084_f_at | Dpm3 | Dolichyl-phosphate mannosyltransferase polypeptide 3 | -5.421 | 1.88E-03 | 0.535 |
| 100629_at | Gstm5 | Glutathione S-transferase, mu 5 | -5.357 | 1.99E-03 | 0.543 |
| 160337_at | Isyna1 | Myo-inositol 1-phosphate synthase A1 | -5.310 | 2.07E-03 | 0.434 |
| 95328_at | Fut9 | Fucosyltransferase 9 | -5.218 | 2.25E-03 | 0.827 |
| 93308_s_at | Pcx | Pyruvate carboxylase | -5.177 | 2.32E-03 | 0.490 |
| 102026_s_at | Chkb | Choline kinase beta | -5.146 | 2.37E-03 | 0.778 |
| 162148_r_at | Apom | Apolipoprotein M | -5.140 | 2.39E-03 | 0.897 |
| 95754_at | Mbtps1 | Membrane-bound transcription factor peptidase, site 1 | -4.960 | 2.78E-03 | 0.672 |
| 103032_at | Tpst1 | Protein-tyrosine sulfotransferase 1 | -4.878 | 2.99E-03 | 0.473 |
| 94282_at | Asah1 | N-acylsphingosine amidohydrolase 1 | -4.804 | 3.22E-03 | 0.616 |
| 98588_at | Fah | Fumarylacetoacetate hydrolase | -4.717 | 3.49E-03 | 0.558 |
| *Transport and trafficking processes* | | |  |  |  |
| 93330_at | Aqp1 | Aquaporin 1 | 11.526 | 6.86E-05 | 5.895 |
| 99329_at | Abcc1 | ATP-binding cassette, sub-family C (CFTR/MRP), member 1 | 7.745 | 3.96E-04 | 2.190 |
| 104007_at | Slc25a15 | Solute carrier family 25 (mitochondrial carrier ornithine transporter), member 15 | 6.599 | 8.05E-04 | 1.506 |
| 93993_at | Lman2 | Lectin, mannose-binding 2 | 5.620 | 1.64E-03 | 1.678 |
| 104475_at | Slc35e1 | Solute carrier family 35, member E1 | 5.341 | 2.02E-03 | 1.537 |
| 160409_at | Pitpna | Phosphatidylinositol transfer protein, alpha | 5.273 | 2.14E-03 | 1.514 |
| 95889_at | Arf3 | ADP-ribosylation factor 3 | 5.529 | 1.74E-03 | 1.292 |
| 93111_at | Kpnb1 | Karyopherin (importin) beta 1 | 5.192 | 2.30E-03 | 1.642 |
| 100417_at | Slc7a6 | Solute carrier family 7 (cationic amino acid transporter, y+ system), member 6 | 5.141 | 2.39E-03 | 1.428 |
| 103218_at | Slc10a3 | Solute carrier family 10 (sodium/bile acid cotransporter family), member 3 | 5.037 | 2.58E-03 | 1.570 |
| 95708_at | D3Ucla1 | DNA segment, Chr 3, University of California at Los Angeles 1 | 4.947 | 2.80E-03 | 1.777 |
| 101877_at | Slc31a1 | Solute carrier family 31, member 1 | 4.788 | 3.28E-03 | 1.712 |
| 98796_at | Mip | Major intrinsic protein of eye lens fiber | 4.718 | 3.49E-03 | 1.183 |
| 93609_at | Spg20 | Spastic paraplegia 20, spartin (Troyer syndrome) homolog (human) | -11.902 | 4.80E-05 | 0.714 |
| 93736_at | Tcn2 | Transcobalamin 2 | -6.069 | 1.18E-03 | 0.536 |
| 93045_at | Abcd3 | ATP-binding cassette, sub-family D (ALD), member 3 | -5.893 | 1.37E-03 | 0.405 |
| 100943_at | Slc1a4 | Solute carrier family 1 (glutamate/neutral amino acid transporter), member 4 | -5.328 | 2.04E-03 | 0.704 |
| 99872_s_at | Ftl1 | Ferritin light chain 1 | -5.115 | 2.44E-03 | 0.676 |
| 97313_at | Gdi1 | Guanosine diphosphate (GDP) dissociation inhibitor 1 | -4.734 | 3.44E-03 | 0.674 |
| 96894_at | Tmed4 | Transmembrane emp24 protein transport domain containing 4 | -4.733 | 3.45E-03 | 0.608 |
| 92598_at | Atp6v1b2 | ATPase, H+ transporting, V1 subunit B, isoform 2 | -4.722 | 3.48E-03 | 0.706 |
| 94549_at | Mfsd1 | Major facilitator superfamily domain containing 1 | -4.631 | 3.77E-03 | 0.581 |
| *Cell cycle and DNA replication* | | |  |  |  |
| 97197_r_at | AI506816 | Expressed sequence | 8.860 | 2.33E-04 | 2.648 |
| 97282_at | Mela | Melanoma antigen | 6.938 | 6.50E-04 | 1.471 |
| 101694_f_at | Myst2 | MYST histone acetyltransferase 2 | 6.688 | 7.66E-04 | 1.274 |
| 103821_at | Cdc6 | Cell division cycle 6 homolog (S. Cerevisiae) | 6.086 | 1.17E-03 | 4.340 |
| 97327_at | Fen1 | Flap structure specific endonuclease 1 | 6.056 | 1.19E-03 | 2.492 |
| 103057_at | Pold1 | Polymerase (DNA directed), delta 1, catalytic subunit | 5.023 | 2.62E-03 | 1.451 |
| 160699_at | Cdca5 | Cell division cycle associated 5 | 4.926 | 2.87E-03 | 1.269 |
| 92879_at | Ppm1g | Protein phosphatase 1G (formerly 2C), magnesium-dependent, gamma isoform | 4.693 | 3.57E-03 | 1.681 |
| 103212_at | Cdca7l | Cell division cycle associated 7 like | 4.611 | 3.85E-03 | 1.719 |
| 95100_at | Anapc5 | Anaphase-promoting complex subunit 5 | -7.445 | 4.85E-04 | 0.598 |
| 96217_at | Polb | Polymerase (DNA directed), beta | -7.364 | 5.08E-04 | 0.797 |
| 96710_at | H2afv | H2A histone family, member V | -6.700 | 7.57E-04 | 0.386 |
| 98789_at | Cdkn2a | Cyclin-dependent kinase inhibitor 2A | -6.009 | 1.24E-03 | 0.147# |
| 104471_at | Hdac6 | Histone deacetylase 6 | -5.800 | 1.46E-03 | 0.655* |
| 97908_at | Rmnd5a | Required for meiotic nuclear division 5 homolog A (S. cerevisiae) | -4.815 | 3.19E-03 | 0.483 |
| *Immunity and defense* | | |  |  |  |
| 98501_at | Il1rl1 | Interleukin 1 receptor-like 1 | 17.278 | 4.58E-06 | 22.80* |
| 93861_f_at | Ctse | Cathepsin E | 7.814 | 3.82E-04 | 2.620# |
| 93860_i_at | Ctse | Cathepsin E | 7.661 | 4.19E-04 | 2.183# |
| 98500_at | Il1rl1 | Interleukin 1 receptor-like 1 | 7.039 | 6.13E-04 | 10.22* |
| 98008_at | Cx3cl1 | Chemokine (C-X3-C motif) ligand 1 | 6.128 | 1.13E-03 | 4.853 |
| 102712_at | Saa3 | Serum amyloid A 3 | -5.553 | 1.70E-03 | 0.184 |
| 160511_at | Cxcl12 | Chemokine (C-X-C motif) ligand 12 | -4.724 | 3.47E-03 | 0.261 |
| *Response to IFN* | | |  |  |  |
| 104760_at | Ifrd2 | Interferon-related developmental regulator 2 | 6.099 | 1.16E-03 | 1.860* |
| 162447_f_at | Mvp | Major vault protein | -5.533 | 1.73E-03 | 0.763 |
| *Cell adhesion and migration* | | |  |  |  |
| 100484_at | Mmp13 | Matrix metallopeptidase 13 | 16.024 | 9.15E-06 | 53.31* |
| 94724_at | Mmp10 | Matrix metallopeptidase 10 | 12.024 | 4.58E-05 | 11.049 |
| 101882_s_at | Col18a1 | Procollagen, type XVIII, alpha 1 | 11.601 | 6.18E-05 | 2.432 |
| 102280_at | Pcdh7 | Protocadherin 7 | 9.120 | 1.97E-04 | 6.074 |
| 98833_at | Mmp3 | Matrix metallopeptidase 3 | 8.283 | 3.00E-04 | 18.795 |
| 101881_g_at | Col18a1 | Procollagen, type XVIII, alpha 1 | 6.438 | 9.15E-04 | 4.359 |
| 96277_at | Arpc5 | Actin related protein 2/3 complex, subunit 5 | 6.312 | 9.95E-04 | 1.342 |
| 162483_f_at | Col18a1 | Procollagen, type XVIII, alpha 1 | 5.265 | 2.16E-03 | 2.135 |
| 98423_at | Gjb2 | Gap junction membrane channel protein beta 2 | 5.140 | 2.39E-03 | 16.653 |
| 100065_r_at | Gja1 | Gap junction membrane channel protein alpha 1 | 4.882 | 2.98E-03 | 7.356 |
| 99638_at | Col18a1 | Procollagen, type XVIII, alpha 1 | 4.812 | 3.21E-03 | 1.864 |
| 97904_at | Actr3 | ARP3 actin-related protein 3 homolog (yeast) | 4.781 | 3.31E-03 | 1.938 |
| 98402_at | Macf1 | Microtubule-actin crosslinking factor 1 | -7.029 | 6.15E-04 | 0.480 |
| 94449_at | Pcdhga12 | Protocadherin gamma subfamily A, 12 | -4.877 | 3.00E-03 | 0.675 |
| 94561_at | Lima1 | LIM domain and actin binding 1 | -4.862 | 3.05E-03 | 0.580 |
| *RNA metabolism and processing* | | |  |  |  |
| 98923_at | Rcl1 | RNA terminal phosphate cyclase-like 1 | 5.770 | 1.48E-03 | 1.457 |
| 160208_at | Sf3b3 | Splicing factor 3b, subunit 3 | 5.527 | 1.75E-03 | 1.237 |
| 102400_at | AU014645 | Expressed sequence AU014645 | 5.464 | 1.82E-03 | 1.627 |
| 99954_at | Rrp9 | RRP9, small subunit (SSU) processome component, homolog (yeast) | 5.411 | 1.90E-03 | 1.656 |
| 99182_at | Rpp14 | Ribonuclease P 14 subunit (human) | 5.248 | 2.19E-03 | 1.229 |
| 103056_at | Utp18 | UTP18, small subunit (SSU) processome component, homolog (yeast) | 4.770 | 3.33E-03 | 2.109 |
| 94017_s_at | Sfrs2 | Splicing factor, arginine/serine-rich 2 (SC-35) | 4.697 | 3.56E-03 | 2.031 |
| 103888_at | Rbpms | RNA binding protein gene with multiple splicing | -6.528 | 8.53E-04 | 0.449 |
| 102399_at | Rbpms | RNA binding protein gene with multiple splicing | -5.910 | 1.35E-03 | 0.415 |
| 104231_at | Hnrph3 | Heterogeneous nuclear ribonucleoprotein H3 | -4.829 | 3.15E-03 | 0.628 |
| *Cell development and differentiation* | | |  |  |  |
| 100127_at | Crabp2 | Cellular retinoic acid binding protein II | 17.124 | 6.86E-06 | 11.97# |
| 160162_at | Tagln2 | Transgelin 2 | 11.543 | 6.63E-05 | 3.134* |
| 160119_at | Mgp | Matrix Gla protein | 8.318 | 2.91E-04 | 2.843 |
| 103957_at | Tfrc | Transferrin receptor | 8.317 | 2.93E-04 | 2.393 |
| 103830_at | Snai1 | Snail homolog 1 (Drosophila) | 6.631 | 7.96E-04 | 2.411 |
| X57349_3_at | Tfrc | Transferrin receptor | 6.338 | 9.68E-04 | 2.302 |
| 92317_at | Elavl2 | ELAV (embryonic lethal, abnormal vision, Drosophila)-like 2 (Hu antigen B) | 6.251 | 1.03E-03 | 1.319 |
| 94187_at | Gsc | Goosecoid | 6.044 | 1.20E-03 | 1.337 |
| 104614_at | Gpc1 | Glypican 1 | 5.826 | 1.44E-03 | 3.235 |
| 95706_at | Lgals3 | Lectin, galactose binding, soluble 3 | 5.544 | 1.72E-03 | 3.978 |
| 93184_at | Tbl2 | Transducin (beta)-like 2 | 5.095 | 2.48E-03 | 1.420 |
| 93228_at | Hells | Helicase, lymphoid specific | 5.040 | 2.57E-03 | 1.968 |
| 93866_s_at | Mgp | Matrix Gla protein | 4.947 | 2.80E-03 | 23.385 |
| 100050_at | Id1 | Inhibitor of DNA binding 1 | 4.905 | 2.94E-03 | 3.490 |
| 95050_at | Chordc1 | Cysteine and histidine-rich domain (CHORD)-containing, zinc-binding protein 1 | 4.799 | 3.24E-03 | 1.385* |
| 97487_at | Serpine2 | Serine (or cysteine) peptidase inhibitor, clade E, member 2 | 4.798 | 3.25E-03 | 5.029 |
| 93550_at | Csrp2 | Cysteine and glycine-rich protein 2 | 4.756 | 3.38E-03 | 2.087 |
| 103824_at | Wfs1 | Wolfram syndrome 1 homolog (human) | 4.688 | 3.59E-03 | 1.414 |
| 97845_at | Wdr5 | WD repeat domain 5 | 4.676 | 3.61E-03 | 1.264 |
| 99964_at | Vdr | Vitamin D receptor | -8.073 | 3.32E-04 | 0.534* |
| 95557_at | Bmp1 | Bone morphogenetic protein 1 | -7.473 | 4.67E-04 | 0.535 |
| 100992_at | Phc1 | Polyhomeotic-like 1 (Drosophila) | -5.996 | 1.25E-03 | 0.594 |
| 100876_at | Fez1 | Fasciculation and elongation protein zeta 1 (zygin I) | -5.992 | 1.25E-03 | 0.322 |
| 101095_at | Mfap2 | Microfibrillar-associated protein 2 | -5.867 | 1.40E-03 | 0.119 |
| 98555_at | Ttc3 | Tetratricopeptide repeat domain 3 | -5.369 | 1.96E-03 | 0.465 |
| *Protein biosynthesis and ribosome biogenesis* | | |  |  |  |
| 97824_at | Nola2 | Nucleolar protein family A, member 2 | 7.494 | 4.55E-04 | 2.907 |
| 93488_at | Tsr1 | TSR1, 20S rRNA accumulation, homolog (yeast) | 6.728 | 7.46E-04 | 2.966 |
| 104702_at | Wdr46 | WD repeat domain 46 | 5.404 | 1.91E-03 | 1.462 |
| 94253_at | Eif2s1 | Eukaryotic translation initiation factor 2, subunit 1 alpha | 5.362 | 1.97E-03 | 2.365* |
| 94260_at | Larp1 | La ribonucleoprotein domain family, member 1 | 5.287 | 2.11E-03 | 1.566 |
| 98605_at | Wars | Tryptophanyl-tRNA synthetase | 5.225 | 2.22E-03 | 1.549 |
| 97414_at | Noc4l | Nucleolar complex associated 4 homolog (S. Cerevisiae) | 4.721 | 3.48E-03 | 1.605 |
| 95054_at | Tras | Threonyl-tRNA synthetase | 4.694 | 3.56E-03 | 1.611 |
| 99599_s_at | Ptov1 | Prostate tumor over expressed gene 1 | -10.896 | 8.69E-05 | 0.481 |
| 101573_f_at | Rpl27a | Ribosomal protein l27a | -6.396 | 9.36E-04 | 0.803 |
| 97647_at | Rps16 | Ribosomal protein S16 | -5.871 | 1.39E-03 | 0.744 |
| 94952_at | Igf2bp2 | Insulin-like growth factor 2 mRNA binding protein 2 | -5.723 | 1.54E-03 | 0.553 |
| 100758_at | Rps28 | Ribosomal protein S28 | -5.259 | 2.16E-03 | 0.672 |
| 101213_at | Arbp | Acidic ribosomal phosphoprotein P0 | -4.669 | 3.64E-03 | 0.750 |
| *Apoptosis* | | |  |  |  |
| 98056_at | Phlda3 | Pleckstrin homology-like domain, family A, member 3 | 7.523 | 4.51E-04 | 2.927 |
| 103217_at | Cflar | CASP8 and FADD-like apoptosis regulator | 4.621 | 3.80E-03 | 1.325 |
| 100442_at | Tbrg4 | Transforming growth factor beta regulated gene 4 | 4.604 | 3.88E-03 | 1.599* |
| 96765_at | Peg3 | Paternally expressed 3 | -6.163 | 1.10E-03 | 0.062* |
| 98424_at | Ptpn13 | Protein tyrosine phosphatase, non-receptor type 13 | -6.071 | 1.17E-03 | 0.372* |
| 160696_at | Tia1 | Cytotoxic granule-associated RNA binding protein 1 | -5.935 | 1.31E-03 | 0.583 |
| 103029_at | Pdcd4 | Programmed cell death 4 | -5.315 | 2.06E-03 | 0.269 |
| 100988_at | Bcl2l11 | BCL2-like 11 (apoptosis facilitator) | -5.129 | 2.41E-03 | 0.635 |
| 101451_at | Peg3 | Paternally expressed 3 | -4.638 | 3.75E-03 | 0.152* |
| *Ubiquitin cycle* | | |  |  |  |
| 101741_at | Psmb5 | Proteasome (prosome, macropain) subunit, beta type 5 | 5.324 | 2.04E-03 | 1.433 |
| 93101_s_at | Nedd4 | Neural precursor cell expressed, developmentally down-regulted gene 4 | -6.553 | 8.40E-04 | 0.407* |
| 97285_f_at | Ubxd1 | UBX domain containing 1 | -6.131 | 1.13E-03 | 0.542 |
| *Electron transport and energy production* | | |  |  |  |
| 96878_at | Cyb5b | Cytochrome b5 type B | 8.869 | 2.29E-04 | 1.613 |
| 94207_at | Pdia6 | Protein disulfide isomerase associated 6 | 5.115 | 2.44E-03 | 2.148 |
| 94208_at | Pdia6 | Protein disulfide isomerase associated 6 | 4.911 | 2.91E-03 | 2.395 |
| 160383_at | Cox7a2l | Cytochrome c oxidase subunit viia polypeptide 2-like | -7.802 | 3.84E-04 | 0.469 |
| 104153_at | Ivd | Isovaleryl coenzyme A dehydrogenase | -6.492 | 8.79E-04 | 0.607 |
| 95652_at | Ndufa7 | NADH dehydrogenase (ubiquinone) 1 alpha subcomplex, 7 (B14.5a) | -5.427 | 1.87E-03 | 0.677 |
| 99979_at | Cyp1b1 | Cytochrome P450, family 1, subfamily b, polypeptide 1 | -4.975 | 2.75E-03 | 0.154* |
| 160237_at | Ndufa6 | NADH dehydrogenase (ubiquinone) 1 alpha subcomplex, 6 (B14) | -4.668 | 3.65E-03 | 0.361 |
| *Cytoskeleton organization and biogenesis* | | |  |  |  |
| 96033_at | Sdc1 | Syndecan 1 | 9.601 | 1.51E-04 | 2.494 |
| 94835_f_at | Tubb2 | Tubulin, beta 2 | 5.210 | 2.27E-03 | 2.480 |
| 98559_at | Smtn | Smoothelin | 4.945 | 2.81E-03 | 1.518 |
| 98454_at | Palm | Paralemmin | -12.675 | 3.43E-05 | 0.675 |
| 98129_at | Tmsb10 | Thymosin, beta 10 | -6.348 | 9.61E-04 | 0.698 |
| 93635_at | Kif3c | Kinesin family member 3C | -4.764 | 3.35E-03 | 0.749 |
| 94863_r_at | Dncl2a | Dynein, cytoplasmic, light chain 2A | -4.645 | 3.73E-03 | 0.654 |
| *Cell growth and proliferation* | | |  |  |  |
| 98802_at | Ereg | Epiregulin | 6.058 | 1.19E-03 | 5.492 |
| 95137_at | Tmem97 | Transmembrane protein 97 | 5.536 | 1.73E-03 | 2.598 |
| 97951_s_at | Tsc2 | Tuberous sclerosis 2 | -11.007 | 8.47E-05 | 0.571 |
| 96920_at | Htra1 | Htra serine peptidase 1 | -10.720 | 9.38E-05 | 0.123 |
| 104390_at | Anp32a | Acidic (leucine-rich) nuclear phosphoprotein 32 family, member A | -7.848 | 3.75E-04 | 0.483 |
| 93780_at | Them2 | Thioesterase superfamily member 2 | -7.589 | 4.35E-04 | 0.684 |
| 94396_at | Ing1 | Inhibitor of growth family, member 1 | -5.814 | 1.45E-03 | 0.684 |
| 97953_g_at | Tsc2 | Tuberous sclerosis 2 | -5.213 | 2.26E-03 | 0.434 |
| 104006_at | Eps15 | Epidermal growth factor receptor pathway substrate 15 | -5.207 | 2.28E-03 | 0.727 |
| *Angiogenesis* | | |  |  |  |
| 103520_at | Vegfa | Vascular endothelial growth factor A | 11.443 | 7.55E-05 | 3.035* |
| 92560_g_at | Vcam1 | Vascular cell adhesion molecule 1 | -9.555 | 1.58E-04 | 0.442 |
| 92365_at | Figf | c-fos induced growth factor | -4.667 | 3.65E-03 | 0.113 |
| *Proteolysis and peptidolysis* | | |  |  |  |
| 96211_at | Dpp8 | Dipeptidylpeptidase 8 | -4.951 | 2.80E-03 | 0.572 |
| 104036_at | Dpp7 | Dipeptidylpeptidase 7 | -4.940 | 2.83E-03 | 0.529 |
| *Protein folding* | | |  |  |  |
| 99082_at | Fkbp10 | FK506 binding protein 10 | 7.822 | 3.78E-04 | 1.681 |
| 98572_at | Dnajb11 | Dnaj (Hsp40) homolog, subfamily B, member 11 | 6.516 | 8.67E-04 | 2.052 |
| 100352_at | Hspa4 | Heat shock protein 4 | 6.489 | 8.81E-04 | 1.353 |
| 101955_at | Hspa5 | Heat shock 70kd protein 5 (glucose-regulated protein) | 6.257 | 1.02E-03 | 2.669 |
| 102414_i_at | Dnajc3 | Dnaj (Hsp40) homolog, subfamily C, member 3 | 5.817 | 1.44E-03 | 2.009* |
| 100353_g_at | Hspa4 | Heat shock protein 4 | 5.015 | 2.63E-03 | 1.760 |
| 96679_at | Dnajb9 | Dnaj (Hsp40) homolog, subfamily B, member 9 | 4.613 | 3.84E-03 | 2.312 |
| 95699_f_at | Dnajc8 | Dnaj (Hsp40) homolog, subfamily C, member 8 | -6.694 | 7.60E-04 | 0.785 |
| *Blood coagulation* | | |  |  |  |
| 92978_s_at | Serpinb2 | Serine (or cysteine) peptidase inhibitor, clade B, member 2 | 14.349 | 2.06E-05 | 33.198 |
| 94147_at | Serpine1 | Serine (or cysteine) peptidase inhibitor, clade E, member 1 | 10.108 | 1.26E-04 | 28.18* |
| 97529_at | Anxa8 | Annexin A8 | 7.124 | 5.74E-04 | 14.896 |
| *DNA repair* | | |  |  |  |
| 102792_at | Ung | Uracil DNA glycosylase | 4.704 | 3.54E-03 | 1.501 |
| *Synaptic transmission* | | |  |  |  |
| 93586_at | Syngr2 | Synaptogyrin 2 | 4.691 | 3.57E-03 | 1.499 |
| 93011_at | Gabarapl1 | Gamma-aminobutyric acid (GABA(A)) receptor-associated protein-like 1 | -5.813 | 1.45E-03 | 0.365 |
| 92184_at | Dtna | Dystrobrevin alpha | -4.988 | 2.70E-03 | 0.749 |
| *Sexual reproduction* | | |  |  |  |
| 161629_i_at | Afp | Alpha fetoprotein | 4.800 | 3.23E-03 | 1.490 |
| *Chromatin assembly or disassembly* | | |  |  |  |
| 104259_at | Cbx5 | Chromobox homolog 5 (Drosophila HP1a) | 4.966 | 2.76E-03 | 1.473 |
| *Unknown* | | |  |  |  |
| 100717_at | U90926 | cDNA sequence U90926 | 9.369 | 1.72E-04 | 9.036 |
| 100471_at | 1110036O03Rik | RIKEN cDNA 1110036O03 gene | 8.848 | 2.38E-04 | 1.987 |
| 161050_at | NA | NA | 8.415 | 2.77E-04 | 1.950 |
| 94995_at | A030007L17Rik | RIKEN cDNA A030007L17 gene | 7.382 | 5.03E-04 | 3.020 |
| 94899_at | AA536749 | Expressed sequence AA536749 | 5.409 | 1.90E-03 | 1.866 |
| 99366_at | Pqlc3 | PQ loop repeat containing | 4.976 | 2.75E-03 | 2.041 |
| 160801_at | Pqlc1 | PQ loop repeat containing 1 | 4.876 | 3.01E-03 | 1.571 |
| 95523_at | 6530401D17Rik | RIKEN cDNA 6530401D17 gene | 4.827 | 3.15E-03 | 1.879 |
| 102920_at | 9130422G05Rik | RIKEN cDNA 9130422G05 gene | 4.673 | 3.62E-03 | 2.678 |
| 160382_at | 1110014J01Rik | RIKEN cDNA 1110014J01 gene | 4.633 | 3.76E-03 | 1.554 |
| 96686_i_at | 2010100O12Rik | RIKEN cDNA 2010100O12 gene | -8.520 | 2.70E-04 | 0.531 |
| 95123_at | 4930566A11Rik | RIKEN cDNA 4930566A11 gene | -7.471 | 4.71E-04 | 0.748 |
| 160563_at | Serf2 | Small EDRK-rich factor 2 | -6.926 | 6.54E-04 | 0.651 |
| 96615_at | Ypel3 | Yippee-like 3 (Drosophila) | -5.978 | 1.28E-03 | 0.355 |
| 160709_at | 1110001A16Rik | RIKEN cDNA 1110001A16 gene | -5.671 | 1.59E-03 | 0.738 |
| 96902_at | Ccdc53 | Coiled-coil domain containing 53 | -5.587 | 1.67E-03 | 0.688 |
| 92542_at | D4Wsu53e | DNA segment, Chr 4, Wayne State University 53, expressed | -5.538 | 1.73E-03 | 0.252 |
| 98495_at | 5033414D02Rik | RIKEN cDNA 5033414D02 gene | -5.261 | 2.16E-03 | 0.688 |
| 100465_i_at | GM1673 | Gene model 1673, (NCBI) | -5.245 | 2.19E-03 | 0.303 |
| 95396_at | NA | DNA cytosine methyltransferase mRNA | -5.206 | 2.28E-03 | 0.104 |
| 102233_at | 4833442J19Rik | RIKEN cDNA 4833442J19 gene | -4.913 | 2.90E-03 | 0.628 |
| 95619_at | 1700040I03Rik | RIKEN cDNA 1700040I03 gene | -4.792 | 3.27E-03 | 0.671 |
| 99154_s_at | 1810020D17Rik | RIKEN cDNA 1810020D17 gene | -4.682 | 3.60E-03 | 0.674 |

**Table S9. Differential gene expression in serum-starved, H-*ras*^-/-^/N-*ras*^-/-^ fibroblasts after incubation of cell cultures in the presence of serum for 8 hours.** List of 1074 differentially expressed probesets (1001 different genes) identified by means of SAM contrast (FDR=0,09) comparing the microarray-generated transcriptional profile of 24h serum-starved, WT fibroblasts to that of similarly starved, H-*ras*^-/-^/N-*ras*^-/-^ fibroblast cultures submitted to subsequent incubation in the presence of 20% FBS for 8 hours. To concentrate on loci exclusively regulated by Ras, genes sharing similar values of differential expression between the WT and H-*ras*^-/-^/N-*ras*^-/-^fibroblasts (ratio of the R-fold values in their respective lists < 1.5) were excluded from this list. The differentially expressed loci are identified by Affymetrix probeset ID, gene name and symbol, and are listed according to their degree of overexpression or repression, quantitated by R.fold value. The * and # symbols in the R.fold column denote independent validation of the transcriptional data obtained by means of quantitative RT-PCR or WB immunoblot, respectively. d(i) is a parameter measuring the statistical distance separating the calculated expression value of each gene probeset from the null hypothesis (no-change). p-value is an statistical measure indicating the probability of random expression for that probeset. R fold is the log2 value of the fold change measuring the overexpression or repression of the probesets in the collection of microarrays.

| **Probeset ID** | **Gene symbol** | **Gene name** | **d(i)** | **p-value** | **R.fold** |
| --- | --- | --- | --- | --- | --- |
| *Signal transduction* | |  |  |  |  |
| 94853_at | Gnb1 | Guanine nucleotide binding protein, beta 1 | 11.758 | 1.78E-04 | 2.356 |
| 161609_at | Rgs16 | Regulator of G-protein signaling 16 | 9.183 | 5.06E-04 | 3.968* |
| 97890_at | Sgk | Serum/glucocorticoid regulated kinase | 9.053 | 5.47E-04 | 5.227* |
| 97991_at | Kras | v-Ki-ras2 Kirsten rat sarcoma viral oncogene homolog | 7.963 | 9.17E-04 | 2.020 |
| 98602_at | Rangap1 | RAN GTPase activating protein 1 | 7.585 | 1.12E-03 | 1.748* |
| 94378_at | Rgs16 | Regulator of g-protein 82raline82ng 16 | 7.470 | 1.18E-03 | 4.461* |
| 98603_s_at | Rangap1 | RAN GTPase activating protein 1 | 7.276 | 1.35E-03 | 1.885 |
| 103642_at | G3bp1 | Ras-GTPase-activating protein SH3-domain binding protein | 6.884 | 1.70E-03 | 2.921 |
| 94828_at | Oprs1 | Opioid receptor, sigma 1 | 6.703 | 1.86E-03 | 1.624 |
| 104697_at | Rhoj | Ras homolog gene family, member j | 6.645 | 1.94E-03 | 1.941 |
| 97369_g_at | Akap1 | A kinase (PRKA) anchor protein 1 | 6.588 | 2.02E-03 | 1.493 |
| 95721_at | Mapkapk2 | MAP kinase-activated protein kinase 2 | 6.426 | 2.21E-03 | 1.488 |
| 100309_at | Met | Met proto-oncogene | 6.321 | 2.37E-03 | 1.740 |
| 99005_at | Rad1 | RAD1 homolog (S. pombe) | 6.126 | 2.74E-03 | 1.294 |
| 93680_at | Stk10 | Serine/threonine kinase 10 | 6.033 | 2.86E-03 | 2.066 |
| 95523_at | 6530401D17Rik | RIKEN cDNA 6530401D17 gene | 5.989 | 2.94E-03 | 2.022 |
| 104112_at | Rab21 | RAB21, member RAS oncogene family | 5.890 | 3.13E-03 | 1.541 |
| 95001_at | Akap8 | A kinase (PRKA) anchor protein 8 | 5.852 | 3.23E-03 | 1.279 |
| 97711_at | Npr3 | Natriuretic peptide receptor 3 | 5.836 | 3.27E-03 | 2.448 |
| 97979_at | Ppp1r7 | Protein phosphatase 1, regulatory (inhibitor) subunit 7 | 5.727 | 3.51E-03 | 1.830 |
| 103406_at | Xab1 | XPA binding protein 1 | 5.707 | 3.56E-03 | 1.289 |
| 93315_at | Map2k3 | Mitogen activated protein kinase kinase 3 | 5.544 | 3.96E-03 | 2.352* |
| 93314_g_at | Map2k3 | Mitogen activated protein kinase kinase 3 | 5.394 | 4.33E-03 | 1.371* |
| 101571_g_at | Igfbp4 | Insulin-like growth factor binding protein 4 | 5.173 | 5.11E-03 | 4.665 |
| 92882_at | Rab1 | RAB1, member RAS oncogene family | 5.103 | 5.43E-03 | 1.503 |
| 92875_s_at | Cops7a | COP9 (constitutive photomorphogenic) homolog, subunit 7a (Arabidopsis thaliana) | 5.097 | 5.46E-03 | 1.267 |
| 99960_at | Map2k4 | Mitogen activated protein kinase kinase 4 | 5.018 | 5.76E-03 | 1.547 |
| 104047_at | Mapk8 | Mitogen activated protein kinase 8 | 5.001 | 5.85E-03 | 1.751 |
| 104527_at | Rad51 | RAD51 homolog (S. Cerevisiae) | 4.993 | 5.89E-03 | 1.956 |
| 99655_at | Sec11l1 | Sec11-like 1 (S. cerevisiae) | 4.976 | 5.98E-03 | 1.381 |
| 160100_at | Efhd2 | EF hand domain containing 2 | 4.899 | 6.32E-03 | 1.277 |
| 93602_at | Rps6ka4 | Ribosomal protein S6 kinase, polypeptide 4 | 4.860 | 6.49E-03 | 2.841 |
| 101030_at | Rhob | Ras homolog gene family, member b | 4.822 | 6.71E-03 | 2.440 |
| 97308_at | Tmem11 | Transmembrane protein 11 | 4.759 | 7.01E-03 | 1.613 |
| 101254_at | Ran | RAN, member RAS oncogene family | 4.627 | 7.78E-03 | 1.966 |
| 102821_s_at | Rasl2-9 | RAS-like, family 2, locus 9 | 4.496 | 8.64E-03 | 2.311 |
| 98948_at | Gnl3 | Guanine nucleotide binding protein-like 3 (nucleolar) | 4.493 | 8.67E-03 | 2.070 |
| 160989_r_at | Npr3 | Natriuretic peptide receptor 3 | 4.444 | 9.03E-03 | 2.212 |
| 103332_at | Gpr125 | G protein-coupled receptor 125 | 4.378 | 9.57E-03 | 1.234 |
| 92539_at | S100a10 | S100 calcium binding protein A10 (calpactin) | 4.375 | 9.59E-03 | 1.542 |
| 95666_at | Cops8 | COP9 (constitutive photomorphogenic) homolog, subunit 8 (Arabidopsis thaliana) | 4.367 | 9.68E-03 | 1.765 |
| 93676_at | Rad51ap1 | RAD51 associated protein 1 | 4.364 | 9.70E-03 | 1.533 |
| 104180_at | Rac3 | RAS-related C3 botulinum substrate 3 | 4.166 | 1.16E-02 | 1.208 |
| 97367_at | Akap1 | A kinase (PRKA) anchor protein 1 | 4.149 | 1.18E-02 | 1.471 |
| 99510_at | Prkcb1 | Protein kinase C, beta 1 | 4.138 | 1.19E-02 | 1.093 |
| 99806_at | Npr3 | Natriuretic peptide receptor 3 | 4.096 | 1.23E-02 | 1.465 |
| 94784_at | Iqgap3 | IQ motif containing GTPase activating protein 3 | 4.086 | 1.24E-02 | 1.506 |
| 160890_at | Lbr | Lamin B receptor | 4.066 | 1.26E-02 | 1.497 |
| 101582_at | Gnl2 | Guanine nucleotide binding protein-like 2 (nucleolar) | 3.993 | 1.35E-02 | 1.214 |
| 93732_f_at | Rgs19ip1 | Regulator of g-protein 83raline83ng 19 interacting protein 1 | 3.978 | 1.36E-02 | 1.326 |
| 99978_s_at | Mapk14 | Mitogen activated protein kinase 14 | 3.966 | 1.38E-02 | 1.609 |
| 92434_at | Traip | TRAF-interacting protein | 3.961 | 1.38E-02 | 1.350 |
| 103923_at | Gpr137B | G protein-coupled receptor 137B | 3.954 | 1.39E-02 | 1.749 |
| 95438_at | Rhot1 | Ras homolog gene family, member t1 | 3.946 | 1.40E-02 | 1.250 |
| 104179_at | Arf6 | ADP-ribosylation factor 6 | 3.925 | 1.43E-02 | 1.997 |
| 104152_at | Stk40 | Serine/threonine kinase 40 | 3.829 | 1.56E-02 | 1.226 |
| 97699_at | Plxna1 | Plexin A1 | 3.788 | 1.62E-02 | 1.362 |
| 102259_at | Ywhag | 3-monooxygenase/tryptophan 5-monooxygenase activation protein, gamma polypeptide | 3.748 | 1.68E-02 | 2.256 |
| 96700_r_at | Rap1b | RAS related protein 1b | 3.732 | 1.70E-02 | 1.383 |
| 93070_at | Ranbp5 | RAN binding protein 5 | 3.729 | 1.70E-02 | 3.227* |
| 101016_at | Arf1 | ADP-ribosylation factor 1 | 3.726 | 1.71E-02 | 1.446 |
| 104339_at | Pygo2 | Pygopus 2 | 3.703 | 1.75E-02 | 1.170 |
| 98573_r_at | Ranbp1 | RAN binding protein 1 | 3.668 | 1.81E-02 | 1.706 |
| 102942_at | Specc1 | Sperm antigen with calponin homology and coiled-coil domains 1 | 3.667 | 1.81E-02 | 2.509 |
| 101362_at | Mapk9 | Mitogen activated protein kinase 9 | 3.656 | 1.82E-02 | 1.204 |
| 94947_g_at | Map3k3 | Mitogen activated protein kinase kinase kinase 3 | 3.650 | 1.83E-02 | 1.347 |
| 161817_f_at | Spsb1 | Spla/ryanodine receptor domain and SOCS box containing 1 | 3.642 | 1.84E-02 | 4.203 |
| 160925_at | **Nras** | **Neuroblastoma ras oncogene** | -16.784 | 4.12E-05 | 0.358 |
| 103833_at | Hipk2 | Homeodomain interacting protein kinase 2 | -15.200 | 5.95E-05 | 0.673 |
| 94362_at | **Nras** | **Neuroblastoma ras oncogene** | -11.456 | 2.01E-04 | 0.154 |
| 93702_at | Ptpn23 | Protein tyrosine phosphatase, non-receptor type 23 | -9.447 | 4.21E-04 | 0.647 |
| 97058_f_at | Rab33b | RAB33B, member of RAS oncogene family | -9.147 | 5.12E-04 | 0.749 |
| 92619_at | Wbp1 | WW domain binding protein 1 | -7.290 | 1.33E-03 | 0.765 |
| 93591_at | Pef1 | Penta-EF hand domain containing 1 | -7.242 | 1.37E-03 | 0.632 |
| 100056_at | Fbxw2 | F-box and WD-40 domain protein 2 | -6.900 | 1.67E-03 | 0.719 |
| 96132_at | Apcdd1 | Adenomatosis polyposis coli down-regulated 1 | -6.848 | 1.72E-03 | 0.325 |
| 103330_at | Rabgap1 | RAB gtpase activating protein 1 | -6.258 | 2.47E-03 | 0.566 |
| 104432_at | Rnd2 | Rho family GTPase 2 | -5.918 | 3.06E-03 | 0.675 |
| 160941_at | Pde8a | Phosphodiesterase 8A | -5.848 | 3.24E-03 | 0.551 |
| 96767_at | Mbc2 | Membrane bound C2 domain containing protein | -5.728 | 3.51E-03 | 0.576 |
| 160747_at | Rgs3 | Regulator of G-protein 83raline83ng 3 | -5.611 | 3.80E-03 | 0.651 |
| 94069_r_at | Ccdc47 | Coiled-coil domain containing 47 | -5.583 | 3.87E-03 | 0.898 |
| 93826_at | Ppp2r5a | Protein phosphatase 2, regulatory subunit B (B56), alpha isoform | -5.527 | 4.01E-03 | 0.363* |
| 92698_at | Mertk | c-mer proto-oncogene tyrosine kinase | -5.433 | 4.23E-03 | 0.725 |
| 103592_at | Map2k5 | Mitogen activated protein kinase kinase 5 | -5.534 | 3.99E-03 | 0.540 |
| 100530_at | Ralgds | Ral guanine nucleotide dissociation stimulator | -5.157 | 5.17E-03 | 0.352* |
| 101947_at | Akap8l | A kinase (PRKA) anchor protein 8-like | -5.099 | 5.45E-03 | 0.401 |
| 95516_at | Rab9 | RAB9, member RAS oncogene family | -5.061 | 5.58E-03 | 0.225 |
| 104208_at | Pik4ca | Phosphatidylinositol 4-kinase, catalytic, alpha polypeptide | -5.016 | 5.77E-03 | 0.848 |
| 94087_at | Prkcm | Protein kinase C, mu | -4.998 | 5.87E-03 | 0.695 |
| 103699_i_at | Frat2 | Frequently rearranged in advanced T-cell lymphomas 2 | -4.992 | 5.90E-03 | 0.632 |
| 104358_at | D10Ertd610e | DNA segment, Chr 10, ERATO Doi 610, expressed | -4.848 | 6.56E-03 | 0.642 |
| 99596_f_at | Gnai2 | Guanine nucleotide binding protein, alpha inhibiting 2 | -4.772 | 6.93E-03 | 0.719 |
| 96911_at | Gnb2 | Guanine nucleotide binding protein, beta 2 | -4.722 | 7.23E-03 | 0.791 |
| 97358_at | Lphn1 | Latrophilin 1 | -4.684 | 7.45E-03 | 0.745 |
| 96799_at | Fbxw5 | F-box and WD-40 domain protein 5 | -4.646 | 7.65E-03 | 0.825 |
| 99505_at | Rab4b | RAB4B, member RAS oncogene family | -4.521 | 8.49E-03 | 0.696 |
| 104425_at | Inadl | Inad-like (drosophila) | -4.458 | 8.92E-03 | 0.821 |
| 161811_f_at | Ptpra | Protein tyrosine phosphatase, receptor type, A | -4.427 | 9.16E-03 | 0.816 |
| 102759_at | Pik3r2 | Phosphatidylinositol 3-kinase, regulatory subunit, polypeptide 2 (p85 beta) | -4.329 | 1.00E-02 | 0.791* |
| 102356_at | Wdr23 | WD repeat domain 23 | -4.276 | 1.05E-02 | 0.608 |
| 93347_at | Rab24 | RAB24, member RAS oncogene family | -4.263 | 1.07E-02 | 0.786 |
| 161067_at | Trib3 | Tribbles homolog 3 (Drosophila) | -4.245 | 1.08E-02 | 0.469 |
| *Transcription* | |  |  |  |  |
| 104491_at | Phtf2 | Putative homeodomain transcription factor 2 | 19.511 | 2.29E-05 | 1.330 |
| 98021_at | Praf1 | Polymerase (RNA) I associated factor 1 | 12.436 | 1.26E-04 | 2.013 |
| 162269_at | Zfp422 | Zinc finger protein 422 | 11.447 | 2.04E-04 | 1.500 |
| 103393_at | Pspc1 | Paraspeckle protein 1 | 10.718 | 2.52E-04 | 2.436 |
| 94064_at | Zfp91 | Zinc finger protein 91 | 9.675 | 3.73E-04 | 1.476 |
| 104582_g_at | Zdhhc6 | Zinc finger, DHHC domain containing 6 | 9.466 | 4.14E-04 | 1.513* |
| 102363_r_at | Junb | Jun-B oncogene | 8.808 | 6.11E-04 | 1.907 |
| 102371_at | Nr4a1 | Nuclear receptor subfamily 4, group A, member 1 | 8.496 | 7.14E-04 | 1.277 |
| 94010_g_at | Tceb1 | Transcription elongation factor B (SIII), polypeptide 1 | 7.884 | 9.45E-04 | 1.573 |
| 104476_at | Rbl1 | Retinoblastoma-like 1 (p107) | 7.846 | 9.70E-04 | 1.712 |
| 94239_at | Pnn | Pinin | 7.700 | 1.05E-03 | 1.855 |
| 97315_at | Polr2d | Polymerase (RNA) II (DNA directed) polypeptide D | 7.647 | 1.08E-03 | 1.545 |
| 97364_at | Asf1b | ASF1 anti-silencing function 1 homolog B (S. cerevisiae) | 7.611 | 1.11E-03 | 1.558 |
| 97565_r_at | Tfdp1 | Transcription factor Dp 1 | 7.008 | 1.59E-03 | 1.367 |
| 102868_g_at | Tead4 | TEA domain family member 4 | 6.613 | 1.98E-03 | 1.396 |
| 103204_r_at | E2f8 | E2F transcription factor 8 | 6.538 | 2.08E-03 | 2.270 |
| 94008_at | Tceb1 | Transcription elongation factor B (SIII), polypeptide 1 | 6.388 | 2.26E-03 | 2.765 |
| 93071_at | Trim28 | Tripartite motif protein 28 | 6.332 | 2.35E-03 | 1.297 |
| 160738_at | Polrmt | Polymerase (RNA) mitochondrial (DNA directed) | 6.233 | 2.53E-03 | 1.166 |
| 103964_at | Esrra | Estrogen related receptor, alpha | 6.130 | 2.73E-03 | 1.315 |
| 161418_r_at | Nr5a1 | Nuclear receptor subfamily 5, group A, member 1 | 5.910 | 3.08E-03 | 1.300 |
| 93916_at | Smyd5 | SET and MYND domain containing 5 | 5.906 | 3.09E-03 | 1.544 |
| 99109_at | Ier2 | Immediate early response 2 | 5.894 | 3.12E-03 | 2.191* |
| 98038_at | Hmgb3 | High mobility group box 3 | 5.752 | 3.45E-03 | 1.794 |
| 96625_at | Wdhd1 | WD repeat and HMG-box DNA binding protein 1 | 5.688 | 3.61E-03 | 2.059 |
| 93551_at | Polr2l | Polymerase (RNA) II (DNA directed) polypeptide L | 5.636 | 3.74E-03 | 1.612 |
| 100023_at | Mybl2 | Myeloblastosis oncogene-like 2 | 5.487 | 4.10E-03 | 1.534 |
| 161287_f_at | Mybbp1a | MYB binding protein (P160) 1a | 5.486 | 4.10E-03 | 1.459 |
| 93701_at | Smarca5 | SWI/SNF related, matrix associated, actin dependent regulator of chromatin, subfamily a, member 5 | 5.391 | 4.35E-03 | 1.260 |
| 102069_at | Mtf2 | Metal response element binding transcription factor 2 | 5.269 | 4.77E-03 | 1.203 |
| 103203_f_at | E2f8 | E2F transcription factor 8 | 5.226 | 4.91E-03 | 3.895 |
| 99338_at | Cand1 | Cullin associated and neddylation disassociated 1 | 5.213 | 4.96E-03 | 1.739 |
| 102362_i_at | Junb | Jun-B oncogene | 5.192 | 5.04E-03 | 2.242 |
| 97462_at | Zfp706 | Zinc finger protein 706 | 5.124 | 5.32E-03 | 1.560 |
| 160794_at | Smyd2 | SET and MYND domain containing 2 | 5.122 | 5.33E-03 | 1.508 |
| 99917_at | Ezh2 | Enhancer of zeste homolog 2 (Drosophila) | 5.025 | 5.72E-03 | 1.810 |
| 93250_r_at | Hmgb2 | High mobility group box 2 | 4.924 | 6.19E-03 | 1.415 |
| 101958_f_at | Tfdp1 | Transcription factor Dp 1 | 4.879 | 6.40E-03 | 2.082 |
| 94467_at | Cebpz | CCAAT/enhancer binding protein zeta | 4.791 | 6.84E-03 | 1.260 |
| 95596_at | Foxk2 | Forkhead box K2 | 4.786 | 6.86E-03 | 1.478 |
| 94332_at | Ets1 | E26 avian 85eukaemia oncogene 1, 5’ domain | 4.716 | 7.26E-03 | 2.307 |
| 104610_at | Pprc1 | Peroxisome proliferative activated receptor, gamma, coactivator-related 1 | 4.692 | 7.41E-03 | 1.987 |
| 93944_r_at | Zfp36l2 | Zinc finger protein 36, C3H type-like 2 | 4.642 | 7.67E-03 | 1.498 |
| 97133_at | AA673488 | Expressed sequence AA673488 | 4.623 | 7.80E-03 | 1.106 |
| 92804_at | Polr2h | Polymerase (RNA) II (DNA directed) polypeptide H | 4.612 | 7.87E-03 | 1.304 |
| 101088_f_at | Cnbp1 | Cellular nucleic acid binding protein 1 | 4.522 | 8.48E-03 | 1.582 |
| 101959_r_at | Tfdp1 | Transcription factor Dp 1 | 4.503 | 8.60E-03 | 2.028 |
| 93325_at | Polr2e | Polymerase (RNA) II (DNA directed) polypeptide E | 4.490 | 8.68E-03 | 1.594 |
| 103318_at | Gabpb1 | GA repeat binding protein, beta 1 | 4.486 | 8.70E-03 | 1.274 |
| 102963_at | E2f1 | E2F transcription factor 1 | 4.469 | 8.85E-03 | 1.217 |
| 95755_at | Csda | Cold shock domain protein A | 4.417 | 9.25E-03 | 1.485 |
| 95659_at | Actl6a | Actin-like 6A | 4.364 | 9.70E-03 | 1.651 |
| 94384_at | Ier3 | Immediate early response 3 | 4.354 | 9.82E-03 | 2.685 |
| 102867_at | Tead4 | TEA domain family member 4 | 4.219 | 1.11E-02 | 1.705 |
| 97975_at | Nfatc3 | Nuclear factor of activated T-cells, cytoplasmic, calcineurin-dependent 3 | 4.165 | 1.16E-02 | 1.953 |
| 94030_at | Commd2 | COMM domain containing 2 | 4.161 | 1.16E-02 | 1.368 |
| 98341_f_at | Mybbp1a | MYB binding protein (P160) 1a | 4.130 | 1.19E-02 | 1.481 |
| 99567_at | Zfp162 | Zinc finger protein 162 | 4.070 | 1.25E-02 | 1.174 |
| 160244_at | Fem1a | Feminization 1 homolog a (C. Elegans) | 4.068 | 1.26E-02 | 1.318 |
| 101086_f_at | Cnbp1 | Cellular nucleic acid binding protein 1 | 4.047 | 1.28E-02 | 1.639 |
| 93943_f_at | Zfp36l2 | Zinc finger protein 36, C3H type-like 2 | 4.038 | 1.29E-02 | 2.413 |
| 98343_s_at | Zfy2 | Zinc finger protein 2, Y linked | 4.026 | 1.30E-02 | 1.164 |
| 101527_at | Tcea1 | Transcription elongation factor A (SII) 1 | 4.004 | 1.33E-02 | 1.305 |
| 93935_at | Nlk | Nemo like kinase | 3.972 | 1.37E-02 | 1.506 |
| 94073_at | Polr2g | Polymerase (RNA) II (DNA directed) polypeptide G | 3.953 | 1.39E-02 | 1.911 |
| 99058_at | Hmga2 | High mobility group AT-hook 2 | 3.946 | 1.40E-02 | 3.851 |
| 92568_at | Tfb2m | Transcription factor B2, mitochondrial | 3.936 | 1.42E-02 | 1.501 |
| 98545_at | Phb2 | Prohibitin 2 | 3.920 | 1.44E-02 | 1.937 |
| 103910_at | Taf10 | TAF10 RNA polymerase II, TATA box binding protein (TBP)-associated factor | 3.909 | 1.45E-02 | 1.426 |
| 104583_at | Zdhhc6 | Zinc finger, DHHC domain containing 6 | 3.779 | 1.63E-02 | 1.684* |
| 92225_f_at | Rpo1-2 | RNA polymerase 1-2 | 3.719 | 1.72E-02 | 1.316 |
| 101529_g_at | Tcea1 | Transcription elongation factor A (SII) 1 | 3.770 | 1.65E-02 | 1.398 |
| 92771_at | Zfp207 | Zinc finger protein 207 | 3.736 | 1.69E-02 | 1.733 |
| 103009_at | Hira | Histone cell cycle regulation defective homolog A (S. Cerevisiae) | 3.705 | 1.74E-02 | 1.113 |
| 102354_at | Tcf19 | Transcription factor 19 | 3.672 | 1.79E-02 | 1.457 |
| 103720_at | Rest | RE1-silencing transcription factor | 3.671 | 1.80E-02 | 1.565 |
| 99574_at | Znrf2 | Zinc and ring finger 2 | 3.668 | 1.80E-02 | 1.425 |
| 160901_at | Fos | FBJ osteosarcoma oncogene | 3.650 | 1.83E-02 | 1.447 |
| 103868_at | Nufip1 | Nuclear fragile X mental retardation protein interacting protein | 3.622 | 1.87E-02 | 1.296 |
| 94466_f_at | Cebpz | CCAAT/enhancer binding protein zeta | 3.614 | 1.89E-02 | 1.734 |
| 162384_f_at | Ccrn4l | CCR4 carbon catabolite repression 4-like (S. cerevisiae) | 3,609 | 1,90E-02 | 1,632* |
| 99034_at | Irx3 | Iroquois related homeobox 3 (drosophila) | -11.410 | 2.06E-04 | 0.720 |
| 98790_s_at | Meis1 | Myeloid ecotropic viral integration site 1 | -11.086 | 2.27E-04 | 0.537 |
| 100947_at | Tcf20 | Transcription factor 20 | -10.516 | 2.68E-04 | 0.712 |
| 103504_at | Ssbp2 | Single-stranded DNA binding protein 2 | -8,815 | 6,09E-04 | 0,231 |
| 100475_at | Trim25 | Tripartite motif protein 25 | -7.858 | 9.63E-04 | 0.199 |
| 92935_at | Runx1t1 | Runt-related transcription factor 1; translocated to, 1 (cyclin D-related) | -7.696 | 1.06E-03 | 0.301 |
| 95161_at | Ctdsp2 | CTD (carboxy-terminal domain, RNA polymerase II, polypeptide A) small phosphatase 2 | -7,408 | 1,23E-03 | 0,303 |
| 100522_s_at | Wbp5 | WW domain binding protein 5 | -7.100 | 1.51E-03 | 0.666 |
| 95059_at | Pnrc2 | Proline-rich nuclear receptor coactivator 2 | -6.471 | 2.16E-03 | 0.400* |
| 99552_at | Snai2 | Snail homolog 2 (Drosophila) | -6.429 | 2.20E-03 | 0.480 |
| 99077_at | Thra | Thyroid hormone receptor alpha | -5.900 | 3.11E-03 | 0.724 |
| 161080_f_at | Gtf2ird2 | GTF2I repeat domain containing 2 | -5.765 | 3.42E-03 | 0.550 |
| 162372_f_at | Relb | Avian reticuloendotheliosis viral (v-rel) oncogene related B | -5.712 | 3.55E-03 | 0.678 |
| 160780_at | Tcf3 | Transcription factor 3 | -5.460 | 4.17E-03 | 0.547 |
| 160245_at | Zfp740 | Zinc finger protein 740 | -5.406 | 4.29E-03 | 0.575 |
| 162118_f_at | Btbd14a | BTB (POZ) domain containing 14A | -5.364 | 4.41E-03 | 0.874 |
| 102882_at | Zfp46 | Zinc finger protein 46 | -5.342 | 4.48E-03 | 0.852 |
| 101429_at | Ddit3 | DNA-damage inducible transcript 3 | -5.314 | 4.60E-03 | 0.274 |
| 104731_at | Zfp467 | Zinc finger protein 467 | -5.177 | 5.10E-03 | 0.627 |
| 103466_at | Cyhr1 | Cysteine and histidine rich 1 | -5.080 | 5.52E-03 | 0.636 |
| 94295_at | Gtf2i | General transcription factor II I | -5.010 | 5.80E-03 | 0.649 |
| 92251_f_at | AI607873 | Expressed sequence AI607873 | -4.950 | 6.09E-03 | 0.768 |
| 104010_at | Zfp99 | Zinc finger protein 99 | -4.672 | 7.51E-03 | 0.744 |
| 98122_at | Lmo4 | LIM domain only 4 | -4.644 | 7.66E-03 | 0.414* |
| 104209_at | Cyhr1 | Cysteine and histidine rich 1 | -4.638 | 7.71E-03 | 0.822 |
| 93621_at | Tgif2 | TGFB-induced factor 2 | -4.599 | 7.94E-03 | 0.807 |
| 160138_at | Mxi1 | Max interacting protein 1 | -4.522 | 8.48E-03 | 0.432 |
| 95033_at | Jmjd1a | Jumonji domain containing 1A | -4.488 | 8.69E-03 | 0.552 |
| 103450_at | Bcas3 | Breast carcinoma amplified sequence 3 | -4.472 | 8.81E-03 | 0.845 |
| 104263_at | Zfp258 | Zinc finger protein 258 | -4.454 | 8.95E-03 | 0.410 |
| 93442_at | Zfp316 | Zinc finger protein 316 | -4.440 | 9.06E-03 | 0.624 |
| 95522_i_at | Zfp68 | Zinc finger protein 68 | -4.431 | 9.12E-03 | 0.497* |
| 102048_at | Ankrd1 | Ankyrin repeat domain 1 (cardiac muscle) | -4.424 | 9.20E-03 | 0.134* |
| 103006_at | Atf5 | Activating transcription factor 5 | -4.406 | 9.32E-03 | 0.413 |
| 104458_at | Tcf3 | Transcription factor 3 | -4.387 | 9.48E-03 | 0.763 |
| 160793_at | Pou6f1 | POU domain, class 6, transcription factor 1 | -4.363 | 9.72E-03 | 0.393 |
| 100599_at | Atf4 | Activating transcription factor 4 | -4.279 | 1.05E-02 | 0.663 |
| 103011_at | Sin3a | Transcriptional regulator, SIN3A (yeast) | -4.274 | 1.05E-02 | 0.867 |
| 102256_at | Tbx15 | T-box 15 | -4.259 | 1.07E-02 | 0.306 |
| 160117_at | Tef | Thyrotroph embryonic factor | -4.206 | 1.12E-02 | 0.738 |
| *Primary cell metabolism* | | |  |  |  |
| 99148_at | Fh1 | Fumarate hydratase 1 | 31.594 | 2.29E-06 | 1.961 |
| 97539_at | Hrmt1l3 | Heterogeneous nuclear ribonucleoprotein methyltransferase-like 3 (S. Cerevisiae) | 15.299 | 5.72E-05 | 1.523 |
| 98372_at | Aldh1a3 | Aldehyde dehydrogenase family 1, subfamily A3 | 12.235 | 1.35E-04 | 5.204* |
| 160767_at | Soat1 | Sterol O-acyltransferase 1 | 11.207 | 2.20E-04 | 3.575 |
| 94237_at | Rpn1 | Ribophorin I | 10.621 | 2.56E-04 | 1.880 |
| 160648_at | Fignl1 | Fidgetin-like 1 | 9.193 | 5.03E-04 | 2.963 |
| 104547_at | Dhfr | Dihydrofolate reductase | 9.085 | 5.31E-04 | 1.522 |
| 160107_at | Hprt1 | Hypoxanthine guanine phosphoribosyl transferase 1 | 7.936 | 9.27E-04 | 2.770 |
| 161038_at | Prps2 | Phosphoribosyl pyrophosphate synthetase 2 | 7.919 | 9.31E-04 | 1.352 |
| 96827_at | Cad | Carbamoyl-phosphate synthetase 2, aspartate transcarbamylase, and dihydroorotase | 7.704 | 1.05E-03 | 1.698 |
| 101466_at | Pigf | Phosphatidylinositol glycan, class F | 7.504 | 1.16E-03 | 1.403 |
| 95887_at | Soat1 | Sterol O-acyltransferase 1 | 7.338 | 1.29E-03 | 3.075 |
| 98071_f_at | Dck | Deoxycytidine kinase | 7.218 | 1.40E-03 | 1.981 |
| 104538_at | Ptgis | Prostaglandin I2 (prostacyclin) synthase | 6.840 | 1.73E-03 | 2.956 |
| 98459_at | Shmt1 | Serine hydroxymethyl transferase 1 (soluble) | 6.647 | 1.93E-03 | 2.112* |
| 103683_at | Dhodh | Dihydroorotate dehydrogenase | 6.601 | 2.00E-03 | 1.730 |
| 98922_at | Stt3a | STT3, subunit of the oligosaccharyltransferase complex, homolog A (S. cerevisiae) | 6.500 | 2.12E-03 | 1.370 |
| 100323_at | Amd2 | S-adenosylmethionine decarboxylase 2 | 5.696 | 3.59E-03 | 2.657 |
| 160084_at | Odc1 | Ornithine decarboxylase, structural 1 | 6.098 | 2.77E-03 | 2.600 |
| 96616_at | Atad1 | ATPase family, AAA domain containing 1 | 6.030 | 2.86E-03 | 1.694 |
| 160492_at | Ddx18 | DEAD (Asp-Glu-Ala-Asp) box polypeptide 18 | 5.946 | 3.03E-03 | 1.774 |
| 160289_s_at | Dlst | Dihydrolipoamide S-succinyltransferase (E2 component of 2-oxo-glutarate complex) | 5.914 | 3.07E-03 | 1.643 |
| 95507_at | Prps1 | Phosphoribosyl pyrophosphate synthetase 1 | 5.832 | 3.29E-03 | 2.324 |
| 98619_at | Dtymk | Deoxythymidylate kinase | 5.805 | 3.35E-03 | 1.485 |
| 160385_at | Gcsh | Glycine cleavage system protein H (aminomethyl carrier) | 5.442 | 4.21E-03 | 1.994 |
| 160166_r_at | Ola1 | Obg-like ATPase 1 | 5.336 | 4.50E-03 | 1.534 |
| 101568_at | Prosc | Proline synthetase co-transcribed | 5.257 | 4.82E-03 | 1.593 |
| 93991_at | Mdh2 | Malate dehydrogenase 2, NAD (mitochondrial) | 5.226 | 4.91E-03 | 1.507 |
| 103924_at | Agpat5 | 1-acylglycerol-3-phosphate O-acyltransferase 5 (lysophosphatidic acid acyltransferase, epsilon) | 5.222 | 4.93E-03 | 1.434 |
| 161103_at | Entpd7 | Ectonucleoside triphosphate diphosphohydrolase 7 | 5.215 | 4.96E-03 | 1.622 |
| 104282_at | AW112037 | Expressed sequence AW112037 | 5.212 | 4.97E-03 | 1.213 |
| 100912_at | Dph5 | DPH5 homolog (S. Cerevisiae) | 5.184 | 5.06E-03 | 1.926 |
| 92335_at | Ltbp2 | Latent transforming growth factor beta binding protein 2 | 5.162 | 5.15E-03 | 3.425 |
| 103560_at | Lysmd2 | LysM, putative peptidoglycan-binding, domain containing 2 | 5.111 | 5.39E-03 | 1.698 |
| 94367_at | Uck2 | Uridine-cytidine kinase 2 | 5.095 | 5.47E-03 | 2.738 |
| 101489_at | Amd1 | S-adenosylmethionine decarboxylase 1 | 5.014 | 5.79E-03 | 2.600 |
| 104017_at | Acsl4 | Acyl-coa synthetase long-chain family member 4 | 4.855 | 6.52E-03 | 3.323 |
| 101002_at | Azin1 | Antizyme inhibitor 1 | 4.834 | 6.63E-03 | 2.112 |
| 103442_at | Dhrs7b | Dehydrogenase/reductase (SDR family) member 7B | 4.811 | 6.76E-03 | 1.674 |
| 98544_at | Guk1 | Guanylate kinase 1 | 4.646 | 7.64E-03 | 2.187 |
| 160067_at | Enoph1 | Enolase-phosphatase 1 | 4.641 | 7.68E-03 | 1.312 |
| 161092_at | Umps | Uridine monophosphate synthetase | 4.595 | 7.98E-03 | 1.606 |
| 96139_at | Gcs1 | Glucosidase 1 | 4.593 | 7.99E-03 | 1.362 |
| 100066_at | Gart | Phosphoribosylglycinamide formyltransferase | 4.578 | 8.09E-03 | 1.957 |
| 100026_at | Bcat1 | Branched chain aminotransferase 1, cytosolic | 4.540 | 8.35E-03 | 2.072* |
| 104140_s_at | Nomo1 | Nodal modulator 1 | 4.430 | 9.14E-03 | 1.288 |
| 97880_at | Dlst | Dihydrolipoamide S-succinyltransferase (E2 component of 2-oxo-glutarate complex) | 4.426 | 9.18E-03 | 1.540 |
| 99666_at | Cs | Citrate synthase | 4.375 | 9.60E-03 | 1.516 |
| 92304_at | Piga | Phosphatidylinositol glycan, class A | 4.356 | 9.80E-03 | 1.772 |
| 92553_at | Esd | Esterase D/formylglutathione hydrolase | 4.280 | 1.05E-02 | 1.704 |
| 160191_at | Nat11 | N-acetyltransferase 11 | 4.227 | 1.10E-02 | 1.549 |
| 161161_r_at | Nme1 | Expressed in non-metastatic cells 1, protein | 4.180 | 1.15E-02 | 1.232 |
| 98999_at | Adsl | Adenylosuccinate lyase | 4.064 | 1.26E-02 | 1.769 |
| 160314_at | Ppa1 | Pyrophosphatase (inorganic) 1 | 4.001 | 1.34E-02 | 2.318 |
| 98593_at | Cmas | Cytidine monophospho-N-acetylneuraminic acid synthetase | 3.925 | 1.43E-02 | 1.245 |
| 95468_at | Egln1 | EGL nine homolog 1 (C. elegans) | 3.904 | 1.46E-02 | 1.466 |
| 161897_f_at | Prps1 | Phosphoribosyl pyrophosphate synthetase 1 | 3.846 | 1.54E-02 | 1.305 |
| 100073_at | N6amt2 | N-6 adenine-specific DNA methyltransferase 2 (putative) | 3.833 | 1.55E-02 | 1.561 |
| 162041_f_at | Isyna1 | Myo-inositol 1-phosphate synthase A1 | 3.810 | 1.59E-02 | 1.294 |
| 96925_at | Glt25d1 | Glycosyltransferase 25 domain containing 1 | 3.785 | 1.62E-02 | 1.307 |
| 104534_at | Pgm1 | Phosphoglucomutase 1 | 3.632 | 1.86E-02 | 1.453 |
| 96025_g_at | Ahcy | S-adenosylhomocysteine hydrolase | 3.626 | 1.87E-02 | 1.732 |
| 98618_at | Dtymk | Deoxythymidylate kinase | 3.621 | 1.87E-02 | 1.549 |
| 99610_at | Ss18 | Synovial sarcoma translocation, Chromosome 18 | 3.604 | 1.91E-02 | 1.953* |
| 94644_at | Bche | Butyrylcholinesterase | 3.590 | 1.94E-02 | 1.159 |
| 97924_at | Gne | Glucosamine | 3.589 | 1.94E-02 | 1.248 |
| 102026_s_at | Chkb | Choline kinase beta | -12.231 | 1.37E-04 | 0.661 |
| 98527_at | Dci | Dodecenoyl-Coenzyme A delta isomerase (3,2 trans-enoyl-Coenyme A isomerase) | -9.311 | 4.69E-04 | 0.673 |
| 101473_at | Nnmt | Nicotinamide N-methyltransferase | -9.076 | 5.33E-04 | 0.447 |
| 102094_f_at | Gstm1 | Glutathione S-transferase, mu 1 | -9.075 | 5.35E-04 | 0.351 |
| 93543_f_at | Gstm1 | Glutathione S-transferase, mu 1 | -8.367 | 7.48E-04 | 0.358 |
| 92557_at | Hsd17b1 | Hydroxysteroid (17-beta) dehydrogenase 1 | -7.831 | 9.84E-04 | 0.802 |
| 101000_at | Oaz2 | Ornithine decarboxylase antizyme 2 | -7.446 | 1.20E-03 | 0.351 |
| 160652_at | Ctps2 | Cytidine 5’-triphosphate synthase 2 | -6.625 | 1.96E-03 | 0.544 |
| 93940_at | Pon3 | Paraoxonase 3 | -6.396 | 2.25E-03 | 0.634 |
| 99055_at | St6galnac2 | ST6 (alpha-N-acetyl-neuraminyl-2,3-beta-galactosyl-1,3)-N-acetylgalactosaminide alpha-2,6-sialyltransferase 2 | -6.305 | 2.39E-03 | 0.837 |
| 103066_at | Tyki | Thymidylate kinase family LPS-inducible member | -6.275 | 2.44E-03 | 0.748 |
| 102027_s_at | Chkb | Choline kinase beta | -6.192 | 2.61E-03 | 0.642 |
| 95569_at | Guca2b | Guanylate cyclase activator 2b (retina) | -5.872 | 3.18E-03 | 0.842 |
| 103739_at | Glce | Glucuronyl C5-epimerase | -5.715 | 3.55E-03 | 0.526 |
| 98588_at | Fah | Fumarylacetoacetate hydrolase | -5.689 | 3.61E-03 | 0.498 |
| 99894_at | Ptgfrn | Prostaglandin F2 receptor negative regulator | -5.688 | 3.62E-03 | 0.761 |
| 103637_at | Naga | N-acetyl galactosaminidase, alpha | -5.673 | 3.64E-03 | 0.465 |
| L09192_3_at | Pcx | Pyruvate carboxylase | -5.239 | 4.87E-03 | 0.461 |
| 160770_at | Mvd | Mevalonate (diphospho) decarboxylase | -5.236 | 4.88E-03 | 0.340 |
| 102004_at | Acaa1 | Acetyl-Coenzyme A acyltransferase 1 | -5.222 | 4.94E-03 | 0.797 |
| 103914_at | Pcyt2 | Phosphate cytidylyltransferase 2, ethanolamine | -5.057 | 5.60E-03 | 0.473 |
| 99184_at | Csad | Cysteine sulfinic acid decarboxylase | -4.986 | 5.92E-03 | 0.543* |
| 96608_at | Phyh | Phytanoyl-CoA hydroxylase | -4.980 | 5.95E-03 | 0.373* |
| 162401_f_at | Isyna1 | Myo-inositol 1-phosphate synthase A1 | -4.936 | 6.14E-03 | 0.712 |
| 101082_at | Me1 | Malic enzyme 1, NADP(+)-dependent, cytosolic | -4.866 | 6.46E-03 | 0.492 |
| 97971_at | Phkg2 | Phosphorylase kinase, gamma 2 (testis) | -4.851 | 6.54E-03 | 0.813 |
| 101515_at | Acox1 | Acyl-Coenzyme A oxidase 1, palmitoyl | -4.796 | 6.80E-03 | 0.696 |
| 93868_at | Nsdhl | NAD(P) dependent steroid dehydrogenase-like | -4.733 | 7.16E-03 | 0.433 |
| 94438_at | Pfkm | Phosphofructokinase, muscle | -4.685 | 7.44E-03 | 0.618 |
| 102783_at | 2310009E04Rik | RIKEN cDNA 2310009E04 gene | -4.637 | 7.72E-03 | 0.816 |
| 95019_at | Gstt1 | Glutathione S-transferase, theta 1 | -4.629 | 7.76E-03 | 0.415 |
| 100576_at | Pafah1b3 | Platelet-activating factor acetylhydrolase, isoform 1b, alpha1 subunit | -4.555 | 8.22E-03 | 0.573 |
| 99160_s_at | Grina | Glutamate receptor, ionotropic, N-methyl D-asparate-associated protein 1 (glutamate binding) | -4.522 | 8.48E-03 | 0.454 |
| 160883_at | D2hgdh | D-2-hydroxyglutarate dehydrogenase | -4.519 | 8.50E-03 | 0.780 |
| 100042_at | Hagh | Hydroxyacyl glutathione hydrolase | -4.494 | 8.66E-03 | 0.665 |
| 104677_at | Man1b1 | Mannosidase, alpha, class 1B, member 1 | -4.484 | 8.72E-03 | 0.630 |
| 160563_at | Serf2 | Small EDRK-rich factor 2 | -4.466 | 8.87E-03 | 0.777 |
| 93308_s_at | Pcx | Pyruvate carboxylase | -4.431 | 9.12E-03 | 0.501 |
| 96789_i_at | Galm | Galactose mutarotase | -4.407 | 9.32E-03 | 0.824 |
| 100068_at | Aldh1a1 | Aldehyde dehydrogenase family 1, subfamily A1 | -4.308 | 1.02E-02 | 0.765 |
| 104605_at | Adipor2 | Adiponectin receptor 2 | -4.308 | 1.02E-02 | 0.713 |
| 97518_at | Fdft1 | Farnesyl diphosphate farnesyl transferase 1 | -4.261 | 1.07E-02 | 0.355* |
| *Transport and trafficking processes* | | |  |  |  |
| 99329_at | Abcc1 | ATP-binding cassette, sub-family C (CFTR/MRP), member 1 | 10.565 | 2.61E-04 | 2.506 |
| 160409_at | Pitpna | Phosphatidylinositol transfer protein, alpha | 9.969 | 3.18E-04 | 1.497 |
| 104145_at | Tcof1 | Treacher Collins Franceschetti syndrome 1, homolog | 8.804 | 6.13E-04 | 1.931 |
| 101877_at | Slc31a1 | Solute carrier family 31, member 1 | 7.950 | 9.22E-04 | 1.243 |
| 98975_at | Nup93 | Nucleoporin 93 | 7.770 | 1.02E-03 | 1.771 |
| 103218_at | Slc10a3 | Solute carrier family 10 (sodium/bile acid cotransporter family), member 3 | 6.664 | 1.91E-03 | 1.773 |
| 95075_at | Nup35 | Nucleoporin 35 | 6.435 | 2.20E-03 | 1.802 |
| 98457_at | Slc4a4 | Solute carrier family 4 (anion exchanger), member 4 | 6.396 | 2.24E-03 | 1.843 |
| 96007_at | Ssr3 | Signal sequence receptor, gamma | 6.298 | 2.40E-03 | 1.590 |
| 100568_at | Abce1 | ATP-binding cassette, sub-family E (OABP), member 1 | 6.220 | 2.55E-03 | 1.893 |
| 102412_at | Nup107 | Nucleoporin 107 | 6.140 | 2.71E-03 | 1.644 |
| 93336_at | Tmed10 | Transmembrane emp24-like trafficking protein 10 (yeast) | 6.038 | 2.85E-03 | 1.474 |
| 100417_at | Slc7a6 | Solute carrier family 7 (cationic amino acid transporter, y+ system), member 6 | 5.946 | 3.03E-03 | 1.467 |
| 160936_at | Tram1 | Translocating chain-associating membrane protein 1 | 5.922 | 3.06E-03 | 1.831 |
| 101370_at | Kpna1 | Karyopherin (importin) alpha 1 | 5.898 | 3.11E-03 | 2.094 |
| 93626_at | Abcg2 | ATP-binding cassette, sub-family G (WHITE), member 2 | 5.888 | 3.14E-03 | 1.660 |
| 99146_at | Stx6 | Syntaxin 6 | 5.743 | 3.47E-03 | 1.834 |
| 92790_at | Kpna2 | Karyopherin (importin) alpha 2 | 5.694 | 3.59E-03 | 3.011 |
| 97551_at | Hip1r | Huntingtin interacting protein 1 related | 5.583 | 3.87E-03 | 1.458* |
| 104510_at | Cacna2d1 | Calcium channel, voltage-dependent, alpha2/delta subunit 1 | 5.539 | 3.98E-03 | 1.214 |
| 95432_f_at | Tomm70a | Translocase of outer mitochondrial membrane 70 homolog A (yeast) | 5.533 | 4.00E-03 | 2.220 |
| 101295_s_at | Clns1a | Chloride channel, nucleotide-sensitive, 1A | 5.509 | 4.05E-03 | 1.379 |
| 92288_at | Ap1g1 | Adaptor protein complex AP-1, gamma 1 subunit | 5.093 | 5.48E-03 | 1.379 |
| 93330_at | Aqp1 | Aquaporin 1 | 5.423 | 4.25E-03 | 2.433 |
| 95708_at | D3Ucla1 | DNA segment, Chr 3, University of California at Los Angeles 1 | 5.400 | 4.31E-03 | 1.782 |
| 93111_at | Kpnb1 | Karyopherin (importin) beta 1 | 5.384 | 4.37E-03 | 1.612 |
| 161355_f_at | Slc35e1 | Solute carrier family 35, member E1 | 5.372 | 4.40E-03 | 1.148 |
| 92450_at | Slc12a4 | Solute carrier family 12, member 4 | 5.274 | 4.75E-03 | 1.364 |
| 101036_at | Tomm20 | Translocase of outer mitochondrial membrane 20 homolog (yeast) | 5.191 | 5.04E-03 | 1.277 |
| 160167_at | Nup62 | Nucleoporin 62 | 5.136 | 5.28E-03 | 1.371 |
| 94419_at | Slc19a1 | Solute carrier family 19 (sodium/hydrogen exchanger), member 1 | 4.834 | 6.63E-03 | 1.639 |
| 96010_at | Kpna3 | Karyopherin (importin) alpha 3 | 4.761 | 6.99E-03 | 1.768 |
| 96760_at | Timm9 | Translocase of inner mitochondrial membrane 9 homolog (yeast) | 4.705 | 7.33E-03 | 1.481 |
| 96762_at | Tnpo1 | Transportin 1 | 4.578 | 8.10E-03 | 1.570 |
| 104565_at | Ap4s1 | Adaptor-related protein complex AP-4, sigma 1 | 4.552 | 8.25E-03 | 1.188 |
| 160543_at | Snx3 | Sorting nexin 3 | 4.537 | 8.37E-03 | 1.449* |
| 104475_at | Slc35e1 | Solute carrier family 35, member E1 | 4.420 | 9.23E-03 | 1.703 |
| 100964_at | Vti1b | Vesicle transport through interaction with t-snares 1B homolog | 4.407 | 9.32E-03 | 1.536 |
| 104007_at | Slc25a15 | Solute carrier family 25 (mitochondrial carrier ornithine transporter), member 15 | 4.395 | 9.41E-03 | 1.299 |
| 93471_at | Slc4a7 | Solute carrier family 4, sodium bicarbonate cotransporter, member 7 | 4.350 | 9.85E-03 | 2.450 |
| 102342_at | Nsf | N-ethylmaleimide sensitive fusion protein | 4.245 | 1.08E-02 | 1.479 |
| 93414_at | Abcb1b | ATP-binding cassette, sub-family B (MDR/TAP), member 1B | 4.180 | 1.15E-02 | 2.242 |
| 94870_f_at | Sar1b | SAR1a gene homolog B (S. cerevisiae) | 4.158 | 1.17E-02 | 1.502 |
| 95978_at | Atp13a3 | ATPase type 13A3 | 4.157 | 1.17E-02 | 1.486 |
| 160739_at | Wnk1 | WNK lysine deficient protein kinase 1 | 4.043 | 1.29E-02 | 2.401 |
| 92398_at | Vps37b | Vacuolar protein sorting 37B (yeast) | 3.986 | 1.35E-02 | 1.151 |
| 100617_at | Slc25a5 | Solute carrier family 25 (mitochondrial carrier, adenine nucleotide translocator), member 5 | 3.947 | 1.40E-02 | 1.366 |
| 94228_at | Xpo1 | Exportin 1, CRM1 homolog (yeast) | 3.836 | 1.55E-02 | 1.580 |
| 98956_at | Tram1 | Translocating chain-associating membrane protein 1 | 3.767 | 1.65E-02 | 2.168 |
| 160291_at | Sec61a1 | Sec61 alpha 1 subunit (S. cerevisiae) | 3.739 | 1.69E-02 | 1.664 |
| 99579_at | Atp1b3 | ATPase, Na+/K+ transporting, beta 3 polypeptide | 3.736 | 1.69E-02 | 1.578 |
| 160653_at | Tomm40 | Translocase of outer mitochondrial membrane 40 homolog (yeast) | 3.731 | 1.70E-02 | 1.408 |
| 97526_at | Ap3m1 | Adaptor-related protein complex 3, mu 1 subunit | 3.646 | 1.84E-02 | 1.388 |
| 99188_at | Use1 | Unconventional SNARE in the ER 1 homolog (S. cerevisiae) | -10,449 | 2,72E-04 | 0,487 |
| 93304_at | Slc3a1 | Solute carrier family 3, member 1 | -10.264 | 2.95E-04 | 0.875 |
| 162479_f_at | Ftl1 | Ferritin light chain 1 | -9.000 | 5.63E-04 | 0.600 |
| 97243_at | Slc9a3r1 | Solute carrier family 9 (sodium/hydrogen exchanger), isoform 3 regulator 1 | -7.785 | 1.01E-03 | 0.582* |
| 93045_at | Abcd3 | ATP-binding cassette, sub-family D (ALD), member 3 | -6.493 | 2.12E-03 | 0.452 |
| 100596_at | Selenbp1 | Selenium binding protein 1 | -6.256 | 2.47E-03 | 0.843 |
| 162039_f_at | Scamp2 | Secretory carrier membrane protein 2 | -6.172 | 2.64E-03 | 0.855 |
| 99872_s_at | Ftl1 | Ferritin light chain 1 | -5.871 | 3.19E-03 | 0.555 |
| 98108_at | Crabp1 | Cellular retinoic acid binding protein I | -5.479 | 4.12E-03 | 0.091 |
| 162486_f_at | Dscr3 | Down syndrome critical region gene 3 | -5.446 | 4.19E-03 | 0.715 |
| 98483_at | Cacnb3 | Calcium channel, voltage-dependent, beta 3 subunit | -5.280 | 4.72E-03 | 0.670 |
| 160076_at | Mtx2 | Metaxin 2 | -5.258 | 4.81E-03 | 0.751 |
| 101441_i_at | Itpr2 | Inositol 1,4,5-triphosphate receptor 2 | -5.157 | 5.18E-03 | 0.489 |
| 98950_at | Rragc | Ras-related GTP binding C | -5.069 | 5.56E-03 | 0.693 |
| 103031_g_at | Dnm1 | Dynamin 1 | -5.009 | 5.81E-03 | 0.350 |
| 94244_at | Ihpk1 | Inositol hexaphosphate kinase 1 | -4.818 | 6.72E-03 | 0.602 |
| 96160_at | Slc6a9 | Solute carrier family 6 (neurotransmitter transporter, glycine), member 9 | -4.628 | 7.77E-03 | 0.709 |
| 100943_at | Slc1a4 | Solute carrier family 1 (glutamate/neutral amino acid transporter), member 4 | -4.612 | 7.86E-03 | 0.592 |
| 99139_at | Vps26b | Vacuolar protein sorting 26 homolog B (yeast) | -4.588 | 8.02E-03 | 0.776 |
| 100571_at | Laptm4b | Lysosomal-associated protein transmembrane 4B | -4.539 | 8.36E-03 | 0.486 |
| 103030_at | Dnm1 | Dynamin 1 | -4.536 | 8.37E-03 | 0.449 |
| 96832_at | Slc39a1 | Solute carrier family 39 (zinc transporter), member 1 | -4.471 | 8.82E-03 | 0.731 |
| 93736_at | Tcn2 | Transcobalamin 2 | -4.366 | 9.68E-03 | 0.559 |
| 93440_at | Sec22b | SEC22 vesicle trafficking protein homolog B (S. cerevisiae) | -4.327 | 1.00E-02 | 0.807 |
| 96894_at | Tmed4 | Transmembrane emp24 protein transport domain containing 4 | -4.279 | 1.05E-02 | 0.747 |
| 104143_at | Copz2 | Coatomer protein complex, subunit zeta 2 | -4.247 | 1.08E-02 | 0.678 |
| *Cell cycle and DNA replication* | | |  |  |  |
| 103069_at | Lin9 | Lin-9 homolog (C. Elegans) | 17.311 | 3.89E-05 | 1.651 |
| 103821_at | Cdc6 | Cell division cycle 6 homolog (S. Cerevisiae) | 13.481 | 8.69E-05 | 5.952 |
| 97504_at | Ccnd2 | Cyclin D2 | 13.375 | 9.15E-05 | 2.854# |
| 93356_at | Mcm7 | Minichromosome maintenance deficient 7 (S. Cerevisiae) | 9.285 | 4.78E-04 | 1.980 |
| 97327_at | Fen1 | Flap structure specific endonuclease 1 | 8.766 | 6.29E-04 | 3.782 |
| 104690_at | Polm | Polymerase (DNA directed), mu | 8.040 | 8.74E-04 | 1.475 |
| 97182_at | Ccne2 | Cyclin E2 | 7.801 | 1.00E-03 | 1.545 |
| 100156_at | Mcm5 | Minichromosome maintenance deficient 5, cell division cycle 46 (S. Cerevisiae) | 7.551 | 1.13E-03 | 3.206* |
| 160127_at | Ccng1 | Cyclin G1 | 7.513 | 1.15E-03 | 5.743# |
| 101521_at | Birc5 | Baculoviral IAP repeat-containing 5 | 7.496 | 1.16E-03 | 2.367 |
| 160496_s_at | Mcm3 | Minichromosome maintenance deficient 3 (S. Cerevisiae) | 7.367 | 1.27E-03 | 3.409 |
| 96784_at | Anln | Anillin, actin binding protein (scraps homolog, Drosophila) | 7.289 | 1.33E-03 | 2.586 |
| 103057_at | Pold1 | Polymerase (DNA directed), delta 1, catalytic subunit | 7.096 | 1.51E-03 | 1.888 |
| 93112_at | Mcm2 | Minichromosome maintenance deficient 2 mitotin (S. Cerevisiae) | 7.016 | 1.59E-03 | 2.492 |
| 98006_at | Pola2 | Polymerase (DNA directed), alpha 2 | 6.759 | 1.80E-03 | 2.310 |
| 100062_at | Mcm3 | Minichromosome maintenance deficient 3 (S. Cerevisiae) | 6.610 | 1.98E-03 | 4.574 |
| 95612_at | Rfc5 | Replication factor C (activator 1) 5 | 6.555 | 2.05E-03 | 1.610 |
| 160885_at | Nucks1 | Nuclear casein kinase and cyclin-dependent kinase substrate 1 | 6.536 | 2.08E-03 | 2.444 |
| 103034_at | Ccne1 | Cyclin E1 | 6.358 | 2.31E-03 | 1.368 |
| 95527_at | Chaf1a | Chromatin assembly factor 1, subunit A (p150) | 6.140 | 2.71E-03 | 1.422 |
| 103212_at | Cdca7l | Cell division cycle associated 7 like | 6.028 | 2.87E-03 | 2.005 |
| 97896_r_at | Hat1 | Histone aminotransferase 1 | 5.974 | 2.97E-03 | 2.275 |
| 95063_at | Cdca7 | Cell division cycle associated 7 | 5.916 | 3.07E-03 | 2.230 |
| 101920_at | Pole2 | Polymerase (DNA directed), epsilon 2 (p59 subunit) | 5.739 | 3.48E-03 | 1.910 |
| 103071_at | Topbp1 | Topoisomerase (DNA) II beta binding protein | 5.725 | 3.51E-03 | 1.747 |
| 104423_at | Dtl | Denticleless homolog (Drosophila) | 5.642 | 3.71E-03 | 2.149 |
| 92458_at | Orc1l | Origin recognition complex, subunit 1-like (S.cereviaiae) | 5.505 | 4.07E-03 | 1.480 |
| 103444_at | Dna2l | DNA2 DNA replication helicase 2-like (yeast) | 5.408 | 4.29E-03 | 1.487 |
| 94024_at | Ris2 | Retroviral integration site 2 | 5.356 | 4.43E-03 | 2.048 |
| 99186_at | Ccna2 | Cyclin A2 | 5.272 | 4.76E-03 | 1.494 |
| 97895_f_at | Hat1 | Histone aminotransferase 1 | 5.218 | 4.95E-03 | 1.818 |
| 96833_at | Nucks1 | Nuclear casein kinase and cyclin-dependent kinase substrate 1 | 5.157 | 5.17E-03 | 1.987 |
| 95712_at | Orc6l | Origin recognition complex, subunit 6-like (S. Cerevisiae) | 5.131 | 5.30E-03 | 1.918 |
| 103553_at | Mcm10 | Minichromosome maintenance deficient 10 (S. Cerevisiae) | 5.000 | 5.86E-03 | 1.578 |
| 96319_at | Cdc20 | Cell division cycle 20 homolog (S. Cerevisiae) | 4.993 | 5.90E-03 | 1.638 |
| 97891_at | Sac3d1 | SAC3 domain containing 1 | 4.957 | 6.06E-03 | 2.123 |
| 103270_at | Gtse1 | G two S phase expressed protein 1 | 4.851 | 6.54E-03 | 1.970 |
| 100349_at | Rrm2 | Ribonucleotide reductase M2 | 4.840 | 6.60E-03 | 1.189 |
| 103428_at | Pold3 | Polymerase (DNA-directed), delta 3, accessory subunit | 4.793 | 6.82E-03 | 1.302 |
| 102403_at | Cdc45l | Cell division cycle 45 homolog (S. Cerevisiae)-like | 4.783 | 6.88E-03 | 1.480* |
| 93099_f_at | Plk1 | Polo-like kinase 1 (Drosophila) | 4.764 | 6.96E-03 | 1.505 |
| 95427_at | Rpa1 | Replication protein A1 | 4.762 | 6.98E-03 | 2.273 |
| 95032_at | Prc1 | Protein regulator of cytokinesis 1 | 4.710 | 7.30E-03 | 1.664 |
| 102827_at | Nek7 | NIMA (never in mitosis gene a)-related expressed kinase 7 | 4.690 | 7.42E-03 | 1.894 |
| 92879_at | Ppm1g | Protein phosphatase 1G (formerly 2C), magnesium-dependent, gamma isoform | 4.686 | 7.44E-03 | 1.655 |
| 96200_at | Cdca4 | Cell division cycle associated 4 | 4.637 | 7.72E-03 | 1.616 |
| 95497_at | Tipin | Timeless interacting protein | 4.625 | 7.79E-03 | 1.654 |
| 93758_at | Incenp | Inner centromere protein | 4.588 | 8.01E-03 | 2.024 |
| 100128_at | Cdc2a | Cell division cycle 2 homolog A (S. Pombe) | 4.480 | 8.74E-03 | 1.743 |
| 94232_at | Ccnd1 | Cyclin D1 | 4.384 | 9.51E-03 | 3.425 |
| 93041_at | Mcm4 | Minichromosome maintenance deficient 4 homolog (S. Cerevisiae) | 4.369 | 9.65E-03 | 2.171 |
| 96178_at | Myst2 | MYST histone acetyltransferase 2 | 4.312 | 1.02E-02 | 1.235 |
| 99578_at | Top2a | Topoisomerase (DNA) II alpha | 4.308 | 1.02E-02 | 2.204 |
| 104096_at | Orc4l | Origin recognition complex, subunit 4-like (S. Cerevisiae) | 4.288 | 1.04E-02 | 1.307 |
| 98532_at | Cdk2ap1 | CDK2 (cyclin-dependent kinase 2)-associated protein 1 | 4.263 | 1.06E-02 | 1.396 |
| 92412_s_at | Spag5 | Sperm associated antigen 5 | 4.260 | 1.07E-02 | 1.231 |
| 96726_at | Cdk8 | Cyclin-dependent kinase 8 | 4.239 | 1.09E-02 | 1.380 |
| 101065_at | Pcna | Proliferating cell nuclear antigen | 4.224 | 1.10E-02 | 2.274 |
| 160501_at | Kif20a | Kinesin family member 20A | 4.212 | 1.12E-02 | 1.758 |
| 101961_at | Bub3 | Budding uninhibited by benzimidazoles 3 homolog (S. Cerevisiae) | 4.212 | 1.12E-02 | 1.576 |
| 99522_at | Gsg2 | Germ cell-specific gene 2 | 4.187 | 1.14E-02 | 1.293 |
| 100612_at | Rrm1 | Ribonucleotide reductase M1 | 4.180 | 1.15E-02 | 1.624 |
| 93209_at | Nek4 | NIMA (never in mitosis gene a)-related expressed kinase 4 | 4.158 | 1.17E-02 | 1.203 |
| 94231_at | Ccnd1 | Cyclin D1 | 4.124 | 1.20E-02 | 2.719 |
| 100890_at | Chaf1b | Chromatin assembly factor 1, subunit B (p60) | 4.035 | 1.29E-02 | 1.439 |
| 98110_at | Mdm2 | Transformed mouse 3T3 cell double minute 2 | 3.969 | 1.37E-02 | 2.678 |
| 99073_at | Ccnf | Cyclin F | 3.951 | 1.40E-02 | 1.454 |
| 94040_at | Erh | Enhancer of rudimentary homolog (Drosophila) | 3.947 | 1.40E-02 | 1.705 |
| 94881_at | Cdkn1a | Cyclin-dependent kinase inhibitor 1A (P21) | 3.926 | 1.43E-02 | 4.435 |
| 161787_f_at | Ris2 | Retroviral integration site 2 | 3.918 | 1.44E-02 | 1.968 |
| 99632_at | Mad2l1 | MAD2 (mitotic arrest deficient, homolog)-like 1 (yeast) | 3.880 | 1.49E-02 | 2.178 |
| 160069_at | Gmnn | Geminin | 3.873 | 1.50E-02 | 2.174 |
| 97527_at | Cks2 | CDC28 protein kinase regulatory subunit 2 | 3.855 | 1.52E-02 | 1.613 |
| 100957_at | Ssbp1 | Single-stranded DNA binding protein 1 | 3.833 | 1.55E-02 | 1.597 |
| 94412_at | Cdk2 | Cyclin-dependent kinase 2 | 3.824 | 1.57E-02 | 1.509 |
| 96168_at | Kif23 | Kinesin family member 23 | 3.804 | 1.60E-02 | 1.389 |
| 96081_at | Tk1 | Thymidine kinase 1 | 3.798 | 1.60E-02 | 1.656 |
| 161172_f_at | Ncaph | Non-SMC condensin I complex, subunit H | 3.763 | 1.66E-02 | 1.345 |
| 162307_at | Polk | Polymerase (DNA directed), kappa | 3.744 | 1.69E-02 | 1.236 |
| 160952_r_at | Lrrc6 | Leucine rich repeat containing 6 (testis) | 3.725 | 1.71E-02 | 1.082 |
| 96772_at | Prim1 | DNA primase, p49 subunit | 3.666 | 1.81E-02 | 1.730 |
| 160699_at | Cdca5 | Cell division cycle associated 5 | 3.631 | 1.86E-02 | 1.685 |
| 97295_at | Cdca8 | Cell division cycle associated 8 | 3.631 | 1.86E-02 | 1.447 |
| 103797_at | Cdc7 | Cell division cycle 7 (S. Cerevisiae) | 3.606 | 1.90E-02 | 1.231 |
| 102292_at | Gadd45a | Growth arrest and DNA-damage-inducible 45 alpha | -11.255 | 2.15E-04 | 0.201* |
| 104471_at | Hdac6 | Histone deacetylase 6 | -7.709 | 1.04E-03 | 0.730* |
| 95100_at | Anapc5 | Anaphase-promoting complex subunit 5 | -6.937 | 1.64E-03 | 0.678 |
| 101483_at | Ccndbp1 | Cyclin d-type binding-protein 1 | -6.360 | 2.30E-03 | 0.678 |
| 104537_at | Rmnd1 | Required for meiotic nuclear division 1 homolog (S. cerevisiae) | -6.280 | 2.43E-03 | 0.753 |
| 104376_at | Hdac5 | Histone deacetylase 5 | -6.085 | 2.79E-03 | 0.441 |
| 95731_at | Sesn1 | Sestrin 1 | -6.038 | 2.85E-03 | 0.456 |
| 100278_at | Cdkn1b | Cyclin-dependent kinase inhibitor 1B (P27) | -5.499 | 4.08E-03 | 0.738 |
| 93421_at | Pftk1 | PFTAIRE protein kinase 1 | -5.467 | 4.15E-03 | 0.352 |
| 101969_at | Nbl1 | Neuroblastoma, suppression of tumorigenicity 1 | -5.466 | 4.16E-03 | 0.625 |
| 96710_at | H2afv | H2A histone family, member V | -5.414 | 4.27E-03 | 0.601 |
| 93539_at | Anapc13 | Anaphase promoting complex subunit 13 | -5,370 | 4,40E-03 | 0,680 |
| 103326_at | Lrrc35 | Leucine rich repeat containing 35 | -5.284 | 4.69E-03 | 0.586 |
| 101900_at | Cdkn2b | Cyclin-dependent kinase inhibitor 2B (p15, inhibits CDK4) | -5.189 | 5.05E-03 | 0.186* |
| 97908_at | Rmnd5a | Required for meiotic nuclear division 5 homolog A (S. cerevisiae) | -5.051 | 5.63E-03 | 0.556 |
| 162332_f_at | Mapre3 | Microtubule-associated protein, RP/EB family, member 3 | -4.915 | 6.23E-03 | 0.827 |
| 160545_at | Ccnd3 | Cyclin d3 | -4.854 | 6.53E-03 | 0.663 |
| 160285_at | Dhx40 | DEAH (Asp-Glu-Ala-His) box polypeptide 40 | -4.848 | 6.56E-03 | 0.627 |
| 103736_at | Sash1 | SAM and SH3 domain containing 1 | -4.605 | 7.91E-03 | 0.330 |
| 98789_at | Cdkn2a | Cyclin-dependent kinase inhibitor 2A | -4.489 | 8.68E-03 | 0.241# |
| 101930_at | Nfix | Nuclear factor I/X | -4.445 | 9.03E-03 | 0.391 |
| 99635_at | Ing4 | Inhibitor of growth family, member 4 | -4.342 | 9.91E-03 | 0.726 |
| 103501_at | Pura | Purine rich element binding protein A | -4.282 | 1.05E-02 | 0.592 |
| *Immunity and defense* | | |  |  |  |
| 98501_at | Il1rl1 | Interleukin 1 receptor-like 1 | 14.426 | 7.55E-05 | 9.700 |
| 95466_at | Cotl1 | Coactosin-like 1 (Dictyostelium) | 9.499 | 4.05E-04 | 2.636 |
| 160876_at | Bcap29 | B-cell receptor-associated protein 29 | 8.396 | 7.37E-04 | 2.043 |
| 101851_at | Cd200 | Cd200 antigen | 6.737 | 1.83E-03 | 2.427 |
| 98008_at | Cx3cl1 | Chemokine (C-X3-C motif) ligand 1 | 6.743 | 1.82E-03 | 8.834 |
| 92244_at | Exo1 | Exonuclease 1 | 5.656 | 3.67E-03 | 1.925 |
| 97574_f_at | Igh | Immunoglobulin heavy chain complex | 5.437 | 4.22E-03 | 1.109 |
| 103768_at | Ccdc86 | Coiled-coil domain containing 86 | 5.265 | 4.79E-03 | 2.151 |
| 161046_at | Crlf1 | Cytokine receptor-like factor 1 | 5.058 | 5.60E-03 | 5.869 |
| 99413_at | Ccr1 | Chemokine (C-C motif) receptor 1 | 4.647 | 7.64E-03 | 1.378 |
| 93248_at | Thyn1 | Thymocyte protein thy28 | 4.626 | 7.79E-03 | 1.262 |
| 100362_f_at | Igh | Immunoglobulin heavy chain complex | 4.519 | 8.51E-03 | 1.216 |
| 95593_at | Golph2 | Golgi phosphoprotein 2 | 4.460 | 8.91E-03 | 1.137 |
| 100718_at | Ptma | Prothymosin alpha | 4.132 | 1.19E-02 | 1.496 |
| 101346_at | Igh-6 | Immunoglobulin heavy chain 6 (heavy chain of igm) | 3.916 | 1.44E-02 | 1.301 |
| 96971_f_at | Igkv4-74 | Immunoglobulin kappa chain variable 4-74 | 3.836 | 1.55E-02 | 1.157 |
| 97783_at | Ccl17 | Chemokine (C-C motif) ligand 17 | 3.755 | 1.67E-02 | 1.181 |
| 93874_s_at | Il11ra2 | Interleukin 11 receptor, alpha chain 2 | -7.184 | 1.42E-03 | 0.193 |
| 99010_at | Islr | Immunoglobulin superfamily containing leucine-rich repeat | -6.887 | 1.69E-03 | 0.132 |
| 94761_at | Ccl7 | Chemokine (C-C motif) ligand 7 | -6.857 | 1.71E-03 | 0.271 |
| 99491_at | Il10rb | Interleukin 10 receptor, beta | -5.937 | 3.04E-03 | 0.536 |
| 97737_f_at | Nlrx1 | NLR family member X1 | -5.676 | 3.64E-03 | 0.742 |
| 103015_at | Bcl6 | B-cell leukemia/lymphoma 6 | -5.549 | 3.95E-03 | 0.464* |
| 104435_at | Igsf10 | Immunoglobulin superfamily, member 10 | -5.149 | 5.23E-03 | 0.661 |
| 99584_at | Cd82 | CD82 antigen | -5.026 | 5.72E-03 | 0.584 |
| 99992_at | Il17r | Interleukin 17 receptor | -4.935 | 6.15E-03 | 0.731 |
| 103254_at | Trafd1 | TRAF type zinc finger domain containing 1 | -4.846 | 6.57E-03 | 0.543 |
| 97490_at | Bcl7b | B-cell CLL/lymphoma 7B | -4.338 | 9.95E-03 | 0.749 |
| 101468_at | Cfp | Complement factor properdin | -4.224 | 1.10E-02 | 0.851 |
| *Response to interferon* | | |  |  |  |
| 104760_at | Ifrd2 | Interferon-related developmental regulator 2 | 9.877 | 3.41E-04 | 2.388* |
| 99475_at | Socs2 | Suppressor of cytokine signaling 2 | 6.918 | 1.65E-03 | 1.948 |
| 99975_at | Prkrir | Protein-kinase, interferon-inducible double stranded RNA dependent inhibitor, repressor of (P58 repressor) | 5.057 | 5.61E-03 | 1.590 |
| 93425_at | Irf5 | Interferon regulatory factor 5 | 4.415 | 9.26E-03 | 1.215 |
| 162143_f_at | Prkrir | Protein-kinase, interferon-inducible double stranded RNA dependent inhibitor, repressor of (P58 repressor) | 4.310 | 1.02E-02 | 1.205 |
| 162447_f_at | Mvp | Major vault protein | -6.450 | 2.18E-03 | 0.770 |
| 103432_at | Isg20 | Interferon-stimulated protein | -5.834 | 3.28E-03 | 0.358 |
| 104144_at | Gtpbp2 | GTP binding protein 2 | -5.790 | 3.38E-03 | 0.645 |
| 100423_f_at | Stat5a | Signal transducer and activator of transcription 5A | -4.870 | 6.43E-03 | 0.865 |
| *Cell adhesion and migration* | | |  |  |  |
| 99663_g_at | Nup85 | Nucleoporin 85 | 9.553 | 3.91E-04 | 1.823 |
| 99957_at | Mmp9 | Matrix metallopeptidase 9 | 8.792 | 6.20E-04 | 1.844* |
| 99062_at | Pvrl3 | Poliovirus receptor-related 3 | 8.060 | 8.58E-04 | 1.729 |
| 94724_at | Mmp10 | Matrix metallopeptidase 10 | 7.860 | 9.59E-04 | 2.542 |
| 99662_at | Nup85 | Nucleoporin 85 | 7.529 | 1.14E-03 | 2.096 |
| 94896_at | Hnrpab | Heterogeneous nuclear ribonucleoprotein A/B | 7.475 | 1.18E-03 | 2.278 |
| 97875_at | Adrm1 | Adhesion regulating molecule 1 | 7.112 | 1.49E-03 | 1.499 |
| 160777_at | Ttc7b | Tetratricopeptide repeat domain 7B | 6.769 | 1.80E-03 | 1.277 |
| 100704_at | Cmtm4 | CKLF-like MARVEL transmembrane domain containing 4 | 5.943 | 3.03E-03 | 1.114 |
| 95911_at | Hnrpab | Heterogeneous nuclear ribonucleoprotein A/B | 5.503 | 4.07E-03 | 1.419 |
| 97904_at | Actr3 | ARP3 actin-related protein 3 homolog (yeast) | 5.241 | 4.85E-03 | 2.234 |
| 96644_at | Bysl | Bystin-like | 5.050 | 5.64E-03 | 1.202 |
| 96896_at | Actr2 | ARP2 actin-related protein 2 homolog (yeast) | 4.921 | 6.20E-03 | 1.795 |
| 97822_at | Pak2 | p21 (CDKN1A)-activated kinase 2 | 4.775 | 6.92E-03 | 1.501 |
| 102939_s_at | Cd22 | CD22 antigen | 4.773 | 6.93E-03 | 1.170 |
| 92414_at | Adam12 | A disintegrin and metallopeptidase domain 12 (meltrin alpha) | 4.578 | 8.09E-03 | 2.475* |
| 100120_at | Nid1 | Nidogen 1 | 4.507 | 8.57E-03 | 2.598 |
| 99183_at | Ppp3r1 | Protein phosphatase 3, regulatory subunit B, alpha isoform (calcineurin B, type I) | 4.456 | 8.94E-03 | 1.213 |
| 96806_at | Emilin2 | Elastin microfibril interfacer 2 | 4.414 | 9.27E-03 | 1.160 |
| 95511_at | Itga6 | Integrin alpha 6 | 4.282 | 1.05E-02 | 2.237 |
| 98959_at | Memo1 | Mediator of cell motility 1 | 4.067 | 1.26E-02 | 1.314 |
| 94964_at | Vcl | Vinculin | 4.024 | 1.31E-02 | 1.921 |
| 100019_at | Vcan | Versican | 3.972 | 1.37E-02 | 2.697 |
| 98366_at | Itgav | Integrin alpha V | 3.816 | 1.58E-02 | 1.978 |
| 100065_r_at | Gja1 | Gap junction membrane channel protein alpha 1 | 3.712 | 1.73E-02 | 2.698 |
| 99014_at | Apbb1 | Amyloid beta (A4) precursor protein-binding, family B, member 1 | -9.120 | 5.19E-04 | 0.682 |
| 94561_at | Lima1 | LIM domain and actin binding 1 | -5.640 | 3.72E-03 | 0.487 |
| 101359_at | Lamb2 | Laminin, beta 2 | -5.552 | 3.94E-03 | 0.376 |
| 104743_at | Cdh13 | Cadherin 13 | -4.779 | 6.90E-03 | 0.449 |
| 96742_at | Dpt | Dermatopontin | -4.738 | 7.13E-03 | 0.073 |
| 102990_at | Col3a1 | Procollagen, type III, alpha 1 | -4.683 | 7.46E-03 | 0.282 |
| 98614_at | Nphp1 | Nephronophthisis 1 (juvenile) homolog (human) | -4.640 | 7.69E-03 | 0.765 |
| 161021_at | Pak3 | p21 (CDKN1A)-activated kinase 3 | -4.612 | 7.86E-03 | 0.628 |
| 92880_at | Mfge8 | Milk fat globule-EGF factor 8 protein | -4.593 | 7.99E-03 | 0.577 |
| 98848_at | Sh3d4 | SH3 domain protein 4 | -4.296 | 1.03E-02 | 0.638 |
| 101843_at | Sh2bpsm1 | SH2-B PH domain containing signaling mediator 1 | -4.212 | 1.11E-02 | 0.767 |
| *RNA metabolism and processing* | |  |  |  |  |
| 160141_r_at | Sfrs1 | Splicing factor, arginine/serine-rich 1 (ASF/SF2) | 13.874 | 8.01E-05 | 1.948 |
| 97166_at | Elac2 | Elac homolog 2 (E. Coli) | 13.138 | 1.05E-04 | 1.553 |
| 161060_i_at | Ddx51 | DEAD (Asp-Glu-Ala-Asp) box polypeptide 51 | 11.458 | 1.99E-04 | 1.665 |
| 103525_at | Hnrpll | Heterogeneous nuclear ribonucleoprotein L-like | 10.048 | 3.09E-04 | 1.460 |
| 98624_at | Rnpc1 | RNA-binding region (RNP1, RRM) containing 1 | 9.405 | 4.35E-04 | 1.405 |
| 102409_at | Lsm8 | LSM8 homolog, U6 small nuclear RNA associated (S. cerevisiae) | 9.101 | 5.24E-04 | 1.906 |
| 99620_at | Sfpq | Splicing factor 94raline/glutamine rich (polypyrimidine tract binding protein associated) | 9.073 | 5.40E-04 | 2.110 |
| 160071_at | Rpp30 | Ribonuclease P/MRP 30 subunit (human) | 8.569 | 6.93E-04 | 1.575 |
| 96027_at | Sf3a1 | Splicing factor 3a, subunit 1 | 8.334 | 7.57E-04 | 1.622 |
| 98923_at | Rcl1 | RNA terminal phosphate cyclase-like 1 | 8.182 | 8.17E-04 | 2.434 |
| 98973_at | Wdr43 | WD repeat domain 43 | 7.835 | 9.77E-04 | 1.973 |
| 102400_at | AU014645 | Expressed sequence AU014645 | 7.797 | 1.00E-03 | 1.410 |
| 103784_at | Gemin5 | Gem (nuclear organelle) associated protein 5 | 7.740 | 1.03E-03 | 1.829 |
| 160364_at | Sfrs10 | Splicing factor, arginine/serine-rich 10 (transformer 2 homolog, Drosophila) | 6.644 | 1.94E-03 | 1.566 |
| 99182_at | Rpp14 | Ribonuclease P 14 subunit (human) | 6.401 | 2.24E-03 | 1.323 |
| 94292_at | Strap | Serine/threonine kinase receptor associated protein | 6.349 | 2.32E-03 | 2.247 |
| 94552_at | Pcbp1 | Poly(rc) binding protein 1 | 6.282 | 2.43E-03 | 1.289 |
| 103056_at | Utp18 | UTP18, 94rali subunit (SSU) proceossome component, homolog (yeast) | 6.240 | 2.51E-03 | 2.750 |
| 94972_at | Rbms1 | RNA binding motif, single stranded interacting protein 1 | 6.225 | 2.54E-03 | 1.531 |
| 160208_at | Sf3b3 | Splicing factor 3b, subunit 3 | 6.108 | 2.75E-03 | 1.342 |
| 96297_at | Ebna1bp2 | EBNA1 binding protein 2 | 6.107 | 2.75E-03 | 2.121 |
| 94017_s_at | Sfrs2 | Splicing factor, arginine/serine-rich 2 (SC-35) | 6.071 | 2.80E-03 | 2.415 |
| 103885_at | Prpf31 | PRP31 pre-mRNA processing factor 31 homolog (yeast) | 5.973 | 2.97E-03 | 1.605 |
| 98617_at | Wbp11 | WW domain binding protein 11 | 5.915 | 3.07E-03 | 1.386 |
| 102046_at | Fusip1 | FUS interacting protein (serine-arginine rich) 1 | 5.878 | 3.17E-03 | 1.341 |
| 93342_at | Mki67ip | Mki67 (FHA domain) interacting nucleolar phosphoprotein | 5.815 | 3.33E-03 | 2.242 |
| 95650_at | Nhp2l1 | NHP2 non-histone chromosome protein 2-like 1 (S. cerevisiae) | 5.506 | 4.06E-03 | 1.415 |
| 104586_at | Sfrs1 | Splicing factor, arginine/serine-rich 1 (ASF/SF2) | 5.474 | 4.14E-03 | 2.887 |
| 98048_at | Fusip1 | FUS interacting protein (serine-arginine rich) 1 | 5.198 | 5.01E-03 | 1.706 |
| 104463_at | Utp20 | UTP20, small subunit (SSU) processome component, homolog (yeast) | 5.184 | 5.06E-03 | 1.606 |
| 101114_at | Srprb | Signal recognition particle receptor, B subunit | 5.179 | 5.08E-03 | 1.350 |
| 96083_s_at | Hnrpdl | Heterogeneous nuclear ribonucleoprotein D-like | 5.148 | 5.23E-03 | 1.489 |
| 104399_at | Cstf2 | Cleavage stimulation factor, 3’ pre-RNA subunit 2 | 5.112 | 5.38E-03 | 2.051 |
| 100917_at | Wdr55 | WD repeat domain 55 | 5.103 | 5.43E-03 | 1.712 |
| 102144_f_at | LOC100045887 | Similar to PTB-associated splicing factor | 5.083 | 5.50E-03 | 2.079 |
| 160351_at | Rnpep | Arginyl aminopeptidase (aminopeptidase B) | 4.988 | 5.91E-03 | 1.335 |
| 97249_at | Exosc5 | Exosome component 5 | 4.974 | 5.99E-03 | 1.507 |
| 100138_f_at | Rbm14 | RNA binding motif protein 14 | 4.973 | 6.00E-03 | 2.110 |
| 97200_f_at | Snrpe | Small nuclear ribonucleoprotein E | 4.917 | 6.23E-03 | 1.839 |
| 101003_at | Sfrs3 | Splicing factor, arginine/serine-rich 3 (SRp20) | 4.814 | 6.74E-03 | 1.852 |
| 99954_at | Rnu3ip2 | RNA, U3 small nucleolar interacting protein 2 | 4.780 | 6.90E-03 | 1.572 |
| 101004_f_at | Sfrs3 | Splicing factor, arginine/serine-rich 3 (SRp20) | 4.770 | 6.94E-03 | 2.076 |
| 98090_at | Krr1 | KRR1, small subunit (SSU) processome component, homolog (yeast) | 4.689 | 7.42E-03 | 1.267 |
| 160078_at | Ppp1r14b | Protein phosphatase 1, regulatory (inhibitor) subunit 14B | 4.593 | 7.99E-03 | 2.039 |
| 93518_at | Rnps1 | Ribonucleic acid binding protein S1 | 4.503 | 8.60E-03 | 1.368 |
| 95732_at | Sf3b5 | Splicing factor 3b, subunit 5 | 4.663 | 7.55E-03 | 1.613 |
| 94303_at | Hnrpd | Heterogeneous nuclear ribonucleoprotein D | 4.424 | 9.20E-03 | 1.572 |
| 94455_at | Lsm3 | LSM3 homolog, U6 small nuclear RNA associated (S. cerevisiae) | 4.389 | 9.46E-03 | 1.707 |
| 160192_at | Rbmxrt | RNA binding motif protein, X chromosome retrogene | 4.329 | 1.00E-02 | 1.669 |
| 160182_at | Sfrs6 | Splicing factor, arginine/serine-rich 6 | 4.315 | 1.01E-02 | 1.634 |
| 104212_at | Lrpprc | Leucine-rich PPR-motif containing | 4.292 | 1.04E-02 | 1.778 |
| 102796_at | Npm3 | Nucleoplasmin 3 | 4.199 | 1.13E-02 | 2.303 |
| 99621_s_at | Sfpq | Splicing factor 95raline/glutamine rich (polypyrimidine tract binding protein associated) | 4.181 | 1.14E-02 | 1.965 |
| 96245_at | Ddx56 | DEAD (Asp-Glu-Ala-Asp) box polypeptide 56 | 4.168 | 1.16E-02 | 1.515 |
| 101506_at | Snrpa1 | Small nuclear ribonucleoprotein polypeptide A’ | 4.126 | 1.20E-02 | 2.128 |
| 160722_at | Rnmtl1 | RNA methyltransferase like 1 | 4.109 | 1.21E-02 | 1.333 |
| 160531_at | Grcc2f | Gene rich cluster, C2f gene | 4.098 | 1.22E-02 | 1.666 |
| 97293_at | Rbm10 | RNA binding motif protein 10 | 4.065 | 1.26E-02 | 1.249* |
| 160791_at | 3300001P08Rik | RIKEN cDNA 3300001P08 gene | 4.050 | 1.28E-02 | 1.616 |
| 98134_at | 2610101N10Rik | RIKEN cDNA 2610101N10 gene | 4.005 | 1.33E-02 | 1.381 |
| 104766_at | Nola1 | Nucleolar protein family A, member 1 (H/ACA small nucleolar RNPs) | 3.973 | 1.37E-02 | 1.896 |
| 104470_at | Utp14a | UTP14, U3 small nucleolar ribonucleoprotein, homolog A (yeast) | 3.964 | 1.38E-02 | 1.357 |
| 95428_at | Imp4 | Imp4, u3 small nucleolar ribonucleoprotein, homolog (yeast) | 3.906 | 1.46E-02 | 1.535 |
| 101444_at | Thumpd3 | THUMP domain containing 3 | 3.894 | 1.47E-02 | 1.522 |
| 100968_at | Cstf3 | Cleavage stimulation factor, 3’ pre-RNA, subunit 3 | 3.876 | 1.50E-02 | 1.370 |
| 95756_at | Ftsj3 | Ftsj homolog 3 (E. Coli) | 3.825 | 1.56E-02 | 2.638 |
| 98077_at | Snrpd3 | Small nuclear ribonucleoprotein D3 | 3.814 | 1.58E-02 | 1.713 |
| 95913_at | Sfrs17b | Splicing factor, arginine/serine-rich 17b | 3.697 | 1.76E-02 | 1.216 |
| 93118_at | Hnrpa2b1 | Heterogeneous nuclear ribonucleoprotein A2/B1 | 3.671 | 1.80E-02 | 2.060 |
| 95677_at | Wdr57 | WD repeat domain 57 (U5 snRNP specific) | 3.628 | 1.87E-02 | 1.403 |
| 103993_at | Grcc9 | Gene rich cluster, C9 gene | -7.466 | 1.19E-03 | 0.744 |
| 96341_at | Syf2 | SYF2 homolog, RNA splicing factor (S. cerevisiae) | -6.497 | 2.12E-03 | 0.701 |
| 97536_at | Wdtc1 | WD and tetratricopeptide repeats 1 | -6.325 | 2.36E-03 | 0.627 |
| 96650_at | Auh | AU RNA binding protein/enoyl-coenzyme A hydratase | -6.161 | 2.67E-03 | 0.636 |
| 101857_at | Srpk2 | Serine/arginine-rich protein specific kinase 2 | -5.602 | 3.83E-03 | 0.515* |
| 102399_at | Rbpms | RNA binding protein gene with multiple splicing | -5.240 | 4.86E-03 | 0.382 |
| 104231_at | Hnrph3 | Heterogeneous nuclear ribonucleoprotein H3 | -4.904 | 6.29E-03 | 0.647 |
| 101507_at | Scnm1 | Sodium channel modifier 1 | -4.562 | 8.17E-03 | 0.754 |
| 92469_at | Sfrp4 | Secreted frizzled-related sequence protein 4 | -4.493 | 8.67E-03 | 0.815 |
| 103969_at | Srpk2 | Serine/arginine-rich protein specific kinase 2 | -4.261 | 1.07E-02 | 0.326* |
| *Cell development and differentiation* | | |  |  |  |
| 104614_at | Gpc1 | Glypican 1 | 20.189 | 1.83E-05 | 2.540 |
| 160119_at | Mgp | Matrix Gla protein | 15.115 | 6.63E-05 | 2.024 |
| 160162_at | Tagln2 | Transgelin 2 | 13.009 | 1.14E-04 | 3.330* |
| 93228_at | Hells | Helicase, lymphoid specific | 11.805 | 1.72E-04 | 3.639 |
| 94387_at | Spata5 | Spermatogenesis associated 5 | 9.961 | 3.20E-04 | 1.548 |
| 103876_at | Crlz1 | Charged amino acid rich leucine zipper 1 | 9.156 | 5.10E-04 | 1.376 |
| 103957_at | Tfrc | Transferrin receptor | 8.967 | 5.74E-04 | 1.926 |
| 100127_at | Crabp2 | Cellular retinoic acid binding protein II | 8.832 | 6.04E-04 | 13.577# |
| 93866_s_at | Mgp | Matrix Gla protein | 8.653 | 6.66E-04 | 20.740 |
| 101372_at | Trip13 | Thyroid hormone receptor interactor 13 | 8.102 | 8.37E-04 | 2.115 |
| 92999_at | Six4 | Sine oculis-related homeobox 4 homolog (Drosophila) | 7.980 | 9.15E-04 | 1.276 |
| 95050_at | Chordc1 | Cysteine and histidine-rich domain (CHORD)-containing, zinc-binding protein 1 | 7.681 | 1.07E-03 | 1.595* |
| 95637_at | Flnb | Filamin, beta | 7.110 | 1.49E-03 | 3.797 |
| X57349_3_at | Tfrc | Transferrin receptor | 6.925 | 1.65E-03 | 1.929 |
| 160427_at | Hrb2 | HIV-1 Rev binding protein 2 | 6.785 | 1.78E-03 | 1.703 |
| 102375_at | Smyd5 | SET and MYND domain containing 5 | 6.668 | 1.91E-03 | 1.820 |
| X57349_M_at | Tfrc | Transferrin receptor | 6.391 | 2.25E-03 | 1.415 |
| 160330_at | Chordc1 | Cysteine and histidine-rich domain (CHORD)-containing, zinc-binding protein 1 | 6.346 | 2.32E-03 | 1.456* |
| 103342_at | Eed | Embryonic ectoderm development | 6.232 | 2.53E-03 | 2.046 |
| 98890_at | 1700012G19Rik | RIKEN cDNA 1700012G19 gene | 5.595 | 3.84E-03 | 1.449 |
| 99640_at | Minpp1 | Multiple inositol polyphosphate histidine phosphatase 1 | 5.889 | 3.14E-03 | 1.988 |
| 103791_at | Narg1 | NMDA receptor-regulated gene 1 | 5.749 | 3.46E-03 | 2.129 |
| 160532_at | Tpm1 | Tropomyosin 1, alpha | 5.508 | 4.06E-03 | 1.737 |
| 101108_at | Nasp | Nuclear autoantigenic sperm protein (histone-binding) | 5.184 | 5.06E-03 | 1.753 |
| 99327_at | Klk8 | Kallikrein related-peptidase 8 | 5.053 | 5.62E-03 | 1.800 |
| 103359_at | Mettl8 | Methyltransferase like 8 | 4.954 | 6.07E-03 | 1.193 |
| 103980_at | Epha2 | Eph receptor A2 | 4.879 | 6.40E-03 | 1.859 |
| 161050_at | Nav2 | Neuron navigator 2 | 4.831 | 6.65E-03 | 2.327 |
| 92220_s_at | Bin1 | Bridging integrator 1 | 4.824 | 6.69E-03 | 1.308 |
| 93276_at | Hn1 | Hematological and neurological expressed sequence 1 | 4.706 | 7.32E-03 | 1.492 |
| 95486_at | Jmjd6 | Jumonji domain containing 6 | 4.599 | 7.94E-03 | 1.374 |
| 93246_at | Narg1 | NMDA receptor-regulated gene 1 | 4.492 | 8.67E-03 | 2.524 |
| 96781_at | Rrn3 | RRN3 RNA polymerase I transcription factor homolog (yeast) | 4.274 | 1.05E-02 | 1.624 |
| 103491_at | Nav1 | Neuron navigator 1 | 4.196 | 1.13E-02 | 1.522 |
| 99021_at | Prrx1 | Paired related homeobox 1 | 4.066 | 1.26E-02 | 2.835 |
| 103839_at | Sphk1 | Sphingosine kinase 1 | 4.040 | 1.29E-02 | 1.402 |
| 103397_at | Hrb | HIV-1 Rev binding protein | 3.918 | 1.44E-02 | 1.870 |
| 92367_at | Stil | Scl/Tal1 interrupting locus | 3.864 | 1.51E-02 | 1.149 |
| 103824_at | Wfs1 | Wolfram syndrome 1 homolog (human) | 3.783 | 1.63E-02 | 1.319 |
| 98305_at | Foxm1 | Forkhead box M1 | 3.781 | 1.63E-02 | 1.359 |
| 93550_at | Csrp2 | Cysteine and glycine-rich protein 2 | 3.763 | 1.66E-02 | 2.743 |
| 100954_at | Hrb | HIV-1 Rev binding protein | 3.715 | 1.73E-02 | 1.766 |
| 160823_at | Nedd1 | Neural precursor cell expressed, developmentally down-regulated gene 1 | 3.692 | 1.76E-02 | 1.293 |
| 93546_s_at | Cbfb | Core binding factor beta | 3,680 | 1,78E-02 | 1,404 |
| 97487_at | Serpine2 | Serine (or cysteine) peptidase inhibitor, clade E, member 2 | 3.610 | 1.90E-02 | 4.389 |
| 96152_at | Narg1 | NMDA receptor-regulated gene 1 | 3.600 | 1.92E-02 | 1.927 |
| 93066_at | Grn | Granulin | -6.846 | 1.73E-03 | 0.407 |
| 95706_at | Lgals3 | Lectin, galactose binding, soluble 3 | -5.981 | 2.96E-03 | 0.189 |
| 93643_at | Lhx9 | LIM homeobox protein 9 | -5.764 | 3.42E-03 | 0.834 |
| 102798_at | Adm | Adrenomedullin | -5.615 | 3.79E-03 | 0.189* |
| 98555_at | Ttc3 | Tetratricopeptide repeat domain 3 | -5.361 | 4.42E-03 | 0.573 |
| 102266_at | Inha | Inhibin alpha | -5.299 | 4.64E-03 | 0.881 |
| 98028_at | Twist1 | Twist gene homolog 1 (Drosophila) | -5.098 | 5.45E-03 | 0.457* |
| 94341_at | Jarid2 | Jumonji, AT rich interactive domain 2 | -4.908 | 6.26E-03 | 0.878 |
| 104590_at | Mef2c | Myocyte enhancer factor 2C | -4.725 | 7.21E-03 | 0.812 |
| 97885_at | Tmem176b | Transmembrane protein 176B | -4.700 | 7.37E-03 | 0.315 |
| 102579_f_at | Hoxa6 | Homeo box A6 | -4.653 | 7.60E-03 | 0.662 |
| 99929_at | Ext2 | Exostoses (multiple) 2 | -4.476 | 8.76E-03 | 0.820 |
| 102737_at | Edn1 | Endothelin 1 | -4.674 | 7.49E-03 | 0.774 |
| 92607_at | Mest | Mesoderm specific transcript | -4.490 | 8.68E-03 | 0.200 |
| 97474_r_at | Ptn | Pleiotrophin | -4.420 | 9.23E-03 | 0.158 |
| 97498_at | Fhl1 | Four and a half LIM domains 1 | -4.359 | 9.76E-03 | 0.309 |
| 95557_at | Bmp1 | Bone morphogenetic protein 1 | -4.255 | 1.07E-02 | 0.731 |
| 97122_at | Six5 | Sine oculis-related homeobox 5 homolog (Drosophila) | -4.217 | 1.11E-02 | 0.609 |
| *Protein biosynthesis and ribosome organization* | | |  |  |  |
| 94260_at | Larp1 | La ribonucleoprotein domain family, member 1 | 9.678 | 3.71E-04 | 1.989 |
| 97824_at | Nola2 | Nucleolar protein family A, member 2 | 8.759 | 6.34E-04 | 3.660 |
| 97443_at | Mrpl52 | Mitochondrial ribosomal protein L52 | 8.117 | 8.31E-04 | 1.389 |
| 102199_at | Tarsl1 | Threonyl-tRNA synthetase-like 1 | 7.836 | 9.75E-04 | 1.522 |
| 93488_at | Tsr1 | Tsr1, 20s rRNA accumulation, homolog (yeast) | 7.444 | 1.21E-03 | 3.687 |
| 94253_at | Eif2s1 | Eukaryotic translation initiation factor 2, subunit 1 alpha | 7.441 | 1.21E-03 | 2.692* |
| 101530_at | Eftud2 | Elongation factor Tu GTP binding domain containing 2 | 7.244 | 1.37E-03 | 1.585 |
| 97414_at | Noc4l | Nucleolar complex associated 4 homolog (S. Cerevisiae) | 6.788 | 1.77E-03 | 1.720 |
| 93929_s_at | Mrpplf3 | Mitogen regulated protein, proliferin 3 | 6.682 | 1.88E-03 | 14.704 |
| 96296_at | Mrpl15 | Mitochondrial ribosomal protein L15 | 6.642 | 1.94E-03 | 1.572 |
| 99140_at | Mrpl16 | Mitochondrial ribosomal protein L16 | 6,602 | 2,00E-03 | 1,976 |
| 94494_at | Farslb | Phenylalanine-tRNA synthetase-like, beta subunit | 6,360 | 2,30E-03 | 2,326 |
| 94875_at | Mrpl20 | Mitochondrial ribosomal protein L20 | 6,082 | 2,80E-03 | 1,549 |
| 160431_at | Mrpl12 | Mitochondrial ribosomal protein L12 | 6,069 | 2,81E-03 | 1,624 |
| 98605_at | Wars | Tryptophanyl-tRNA synthetase | 5,950 | 3,02E-03 | 1,553 |
| 160226_at | Gfm1 | G elongation factor 1 | 5.545 | 3.96E-03 | 1.336 |
| 160847_at | Trnt1 | tRNA nucleotidyl transferase, CCA-adding, 1 | 5.395 | 4.32E-03 | 1.530 |
| 104702_at | Wdr46 | WD repeat domain 46 | 5.334 | 4.51E-03 | 1.653 |
| 160990_r_at | Rwdd4a | RWD domain containing 4A | 5.205 | 4.99E-03 | 1.602 |
| 160341_at | Jtv1 | JTV1 gene | 5.110 | 5.40E-03 | 1.676 |
| 94363_at | Bms1l | BMS1-like, ribosome assembly protein (yeast) | 5.057 | 5.61E-03 | 1.421 |
| 160231_at | Farsla | Phenylalanine-tRNA synthetase-like, alpha subunit | 4.820 | 6.71E-03 | 1.484 |
| 93752_at | Iars | Isoleucine-tRNA synthetase | 4.779 | 6.90E-03 | 1.350 |
| 93270_at | Gars | Glycyl-tRNA synthetase | 4.703 | 7.35E-03 | 1.523 |
| 104234_at | Mrps25 | Mitochondrial ribosomal protein S25 | 4.527 | 8.44E-03 | 1.410 |
| 103926_at | Eif4g1 | Eukaryotic translation initiation factor 4, gamma 1 | 4.253 | 1.07E-02 | 1.621 |
| 93973_at | Eif3s9 | Eukaryotic translation initiation factor 3, subunit 9 (eta) | 4.236 | 1.09E-02 | 1.489 |
| 104037_at | Rwdd4a | RWD domain containing 4A | 4.231 | 1.10E-02 | 1.502 |
| 160283_at | Rsl1d1 | Ribosomal L1 domain containing 1 | 4.223 | 1.10E-02 | 1.737 |
| 93058_at | Eif1a | Eukaryotic translation initiation factor 1A | 4,200 | 1,13E-02 | 1,991 |
| 93787_f_at | Mrpl18 | Mitochondrial ribosomal protein L18 | 4.173 | 1.15E-02 | 1.542 |
| 96335_at | Mrpl38 | Mitochondrial ribosomal protein L38 | 3.959 | 1.39E-02 | 1.284 |
| 96604_at | Dus1l | Dihydrouridine synthase 1-like (S. Cerevisiae) | 3.935 | 1.42E-02 | 1.222 |
| 94250_at | Eif3s10 | Eukaryotic translation initiation factor 3, subunit 10 (theta) | 3.917 | 1.44E-02 | 1.749 |
| 101634_at | Npm1 | Nucleophosmin 1 | 3.895 | 1.47E-02 | 1.524 |
| 162433_f_at | Nol5a | Nucleolar protein 5A | 3.885 | 1.49E-02 | 1.458 |
| 97422_at | Iars2 | Isoleucine-tRNA synthetase 2, mitochondrial | 3.801 | 1.60E-02 | 1.389 |
| 95054_at | Tars | Threonyl-tRNA synthetase | 3.799 | 1.60E-02 | 1.456* |
| 95159_at | Mrps18b | Mitochondrial ribosomal protein S18B | 3.797 | 1.61E-02 | 1.588 |
| 101409_at | Lgtn | Ligatin | 3.694 | 1.76E-02 | 1.272* |
| 96693_at | Rars | Arginyl-tRNA synthetase | 3.618 | 1.88E-02 | 1.662 |
| 103601_at | Ptrh1 | Peptidyl-tRNA hydrolase 1 homolog (S. cerevisiae) | 3.613 | 1.89E-02 | 1.216 |
| 99599_s_at | Ptov1 | Prostate tumor over expressed gene 1 | -7.285 | 1.34E-03 | 0.643 |
| 101213_at | Arbp | Acidic ribosomal phosphoprotein P0 | -6.214 | 2.56E-03 | 0.832 |
| 94952_at | Igf2bp2 | Insulin-like growth factor 2 mRNA binding protein 2 | -4.840 | 6.60E-03 | 0.653 |
| 92857_at | Rpl22 | Ribosomal protein L22 | -4.664 | 7.54E-03 | 0.720 |
| 98936_at | Sars1 | Seryl-aminoacyl-tRNA synthetase 1 | -4.576 | 8.12E-03 | 0.825 |
| *Apoptosis* | |  |  |  |  |
| 104154_at | Trp53 | Transformation related protein 53 | 11.527 | 1.88E-04 | 1.707 |
| 97825_at | Perp | PERP, TP53 apoptosis effector | 9.916 | 3.29E-04 | 12.262 |
| 98056_at | Phlda3 | Pleckstrin homology-like domain, family A, member 3 | 8.724 | 6.43E-04 | 3.904 |
| 102887_at | Tnfrsf11b | Tumor necrosis factor receptor superfamily, member 11b (osteoprotegerin) | 7.290 | 1.33E-03 | 4.333 |
| 103514_at | Tnfrsf21 | Tumor necrosis factor receptor superfamily, member 21 | 5.688 | 3.62E-03 | 1.480 |
| 104275_g_at | Trp53 | Transformation related protein 53 | 5.348 | 4.46E-03 | 1.616 |
| 99392_at | Tnfaip3 | Tumor necrosis factor, alpha-induced protein 3 | 5.302 | 4.63E-03 | 2.270 |
| 100442_at | Tbrg4 | Transforming growth factor beta regulated gene 4 | 4.704 | 7.34E-03 | 1.710 |
| 98499_s_at | Casp7 | Caspase 7 | 4.629 | 7.75E-03 | 1.468 |
| 99027_at | Bcl2l1 | Bcl2-like 1 | 4.578 | 8.09E-03 | 1.552 |
| 160268_at | Pdcl3 | Phosducin-like 3 | 4.498 | 8.63E-03 | 1.330 |
| 99070_at | Chuk | Conserved helix-loop-helix ubiquitous kinase | 4.439 | 9.07E-03 | 1.801 |
| 101957_f_at | Parp1 | Poly (ADP-ribose) polymerase family, member 1 | 4.304 | 1.02E-02 | 1.563 |
| 94861_at | 4930453N24Rik | RIKEN cDNA 4930453N24 gene | 4.288 | 1.04E-02 | 1.219 |
| 95010_at | Traf3 | Tnf receptor-associated factor 3 | 4.152 | 1.17E-02 | 1.440 |
| 104291_at | Casp8ap2 | Caspase 8 associated protein 2 | 4.049 | 1.28E-02 | 1.105 |
| 101617_s_at | Trp53 | Transformation related protein 53 | 3.987 | 1.35E-02 | 1.406 |
| 160829_at | Phlda1 | Pleckstrin homology-like domain, family A, member 1 | 3.962 | 1.38E-02 | 4.882 |
| 101035_at | Api5 | Apoptosis inhibitor 5 | 3.837 | 1.55E-02 | 1.530 |
| 96858_at | Pdcd8 | Programmed cell death 8 | 3.755 | 1.67E-02 | 1.451 |
| 104322_at | Ckap2 | Cytoskeleton associated protein 2 | 3.640 | 1.85E-02 | 2.150 |
| 93968_at | Pdcd5 | Programmed cell death 5 | 3.625 | 1.87E-02 | 1.359 |
| 95341_at | Tbrg4 | Transforming growth factor beta regulated gene 4 | 3.586 | 1.95E-02 | 1.279 |
| 96765_at | Peg3 | Paternally expressed 3 | -7.388 | 1.25E-03 | 0.052* |
| 98424_at | Ptpn13 | Protein tyrosine phosphatase, non-receptor type 13 | -7.158 | 1.46E-03 | 0.342* |
| 100587_f_at | 5730403B10Rik | RIKEN cDNA 5730403B10 gene | -5.639 | 3.73E-03 | 0.700 |
| 101451_at | Peg3 | Paternally expressed 3 | -5.561 | 3.91E-03 | 0.134* |
| 95660_at | Ethe1 | Ethylmalonic encephalopathy 1 | -4.440 | 9.06E-03 | 0.524 |
| 103338_at | Parp6 | Poly (ADP-ribose) polymerase family, member 6 | -4.368 | 9.66E-03 | 0.608 |
| 99033_at | Itpk1 | Inositol 1,3,4-triphosphate 5/6 kinase | -4.312 | 1.02E-02 | 0.872 |
| 103029_at | Pdcd4 | Programmed cell death 4 | -4.250 | 1.08E-02 | 0.398 |
| 102860_at | Serpina3g | Serine (or cysteine) peptidase inhibitor, clade A, member 3G | -4.212 | 1.12E-02 | 0.411 |
| *Ubiquitin cycle* | |  |  |  |  |
| 102970_at | Psmc3ip | Proteasome (prosome, macropain) 26S subunit, atpase 3, interacting protein | 8.050 | 8.67E-04 | 1.742 |
| 103906_f_at | Nedd4l | Neural precursor cell expressed, developmentally down-regulated gene 4-like | 7.173 | 1.43E-03 | 1.420 |
| 95703_at | Uble1a | Ubiquitin-like 1 (sentrin) activating enzyme E1A | 7.161 | 1.45E-03 | 1.497 |
| 99564_at | Uhrf1 | Ubiquitin-like, containing PHD and RING finger domains, 1 | 7.107 | 1.50E-03 | 2.922 |
| 93971_f_at | Psmd12 | Proteasome (prosome, macropain) 26S subunit, non-atpase, 12 | 6.368 | 2.29E-03 | 1.878 |
| 100955_at | Ube2t | Ubiquitin-conjugating enzyme E2T (putative) | 6.284 | 2.43E-03 | 2.838 |
| 95742_at | Psmd13 | Proteasome (prosome, macropain) 26S subunit, non-atpase, 13 | 5.469 | 4.15E-03 | 1.434 |
| 99149_at | Trim59 | Tripartite motif-containing 59 | 5.335 | 4.51E-03 | 1.653 |
| 160110_at | Wwp2 | WW domain containing E3 ubiquitin protein ligase 2 | 4.962 | 6.04E-03 | 1.522 |
| 160605_s_at | Usp38 | Ubiquitin specific peptidase 38 | 4.938 | 6.13E-03 | 1.535 |
| 96701_at | Uble1b | Ubiquitin-like 1 (sentrin) activating enzyme E1B | 4.862 | 6.47E-03 | 1.759 |
| 92290_at | Siah1b | Seven in absentia 1B | 4.813 | 6.75E-03 | 1.235 |
| 99102_at | Usp9x | Ubiquitin specific peptidase 9, X chromosome | 4.793 | 6.82E-03 | 1.459 |
| 96959_at | Ube2n | Ubiquitin-conjugating enzyme E2N | 4.720 | 7.23E-03 | 1.863 |
| 160491_at | Ube2f | Ubiquitin-conjugating enzyme E2F (putative | 4.576 | 8.12E-03 | 1.215 |
| 99575_at | Ubqln1 | Ubiquilin 1 | 4.556 | 8.21E-03 | 1.649 |
| 96061_at | Usp14 | Ubiquitin specific peptidase 14 | 4.405 | 9.34E-03 | 2.230 |
| 160234_at | Usp1 | Ubiquitin specific peptdiase 1 | 4.386 | 9.49E-03 | 2.235 |
| 103350_at | Psmd7 | Proteasome (prosome, macropain) 26S subunit, non-atpase, 7 | 4.358 | 9.77E-03 | 1.605 |
| 98047_at | 5730410I19Rik | RIKEN cDNA 5730410I19 gene | 4.309 | 1.02E-02 | 1.376 |
| 94865_at | Ublcp1 | Ubiquitin-like domain containing CTD phosphatase 1 | 4.081 | 1.24E-02 | 1.428 |
| 98558_r_at | Psmb4 | Proteasome (prosome, macropain) subunit, beta type 4 | 4.007 | 1.33E-02 | 1.088 |
| 104287_at | Senp3 | SUMO/sentrin specific peptidase 3 | 3.993 | 1.35E-02 | 1.741 |
| 101741_at | Psmb5 | Proteasome (prosome, macropain) subunit, beta type 5 | 3.892 | 1.48E-02 | 1.371 |
| 103717_at | Wwp2 | WW domain containing E3 ubiquitin protein ligase 2 | 3.878 | 1.49E-02 | 1.534 |
| 103861_s_at | Ubfd1 | Ubiquitin family domain containing 1 | 3.860 | 1.52E-02 | 1.408 |
| 95448_at | Psmc2 | Proteasome (prosome, macropain) 26S subunit, atpase 2 | 3.771 | 1.65E-02 | 1.443 |
| 97220_at | Dscr2 | Down syndrome critical region homolog 2 (human) | 3.708 | 1.74E-02 | 1.721 |
| 100512_at | Uchl5 | Ubiquitin carboxyl-terminal esterase L5 | 3.682 | 1.78E-02 | 1.918 |
| 160534_at | Psmc5 | Protease (prosome, macropain) 26S subunit, atpase 5 | 3.607 | 1.90E-02 | 1.213 |
| 99085_at | Usp3 | Ubiquitin specific peptidase 3 | -7.999 | 9.04E-04 | 0.586 |
| 96286_at | Ubap1 | Ubiquitin-associated protein 1 | -6.486 | 2.14E-03 | 0.822 |
| 100529_at | Ube2h | Ubiquitin-conjugating enzyme E2H | -6.264 | 2.46E-03 | 0.568 |
| 99086_g_at | Usp3 | Ubiquitin specific peptidase 3 | -5.445 | 4.19E-03 | 0.650 |
| 93101_s_at | Nedd4 | Neural precursor cell expressed, developmentally down-regulted gene 4 | -5.118 | 5.35E-03 | 0.461* |
| 97285_f_at | Ubxd1 | UBX domain containing 1 | -4.974 | 5.99E-03 | 0.672 |
| 101069_g_at | Mkrn1 | Makorin, ring finger protein, 1 | -4.912 | 6.25E-03 | 0.785 |
| 104348_at | Bre | Brain and reproductive organ-expressed protein | -4.768 | 6.94E-03 | 0.717 |
| 93966_at | Ube4b | Ubiquitination factor E4B, UFD2 homolog (S. Cerevisiae) | -4.667 | 7.54E-03 | 0.735 |
| 101070_at | Mkrn1 | Makorin, ring finger protein, 1 | -4.650 | 7.61E-03 | 0.657 |
| 162125_f_at | Ubc | Ubiquitin C | -4.630 | 7.74E-03 | 0.666 |
| 97171_f_at | Ube2l6 | Ubiquitin-conjugating enzyme E2L 6 | -4.608 | 7.89E-03 | 0.755 |
| 104109_at | Fbxo21 | F-box only protein 21 | -4.524 | 8.46E-03 | 0.583 |
| 102279_at | Ube1l | Ubiquitin-activating enzyme E1-like | -4.426 | 9.16E-03 | 0.541 |
| 161814_f_at | Rnf19 | Ring finger protein (C3HC4 type) 19 | -4.338 | 9.95E-03 | 0.834 |
| 96148_at | Senp6 | SUMO/sentrin specific peptidase 6 | -4.220 | 1.11E-02 | 0.787 |
| *Electron transport and energy production* | | |  |  |  |
| 96878_at | Cyb5b | Cytochrome b5 type B | 12.931 | 1.19E-04 | 2.117 |
| 94526_at | Cisd1 | CDGSH iron sulfur domain 1 | 7.586 | 1.12E-03 | 1.973 |
| 94207_at | Pdia6 | Protein disulfide isomerase associated 6 | 7.343 | 1.28E-03 | 2.941 |
| 160339_at | Pycr2 | Pyrroline-5-carboxylate reductase family, member 2 | 7.246 | 1.37E-03 | 2.400 |
| 92388_at | Cox18 | COX18 cytochrome c oxidase assembly homolog (S. cerevisiae) | 6.982 | 1.61E-03 | 1.488 |
| 97270_at | Nxn | Nucleoredoxin | 5.587 | 3.86E-03 | 1.107 |
| 103619_at | Cyb5b | Cytochrome b5 type B | 5.238 | 4.87E-03 | 1.386 |
| 94242_at | Txndc17 | Thioredoxin domain containing 17 | 4.238 | 1.09E-02 | 2.029 |
| 162417_at | 1500001M20Rik | RIKEN cDNA 1500001M20 gene | 4.190 | 1.14E-02 | 1.256 |
| 100581_at | Cstb | Cystatin B | 4.103 | 1.22E-02 | 1.310 |
| 94208_at | Pdia6 | Protein disulfide isomerase associated 6 | 4.035 | 1.29E-02 | 2.167 |
| 94209_g_at | Pdia6 | Protein disulfide isomerase associated 6 | 3.995 | 1.34E-02 | 2.311 |
| 96256_at | Prdx3 | Peroxiredoxin 3 | 3.980 | 1.36E-02 | 1.707 |
| 160230_at | Cox17 | Cytochrome c oxidase, subunit XVII assembly protein homolog (yeast) | 3.674 | 1.79E-02 | 1.634 |
| 92898_at | Cyp7b1 | Cytochrome P450, family 7, subfamily b, polypeptide 1 | 3.657 | 1.82E-02 | 1.545 |
| 95026_at | Retsat | Retinol saturase (all trans retinol 13,14 reductase) | 3.621 | 1.88E-02 | 1.243 |
| 104153_at | Ivd | Isovaleryl coenzyme A dehydrogenase | -7.311 | 1.31E-03 | 0.621 |
| 160194_at | Gcdh | Glutaryl-Coenzyme A dehydrogenase | -6.436 | 2.20E-03 | 0.750 |
| 92587_at | Fdx1 | Ferredoxin 1 | -5.820 | 3.32E-03 | 0.589 |
| 100059_at | Cyba | Cytochrome b-245, alpha polypeptide | -5.597 | 3.83E-03 | 0.197 |
| 160383_at | Cox7a2l | Cytochrome c oxidase subunit viia polypeptide 2-like | -5.470 | 4.14E-03 | 0.627 |
| 97492_at | Txndc12 | Thioredoxin domain containing 12 (endoplasmic reticulum) | -5.373 | 4.40E-03 | 0.654 |
| 160088_at | Fmo5 | Flavin containing monooxygenase 5 | -5.304 | 4.61E-03 | 0.769 |
| 104011_at | Aox1 | Aldehyde oxidase 1 | -4.746 | 7.09E-03 | 0.785 |
| 101031_at | Surf1 | Surfeit gene 1 | -4.718 | 7.24E-03 | 0.604 |
| 160237_at | Ndufa6 | NADH dehydrogenase (ubiquinone) 1 alpha subcomplex, 6 (B14) | -4.658 | 7.57E-03 | 0.349 |
| 98533_at | Cyb5 | Cytochrome b-5 | -4.604 | 7.92E-03 | 0.380* |
| 160611_at | Cyp4v3 | Cytochrome P450, family 4, subfamily v, polypeptide 3 | -4.435 | 9.09E-03 | 0.426 |
| 94831_at | Ctsb | Cathepsin B | -4.303 | 1.02E-02 | 0.515 |
| *Cytoskeleton organization and biogenesis* | | |  |  |  |
| 103281_at | Cd2ap | CD2-associated protein | 7.198 | 1.41E-03 | 1.461 |
| 101029_f_at | Actc1 | Actin, alpha, cardiac | 6.042 | 2.84E-03 | 1.673 |
| 98107_at | Coro1c | Coronin, actin binding protein 1C | 5.691 | 3.60E-03 | 2.054 |
| 96033_at | Sdc1 | Syndecan 1 | 5.303 | 4.62E-03 | 2.322 |
| 95628_at | Diap3 | Diaphanous homolog 3 (Drosophila) | 4.919 | 6.22E-03 | 1.473 |
| 97302_at | Ivns1abp | Influenza virus NS1A binding protein | 4.701 | 7.36E-03 | 2.708 |
| 160094_at | Arpc4 | Actin related protein 2/3 complex, subunit 4 | 4.302 | 1.03E-02 | 1.495 |
| 100381_at | Acta1 | Actin, alpha 1, skeletal muscle | 4.079 | 1.25E-02 | 1.274 |
| 93100_at | Acta2 | Actin, alpha 2, smooth muscle, aorta | 3.966 | 1.38E-02 | 1.574 |
| 160320_at | Sorbs1 | Sorbin and SH3 domain containing 1 | 3.797 | 1.61E-02 | 2.701 |
| 98454_at | Palm | Paralemmin | -7.785 | 1.01E-03 | 0.737 |
| 94863_r_at | Dncl2a | Dynein, cytoplasmic, light chain 2A | -5.193 | 5.03E-03 | 0.682 |
| 103345_at | Spna2 | Spectrin alpha 2 | -4.694 | 7.39E-03 | 0.557 |
| 92953_at | Fmn1 | Formin 1 | -4.655 | 7.59E-03 | 0.725 |
| 94713_at | Myo7a | Myosin VIIa | -4.617 | 7.84E-03 | 0.686 |
| 160932_at | Nck1 | Non-catalytic region of tyrosine kinase adaptor protein 1 | -4.525 | 8.46E-03 | 0.856 |
| *Cell growth and proliferation* | | |  |  |  |
| 103220_at | Ndnl2 | Necdin-like 2 | 9.359 | 4.55E-04 | 1.688 |
| 160788_at | Pes1 | Pescadillo homolog 1, containing BRCT domain (zebrafish) | 8.332 | 7.60E-04 | 1.548 |
| 93294_at | Ctgf | Connective tissue growth factor | 8.327 | 7.64E-04 | 10.675 |
| 93883_at | Prl2c2 | Prolactin family 2, subfamily c, member 2 | 6.477 | 2.15E-03 | 7.339 |
| 95137_at | Tmem97 | Transmembrane protein 97 | 6.227 | 2.54E-03 | 3.196 |
| 92777_at | Cyr61 | Cysteine rich protein 61 | 6.179 | 2.64E-03 | 2.622 |
| 99537_at | Ruvbl1 | RuvB-like protein 1 | 6.056 | 2.83E-03 | 1.729 |
| 94932_at | Pdgfa | Platelet derived growth factor, alpha | 4.519 | 8.51E-03 | 1.761 |
| 160269_at | Gfer | Growth factor, erv1 (S. Cerevisiae)-like (augmenter of liver regeneration) | 4.374 | 9.61E-03 | 1.482 |
| 160422_at | Ruvbl2 | RuvB-like protein 2 | 4.295 | 1.03E-02 | 1.743 |
| 103413_at | Pwp1 | PWP1 homolog (S. Cerevisiae) | 4.292 | 1.04E-02 | 1.556 |
| 96632_at | Morf4l2 | Mortality factor 4 like 2 | 4.153 | 1.17E-02 | 1.243 |
| 97345_at | Anp32e | Acidic (leucine-rich) nuclear phosphoprotein 32 family, member E | 4.006 | 1.33E-02 | 1.778 |
| 94838_r_at | Prl2c2 | Prolactin family 2, subfamily c, member 2 | 3.598 | 1.92E-02 | 4.660 |
| 93780_at | Them2 | Thioesterase superfamily member 2 | -9.381 | 4.44E-04 | 0.690 |
| 96596_at | Ndrg1 | N-myc downstream regulated gene 1 | -7.736 | 1.03E-03 | 0.158 |
| 96793_at | Dmap1 | DNA methyltransferase 1-associated protein 1 | -7.018 | 1.58E-03 | 0.732 |
| 97951_s_at | Tsc2 | Tuberous sclerosis 2 | -6.994 | 1.60E-03 | 0.650 |
| 93284_at | Cirbp | Cold inducible RNA binding protein | -6.694 | 1.87E-03 | 0.732 |
| 160464_s_at | Ndrg1 | N-myc downstream regulated gene 1 | -6.539 | 2.08E-03 | 0.185 |
| 97429_at | Snrk | SNF related kinase | -5.260 | 4.80E-03 | 0.495 |
| 104390_at | Anp32a | Acidic (leucine-rich) nuclear phosphoprotein 32 family, member A | -4.614 | 7.85E-03 | 0.685 |
| *Angiogenesis* | | |  |  |  |
| 103520_at | Vegfa | Vascular endothelial growth factor A | 5.890 | 3.13E-03 | 3.026 |
| 160484_at | Rtn4 | Reticulon 4 | 5.328 | 4.54E-03 | 1.758 |
| 93216_at | Fgf2 | Fibroblast growth factor 2 | 4.898 | 6.33E-03 | 1.241 |
| 95016_at | Nrp1 | Neuropilin 1 | 4.300 | 1.03E-02 | 1.620 |
| 92560_g_at | Vcam1 | Vascular cell adhesion molecule 1 | -12.432 | 1.28E-04 | 0.354 |
| 92559_at | Vcam1 | Vascular cell adhesion molecule 1 | -7.180 | 1.42E-03 | 0.290 |
| 92365_at | Figf | C-fos induced growth factor | -5.646 | 3.70E-03 | 0.084* |
| 92558_at | Vcam1 | Vascular cell adhesion molecule 1 | -4.940 | 6.12E-03 | 0.360 |
| *Proteolysis and peptidolysis* | | |  |  |  |
| 97772_at | Plau | Plasminogen activator, urokinase | 7.046 | 1.56E-03 | 1.242 |
| 160655_at | Cpd | Carboxypeptidase D | 5.090 | 5.48E-03 | 1.352 |
| 160290_at | Ide | Insulin degrading enzyme | 4.441 | 9.06E-03 | 1.653 |
| 104025_at | Thop1 | Thimet oligopeptidase 1 | 4.431 | 9.12E-03 | 2.196 |
| 97258_at | Lactb2 | Lactamase, beta 2 | 4.368 | 9.66E-03 | 1.475 |
| 100983_at | Prep | Prolyl endopeptidase | 3.905 | 1.46E-02 | 1.541 |
| 94923_f_at | Otud4 | OTU domain containing 4 | 3.807 | 1.59E-02 | 1.775 |
| 93039_at | Pgcp | Plasma glutamate carboxypeptidase | -8.201 | 8.08E-04 | 0.149 |
| 96211_at | Dpp8 | Dipeptidylpeptidase 8 | -6.740 | 1.83E-03 | 0.487 |
| 94365_at | Hint2 | Histidine triad nucleotide binding protein 2 | -6.163 | 2.66E-03 | 0.729 |
| 104036_at | Dpp7 | Dipeptidylpeptidase 7 | -5.303 | 4.62E-03 | 0.429 |
| 93261_at | Lgmn | Legumain | -5.035 | 5.68E-03 | 0.361 |
| 160328_at | Lonp1 | Lon peptidase 1, mitochondrial | -4.606 | 7.90E-03 | 0.644 |
| 102316_at | Capn5 | Calpain 5 | -4.309 | 1.02E-02 | 0.537 |
| *Microtubule dynamics* | |  |  |  |  |
| 96298_f_at | Dnclc1 | Dynein, cytoplasmic, light chain 1 | 9.983 | 3.13E-04 | 1.650 |
| 160462_f_at | Tubb3 | Tubulin, beta 3 | 4.554 | 8.24E-03 | 2.202 |
| 160461_f_at | Tubb6 | Tubulin, beta 6 | 4.037 | 1.29E-02 | 2.661 |
| 92693_at | Nin | Ninein | 3.772 | 1.64E-02 | 1.255 |
| 95135_at | Mid1ip1 | Mid1 interacting protein 1 (gastrulation specific G12-like (zebrafish)) | -4.253 | 1.07E-02 | 0.179 |
| *Protein folding* | |  |  |  |  |
| 100352_at | Hspa4 | Heat shock protein 4 | 9.141 | 5.15E-04 | 1.667* |
| 100353_g_at | Hspa4 | Heat shock protein 4 | 8.009 | 8.95E-04 | 2.178* |
| 92571_at | Hspa4 | Heat shock protein 4 | 7.682 | 1.07E-03 | 3.449* |
| 101562_at | Hspa14 | Heat shock 70kda protein 14 | 7.065 | 1.53E-03 | 1.361 |
| 96254_at | Dnajb1 | DnaJ (Hsp40) homolog, subfamily B, member 1 | 6.512 | 2.10E-03 | 1.614 |
| 161745_f_at | Hspa4 | Heat shock protein 4 | 6.488 | 2.13E-03 | 1.693* |
| 92829_at | Hspe1 | Heat shock protein 1 (chaperonin 10) | 6.373 | 2.28E-03 | 1.977 |
| 160562_at | Cct7 | Chaperonin subunit 7 (eta) | 5.662 | 3.66E-03 | 1.619 |
| 160203_at | Dnajc9 | DnaJ (Hsp40) homolog, subfamily C, member 9 | 5.632 | 3.75E-03 | 2.129 |
| 160395_at | Nudcd2 | Nudc domain containing 2 | 5.507 | 4.06E-03 | 2.233 |
| 99082_at | Fkbp10 | FK506 binding protein 10 | 5.282 | 4.71E-03 | 1.595 |
| 96594_at | Hspa4 | Heat shock protein 4 | 5.022 | 5.74E-03 | 2.571* |
| 101890_f_at | Dnajc2 | DnaJ (Hsp40) homolog, subfamily C, member 2 | 4.930 | 6.17E-03 | 2.163 |
| 104738_at | Dnajc2 | DnaJ (Hsp40) homolog, subfamily C, member 2 | 4.921 | 6.21E-03 | 1.856 |
| 102414_i_at | Dnajc3 | DnaJ (Hsp40) homolog, subfamily C, member 3 | 3.834 | 1.55E-02 | 1.465 |
| 160456_at | Ppih | Peptidyl prolyl isomerase H | 3.594 | 1.93E-02 | 1.133 |
| 160589_at | Ppig | Peptidyl-prolyl isomerase G (cyclophilin G) | -5.889 | 3.13E-03 | 0.719 |
| 95112_f_at | Ppil2 | Peptidylprolyl isomerase (cyclophilin)-like 2 | -4.536 | 8.38E-03 | 0.742 |
| 95110_at | Ppil2 | Peptidylprolyl isomerase (cyclophilin)-like 2 | -4.336 | 9.97E-03 | 0.741 |
| *Blood coagulation* | |  |  |  |  |
| 92978_s_at | Serpinb2 | Serine (or cysteine) peptidase inhibitor, clade B, member 2 | 19.356 | 2.52E-05 | 59.448 |
| 101816_at | Serpinb3a | Serine (or cysteine) peptidase inhibitor, clade B (ovalbumin), member 3A | 4.346 | 9.87E-03 | 1.158 |
| 97529_at | Anxa8 | Annexin A8 | 3.909 | 1.45E-02 | 4.212 |
| 95355_at | Agtrap | Angiotensin II, type I receptor-associated protein | -6.457 | 2.17E-03 | 0.562 |
| *DNA repair* | |  |  |  |  |
| 102001_at | Rrm2 | Ribonucleotide reductase M2 | 10.427 | 2.75E-04 | 6.414 |
| 102792_at | Ung | Uracil DNA glycosylase | 8.684 | 6.50E-04 | 1.619 |
| 102976_at | Brca1 | Breast cancer 1 | 6.134 | 2.72E-03 | 1.783 |
| 102911_at | Brca2 | Breast cancer 2 | 5.855 | 3.22E-03 | 1.360 |
| 93138_at | 2410012H22Rik | RIKEN cDNA 2410012H22 gene | 5.335 | 4.51E-03 | 1.835 |
| 101180_at | Atm | Ataxia telangiectasia mutated homolog (human) | 5.029 | 5.70E-03 | 1.925 |
| 103565_at | 1810009A15Rik | RIKEN cDNA 1810009A15 gene | 3.937 | 1.42E-02 | 2.238 |
| 102853_at | Smc3 | Structural maintenance of chromosomes 3 | 3.617 | 1.88E-02 | 1.297 |
| 101540_at | Tdg | Thymine DNA glycosylase | 3.590 | 1.94E-02 | 1.817 |
| *Autophagy* | |  |  |  |  |
| 100931_at | Arsa | Arylsulfatase A | -4.321 | 1.01E-02 | 0.264* |
| 95138_at | Wipi2 | WD repeat domain, phosphoinositide interacting 2 | -4.254 | 1.07E-02 | 0.711 |
| *Nuclear stability, chromatin structure and gene expression* | | |  |  |  |
| 101414_at | Lmnb2 | Lamin B2 | 3.882 | 1.49E-02 | 1.571 |
| 101459_at | Chd1 | Chromodomain helicase DNA binding protein 1 | 3.728 | 1.70E-02 | 1.534 |
| *Response to stress* | | |  |  |  |
| 95520_at | Tmbib1 | Transmembrane BAX inhibitor motif containing 1 | 8.150 | 8.21E-04 | 1.794 |
| 100081_at | Stip1 | Stress-induced phosphoprotein 1 | 3.618 | 1.88E-02 | 1.530 |
| *Synaptic transmission* | |  |  |  |  |
| 93586_at | Syngr2 | Synaptogyrin 2 | 9.697 | 3.66E-04 | 1.723 |
| 93011_at | Gabarapl1 | Gamma-aminobutyric acid (GABA(A)) receptor-associated protein-like 1 | -4.690 | 7.41E-03 | 0.361 |
| *GPI anchor biosynthetic process* | |  |  |  |  |
| 160444_at | Pigx | Phosphatidylinositol glycan, class X | -8.611 | 6.82E-04 | 0.846 |
| *Peroxisome organization and biogenesis* | | |  |  |  |
| 99469_at | Pex6 | Peroxisomal biogenesis factor 6 | -5.292 | 4.67E-03 | 0.702 |
| *Protein modification* | |  |  |  |  |
| 95057_at | Herpud1 | Homocysteine-inducible, endoplasmic reticulum stress-inducible, ubiquitin-like domain member 1 | -4.769 | 6.94E-03 | 0.635 |
| *Spermidine biosynthetic process* | |  |  |  |  |
| 92540_f_at | Srm | Spermidine synthase | 5.343 | 4.48E-03 | 2.690* |
| *Unknown* | |  |  |  |  |
| 99366_at | Pqlc3 | PQ loop repeat containing | 8.086 | 8.47E-04 | 2.631 |
| 98432_at | X99384 | cDNA sequence X99384 | 8.018 | 8.85E-04 | 1.329 |
[truncated: 7,617 more chars]
